# Remote Amino Acid Recognition Enables Effective Hydrogen Peroxide Activation at a Manganese Oxidation Catalyst

**DOI:** 10.1002/anie.202114932

**Published:** 2021-12-23

**Authors:** Laia Vicens, Giorgio Olivo, Miquel Costas

**Affiliations:** ^1^ Institut de Química Computacional i Catàlisi (IQCC) Departament de Química Universitat de Girona Campus Montilivi 17071 Girona, Catalonia Spain; ^2^ Dipartamento di Chimica Università “La Sapienza” di Roma Piazzale Aldo Moro 5 00185 Rome Italy

**Keywords:** Amino acids, Bioinorganic chemistry, Bioinspired catalysis, H_2_O_2_ activation, Supramolecular chemistry

## Abstract

Precise delivery of a proton plays a key role in O_2_ activation at iron oxygenases, enabling the crucial O−O cleavage step that generates the oxidizing high‐valent metal–oxo species. Such a proton is delivered by acidic residues that may either directly bind the iron center or lie in its second coordination sphere. Herein, a supramolecular strategy for enzyme‐like H_2_O_2_ activation at a biologically inspired manganese catalyst, with a nearly stoichiometric amount (1–1.5 equiv) of a carboxylic acid is disclosed. Key for this strategy is the incorporation of an α,ω‐amino acid in the second coordination sphere of a chiral catalyst via remote ammonium‐crown ether recognition. The properly positioned carboxylic acid function enables effective activation of hydrogen peroxide, leading to catalytic asymmetric epoxidation. Modulation of both amino acid and catalyst structure can tune the efficiency and the enantioselectivity of the reaction, and a study on the oxidative degradation pathway of the system is presented.

## Graphical abstract

Amino acid supramolecular recognition in the 2^nd^ coordination sphere of a bioinspired manganese catalyst allows efficient enzyme‐like activation of H_2_O_2_ by locating the carboxylic acid moiety in proper position to access the 1^st^ coordination sphere of the metal.

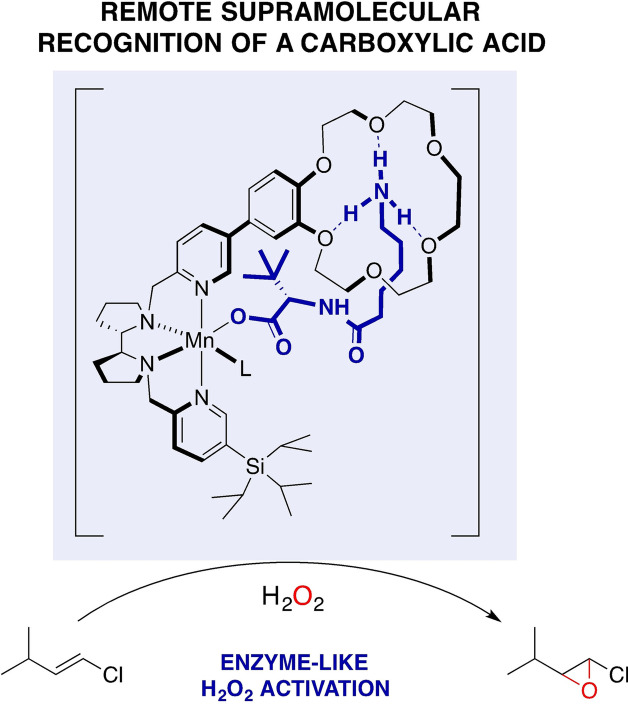

## Introduction

**Figure 1:**
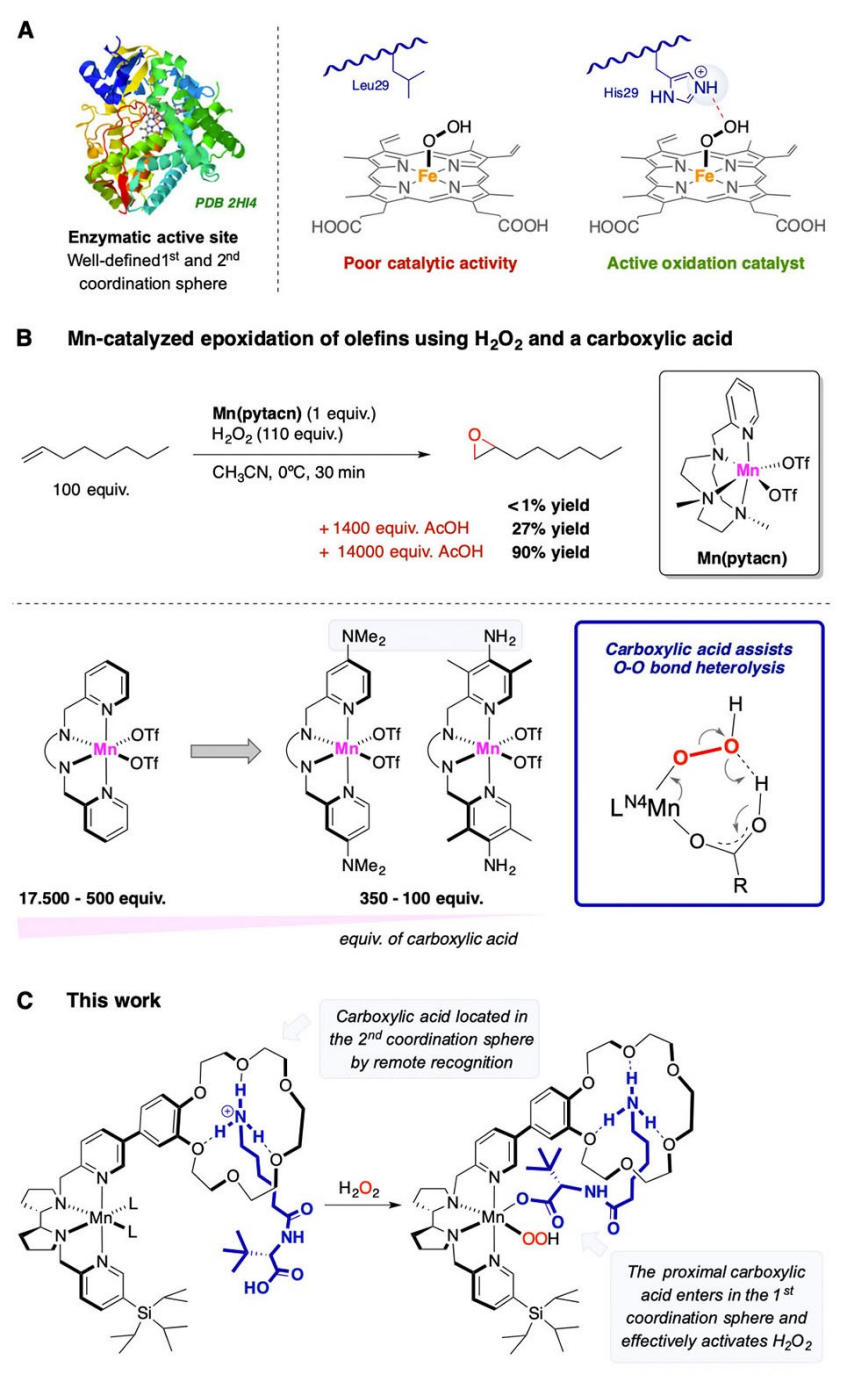
A) Assistance of an acidic residue in the second coordination sphere of oxygenases in O−O cleavage. B) Assistance of external carboxylic acids in H_2_O_2_ activation on bioinspired Mn catalysts, and effect of acid loading. C) Design of this work: inclusion of a carboxylic acid in the second metal coordination sphere to unlock enzyme‐like H_2_O_2_ activation with stoichiometric acid amounts.

The selective oxygenation of organic substrates via activation of O_2_ or peroxides by iron oxygenases has fascinated chemists for decades.^[1]^ In particular, their exceptional activity and selectivity result from a precise control of both the primary and the secondary iron coordination sphere. Such a preorganized environment allows for a fine control over the complex sequence of processes that lead to the high‐valent Fe=O oxidizing species—O_2_ binding to Fe, its reduction, heterolytic O−O bond cleavage—as well as over the oxidation selectivity.^[1]^ Cytochrome P‐450 represents a paradigmatic example; amino acids located in the second coordination sphere play a paramount role: a distal acidic residue assists O−O heterolysis via proton transfer to the Fe^III^−OOH intermediate, aiding the formation of the Fe=O species and a neutral H_2_O molecule.^[2]^ The absence of such acid function—as found in myoglobin, an O_2_ carrier protein—slows down or prevents O−O cleavage. On the other hand, introduction of this acidic residue (site‐directed mutagenesis from Leu29 to protonated His29) converts inert myoglobin into an active oxidation catalyst (Figure 1A).[^2^, ^3^] Optimal pre‐orientation of this residue enables effective O−O cleavage with a single acidic function in a neutral bulk solution.

Conversely, chemists have mainly focused on the first coordination sphere of artificial catalysts,^[4]^ with second coordination sphere effects being scrutinized only recently.^[5]^ Also in bioinspired Fe and Mn catalysts a proton source, usually a carboxylic acid ligand bound in a *cis* coordination site to the peroxide, is required for peroxide activation (Figure 1B).^[6]^ However, while a single acidic function carries out this task in enzymatic systems, a high excess of carboxylic acid is required by artificial catalysts, especially with manganese systems (500–17 500 equiv with respect to the catalyst), resulting in a highly acidic bulk solution, producing a large amount of carboxylic acid waste and limiting the number of carboxylic acids susceptible to be employed as co‐catalysts.[^4f^, ^7^] For instance, Mn(pytacn) is totally inactive in olefin epoxidation without carboxylic acid, slightly active (27 % yield) with 1400 equivalents of acetic acid and reaches its full activity (90 % yield) with 14 000 equivalents (Figure 1B).^[8]^ Effective ways to reduce this acid loading require the use of strong Bronsted acids^[9]^ or strongly electron donating ligands (Figure 1B).^[10]^

Taking inspiration from the machinery operating in enzymatic O−O activation, we reasoned that introduction of a carboxylic acid in proximity to the metal center of a manganese oxidation catalyst may enable enzyme‐like H_2_O_2_ activation with a single acid function. Moreover, since the carboxylic acid structure modulates stereoselectivity in related oxidation catalysts,[^6c^, ^10a^, ^11^] the design of a well‐defined and presumably more rigid Mn complex‐acid adduct may tune enantioselectivity. In this regard, we and others have shown that *N*‐protected amino acids and peptides are optimally structured and tunable platforms for oxidation catalysis.^[12]^ Unfortunately, the covalent linking of such elaborated carboxylic acids to Fe or Mn catalysts is synthetically challenging. Catalysts incorporating carboxylic acid moieties are rare, rather structurally limited and have yielded mixed results so far when applied in peroxide activation reactions, preventing a clear validation of the hypothesis. In fact, these complexes either displayed an activity comparable to the model system devoid of the acid or triggered distinct mechanistic pathways, such as peroxide disproportionation^[13]^ and/or ligand degradation.^[14]^

**Figure 2:**
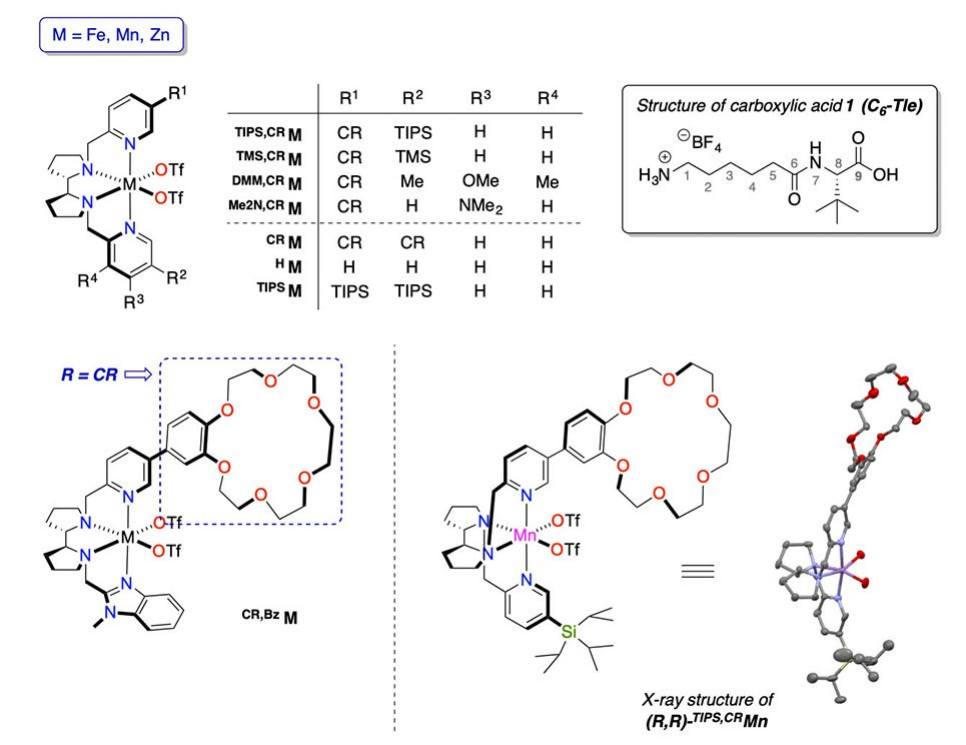
Structure of catalysts used in this work and carboxylic acid **1**. On the bottom right the crystal structure of (*R*,*R*)‐^**CR,TIPS**^ **Mn** (50 % probability ellipsoids) is displayed. Triflate groups, except for O atoms directly bound to the metal, are omitted for clarity. [CR=18‐benzo‐crown‐6‐ether].

Supramolecular recognition may represent a versatile tool to overcome the synthetic challenges of tuning the second coordination sphere of a catalyst,[^5j^, ^15^, ^16^] and test the validity of this approach (Figure 1C). In particular, catalysts ^**CR**^ **M** and ^**CR,X**^ **M** (Figure 2, M=Fe, Mn) bearing two or one 18‐benzo‐crown‐6‐ether (CR) receptors, respectively, strongly bind protonated primary amines, promoting their selective C−H oxidation.^[17]^ We envisaged that these receptors could locate the carboxylic acid head of an opportunely designed α,ω‐protonated amino acid in the second coordination sphere of the metal. The proximity of the acid would then favor its access to the first coordination sphere, deliver a proton in the crucial O−O cleavage step and overall facilitate effective H_2_O_2_ activation with stoichiometric acid amounts. Moreover, the rigid and organized structure of the oxidizing species may offer a chance to fine tune the stereoselectivity of the reaction. Herein, we report the results of this approach and showcase the use of the controlled activation of H_2_O_2_ at a supramolecular Mn catalyst in the asymmetric epoxidation of olefins.

## Results and Discussion

Previously described C_2_ symmetric catalysts containing and devoid of two crown ether receptors (^**CR**^ **M** ^[17a]^ and ^**H**^ **M** ^[18]^ or ^**TIPS**^ **M** ^[11b]^ respectively, M=Fe, Mn, Zn; Figure 2) were chosen for the study. In addition, we synthesized a family of C_1_ symmetric complexes ^**CR,X**^ **M** that combine one crown‐ether containing pyridine (Figure 2) with a second arm bearing different groups that affect the steric and/or the electronic properties (^**CR,TIPS**^ **M**, ^**CR,TMS**^ **M**,^[17c]^ ^**CR,Bz**^ **M,** ^**CR,DMM**^ **M**, ^**CR,Me2N**^ **M**) of the catalyst.^[19]^ Details on the preparation and characterization of the complexes are collected in the Supporting Information. Acid **1** was designed by coupling an L‐α‐amino acid (*tert*‐leucine, Tle) with an ϵ amino acid to include a protonated primary ammonium separated by 9 C−C(N) bonds. This distance is consistent with the C8–C9 remote selectivity previously observed in the remote C−H oxidation of alkyl amines,^[17]^ and was envisioned as the optimal length to ensure ammonium‐crown ether recognition while placing the carboxylic acid at binding distance from the manganese center.

**Figure 3:**
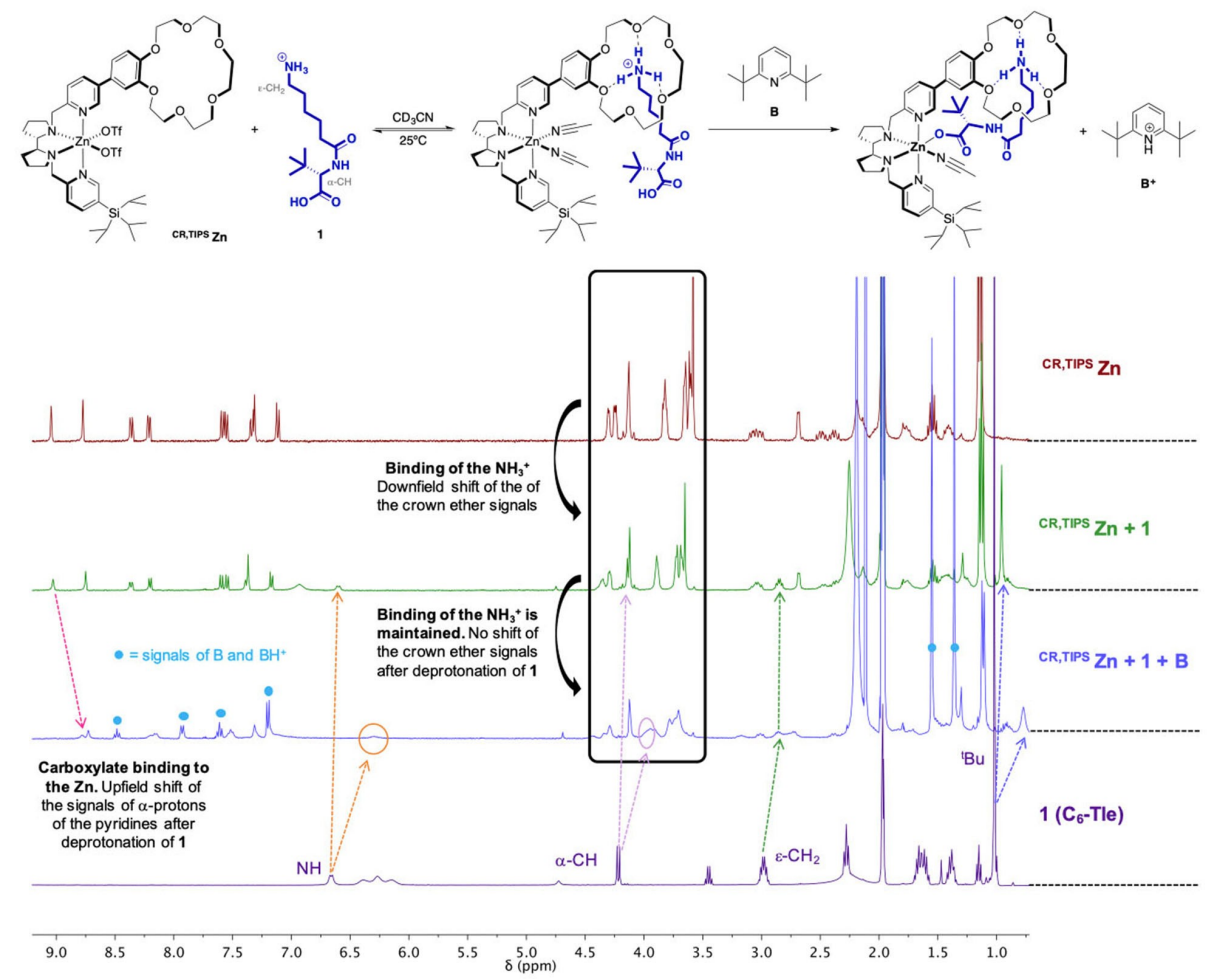
NMR analysis of the binding of **1** to ^**CR,TIPS**^ **Zn** and addition of base **B** (see also Supporting Information, Section 5). ^1^H NMR spectra of ^**CR,TIPS**^ **Zn** (top, red spectrum) before and after addition of one equivalent of **1** (green, second spectrum from top) and base **B** (blue, third spectrum from top), and of pure **1** (bottom spectrum, purple) and the main shifts of signals.

**Figure 4:**
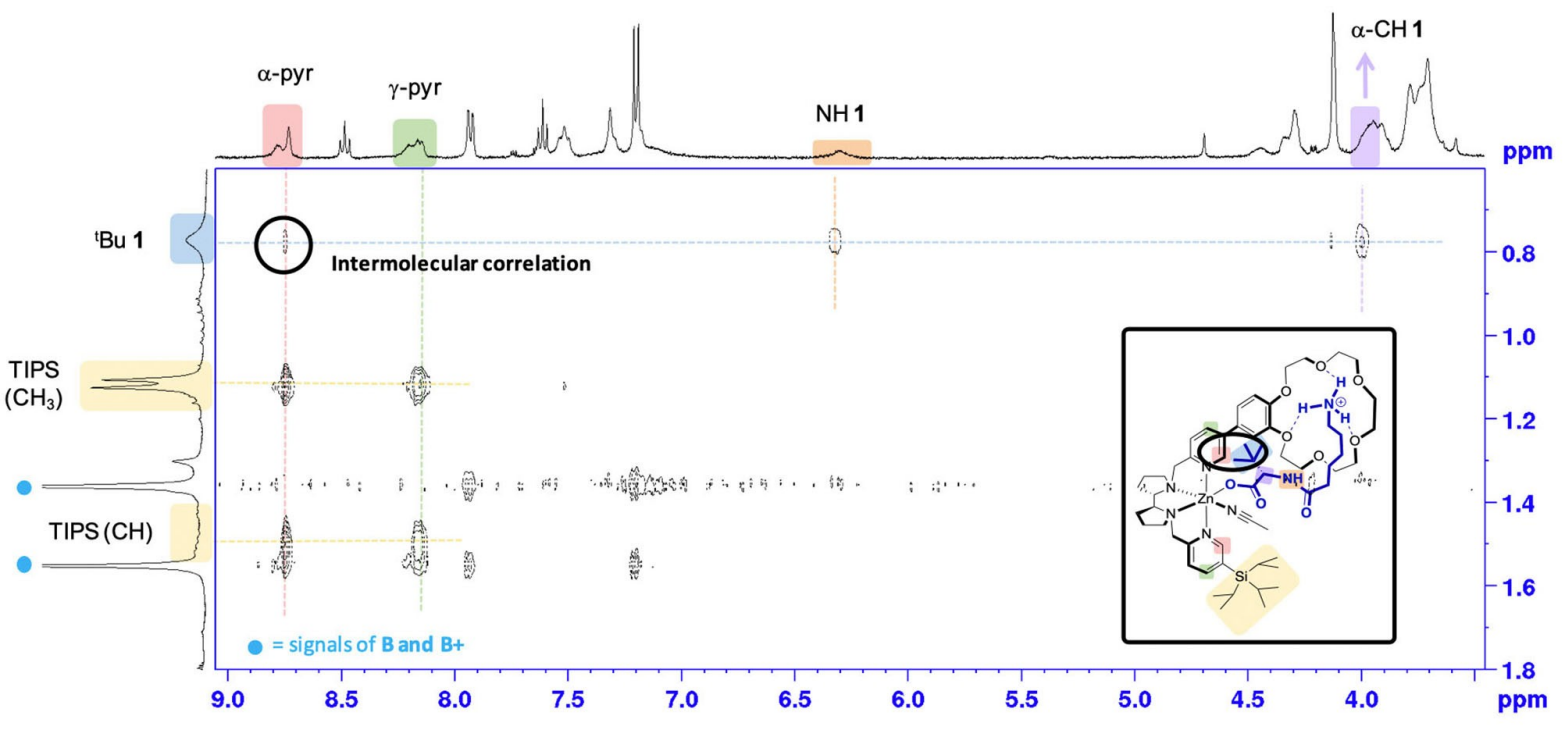
Intermolecular NOESY correlation between deprotonated **1** and ^**CR,TIPS**^ **Zn**, indicating carboxylate coordination (proximity between the ^t^Bu group of **1** and the pyridine α‐C−H signals of the complex.

*Binding of the amino acid*: With these compounds in hand, we investigated the possibility that amino acid **1** binds to ^**CR,TIPS**^ **M** catalyst via NMR analysis (Figure 3). ^**CR,TIPS**^ **Zn** complex was used as it shares the same topology of Fe and Mn complexes but its diamagnetic character simplifies NMR spectra.^[17c]^ As expected, the NH_3_^+^ group of **1** strongly binds to the crown ether receptor at a 2 mM concentration in CD_3_CN (Figure 3), conditions that are comparable to those of catalytic oxidations. The adduct between the catalyst and **1** was also observed by HRMS (Supporting Information, Section 6). The association constant could not be reliably determined but an almost complete association can be estimated with 1 equivalent of **1** (Supporting Information, Figure S7). Under these conditions, the carboxylic acid did not bind to the Zn cation: the only intermolecular NOESY correlation showed proximity between the NH_3_^+^ and the crown signals (Supporting Information, Figure S5). To verify the possibility of this crucial coordination, we treated this mixture with a sterically hindered base (2,6 di‐*tert*‐butylpyridine, **B**) to selectively deprotonate the carboxylic acid and favor its binding while preventing base coordination to the Zn. Upon addition of **B**, all the signals of the Tle portion of **1** (NH, α‐CH and ^t^Bu) shifted upfield by 0.1 ppm, consistent with carboxylic acid deprotonation, while the NH_3_^+^, ϵ‐CH_2_ of **1** and the crown ether ones remained unaltered, indicating that the ammonium‐crown ether binding is unaffected (Figure 3). Most significant, broadening and an upfield shift of pyridine signals was observed, consistent with carboxylate coordination to the metal center of ^**CR,TIPS**^ **Zn**. Detection of an intermolecular NOESY correlation between the ^*t*^Bu of **1** and the α‐CH pyridine of ^**CR,TIPS**^ **Zn** confirmed the targeted carboxylate binding (Figure 4). A control experiment with ^**H**^ **Zn** devoid of the crown ether and **1** showed carboxylic acid deprotonation after addition of **B**, but no sign of strong carboxylate coordination was detected (neither a NOESY correlation nor a shift in the pyridine signals, Supporting Information Figure S15). This experiment highlighted the importance of including **1** in the second coordination sphere to ensure effective carboxylate coordination to the metal.

*Effect of **1** binding on catalytic activity*: Once ascertained that **1** binds to ^**CR,TIPS**^ **M** complexes as depicted in Figure 4, we explored the ability of the resulting supramolecular complex to catalyze olefin epoxidation via effective H_2_O_2_ activation with catalytic amounts of acid (1.5 equiv respect to the metal).^[20]^ 1‐chloro‐3‐methylbut‐2‐ene (**S1**) was chosen as model substrate, with 2 equivalents of H_2_O_2_ in CH_3_CN at 0 °C, and the results are shown in Table 1.

**Table 1:** Influence of acid recognition on **S1** epoxidation.

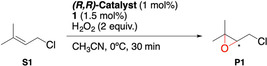				
Entry	Catalyst	Conv. [%]^[a]^	Yield [%]^[a]^	*ee* [%]^[a]^
Influence of acid‐ligand binding and catalyst structure				
1	^CR,TIPS^Mn	55	25	44
2^[b]^	^TIPS,CR^Mn	45	5	9
3^[b,c]^	^TIPS,CR^Mn	48	10	12
**4**	^**CR,TIPS**^ **Mn**	**89**	**63**	**58**
5	^CR,TIPS^Fe	–	–	–
Influence of 10‐fold dilution				
**6^[d]^**	^**CR,TIPS**^ **Mn**	**65**	**40**	**58**
7^[d]^	^TIPS^Mn	–	–	–
Influence of temperature (−40 °C)				
**8^[e]^**	^**CR,TIPS**^ **Mn**	**88**	**65**	**67**
9^[e]^	^TIPS^Mn	–	–	–

Reaction conditions: **S1** (22 μmol, 1 equiv), catalyst (0.22 μmol, 1 mol %), **1** (0.33 μmol, 1.5 mol %), H_2_O_2_ (44 μmol, 2 equiv), CH_3_CN (0.1 M), 0 °C, 30 min (see Supporting Information). [a] Conversions, yields and *ee* determined by GC. The results are expressed as an average of 2–3 runs with an error <5 %. [b] (*S*,*S*) catalyst was used. [c] 3 mol % of **1**. [d] Concentration of **S1** was 0.01 M. [e] *T*=−40 °C

Catalyst and acid **1** loadings were fixed at 1 and 1.5 mol %, respectively,^[20]^ to ensure saturation of the host under reaction conditions (1 mM catalyst concentration). Under these conditions, the non‐supramolecular ^**TIPS**^ **Mn** led to modest epoxide yield (25 %, Table 1, entry 1). ^**CR**^ **Mn** complexes with two crown ether receptors also provide low product yield (up to 10 %), likely because of the saturation of the Mn coordination sphere with two molecules of **1**, each bound to a crown ether receptor (entries 2 and 3), which may hamper peroxide access to the manganese center. Catalyst ^**CR,TIPS**^ **Mn** with a single receptor provided a two‐fold increase of product yield compared to the non‐supramolecular system (63 % yield, entry 4), together with an improvement of enantioselectivity of the epoxide (58 % *ee* entry 4). The analogue ^**CR,TIPS**^ **Fe** was unreactive (entry 5), likely because of catalyst deactivation via amide coordination to Fe as observed before in related catalysts.^[4f]^

At this point, we performed a series of control experiments to verify that the high activity of ^**CR,TIPS**^ **Mn** is the result of the proximity of acid **1** due to remote recognition. In these experiments, the enantioselectivity was used as a probe of the structure of the oxidizing species:^[21]^ as long as the *ee* is unaltered it can be inferred that its structure remains unchanged.

- 10‐fold dilution of the reaction (0.1 mM catalyst concentration, Table 1, entries 6 and 7) to disfavor intermolecular coordination of **1** slightly lowers catalytic activity with ^**CR,TIPS**^ **Mn** (40 % yield), but the enantioselectivity is fully retained (58 % *ee*, entry 6). In an opposite manner, an analogous experiment using ^**TIPS**^ **Mn** results in a complete loss of catalytic activity.
- Performing the reaction at a lower temperature (−40 °C) did not affect the activity of ^**TIPS,CR**^ **Mn** (entry 8) and boosted the enantioselectivity up to 67 % *ee*. Notably, this *ee* is the highest reported for the epoxidation of **S1** in literature. In fact, **S1**, which is an aliphatic olefin and hence a challenging substrate for asymmetric epoxidation.^[22]^ In stark contrast, the analogous experiment with the non‐supramolecular catalyst, ^**TIPS**^ **Mn**, (entry 9) results in a complete loss of catalytic activity.
- Inhibition of **1** binding (Figure 5
  **Figure 5:**
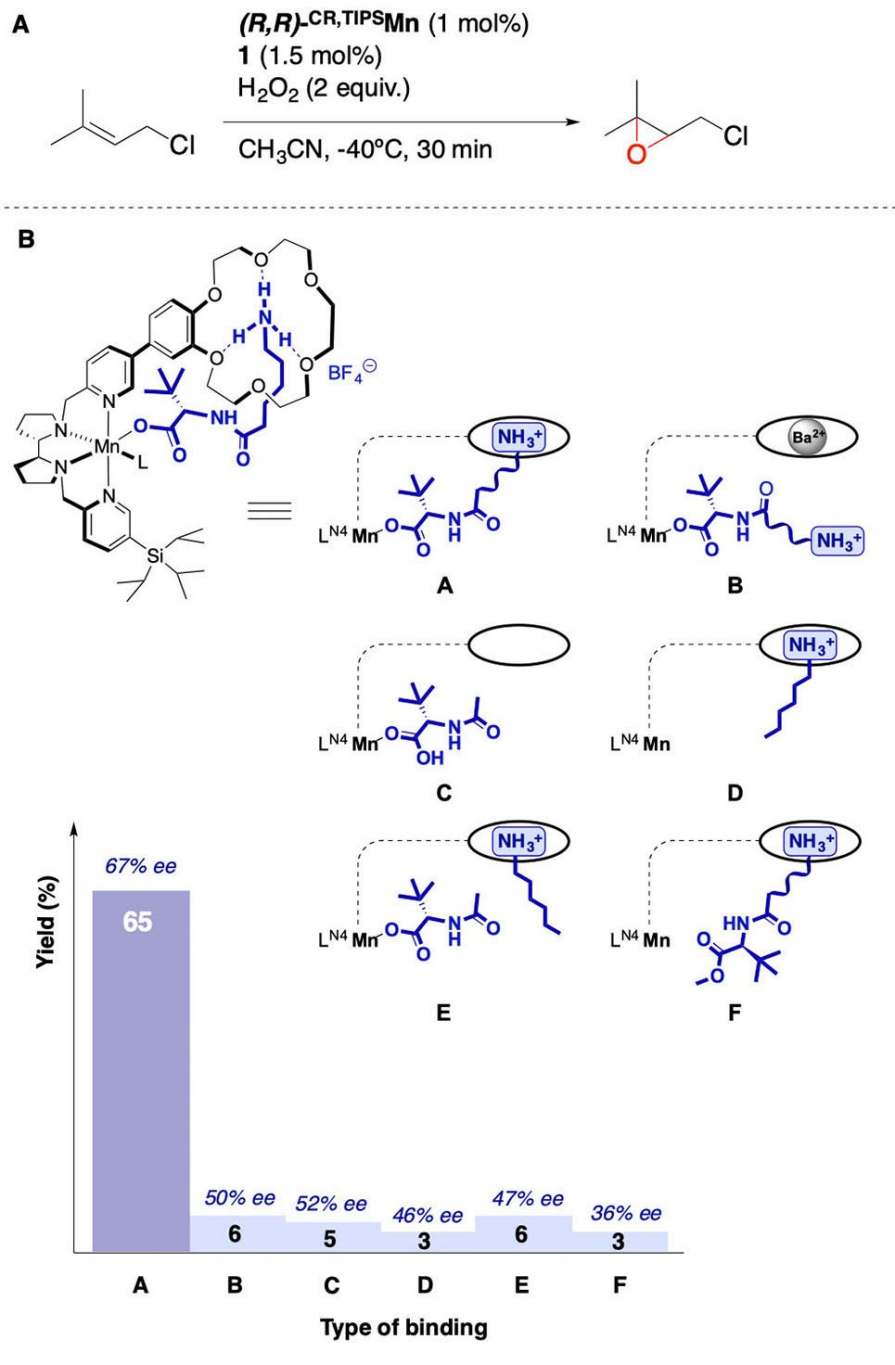
A) Schematic representation of the oxidizing species structure, tuned by modulation of the binding (see Supporting Information, Section 8.3). B) Influence of the modulation of **1** binding over **S1** epoxidation.
  A, structure **B**) via addition of a stronger guest that displaces ammonium ions from the crown ethers (Ba^2+^ cation)^[23]^ reduces the epoxide yield down to 6 % and the *ee* to 50 % (Figure 5B), suggesting ineffective H_2_O_2_ activation and a substantial structural reorganization of the oxidizing species.
- Separation of the components of **1** leads to ineffective catalysis: very low epoxide yields (3–6 %) are obtained either when the acid or the ammonium sections are used independently (structures **C** and **D**, respectively) or are added together but lack a covalent connection (structure **E**). In all these cases, the *ee* is lower than that obtained with **1**.
- Use of an ester derivative of **1** devoid of the carboxylic acid proton shuts down catalytic activity (structure **F**), highlighting the importance of the acid function in the activation of the H_2_O_2_.

Taken together, all these control experiments indicate that incorporation of stoichiometric amounts of **1** in the second coordination sphere of the metal center via supramolecular recognition is key for its facile binding to the metal and a high catalytic activity, consistently with the proposed design. Moreover, binding of **1** to the crown ether improves the enantioselectivity of the reactions, probably reflecting an improved spatial organization of the oxidizing species.

Finally, competition of **1** and acetic acid for the Mn center was explored (Table 2). Enantioselectivity was again used as a probe of the nature of the oxidizing species structure: 67 % *ee* was obtained for **1/**(*R,R*)*‐* ^**CR,TIPS**^ **Mn** (entry 1), and a more modest 44 % ee was observed for AcOH/(*R,R*)*‐* ^**CR,TIPS**^ **Mn** (entry 2). This difference is explained considering that the carboxylic acid is a ligand of the Mn^V^(O)(carboxylate) oxidizing species, and therefore influences the stereoselectivity of the reaction.[^6c^, ^24^] When 1.5 mol % of both **1** and AcOH (1 : 1 mixture) were used (entry 3) the epoxide was obtained with 67 % *ee*, indicating that **1** outcompetes acetic acid binding to the oxidizing species. An analogous behavior was also observed when other aliphatic carboxylic acids were used instead of acetic acid (see Supporting Information, Section 8.5). Also, when higher amounts of acetic acid were added in the reaction mixture together with 1.5 mol % of **1** (entries 4–6), the 67 % *ee* was retained. These results suggest that **1** dominates the O−O activation step and binds to the oxidizing species in all cases, even in the presence of much higher amounts of external acid (over 300‐fold), which can be only explained considering a high effective concentration and optimal orientation of its carboxylic function towards the metal center resulting from supramolecular recognition.

**Table 2:** Epoxidation of **S1** using mixtures of **1** and AcOH as external carboxylic acid.

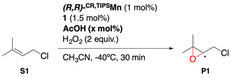						
Entry	**1**	AcOH	**1** : AcOH	Conv. [%]^[a]^	Yield [%]^[a]^	*ee* [%]^[a]^
1	1.5 mol %	–	–	88	65	**67**
2^[b]^	–	1.5 mol %	–	25	10	**44**
3	1.5 mol %	1.5 mol %	1 : 1	99	77	**67**
4	1.5 mol %	0.1 equiv	1 : 7	98	78	**67**
5	1.5 mol %	1 equiv	1 : 67	98	79	**67**
6	1.5 mol %	5 equiv	1 : 333	93	78	**67**

Reaction conditions: **S1** (22 μmol, 1 equiv), **(*R***, ***R*** **)‐^CR,TIPS^Mn** (0.22 μmol, 1 mol %), **1** (0.33 μmol, 1.5 mol %), AcOH, H_2_O_2_ (44 μmol, 2 equiv), CH_3_CN (0.1 M), −40 °C, 30 min (see Supporting Information). [a] Conversions, yields and *ee* determined by GC. The results are expressed as an average of 2–3 runs with an error <5 %. ^[b]^ Peroxide titration at the end of the reaction indicated 90 % of H_2_O_2_ consumption (see Supporting Information, Section 8.7).

In addition, this set of experiments further confirmed that supramolecular binding of **1** is crucial to activate H_2_O_2_ in a productive manner and obtain high product yields. In fact, the reaction performed with acetic acid (entry 2) affords only 10 % yield of epoxide, but the amount of H_2_O_2_ consumed is 90 % according to peroxide titration at the end of the reaction (see supporting information, Section 8.7).^[25]^ These results indicate that under these conditions the catalyst reacts with H_2_O_2_ mainly in a non‐productive pathway, presumably peroxide disproportionation.

*Tune of the catalyst structure*: With an effective catalytic system in hands, we set out to further tune the structure of the oxidizing species via manipulation of catalyst and amino acid structure.

At first, modification of the Mn complex was explored, varying the ligand chirality or the arm devoid of the crown ether receptor (Table 3, catalysts depicted in Figure 2). Inversion of catalyst chirality reveals a noticeable match/mismatch effect, with the (*S,S*)‐^**CR,TIPS**^ **Mn**/**1** combination providing lower yields and enantioselectivities (entry 2). Manipulation of the other pyridine had a lower influence: neither the replacement of triisopropylsilyl (TIPS) group with a less‐hindered trimethylsilyl one (TMS, ^**CR,TMS**^ **Mn**, entry 3) nor with an electron‐rich pyridine (^**CR,DMM**^ **Mn**, entry 4) provided significant changes. As expected,[^10b^, ^10c^, ^10d^, ^10e^, ^10f^] the use of a stronger electron‐donating substituent (^**CR,Me2N**^ **Mn**, entry 5) increased the *ee*, although at the expense of the yield. Replacement of the pyridine with a benzimidazole ring (^**Bz,CR**^ **Mn**, entry 6) provided modest results. Overall, match/mismatch effects seem to play a significant role, consistent with the formation of a rigid and well‐defined cavity around the metal, while substitution on the crown‐free pyridine affects this structure only to a limited extent.

**Table 3:** Influence of catalyst structure on **S1** epoxidation.

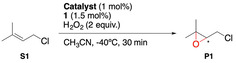				
Entry	Catalyst	Conv. [%]^[a]^	Yield [%]^[a]^	*ee* [%]^[a]^
1	**(*R,R*)*‐*** ^**CR,TIPS**^ **Mn**	88	65	67
2	**(*S,S*)*‐*** ^**CR,TIPS**^ **Mn**	92	61	52
3	**(*S,S*)*‐*** ^**CR,TMS**^ **Mn**	90	58	55
4	**(*S,S*)*‐*** ^**CR,DMM**^ **Mn**	95	63	52
5	**(*S,S*)*‐*** ^**CR,Me2N**^ **Mn**	74	43	72
6	**(*S,S*)*‐*** ^**CR,Bz**^ **Mn**	42	15	44

Reaction conditions: **S1** (22 μmol, 1 equiv), **catalysts** (0.22 μmol, 1 mol %), **1** (0.33 μmol, 1.5 mol %), H_2_O_2_ (44 μmol, 2 equiv), CH_3_CN (0.1 M), −40 °C, 30 min (see Supporting Information). [a] Conversions, yields and *ee* determined by GC. The results are expressed as an average of 2–3 runs with an error <5 %.

**Figure 6:**
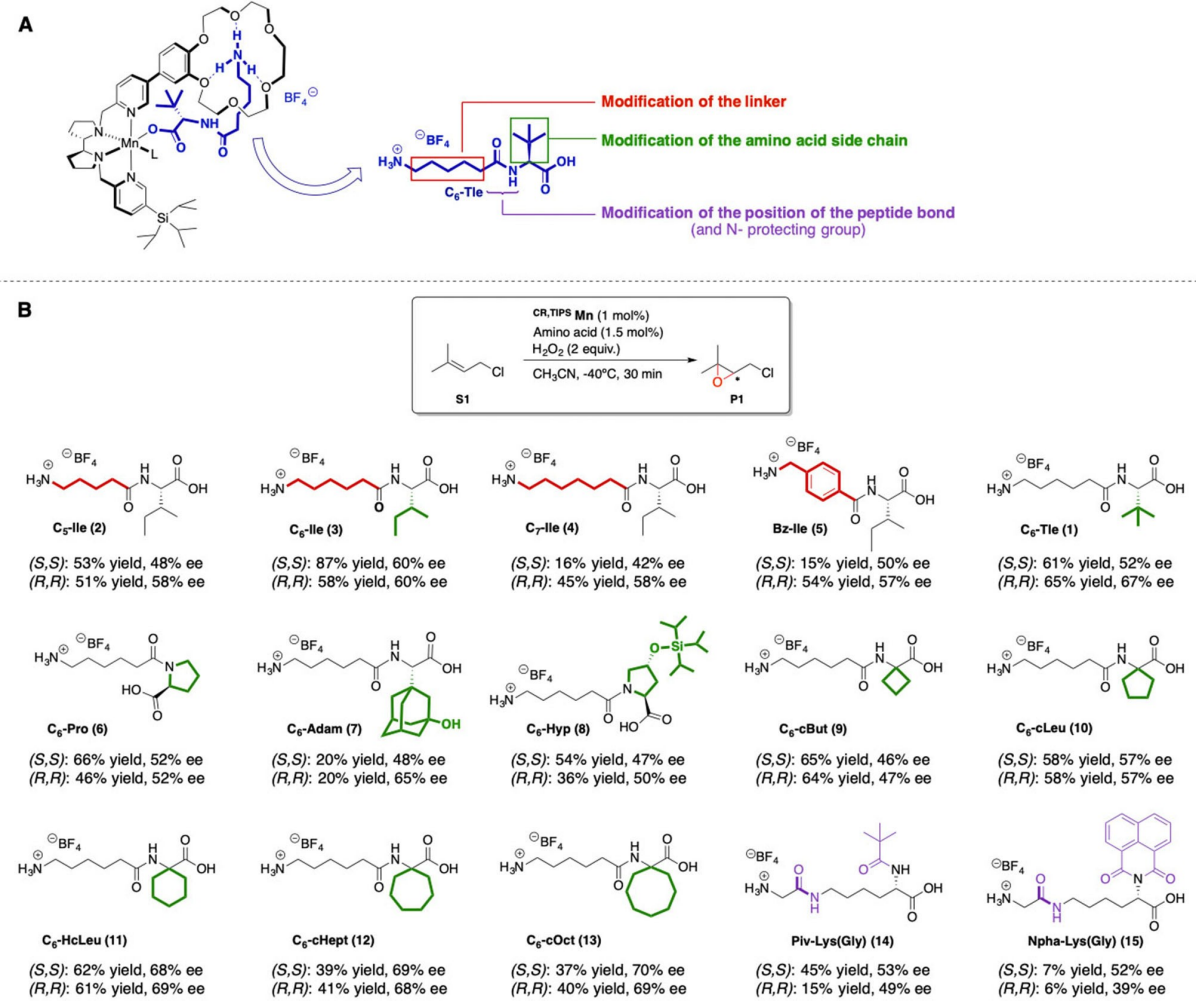
Tuning of the amino acid structure. A) Schematic representation of amino acid modifications. B) Effect of the modifications on **S1** epoxidation. Further results with other olefins are reported in the Supporting Information (Section 9.2).

Then, we focused our attention on the amino acid structure (Figure 6A), modulating the distance between the acid and the ammonium group, the lateral chain of the α‐amino acid and the position of the amidic bond. To evaluate match‐mismatch effects both catalyst enantiomers were tested with each of the modified amino acids.

The optimal acid‐ammonium distance is provided by a 6‐carbon linker (**3**, C_6_‐Ile, Figure 6B, **2**–**4**): both a decrease (**2**, C_5_‐Ile) and an increase (**4**, C_7_‐Ile) of the aliphatic chain length eroded yields and *ee*’s. Also, increase of linker rigidity by incorporation of an aromatic ring led to lower activity (Figure 6B, **5**).

Modification of the α‐amino acid side chain allows for a more effective modulation of the environment close to the metal, and hence of enantioselectivity. Replacement of the bulky *tert‐*butyl group (**1**, C_6_‐Tle) with a chiral isobutyl one (**3**, C_6_‐Ile) slightly eroded the yield and enantiomeric excess for the (*R,R*) catalyst, but improved both yield and *ee* for the (*S,S*) catalyst, evidencing once again the importance of match/mismatch effects between the catalyst and the amino acid chain. Use of a rigid and structurally different amino acid like proline (**7**, C_6_‐Pro) led to a decrease in both yield and enantioselectivity, suggesting that α‐amino acid lateral chains may have a significant effect in defining the yield and enantioselectivity. Along this line, we introduced bulky groups in the lateral chains to increase the steric hindrance of the amino acid‐ from tert‐butyl (**1**) to an adamantyl (**7**, C_6_‐Adam) and from proline (**6**) to a TIPS‐protected hydroxyproline (**8**, C_6_‐Hyp). These bulkier chains did not result in further improvements, obtaining lower epoxide yields and comparable enantioselectivities to the previous ones, suggesting that the hindrance of these groups does not affect substrate approach trajectories. Comparable results to (*R,R*)‐^**CR,TIPS**^ **Mn** and C_6_‐Tle (**1**) were only obtained with homocycloleucine (**11**, C_6_‐HcLeu), a non‐natural achiral α,α‐disubstituted α‐amino acid. As expected, given its lack of chirality, the results obtained with the two enantiomers of the catalyst coincide within experimental error (±1 %). When the cyclohexyl moiety was replaced by a cyclobutyl (**9**, C_6_‐cBut) or a cyclopentyl (**10**, C_6_‐cLeu), comparable yields were obtained, but the lower the size of the ring the lower was the enantioselectivity. When larger rings were used (**12**, C_6_‐cHept and **13**, C_6_‐cOct), an almost imperceptible improvement of the enantioselectivity was observed, accompanied by a substantial decrease of the yields.

Finally, we explored a different amino acid design, where an *N*_α_‐protected lysine derivative is connected to glycine via the distal *N*_ϵ_‐group. This way, the peptide bond is shifted in a more remote position from the carboxylic acid (Figure 6B, **14** and **15**). Curiously, in these cases the (*S,S*)‐^**CR,TIPS**^ **Mn** forms the optimal combination with the amino acid, suggesting a slightly different arrangement. However, modest yields and *ee*’s were achieved with both *N*‐pivaloyl (**14**) and *N*‐naphthalenimide (**15**) lysine derivatives.

Overall, almost all the screened amino acid furnished epoxide **P1** in good to high yield, demonstrating the generality of our recognition strategy. Two combinations were identified as the best catalytic systems for enantioselectivity: (*R,R*)‐^**CR,TIPS**^ **Mn/1** (65 % yield, 67 % *ee*) and ^**CR,TIPS**^ **Mn/11** (61 % yield, 69 % *ee*).

The best combinations previously identified were also applied in the epoxidation of other aliphatic olefins with good to excellent yields but unfortunately low to modest enantioselectivities were obtained, with the exception of a chromene derivative, in which up to 93 % *ee* was achieved (see Supporting Information, Section 11).

**Scheme 1:**
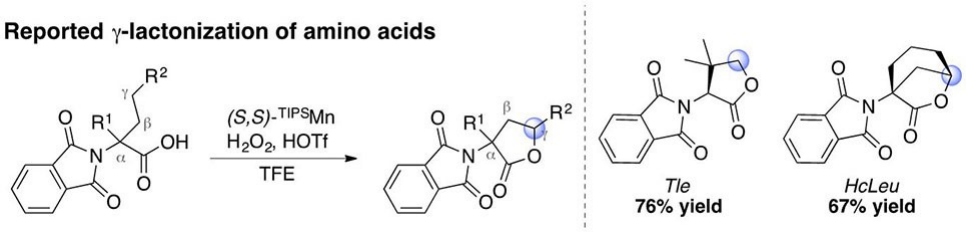
Reported γ‐lactonization of amino acids by (*S*,*S*)‐^**TIPS**^ **Mn**.^[26]^

*Immolative self‐oxidation of the amino acid chain and correlation with catalytic activity*: Analysis of the structure of compounds **1** and **11** suggests that the amino acid residue at the C‐terminus of the peptide chain may be susceptible to self‐oxidation via carboxylic acid directed intramolecular γ−C−H lactonization (Scheme 1).^[26]^ We envisioned that such process would consume the bound carboxylic acid function, preventing productive activation of H_2_O_2_ and therefore blocking the epoxidation reaction.

**Figure 7:**
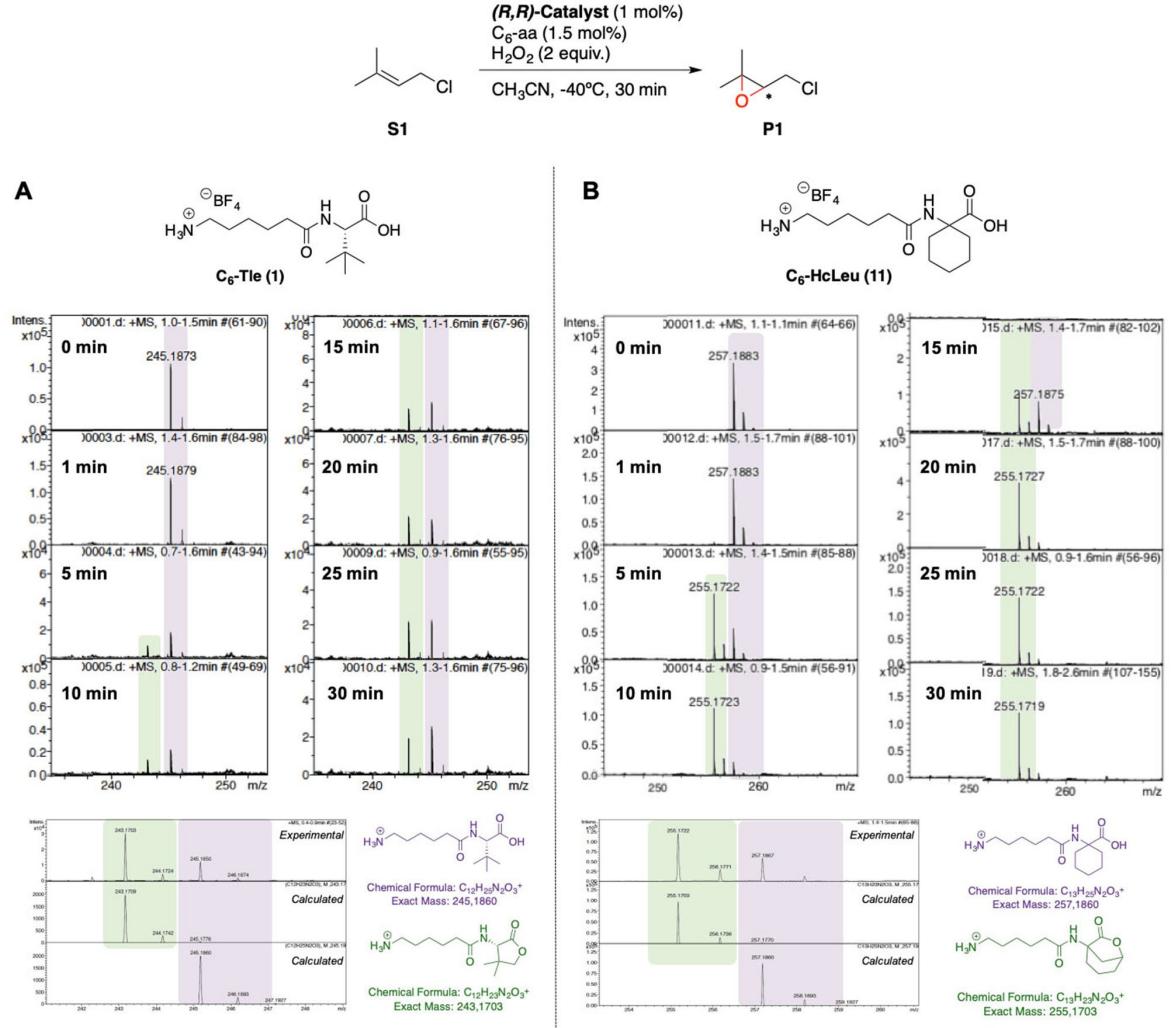
HRMS time‐course analysis of the epoxidation of **S1** using (*R*,*R*)‐^**CR,TIPS**^ **Mn**/**1** (A) and (*R*,*R*)‐^**CR,TIPS**^ **Mn**/**11** (B).

To investigate this hypothesis, we performed a time‐course GC and HRMS analysis of the reaction using both amino acid chains C_6_‐Tle (**1**) and C_6_‐HcLeu (**11**) (Figure 7). At the beginning of the reaction, HRMS spectra of two standard catalytic oxidations showed a main peak corresponding to the amino acid (**1**, *m*/*z*=245 and **11**, *m*/*z*=257) (Figure 7, purple). After 5 minutes, a second peak with M‐2 (*m*/*z*=243 and 255, respectively) appeared, consistent with the expected lactone product (Figure 7, green). Over time, the relative intensity of the amino acid peak decreased in favor of the lactone one, reflecting intramolecular amino acid oxidation. The progression and extent of this self‐oxidation depends on the nature of the amino acid. While **11** was fully consumed after 20 minutes, the peak of **1** remained of comparable intensity to that of the lactone even at the end of the reaction. Such different behavior between the two chains can be ascribed to the different reactivity of the γ−C−H bonds in the two amino acids. Lactonization of **1** involves challenging oxidation of a strong primary C−H bond, while a weaker methylenic site is oxidized in **11**.

**Figure 8:**
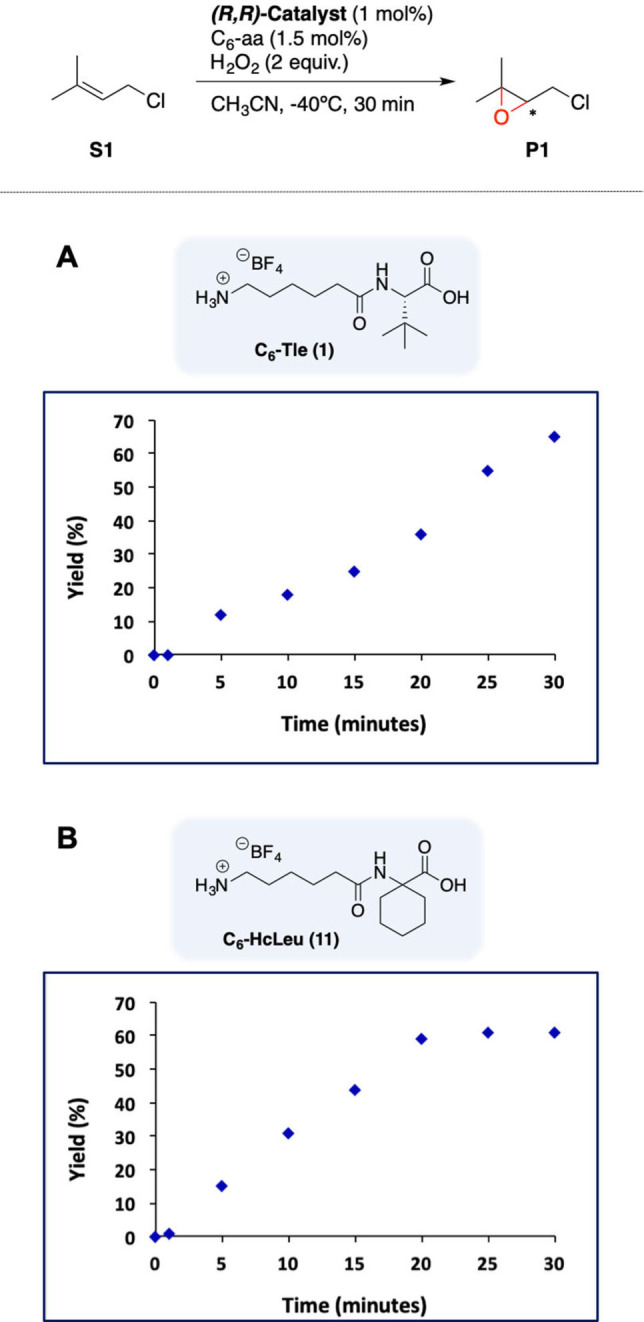
Time profile of **S1** epoxidation using (*R*,*R*)‐^**CR,TIPS**^Mn/**1** (A) (*R*,*R*)‐^**CR,TIPS**^Mn/**11** (B).

Consistently, we observed a correlation between the progression of amino acid self‐oxidation and catalytic epoxidation (Figure 8).

In the case of (*R,R*)‐^**CR,TIPS**^ **Mn/1**, epoxidation continued during all the peroxide addition time (30 minutes), in line with the observation of **1** by HRMS even at the end of the reaction. Conversely, the epoxidation activity of ^**CR,TIPS**^ **Mn/11** stopped abruptly at 20 minutes, when HRMS analysis indicated that **11** has been fully converted to lactone. Therefore, intramolecular lactonization of the amino acid competes with intermolecular olefin oxidation, leading to catalyst deactivation once the acid is fully consumed. Of notice, in the case of (*R,R*)‐^**CR,TIPS**^ **Mn/1**, the acid is not fully consumed so catalytic activity remains during all the addition of peroxide. This observation is notable because amino acid oxidation entails cleavage of strong aliphatic C−H bonds (≈100 kcal mol^−1^ in the case of primary C−H bonds), which becomes competitive with the a priori much easier epoxidation of an olefin, present in large excess. It is likely that such degradation of the carboxylic acid co‐catalyst may be relevant also in other related Fe‐ and Mn‐catalyzed oxidations.[^4a^, ^4e^]

## Conclusion

In conclusion, we describe herein an effective, enzyme‐like hydrogen peroxide activation at a Mn catalyst with amounts of acid that are almost stoichiometric to the catalyst. Remote, supramolecular crown ether‐ammonium recognition of an α,ω‐amino acid places a carboxylic acid function in the second coordination sphere of the manganese, ready to access its first coordination sphere and enter into the catalytic cycle, enabling productive H_2_O_2_ activation for asymmetric epoxidation reactions. Such a supramolecular approach furnishes a versatile strategy to insert different, tunable amino acid structures in the catalytic system without the need for elaborated and often synthetically challenging covalent modifications. In fact, use of different amino acids allows facile tuning of the stereoselectivity of the reaction.

NMR analysis and catalytic oxidation experiments highlighted that remote binding of the amino acid to the ligand is key for effective epoxidation, while its absence results in sluggish activity. These results thus can be regarded as a proof‐of‐concept for the use of supramolecular chemistry to readily tune the second coordination sphere and design enzyme‐like artificial catalysts. On the other hand, a time‐course study of the reaction unveils some of the limits of this approach, revealing that self‐oxidation of the bound amino acid to form the corresponding lactone competes with intermolecular epoxidation reaction and in some cases, gradually leads to catalyst deactivation. Despite having a detrimental significance in the context of catalyst stability, this reactivity hints at future exciting possibilities for these catalysts, using supramolecular recognition to promote selective oxidation of notoriously strong aliphatic C−H bonds in the presence of a priori oxidation sensitive functionalities.

## Conflict of interest

The authors declare no conflict of interest.

1

## Supporting information

As a service to our authors and readers, this journal provides supporting information supplied by the authors. Such materials are peer reviewed and may be re‐organized for online delivery, but are not copy‐edited or typeset. Technical support issues arising from supporting information (other than missing files) should be addressed to the authors.

Supporting Information

## Data Availability

The data that support the findings of this study are available in the Supporting Information of this article.
