# Supplementary material for: Remote Amino Acid Recognition Enables Effective Hydrogen Peroxide Activation at a Manganese Oxidation Catalyst
Source: Angew Chem Int Ed Engl. 2021 Dec 23;61(7):e202114932. doi: 10.1002/anie.202114932 (PMC9304166; doi:10.1002/anie.202114932)
Supplement: Supplementary file 1 — Supporting Information [file ANIE-61-0-s001.pdf]

Angewandte Chemie
Eine Zeitschrift der Gesellschaft Deutscher Chemiker

Supporting Information

Remote Amino Acid Recognition Enables Effective Hydrogen Peroxide Activation at a Manganese Oxidation Catalyst

L. Vicens, G. Olivo, M. Costas**

Table of Contents

1. Materials and Methods 4
1.1. Material 4
1.2. Instrumentation 4

2. Synthesis of the catalysts 4
2.1. Synthesis of ligands 5
2.2. Synthesis of the complexes 7

3. General procedures for the synthesis of the α,ω-amino acids 10
3.1. General Procedure A: Esterification of the amino acids 10
3.2. General Procedure B: Boc-protection of the linkers 11
3.3. General Procedure C: Synthesis of succinimidyl esters 11
3.4. General Procedure D: Coupling of the linker and the amino ester 12
3.5. General Procedure E: Hydrolysis of the ester 12
3.6. General Procedure F: Coupling of the succinimidyl esters and the amino acids 13
3.7. General Procedure G: Deprotection of the Boc and protonation of the amine 13

4. Synthesis and characterization of the α,ω-amino acids 14
4.1. C_6-Tle (1) 14
4.2. C_5-Ile (2) 16
4.3. C_6-Ile (3) 18
4.4. C_7-Ile (4) 19
4.5. Bz-Ile (5) 22
4.6. C_6-Pro (6) 24
4.7. C_6-Adam (7) 26
4.8. C_6-Hyp (8) 28
4.9. C_6-cBut (9) 30
4.10. C_6-cLeu (10) 31
4.11. C_6-HcLeu (11) 32
4.12. C_6-cHept (12) 34
4.13. C_6-cOct (13) 35
4.14. Piv-Lys(Gly) (14) 37
4.15. Npha-Lys(Gly) 39

5. NMR analysis of the binding 42
5.1. Binding of 1 to *(S,S)*-^{CR,TIPS}Zn 42
5.2. Titration of *(S,S)*-^{CR,TIPS}Zn with 1 45
5.3. Addition of a base to favor carboxylate coordination to Zn 46
5.4. Control experiment: Binding of 1 to *(S,S)*-^{H}Zn 50

6. HRMS analysis of the binding 52

7. Reaction conditions for the epoxidation of olefins 54

8. Control experiments **55**
8.1. Selected experiments using 1 equiv. of amino acid 55
8.2. Synthesis of the co-ligands 56
8.3. Reaction conditions for each type of binding (Figure 4C) 57
8.4. Selected control experiments in the epoxidation of styrene 58
8.5. Competitive reactions using an external carboxylic acid and 1 (C_6-Tle) 59
8.6. Competitive reactions using an external carboxylic acid and 11 (C_6-HcLeu) 60
8.7. Determination of the consumed H_2O_2 in the reaction using AcOH 61

9. Optimization of the catalytic system **61**
9.1. Optimization of catalyst structure 61
9.2. Optimization of the amino acid structure 63

10. Time-course analysis of the epoxidation of S1 .... **65**
10.1. Time-course analysis using *(R,R)*-^{CR,TIPS}Mn and C_6-Tle (1) 65
10.2. Time-course analysis using *(R,R)*-^{CR,TIPS}Mn and C_6-HcLeu (11) 70

11. Substrate scope **75**
11.1. Synthesis and characterization of racemic epoxides 75
11.2. Epoxidation of geranyl acetate 78

12. ^{1}H and ^{13}C{H} NMR spectra of the ligands and complexes **82**

13. ^{1}H and ^{13}C{H} NMR spectra of the α,ω-amino acids **87**

14. ^{1}H and ^{13}C{H} NMR spectra of epoxides **130**

15. GC traces of epoxides **132**

16. References **145**

1. Materials and Methods

1.1. Material

Reagents and solvents used were of commercially available reagent quality unless stated otherwise. Solvents were purchased from SDS, Aldrich, Scharlab and Fluorochem. Sigma-Aldrich HPLC-grade acetonitrile was employed for oxidation reactions. Liquid substrates were filtrated by a small plug of basic alumina prior its use.

1.2. Instrumentation

NMR spectra were taken on a Bruker Ultrashield DPX300 or on a Bruker Ultrashield ASCEND Nanobay spectrometer using standard conditions. Spectra were referenced to the residual proton solvents peaks or TMS (tetramethylsilane) for ^{1}H. High resolution mass spectra (HRMS) were recorded on a Bruker MicroTOF-QII instrument with an ESI source and a quadrupole analyzer at Serveis Tècnics de Recerca of the University of Girona. Samples were introduced into the mass spectrometer ion source by direct infusion through a syringe pump and were externally calibrated using sodium formate. X-Ray measurements were carried out on a BRUKER D8 QUEST ECO diffractometer using graphite-monochromated Mo Kα radiation (λ = 0.71 Å) from an X-ray tube. IR spectra were taken in a Bruker Alpha FT-IR spectrometer using a MKII Golden Gate single reflection ATR system. Elemental analyses were performed using a CHNS-O EA2400 serie II elemental analysis from Perkin Elmer. GC-MS spectral analyses were performed on an Agilent 7890A gas chromatograph (HP-5MS column, 30 m x 0.25 mm, 0.25 μm, Agilent J&W) interfaced with an Agilent 5975X mass spectrometer. NH_3 was used as the ionization gas. Oxidation products were identified by comparison of their GC retention times with those of authentic compounds. NMR Chromatographic resolution of enantiomers was performed on an GC-7820-A chromatograph from Agilent Technologies using a Cyclosil-B column (30 m x 0.25 mm, 0.25 μm, Agilent J&W).

2. Synthesis of the catalysts

The complexes $[Mn(OTf)_2(^{TIPS}pdp)]$,[1] $[Mn(OTf)_2(^{CR}pdp)]$[2] and $[Mn(OTf)_2(^{CR,TMS}pdp)]$[3] were prepared according to the reported procedures. The complexes $[Mn(OTf)_2(^{CR,TIPS}pdp)]$, $[Mn(OTf)_2(^{CR,DMM}pdp)]$, $[Mn(OTf)_2(^{CR,Me2N}pdp)]$ and $[Mn(OTf)_2(^{CR,Bz}pdp)]$ were prepared according the procedures described in this section.

2.1. Synthesis of ligands

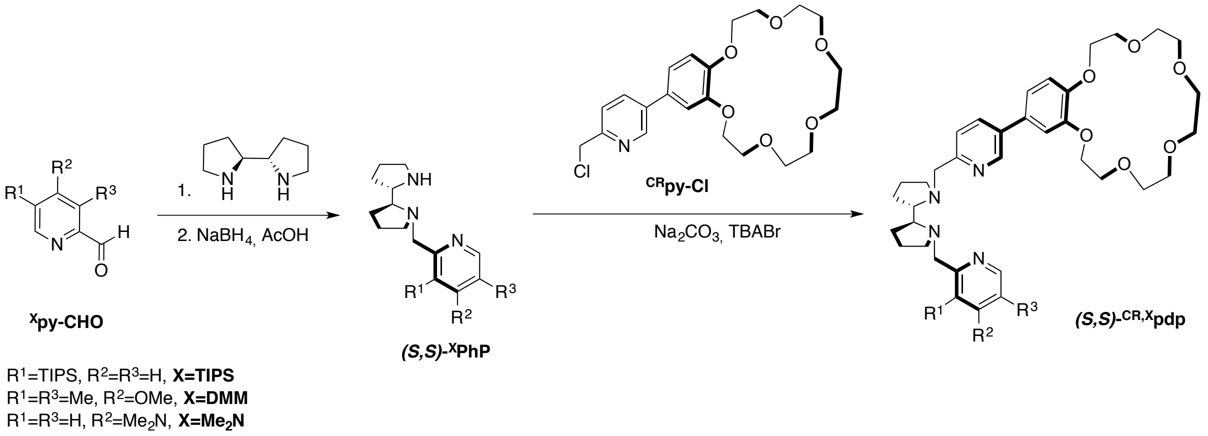

^{TIPS}py-CHO,[4] ^{DMM}py-CHO, ^{Me2N}py-CHO,[5] *(S,S)* ^{DMM}PhP,[6] *(S,S)*-^{Me2N}PhP,[7] *(S,S)*-^{TIPS}PhP,[7] *(R,R)*-^{TIPS}PhP[7] and ^{CR}py-Cl[2] were prepared according to reported procedures. *(S,S)*- or *(R,R)*-^{CR,X}pdp were synthesized from the corresponding *(S,S)*- or *(R,R)*-^{X}PhP following an adapted version of a reported procedure[8] as described below.

An 8 mL vial was charged with ^{CR}py-Cl (1.05 equiv.), the desired monoalkylated bipyrrolidine *(S,S)*- or *(R,R)*-^{X}PhP (1 equiv.) and NaOH (15 equiv.), and then 3 mL of CH_2Cl_2 and 2 mL of distilled H_2O were added. The organic solution turned red after complete dissolution of the reactants, to gradually evolve towards a brownish color. The biphasic mixture was vigorously stirred overnight. After this time, the mixture was diluted with 1M aqueous NaOH (5 mL) and then extracted with CH_2Cl_2 (4x10 mL). The combined organic phases were dried over anhydrous $MgSO_4$, filtered and the solvent was removed under reduced pressure, to yield a brown oil. The crude was then purified by flash chromatography over silica gel (CH_2Cl_2:MeOH:NH_3, 100:8:4) followed by washing with NaOH 1M to afford the pure ligand.

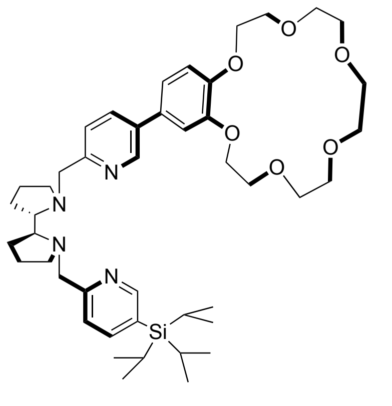

***(R,R)*-^{CR,TIPS}pdp**. Prepared from ^{CR}py-Cl (106.0 mg, 0.244 mmol) and *(R,R)*-^{TIPS}PhP (90.0 mg, 0.232 mmol) following the general procedure. After purification, the product was obtained as a clear oil (106.0 mg, 0.135 mmol, 58% yield). ^{1}H-NMR (400 MHz, $CDCl_3$) δ, ppm: 8.67 (dd, *J* = 2.4, 0.8 Hz, 2H), 8.56 (dd, *J* = 1.8, 0.8 Hz, 2H), 7.71 (ddd, *J* = 11.2, 7.9, 2.1 Hz, 2H), 7.41 (t, *J* = 7.6 Hz, 2H), 7.15 – 7.04 (m, 2H), 6.95 (d, *J* = 8.2 Hz, 1H), 4.29 – 4.15 (m, 6H), 3.95 (dd, *J* = 5.7, 3.5 Hz, 4H), 3.83 – 3.71 (m, 4H), 3.77 – 3.67 (m, 4H), 3.69 (s, 4H), 3.51 (dd, *J* = 14.2, 4.5 Hz, 2H), 3.04 (tt, *J* = 9.3, 4.3 Hz, 2H), 2.91 – 2.73 (m, 2H), 2.33 – 2.16 (m, 2H), 1.92 – 1.75 (m, 2H), 1.79 – 1.61 (m, 4H), 1.38 (dt, *J* = 14.7, 7.5 Hz, 3H), 1.05 (d, *J* = 7.5 Hz, 18H). ^{13}C-NMR (100 MHz, $CDCl_3$) δ, ppm: 160.3, 158.7, 154.6, 149.0, 146.9, 143.4, 134.4, 131.2, 127.2,

122.5, 122.0, 120.0, 114.3, 113.1, 70.7, 69.6, 69.3, 69.1, 65.4, 61.3, 60.8, 55.5, 55.3, 25.9, 23.6, 18.4, 10.6. HRMS (ESI-MS) *m/z* calculated for $C_{45}H_{68}N_4O_6Si$ $[M+H+Na]^{2+}$ 406.2437, found 406.2450.

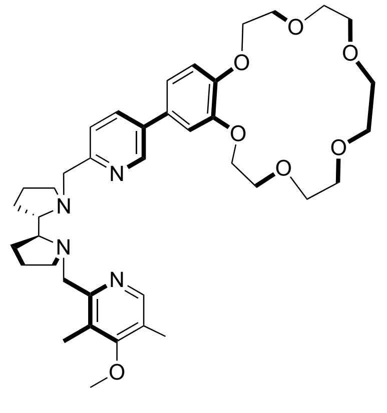

***(S,S)*-^{CR,DMM}pdp**. Prepared from ^{CR}py-Cl (64.0 mg, 0.145 mmol) and *(S,S)*-^{DMM}PhP (40.0 mg, 0.138 mmol) following the general procedure. After purification, the product was obtained as a clear oil (50.0 mg, 0.072 mmol, 52% yield). ^{1}H-NMR (400 MHz, $CDCl_3$) δ, ppm: 8.68 (dd, *J* = 2.4, 0.8 Hz, 1H), 8.16 (s, 1H), 7.76 (dd, *J* = 8.0, 2.3 Hz, 1H), 7.45 (dd, *J* = 5.4, 3.2 Hz, 1H), 7.16 – 7.06 (m, 2H), 6.97 (d, *J* = 8.1 Hz, 1H), 4.26 – 4.19 (m, 4H), 4.11 (d, *J* = 12.2 Hz, 2H), 3.99 – 3.93 (m, 4H), 3.81 – 3.77 (m, 4H), 3.75 (s, 3H), 3.74 – 3.71 (m, 4H), 3.70 – 3.67 (m, 4H), 3.54 – 3.37 (m, 2H), 3.08 – 2.93 (m, 1H), 2.88 – 2.61 (m, 3H), 2.31 (s, 3H), 2.24 (s, 3H), 1.90 – 1.55 (m, 10H). ^{13}C-NMR (100 MHz, $CDCl_3$) δ, ppm: 152.3, 149.4, 149.3, 148.2, 147.0, 134.8, 131.9, 131.1, 130.9, 125.5, 125.3, 120.1, 114.4, 113.2, 70.9, 70.7, 70.6, 69.6, 69.6, 69.4, 69.1, 60.3, 59.9, 55.1, 54.6, 54.1, 53.4, 29.7, 24.0, 23.9, 13.3, 10.7. HRMS (ESI-MS) *m/z* calculated for $C_{39}H_{54}N_4O_7$ $[M+H+Na]^{2+}$ 357.1979, found 357.1993.

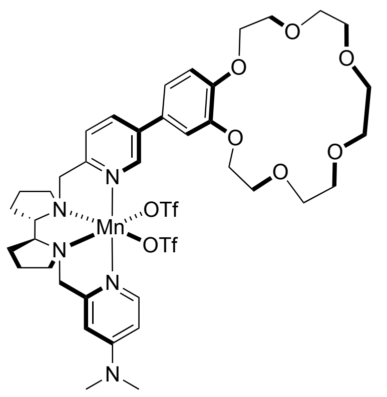

***(S,S)*-^{CR,Me2N}pdp**. Prepared from ^{CR}py-Cl (50.0 mg, 0.114 mmol) and *(S,S)*-^{Me2N}PhP (30.0 mg, 0.110 mmol) following the general procedure. After purification, the product was obtained as a clear oil (45.0 mg, 0.067 mmol, 61% yield). ^{1}H-NMR (400 MHz, $CDCl_3$) δ, ppm: 8.66 (d, *J* = 2.3 Hz, 1H), 8.12 (d, *J* = 5.9 Hz, 1H), 7.72 (dd, *J* = 8.1, 2.3 Hz, 1H), 7.42 (d, *J* = 8.1 Hz, 1H), 7.12 – 7.05 (m, 2H), 6.95 (d, *J* = 8.1 Hz, 1H), 6.66 (d, *J* = 2.6 Hz, 1H), 6.34 (dd, *J* = 6.0, 2.7 Hz, 1H), 4.30 – 4.19 (m, 5H), 4.10 (d, *J* =14.2 Hz, 1H), 3.96 – 3.92 (m, 4H), 3.77 (dd, *J* = 6.2, 3.3 Hz, 4H), 3.72 (dd, *J* = 6.1, 3.5 Hz, 4H), 3.68 (s, 4H), 3.49 (dd, *J* = 32.9, 14.2 Hz, 2H), 3.04 (ddd, *J* = 18.4, 9.3, 4.5 Hz, 2H), 2.94 (s, 6H), 2.88 – 2.67 (m, 2H), 2.26 (s, 2H), 1.89 – 1.66 (m, 8H). ^{13}C-NMR (100 MHz, $CDCl_3$) δ, ppm: 159.9, 158.7, 154.9, 149.3, 149.0, 148.7, 146.8, 134.4, 134.3, 131.2, 122.6, 120.0, 114.3, 113.1, 105.1, 105.0, 70.8, 70.7, 70.7, 69.6, 69.6, 69.3, 69.1, 65.6, 65.5, 61.4, 60.9, 55.3, 55.2, 39.1, 26.2, 26.1, 23.6, 23.6. HRMS (ESI-MS) *m/z* calculated for $C_{38}H_{53}N_5O_6$ $[M+H+Na]^{2+}$ 349.6980, found 349.6989.

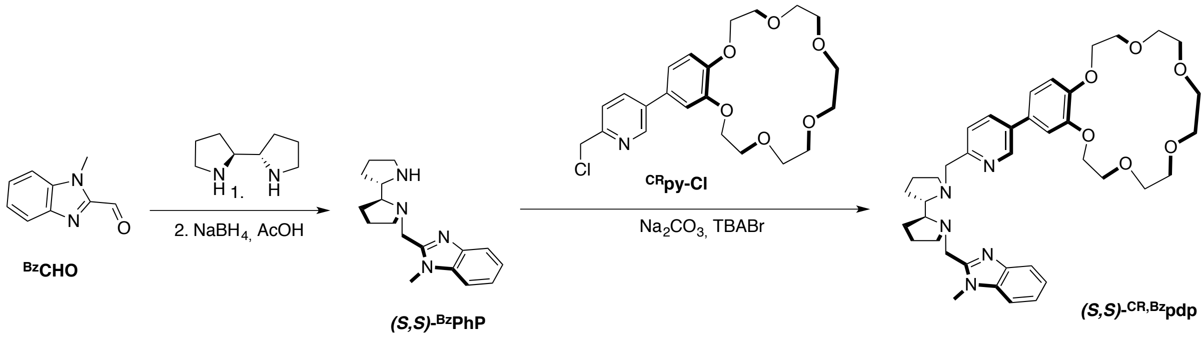

BzCHO was purchased from Sigma Aldrich. *(S,S)-*BzPhP was prepared according to a reported procedures.[4] *(S,S)-*CR,Bzpdp was synthesized from the corresponding *(S,S)-*BzPhP following the procedure described above.

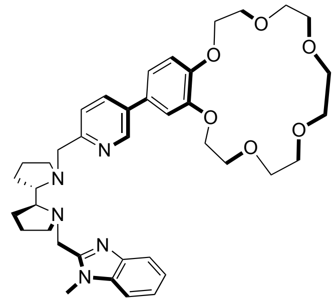

***(S,S)-*CR,Bzpdp**. Prepared from CRpy-Cl (40.0 mg, 0.091 mmol) and *(S,S)-*BzPhP (25.0 mg, 0.087 mmol) following the general procedure. After purification, the product was obtained as a clear oil (34.0 mg, 0.049 mmol, 55% yield). ^{1}H-NMR (400 MHz, $CDCl_3$) δ, ppm: 8.69 (s, 1H), 7.74 (t, *J* = 8.7 Hz, 2H), 7.42 (d, *J* = 8.1 Hz, 1H), 7.32 (d, *J* = 7.8 Hz, 1H), 7.29 – 7.21 (m, 3H), 7.14 – 7.07 (m, 2H), 6.96 (d, *J* = 8.2 Hz, 1H), 4.21 (ddd, *J* = 23.2, 18.8, 13.9 Hz, 6H), 3.95 (t, *J* = 4.6 Hz, 4H), 3.84 (s, 3H), 3.78 (d, *J* = 5.0 Hz, 4H), 3.73 (d, *J* = 5.0 Hz, 4H), 3.69 (s, 4H), 3.68 – 3.56 (m, 2H), 3.08 – 3.00 (m, 1H), 2.93 – 2.85 (m, 1H), 2.84 – 2.73 (m, 2H), 2.33 (dt, *J* = 20.4, 9.0 Hz, 2H), 1.88 – 1.62 (m, 8H). ^{13}C-NMR (100 MHz, $CDCl_3$) δ, ppm: 158.4, 152.6, 149.3, 149.1, 147.0, 142.2, 136.3, 134.6, 134.5, 131.1, 122.7, 122.4, 121.8, 120.0, 119.6, 114.3, 113.1, 109.0, 70.9, 70.7, 69.6, 69.6, 69.3, 69.1, 65.7, 64.6, 60.6, 55.4, 55.2, 52.6, 30.0, 26.0, 24.0, 23.5. HRMS (ESI-MS) *m/z* calculated for $C_{39}H_{51}N_5O_6$ $[M+H+Na]^{2+}$ 354.6902, found 354.6915.

2.2. Synthesis of the complexes

(S,S)-[Mn(OTf)2(TIPSpdp)],[1] *(S,S)-*[Mn(OTf)2(CRpdp)][2] and *(S,S)-*[Mn(OTf)2(CR,TMSpdp)][8] were prepared as previously according to a reported procedure. The other complexes were synthesized from the corresponding ligands following a reported procedure described below.[2]

Under N_2 atmosphere, the ligand ***(S,S)*** or ***(R,R)-* CR,Xpdp** (1 equiv.) was solved in anhydrous THF (1 mL) and solid $Mn(OTf)_2$, $Fe(OTf)_2(CH_3CN)_2$ or $Zn(OTf)_2$ (1 equiv.) was directly added. The reaction mixture was left under stirring overnight at room temperature. At this point, diethyl ether (5 mL) was added under vigorous stirring. A white precipitate is formed, and this was left deposit. The supernatant was removed and the solid was washed with diethyl ether (x2). The solid was dried under vacuum, dissolved in CH_2Cl_2, filtered over a plug of Celite© and

crystallized by layering this solution with hexane to afford, after few days, white crystals or yellow crystals.

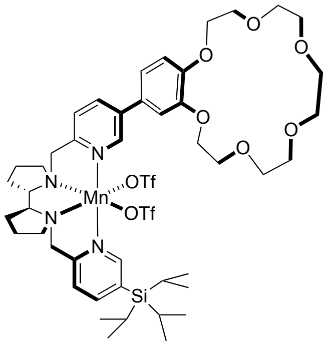

***(R,R)*-[Mn(OTf)$_2$(^{CR,TIPS}pdp)]**. Prepared from ***(R,R)*-^{CR,TIPS}pdp** (22.0 mg, 0.063 mmol) and Mn(OTf)$_2$ (50.0 mg, 0.063 mmol) following the general procedure. After crystallization, the product was obtained as white needles (65.0 mg, 0.058 mmol, 91% yield). Elemental analysis calculated (%) for $C_{47}H_{68}F_6MnN_4O_{12}S_2Si$ (MW = 1142.21): C 49.42, H 6.00, N 4.81, found C 49.46, H 6.03, N 4.78. FT-IR(ATR) ν, cm^{-1}: 2947, 2867, 1591, 1493, 1301, 1232, 1214, 1140, 1035, 634, 512. HRMS (ESI-MS) *m/z* calculated for $C_{46}H_{68}F_3MnN_4O_9SSi$ [M-OTf]$^+$ 992.3803, found 992.3799. XRD structure available on CCDC with deposition number 2119811.

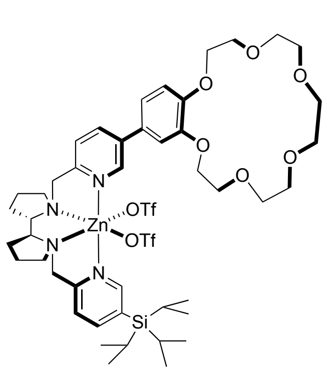

***(S,S)*-[Zn(OTf)$_2$(^{TIPS,CR}pdp)]**. Prepared from ***(S,S)*-^{CR,TIPS}pdp** (20.0 mg, 0.025 mmol) and Zn(OTf)$_2$ (9.0 mg, 0.025 mmol) following the general procedure. After crystallization, the product was obtained as white needles (27.0 mg, 0.024 mmol, 94% yield). ^{1}H-NMR (400 MHz, CDCl$_3$) δ, ppm: 9.01 (d, *J* = 2.2 Hz, 1H), 8.75 (s, 1H), 8.33 (dd, *J* = 8.2, 2.3 Hz, 1H), 8.23 – 8.15 (m, 1H), 7.54 (dd, *J* = 13.9, 8.0 Hz, 2H), 7.34 – 7.24 (m, 2H), 7.09 (d, *J* = 8.3 Hz, 1H), 4.25 (ddd, *J* = 22.2, 6.1, 3.3 Hz, 4H), 4.10 (d, *J* = 3.7 Hz, 4H), 3.86 – 3.75 (m, 4H), 3.69 – 3.60 (m, 4H), 3.60 – 3.56 (m, 4H), 3.55 (s, 4H), 3.02 (ddd, *J* = 22.3, 13.0, 8.0 Hz, 2H), 2.70 – 2.62 (m, 2H), 2.46 (td, 11.6, 7.9 Hz, 1H), 2.35 (td, *J* = 11.7, 7.9 Hz, 1H), 2.14 – 2.07 (m, 2H), 1.80 – 1.68 (m, 2H), 1.52 (dt, *J* = 14.8, 7.5 Hz, 3H), 1.42 (s, 2H), 1.11 (d, *J* = 7.5 Hz, 18H). ^{13}C-NMR (100 MHz, CDCl$_3$) δ, ppm: 156.1, 153.8, 152.8, 148.3, 145.6, 139.1, 138.3, 132.5, 128.9, 127.4, 126.0, 125.4, 120.6, 113.6, 111.7, 71.7, 71.0, 70.9, 69.7, 69.6, 68.9, 68.8, 67.7, 48.7, 58.3, 53.9, 53.6, 25.2, 25.1, 23.4, 23.3, 18.6, 18.6, 11.3. Elemental analysis calculated (%) for $C_{47}H_{68}F_6N_4O_{12}S_2SiZn$ (MW = 1152.65): C 48.98, H 5.95, N 4.86, found C 49.01, H 5.96, N 4.83. FT-IR(ATR) ν, cm^{-1}: 2945, 2868, 1617, 1492, 1305, 1235, 1216, 1149, 1028, 635, 514. HRMS (ESI-MS) *m/z* calculated for $C_{46}H_{68}F_3ZnN_4O_9SSi$ [M-OTf]$^+$ 1101.3714, found 1001.3696.

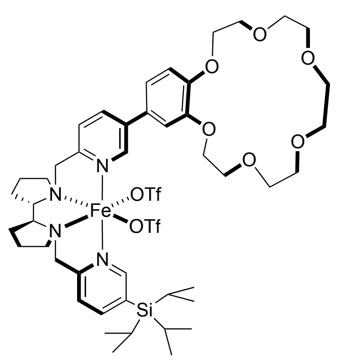

***(S,S)*-[Fe(OTf)$_2$(^{CR,TIPS}pdp)]**. Prepared from ***(S,S)*-^{CR,TIPS}pdp** (20.0 mg, 0.025 mmol) and Fe(OTf)$_2$(CH$_3$CN)$_2$ (10.9 mg, 0.025 mmol) following the general procedure. After crystallization, the product was obtained as yellow needles (25.0 mg, 0.22 mmol, 88% yield). Elemental analysis calculated (%) for $C_{47}H_{68}F_6FeN_4O_{12}S_2Si$ (MW = 1143.12): C 49.38, H 6.00, N 4.90, found C 49.41, H 6.02, N 4.85. FT-IR(ATR) ν, cm^{-1}: 2921, 2853, 1590, 1493, 1458, 1288, 1220,

1152, 1026, 948, 634, 514. HRMS (ESI-MS) *m/z* calculated for $C_{46}H_{68}F_3FeN_4O_9SSi$ $[M-OTf]^+$ 993.3819, found 993.3824.

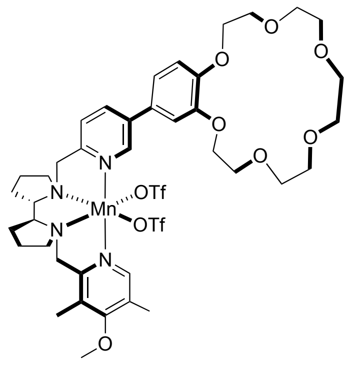

***(S,S)*-[Mn(OTf)$_2$(^{CR,DMM}pdp)]**. Prepared from ***(S,S)*-^{CR,DMM}pdp** (30.0 mg, 0.043 mmol) and $Mn(OTf)_2$ (30.0 mg, 0.043 mmol) following the general procedure. After crystallization, the product was obtained as white needles (38.0 mg, 0.036 mmol, 84% yield). Elemental analysis calculated (%) for $C_{41}H_{54}F_6MnN_4O_{13}S_2$ (MW = 1043.95): C 47.17, H 5.21, N 5.37, found C 47.19, H 5.21, N 5.36. FT-IR(ATR) ν, cm^{-1}: 2924, 1600, 1492, 1309, 1234, 1217, 1143, 1031, 636, 514. HRMS (ESI-MS) *m/z* calculated for $C_{40}H_{54}F_3MnN_4O_{10}S$ $[M-OTf+Na]^{2+}$ 458.6390, found 458.6394. XRD structure available on CCDC with deposition number 2119810.

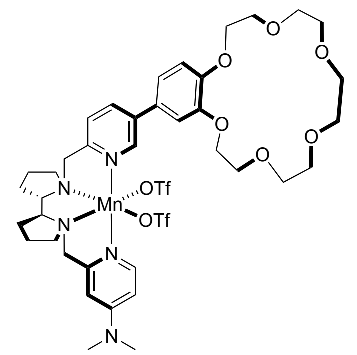

***(S,S)*-[Mn(OTf)$_2$(^{CR,Me2N}pdp)]**. Prepared from ***(S,S)*-^{CR,Me2N}pdp** (16.0 mg, 0.044 mmol) and $Mn(OTf)_2$ (30.0 mg, 0.043 mmol) following the general procedure. After crystallization, the product was obtained as white needles (37.0 mg, 0.036 mmol, 81% yield). Elemental analysis calculated (%) for $C_{40}H_{53}F_6MnN_5O_{12}S_2$ (MW = 1028.94): C 46.69, H 5.19, N 6.81, found C 46.74, H 5.23, N 6.79. FT-IR(ATR) ν, cm^{-1}: 2931, 2866, 1617, 1492, 1305, 1235, 1216, 1149, 1028, 635, 514. HRMS (ESI-MS) *m/z* calculated for $C_{39}H_{53}F_3MnN_5O_9S$ $[M-OTf]^+$ 879.2891, found 879.2899.

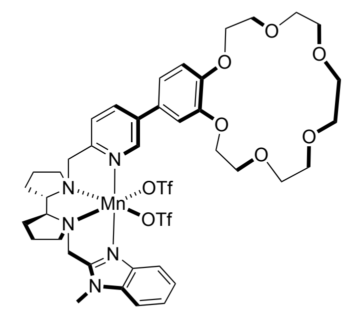

***(S,S)*-[Mn(OTf)$_2$(^{CR,Bz}pdp)]**. Prepared from ***(S,S)*-^{CR,Bz}pdp** (20.0 mg, 0.029 mmol) and $Mn(OTf)_2$ (10.0 mg, 0.029 mmol) following the general procedure. After crystallization, the product was obtained as white needles (27.0 mg, 0.026 mmol, 90% yield). Elemental analysis calculated (%) for $C_{41}H_{51}F_6MnN_5O_{12}S_2$ (MW = 1038.93): C 47.40, H 4.95, N 6.74, found C 47.43, H 4.98, N 6.72. FT-IR(ATR) ν, cm^{-1}: 2891, 1606, 1493, 1456, 1236, 1217, 1148, 1027, 897, 747, 634, 515. HRMS (ESI-MS) *m/z* calculated for $C_{40}H_{51}F_3MnN_5O_9S$ $[M-OTf]^+$ 889.2735, found 889.2750.

3. General procedures for the synthesis of the α,ω-amino acids

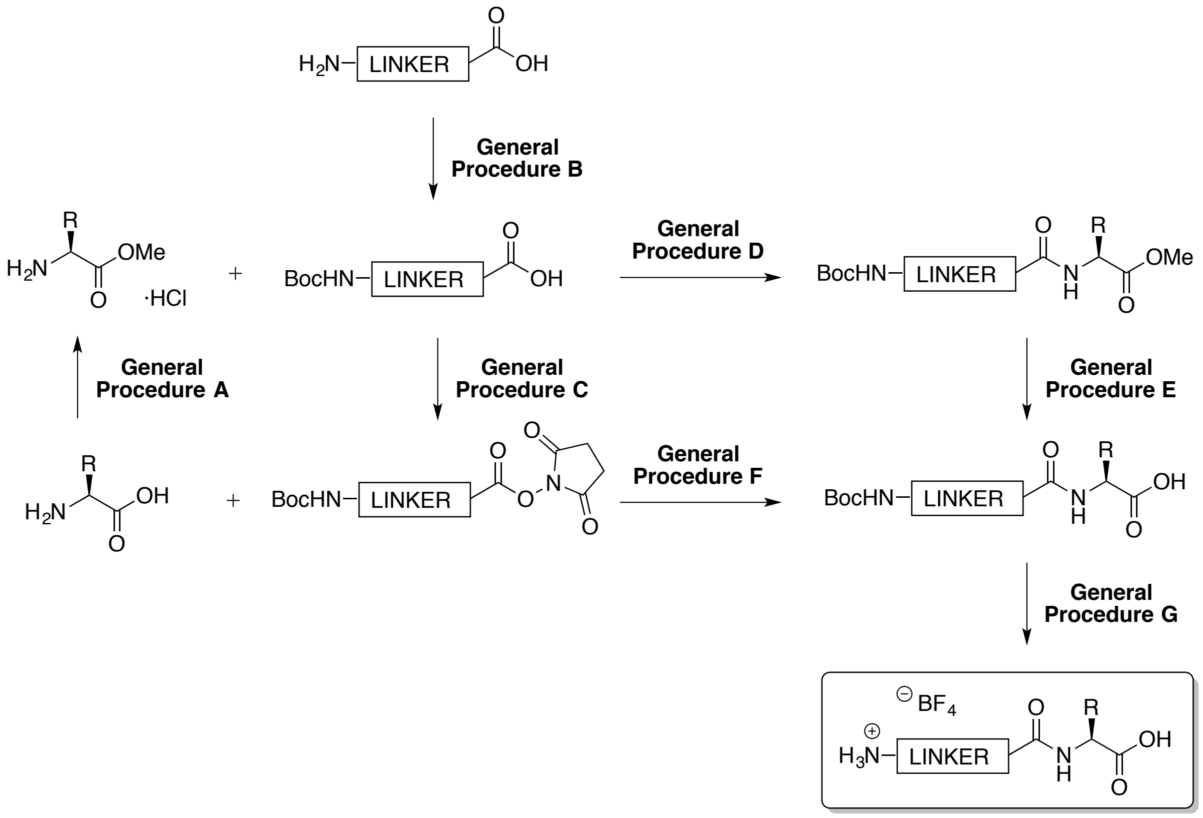

Scheme S1. General synthetic strategy for the synthesis of the α,ω-amino acids.

3.1. General Procedure A: Esterification of the amino acids

The amino acids were esterified following a slightly modified reported procedure.[9]

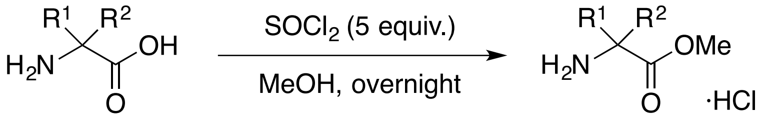

The amino acid was solved in MeOH (0.5M) and the solution was cooled to 0°C with an ice bath. Thionyl chloride (5 equiv.) was added dropwise and the mixture was stirred at room temperature overnight. After this time, the solvent was removed under pressure to give the corresponding amino acid methyl ester hydrochloride.

3.2. General Procedure B: Boc-protection of the linkers

The Boc- protection of the linkers was done following a slightly modified reported procedure.[10]

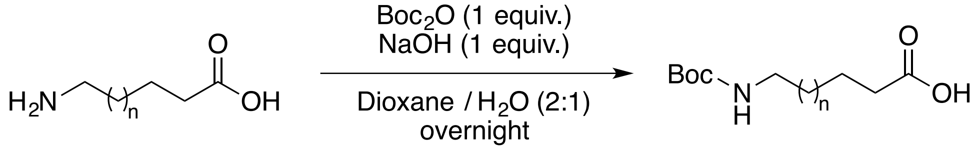

The amino acid (1 equiv.) and NaOH (1 equiv.) were solved in a dioxane/H_2O mixture (2:1, 0.35M). The mixture was cooled to 0°C with an ice bath and fitted with N_2 atmosphere. Boc_2O (1 equiv.) was added to the solution in three portions and the mixture was stirred at room temperature overnight. After this time, the solvent was removed under reduced pressure. The residue was solved in water and washed with EtOAc (x2). The combined aqueous layers were then acidified until pH=1-2 with 2M HCl. The solution was extracted with EtOAc (x3) and the combined organic layers were dried over anhydrous $MgSO_4$, filtered and the solvent was removed under reduced pressure to obtain the corresponding Boc-protected amino acid, which was used in the next step without further purification.

3.3. General Procedure C: Synthesis of succinimidyl esters

The synthesis of the succinimidyl esters was done following a slightly modified reported procedure.[11]

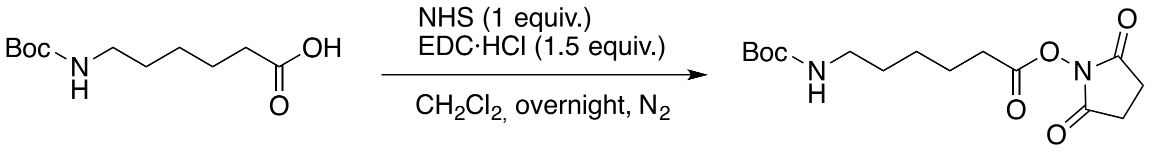

The Boc-protected amino acid (1 equiv.) was solved in anhydrous CH_2Cl_2 (0.1M) and 1 equiv. of *N*-hydroxysuccinimide were added. The reaction was fitted with N_2 atmosphere and 1.5 equiv. of EDC·HCl were added. The mixture was stirred at room temperature overnight. After this time, the reaction was quenched with water and stirred further for 10 minutes. The layers were separated and the aqueous phase was extracted with CH_2Cl_2 (x2). The organic layer was washed with brine (x3) and the combined organic layers were layers were dried over anhydrous $MgSO_4$, filtered and the solvent was removed under reduced pressure. The crude was purified by flash chromatography over silica gel to obtain the pure product.

3.4. General Procedure D: Coupling of the linker and the amino ester

The coupling of the Boc-protected linked and the amino esters was done following a slightly modified reported procedure.[12]

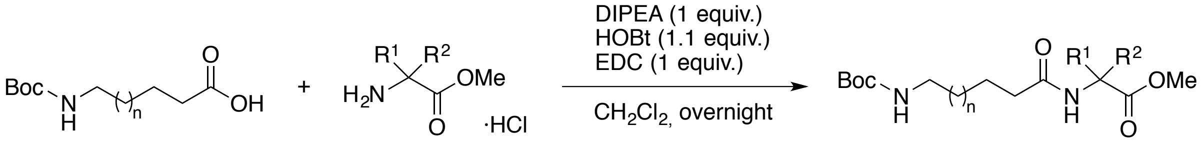

The amino acid methyl ester hydrochloride (1 equiv.) was disolved in CH_2Cl_2 (0.2M) and cooled to 0°C with an ice bath. Diisoropyl ethylamine (1 equiv.) was added dropwise. Next were added, in the following order, the N-Boc-protected amino acid (1 equiv.), HOBt (1.1 equiv.) and EDC·HCl (1 equiv.). The mixture was stirred at room temperature overnight. After this time, the reaction crude was washed with saturated $NaHCO_3$ (x2) and citric acid (10% w/w, x2), and extracted with CH_2Cl_2. The combined organic layers were dried over anhydrous $MgSO_4$, filtered and the solvent was removed under reduced pressure. The crude was purified by flash chromatography over silica gel to obtain the pure products.

3.5. General Procedure E: Hydrolysis of the ester

The hydrolysis of the esters was done following a slightly modified reported procedure.[13]

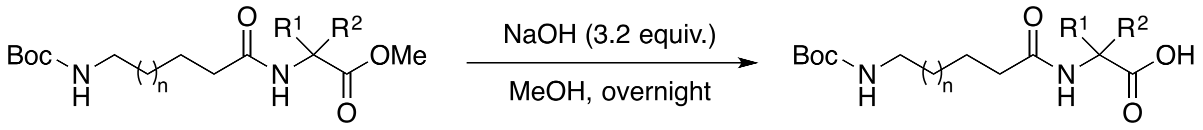

The ester (1 equiv.) was dissolved in MeOH (0.2M) and 3.2 equiv. of 2M NaOH solution were added. The solution was stirred at room temperature overnight. After this time, the solvent was removed under reduced pressure. The resulting residue was dissolved in water and acidified to pH=2-3 with 2M HCl. The solution was extracted with EtOAc (x3) and the combined organic layers were dried over anhydrous $MgSO_4$, filtered and the solvent was removed under reduced pressure. The crude was dissolved with the minimum amount of CH_2Cl_2 and crystallized by layering this solution with hexane overnight to afford the pure carboxylic acid as a colorless solid.

3.6. General Procedure F: Coupling of the succinimidyl esters and the amino acids

The coupling of the succinimidyl esters and the amino acids was done following a slightly modified reported procedure.[14]

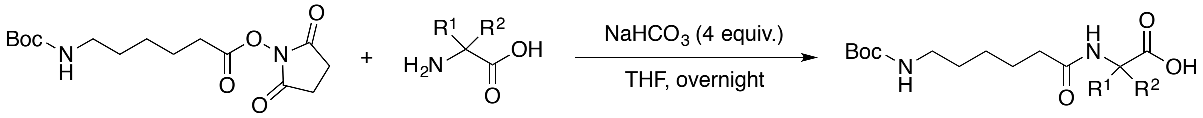

The amino acid (1 equiv.) was disolved in THF (0.2M) and 4 equiv. of 1M $NaHCO_3$ solution were added. The succinimidyl ester (1 equiv.) was added and the mixture was stirred at room temperature overnight. After this time, the solution was diluted with CH_2Cl_2/MeOH (80:20) and the phases were separated. The aqueous phase was acidified with with citric acid (10% w/w, x2) until pH=2 and extracted with EtOAc (x3). The combined organic layers were dried over anhydrous $MgSO_4$, filtered and the solvent was removed under reduced pressure to obtain the product, which was used in the next step without further purification.

3.7. General Procedure G: Deprotection of the Boc and protonation of the amine

The deprotection of the Boc and protonation of the amines were done following a slightly modified reported procedure.[2]

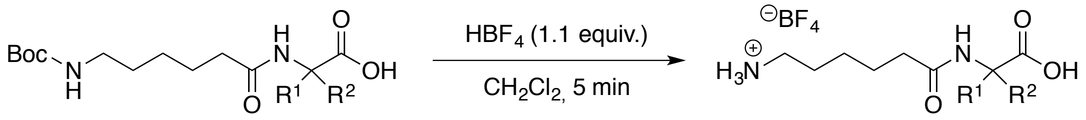

The Boc-protected amine (1 equiv.) was dissolved in CH_2Cl_2 (0.02M) and cooled to 0°C with an ice bath under vigorously stirring. A 0.25M CH_2Cl_2 solution of tetrafluoroboric acid diethyl ether complex (1.1 equiv.) was added dropwise to the solution and the reaction was stirred for 5 minutes at 0°C. After this time, the solvent was removed under reduced pressure to obtain the corresponding protonated salt. The solid was then suspended in diethyl ether (8 mL) and vigorously stirred for 5 minutes. The supernatant solution was removed and the washing process was repeated three times. Finally, the solid was dried under vacuum to obtain the corresponding protonated amine.

4. Synthesis and characterization of the α,ω-amino acids

4.1. C_6-Tle (1)

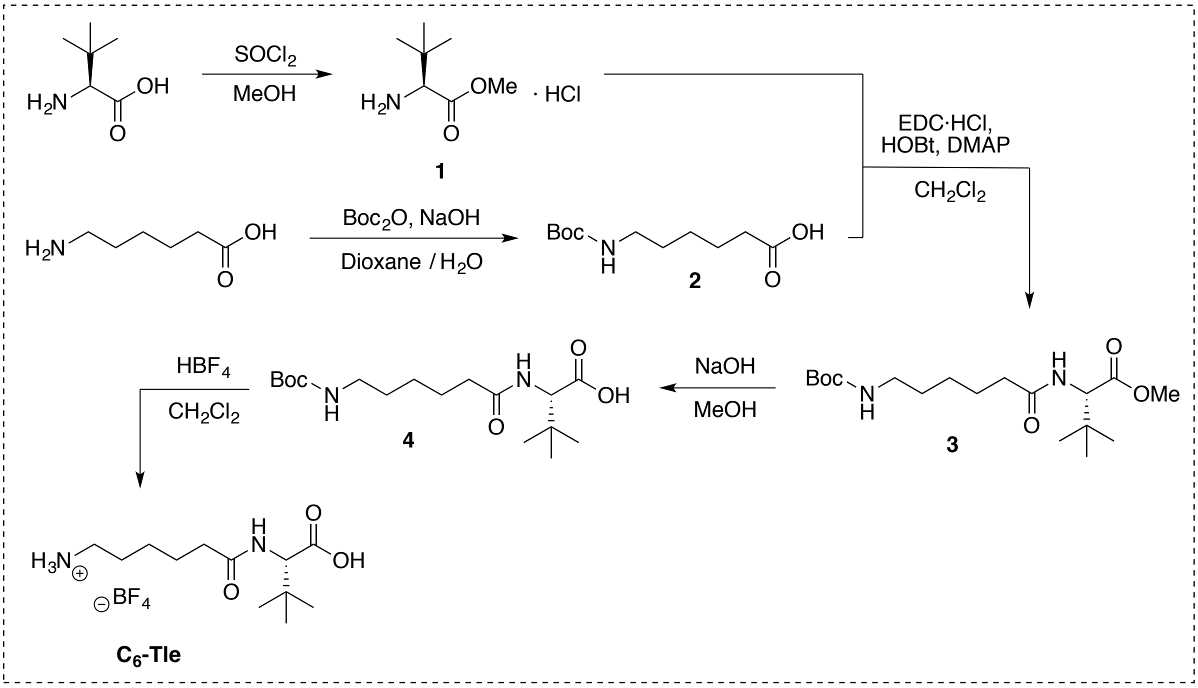

Scheme S2. Synthetic pathway for the synthesis of C_6-Tle (Figure 5, **1**).

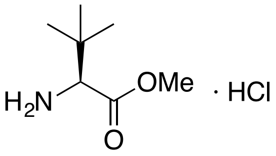

1

Methyl *(S)*-2-amino-3,3-dimethylbutanoate hydrochloride (1). Prepared from *L*-tert-leucine (565.0 mg, 4.18 mmol) following the general procedure A. The product was obtained as a colorless solid (736.2 mg, 4.05 mmol, 97% yield). Spectral data match those reported in literature.[15] ^{1}H-NMR (300 MHz, CD_3OD) δ, ppm: 3.86 (s, 3H), 3.83 (s, 1H), 1.12 (s, 9H).

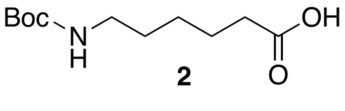

6-((*tert*-butoxycarbonyl)amino)hexanoic acid (2). Prepared from 6-aminohexanoic acid (6.00 g, 45.7 mmol) following the general procedure B. The product was obtained as an colorless liquid (10.5 g, 45.4 mmol, 99% yield). Spectral data match those reported in literature.[16] ^{1}H-NMR (400 MHz, $CDCl_3$) δ, ppm: 4.57 (br, 1H), 3.18 – 3.05 (m, 2H), 2.37 (t, *J* = 7.2 Hz, 2H), 1.71 – 1.61 (m, 2H), 1.58 – 1.33 (m, 4H), 1.46 (s, 9H)

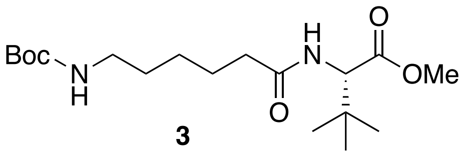

3

Methyl *(S)*-2-(6-((*tert*-butoxycarbonyl)amino) hexanamido)-3,3-dimethylbutanoate (3). Prepared from **1** (301.9 mg, 2.08 mmol) and **2** (537.4 mg, 2.08 mmol) following the general procedure D. Purification by flash chromatography over silica gel (hexane:EtOAc, 6:4) affords the product as a colorless solid (697.6 mg, 1.95 mmol, 94% yield). ^{1}H-NMR (400 MHz, $CDCl_3$) δ, ppm: 6.00 (d, *J* = 9.4 Hz, 1H), 4.58 (bs, 1H), 4.50 (d, *J* = 9.4 Hz, 1H), 3.74 (s, 3H), 3.12 (q, *J* = 6.5 Hz, 2H), 2.25 (t, *J* = 7.5 Hz, 2H), 1.68 (quint, *J* = 7.5 Hz, 2H), 1.57 – 1.48 (m, 2H), 1.45 (s, 9H), 1.42 – 1.29 (m, 2H), 0.98 (s, 3H). ^{13}C-NMR (100 MHz, $CDCl_3$) δ, ppm: 172.5, 172.4, 156.0, 79.0, 59.7, 51.8, 40.3, 36.5, 34.7, 29.7, 28.4, 26.6, 26.4, 25.2. HRMS (ESI-MS) *m/z* calculated for $C_{18}H_{34}N_2O_5$ $[M-H]^-$ 357.2395, found 357.2387.

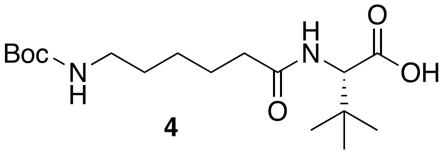

4

***(S)*-2-(6-((*tert*-butoxycarbonyl)amino)hexanamido)-3,3-dimethylbutanoic acid (4).** Prepared from **3** (132.2 mg, 0.369 mmol) following the general procedure E. The product was obtained as a colorless solid (72.1 mg, 0.209 mmol, 57% yield). ^{1}H-NMR (400 MHz, CD_3OD) δ, ppm: 4.31 (s, 1H), 3.04 (t, *J* = 7.0 Hz, 2H), 2.32 (td, *J* = 7.4 Hz, 1.5 Hz, 2H), 1.65 (quint, *J* = 7.4 Hz, 2H), 1.56 – 1.48 (m, 2H), 1.45 (s, 9H), 1.42 – 1.31 (m, 2H), 1.04 (s, 9H). ^{13}C-NMR (100 MHz, CD_3OD) δ, ppm: 174.7, 172.9, 157.1, 78.4, 60.6, 39.8, 35.1, 33.4, 29.2, 27.4, 26.0, 25.8, 25.3. HRMS (ESI-MS) *m/z* calculated for $C_{17}H_{32}N_2O_5$ $[M-H]^-$ 343.2238, found 343.2235.

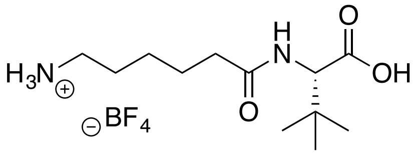

C_6-Tle

***(S)*-(6-((1-carboxy-2,2-dimethylpropyl)amino)-6-oxohexan-1-aminium tetrafluoroborate (C_6-Tle).** Prepared from **4** (45.0 mg, 0.131 mmol) following the general procedure G. The product was obtained as a colorless solid (26.5 mg, 0.080 mmol, 61% yield). ^{1}H-NMR (400 MHz, CD_3CN) δ, ppm: 6.66 (d, *J* = 8.7 Hz, 1H), 6.27 (bs, 3H), 4.22 (d, *J* = 8.7 Hz, 1H), 3.09 – 2.88 (m, 2H), 2.28 (t, *J* = 7.5 Hz, 1H), 1.74 – 1.53 (m, 4H), 1.44 – 1.31 (m, 2H), 1.15 (t, *J* = 7.0 Hz, 1H), 1.02 (s, 9H) ^{13}C-NMR (100 MHz, CD_3CN) δ, ppm: 173.2, 171.9, 60.4, 40.0, 34.8, 33.3, 26.0, 25.9, 25.0, 24.2. HRMS (ESI-MS) *m/z* calculated for $C_{12}H_{24}N_2O_3$ $[M-H]^-$ 243.1714, found 243.1707.

4.2. C_5-Ile (2)

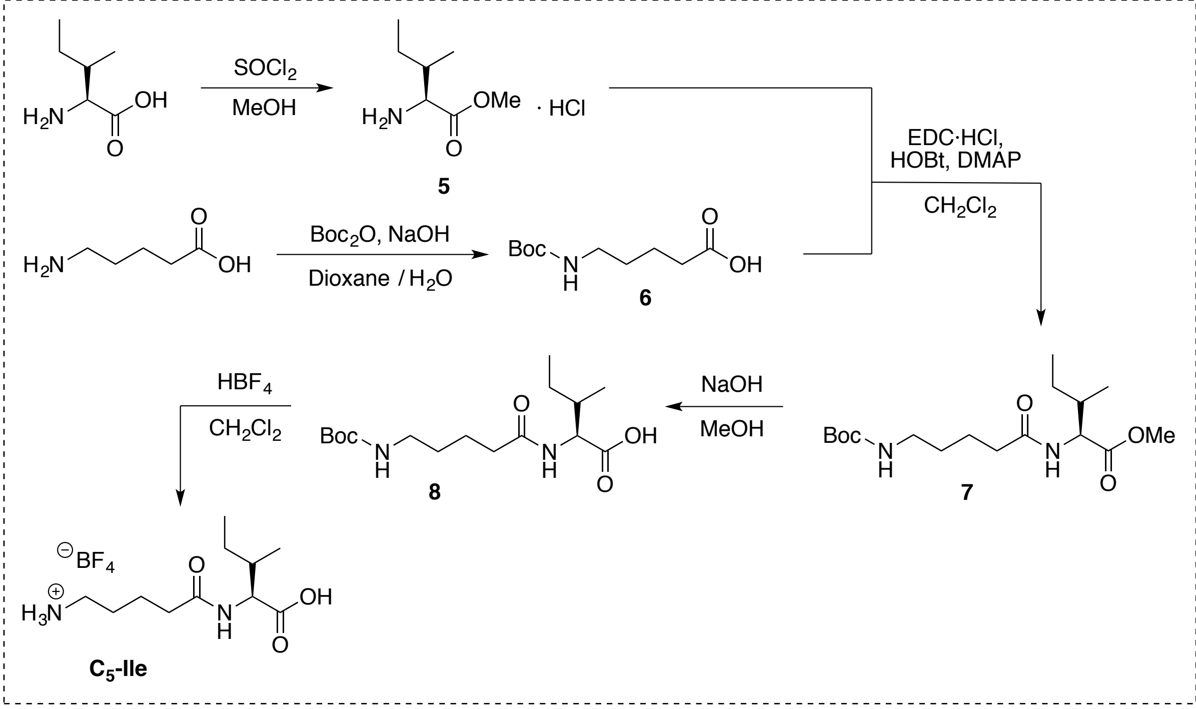

Scheme S3. Synthetic pathway for the synthesis of C_5-Ile (Figure 5, **2**).

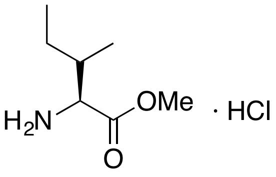

5

Methyl-*L*-isoleucinate hydrochloride (5). Prepared from *L*-isoleucine (669.8 mg, 5.00 mmol) following the general procedure A. The product was obtained as a colorless solid (905.2 mg, 4.98 mmol, 99% yield). Spectral data match those reported in literature.[17] ^{1}H-NMR (300 MHz, CD_3OD) δ, ppm: 3.98 (d, *J* = 3.6 Hz, 1H), 3.85 (s, 3H), 2.05 – 1.94 (m, 1H), 1.67 – 1.49 (m, 1H), 1.49 – 1.30 (m, 1H), 1.10 – 0.94 (m, 6H).

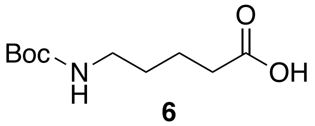

6

5-((*tert*-butoxycarbonyl)amino)pentanoic acid (6). Prepared from 5-aminopentanoic acid (1.00 g, 8.55 mmol) following the general procedure B. The solid was obtained as an colorless liquid (1.85 g, 8.51 mmol, quantitative). Spectral data match those reported in literature.[18] ^{1}H-NMR (400 MHz, $CDCl_3$) δ, ppm: 4.60 (br, 1H), 3.22 – 3.00 (m, 2H), 2.37 (t, *J* = 7.3 Hz, 2H), 1.71 – 1.60 (m, 2H), 1.60 – 1.49 (m, 2H), 1.47 (s, 9H).

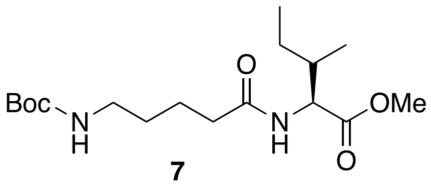

7

Methyl (5-((*tert*-butoxycarbonyl)amino)pentanoyl)-*L*-isoleucinate (7). Prepared from **5** (674.0 mg, 3.71 mmol) and **6** (806.1 mg, 3.71 mmol) following the general procedure D. Purification by flash chromatography over silica gel (hexane:EtOAc, 6:4) affords the product as a clear oil (377.1 mg, 1.09 mmol, 29% yield) with some small impurities. The product was taken on to subsequent synthetic steps during the course of which, the impurities were removed. ^{1}H-NMR (400 MHz, $CDCl_3$) δ, ppm: 6.07 (d, *J* = 8.7 Hz, 1H), 4.75 – 4.53 (m, 2H), 3.75 (s, 3H), 3.15 (q, *J* = 6.6 Hz, 2H), 2.28 (t, *J* = 7.4 Hz, 2H), 1.95 – 1.82 (m, 2H), 1.75 – 1.63 (m, 2H), 1.63 – 1.48 (m, 2H), 1.48 – 1.43 (m, 1H), 1.45 (s, 9H), 0.94 (t, *J* = 7.4 Hz, 3H), 0.92 (d, *J* = 6.8 Hz, 3H). ^{13}C-NMR (100 MHz, $CDCl_3$) δ, ppm: 176.7, 172.7, 156.1, 79.2, 56.3, 52.1, 37.0, 35.9, 33.3, 29.4, 28.4, 25.2, 22.6, 15.4, 11.6. HRMS (ESI-MS) *m/z* calculated for $C_{17}H_{32}N_2O_5$ $[M+Na]^+$ 367.2203, found 367.2217.

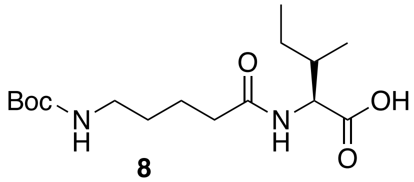

8

(5-((*tert*-butoxycarbonyl)amino)pentanoyl)-*L*-isoleucine (8). Prepared from **7** (90.0 mg, 0.261 mmol) following the general procedure E. The product was obtained as a colorless solid (48.6 mg, 0.147 mmol, 56% yield). ^{1}H-NMR (400 MHz, CD_3OD) δ, ppm: 4.37 (d, *J* = 5.6 Hz, 1H), 3.06 (t, *J* = 6.8 Hz, 2H), 2.30 (t, *J* = 7.4 Hz, 2H), 1.96 – 1.82 (m, 1H), 1.71 – 1.58 (m, 2H), 1.57 – 1.37 (m, 3H), 1.45 (s, 9H), 1.31 – 1.19 (m, 1H), 0.97 (d, *J* = 6.8 Hz, 3H), 0.95 (t, *J* = 7.4 Hz, 3H). ^{13}C-NMR (100 MHz, CD_3OD) δ, ppm: 174.7, 174.0, 157.2, 78.5, 57.0, 39.5, 36.9, 34.9, 29., 27.4, 24.8, 22.8, 14.7, 10.4. HRMS (ESI-MS) *m/z* calculated for $C_{16}H_{30}N_2O_5$ $[M-H]^-$ 329.2082, found 329.2073.

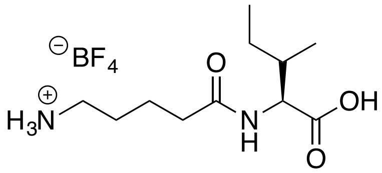

C_5-Ile

5-(((*1S,2S)*-1-carboxy-2-methylbutyl)amino)-5-oxopentan-1-aminium tetrafluoroborate (C_5-Ile). Prepared from **8** (32.5 mg, 0.098 mmol) following the general procedure G. The product was obtained as a colorless solid (15.6 mg, 0.049 mmol, 50% yield). ^{1}H-NMR (400 MHz, CD_3CN) δ, ppm: 6.78 (br-s, 1H), 6.45 (br-s, 3H), 4.32 (dd, *J* = 8.1, 5.6 Hz, 1H), 3.01 – 2.93 (m, 2H), 2.35 – 2.29 (m, 2H), 1.92 – 1.81 (m, 1H), 1.75 – 1.63 (m, 4H), 1.56 – 1.43 (m, 2H), 1.30 – 1.17 (m, 1H), 0.95 (d, *J* = 6.9 Hz, 3H), 0.94 (t, *J* = 7.4 Hz, 3H). ^{13}C-NMR (100 MHz, CD_3CN) δ, ppm: 173.3, 172.7, 56.8, 39.9, 36.6, 34.8, 26.0, 25.0, 24.0, 15.0, 10.8. HRMS (ESI-MS) *m/z* calculated for $C_{11}H_{22}N_2O_3$ $[M-H]^-$ 229.1558, found 229.1555.

4.3. C_6-Ile (3)

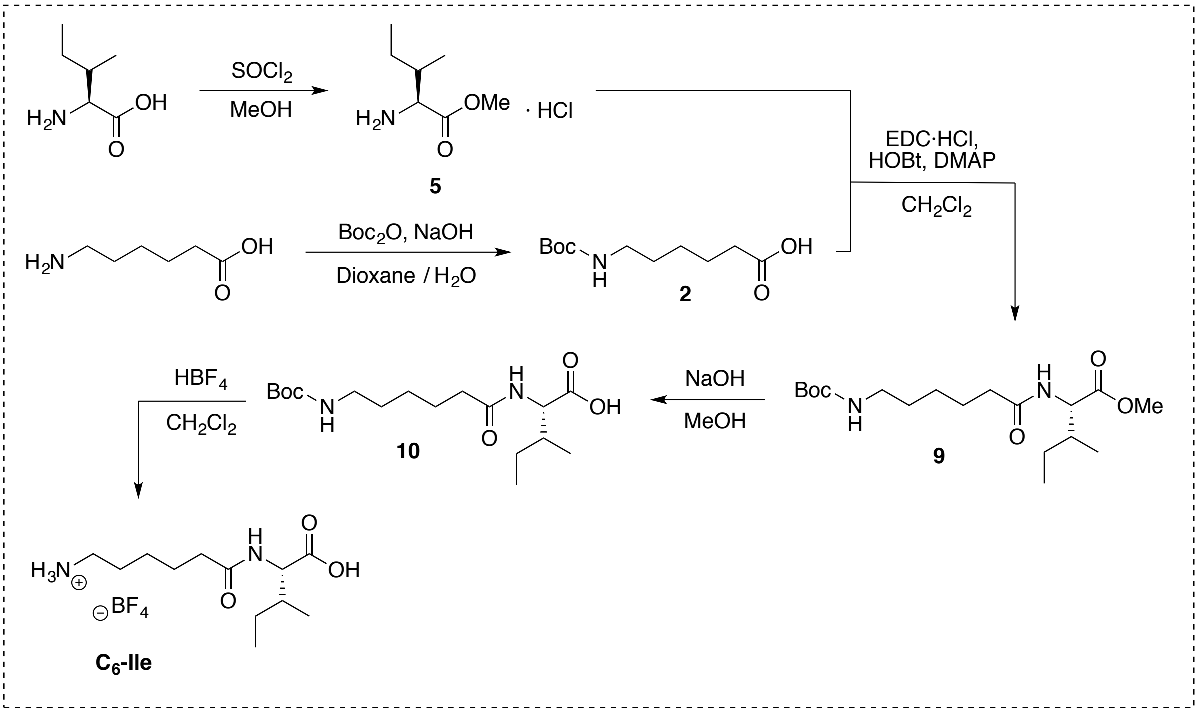

Scheme S4. Synthetic pathway for the synthesis of C_6-Ile (Figure 5, **3**).

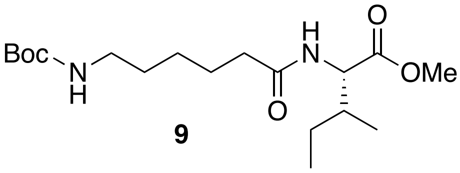

9

Methyl (6-((*tert*-butoxycarbonyl)amino)hexanoyl)-*L*-isoleucinate (9). Prepared from **2** (275.9 mg, 1.54 mmol) and **5** (334.1 mg, 1.54 mmol) following the general procedure D. Purification by flash chromatography over silica gel (hexane:EtOAc, 6:4) affords the product as a clear oil (342.0 mg, 0.993 mmol, 64% yield). ^{1}H-NMR (400 MHz, $CDCl_3$) δ, ppm: 6.00 (d, *J* = 8.5 Hz, 1H), 4.80 – 4.45 (m, 2H), 3.74 (s, 3H), 3.12 (q, *J* = 6.7 Hz, 2H), 2.24 (t, *J* = 7.5 Hz, 2H), 1.98 – 1.73 (m, 2H), 1.73 – 1.63 (m, 2H), 1.56 – 1.48 (m, 2H), 1.45 (s, 9H), 1.41 – 1.31 (m, 2H), 1.25 – 1.11 (m, 1H), 0.93 (t, *J* = 7.5 Hz, 3H), 0.91 (d, *J* = 6.8 Hz, 3H). ^{13}C-NMR (100 MHz, $CDCl_3$) δ, ppm: 172.7, 172.6, 156.0, 79.0, 56.2, 52.1, 40.3, 37.9, 36.4, 29.7, 28.4, 26.3, 25.2, 25.2, 15.4, 11.6. HRMS (ESI-MS) *m/z* calculated for $C_{18}H_{34}N_2O_5$ $[M+Na]^+$ 381.2360, found 381.2364.

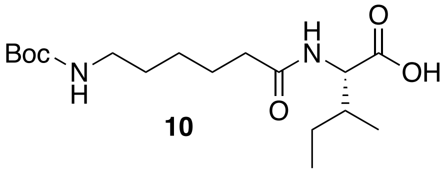

10

(6-((*tert*-butoxycarbonyl)amino)hexanoyl)-*L*-isoleucine (10). Prepared from **9** (208.9 mg, 0.583 mmol) following the general procedure E. The product was obtained as a colorless solid 66.6 mg, 0.193 mmol, 33% yield). ^{1}H-NMR (400 MHz, CD_3OD)

δ, ppm: 4.38 (d, *J* = 5.8 Hz, 1H), 3.04 (t, *J* = 7.0 Hz, 2H), 2.29 (t, *J* = 7.4 Hz, 2H), 1.97 – 1.84 (m, 1H), 1.65 (quint, *J* = 7.5 Hz), 1.56 – 1.43 (m, 12H), 1.42 – 1.33 (m, 2H), 0.97 (d, *J* = 6.9 Hz, 3H), 0.95 (t, *J* = 6.4 Hz, 3H). ^{13}C-NMR (100 MHz, CD_3OD) δ, ppm: 174.9, 173.6, 157.2, 78.4, 56.7, 39.8, 36.8, 35.2, 29.2, 27.4, 26.0, 25.3, 24.9, 14.7, 10.4. HRMS (ESI-MS) *m/z* calculated for $C_{17}H_{32}N_2O_5$ [M-H]$^-$ 343.2238, found 343.2230.

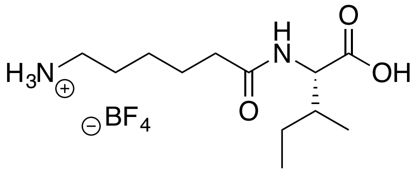

C_6-Ile

6-(((*1S,2S)*-1-carboxy-2-methylbutyl)amino)-6-oxohexan-1-aminium tetrafluoroborate (C_6-Ile). Prepared from **10** (25.0 mg, 0.073 mmol) following the general procedure G. The product was obtained as a colorless solid (18.8 mg, 0.057 mmol, 78% yield). ^{1}H-NMR (400 MHz, CD_3CN) δ, ppm: 6.69 (d, *J* = 8.1 Hz, 1H), 6.28 (br-s, 3H), 4.31 (dd, *J* = 8.1, 5.8 Hz, 1H), 3.06 – 2.90 (m, 2H), 2.26 (t, *J* = 7.2 Hz, 2H), 1.92 – 1.79 (m, 1H), 1.72 – 1.54 (m, 4H), 1.54 – 1.46 (m, 2H), 1.43 – 1.34 (m, 2H), 1.29 – 1.19 (m, 1H), 0.95 (d, *J* = 6.9 Hz, 3H), 0.93 (t, *J* = 7.3 Hz, 3H). ^{13}C-NMR (100 MHz, CD_3CN) δ, ppm: 173.4, 172.4, 56.6, 40.0, 36.6, 34.8, 26.0, 25.0, 24.9, 24.2, 15.0, 10.8. HRMS (ESI-MS) *m/z* calculated for $C_{12}H_{24}N_2O_3$ [M-H]$^+$ 243.1714, found 243.1709.

4.4. C_7-Ile (4)

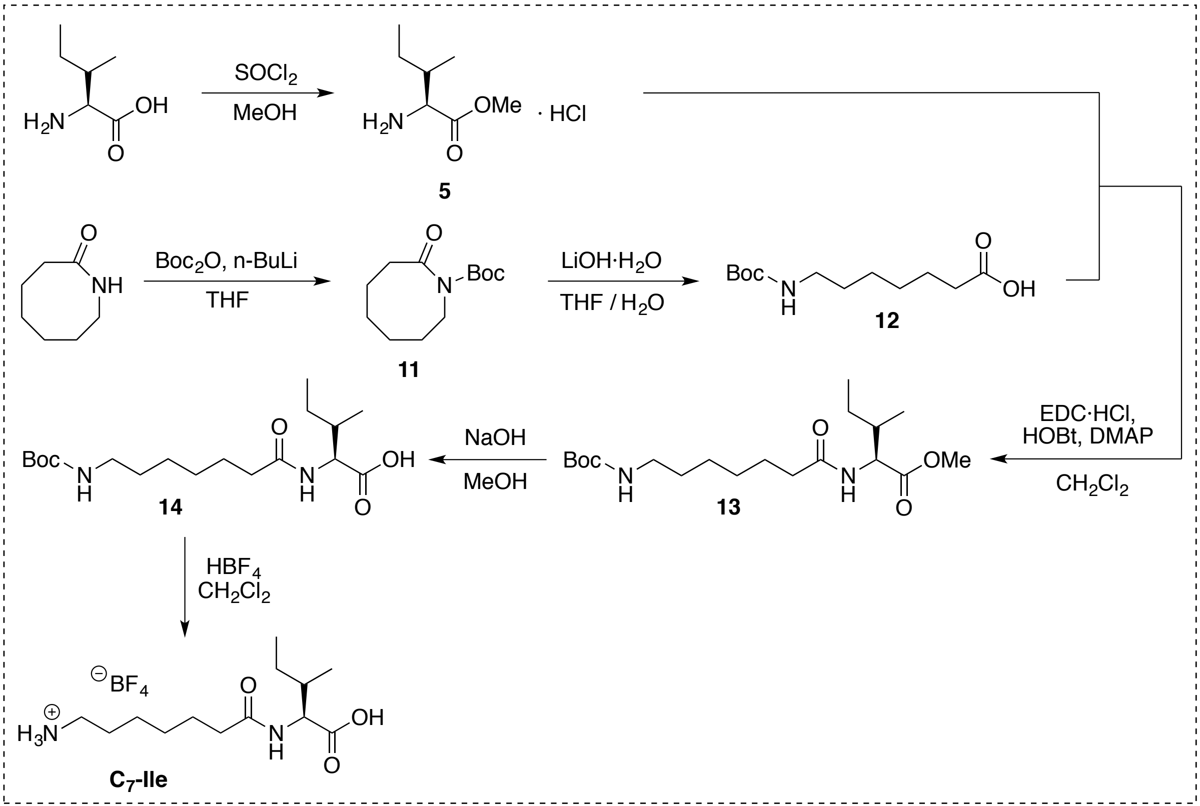

Scheme S5 Synthetic pathway for the synthesis of C_7-Ile (Figure 5, **4**).

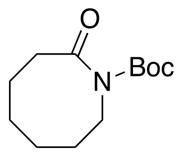

11 ***tert*-butyl 2-oxoazocane-1-carboxylate (11).** It was prepared according to a slightly modified reported procedure.[19] 2-azacyclooctanone (391.8 mg, 2.92 mmol) was dissolved in anhydrous THF (30 mL, 0.1M) and cooled to -78°C using an EtOAc/N_2 bath. Under N_2 atmosphere, 1 equiv. of n-BuLi (1.8 mL, 2.92 mmol) was added dropwise. Then, 1 equiv. of Boc_2O (651.0 mg, 2.92 mmol) was added slowly as a solution in anhydrous THF (0.1M). The reaction mixture was allowed to warm to room temperature and stirred overnight. After this time, the reaction was quenched with H_2O (30 mL) and extracted with CH_2Cl_2 (3 x 30 mL). The combined organic layers were dried over anhydrous $MgSO_4$, filtered and the solvent was removed under reduced pressure. Purification by flash chromatography over silica gel (hexane:EtOAc, 8:2) affords the product as a clear oil (446.7 mg, 1.97 mmol, 67% yield). Spectral data match those reported in literature.[19] ^{1}H-NMR (400 MHz, $CDCl_3$) δ, ppm: 3.86 – 3.77 (m, 2H), 2.65 – 2.56 (m, 2H), 1.91 – 1.82 (m, 2H), 1.81 – 1.71 (m, 2H), 1.64 – 1.59 (m, 2H), 1.59 – 1.49 (m, 2H), 1.54 (s, 9H).

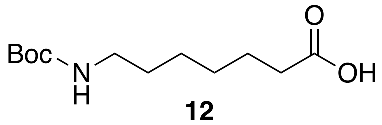

12 **7-((*tert*-butoxycarbonyl)amino)heptenoic acid (12).** It was prepared according to a slightly modified reported procedure.[20] **11** (487.5 mg, 2.14 mmol, 1 equiv.) was dissolved in a THF/H_2O (2:1, 30 mL, 0.07M). 3 equiv. of LiOH·H_2O was added (275.5 mg, 6.43 mmol) and the mixture was stirred at room temperature for 30 minutes. After this time, the reaction was quenched with saturated aqueous NH_4Cl (5 mL), diluted with Et_2O (20 mL) and acidified with 2 mL of 2M HCl. The phases were separated and the aqueous phase was extracted with Et_2O (2 x 20 mL). The combined organic layers were dried over anhydrous $MgSO_4$, filtered and the solvent was removed under reduced pressure. The product was obtained as a colorless solid (458.2 mg, 1.87 mmol, 87% yield) and used in the next step without further purification. Spectral data match those reported in literature.[21] ^{1}H-NMR (400 MHz, $CDCl_3$) δ, ppm: 4.53 (br, 1H), 3.19 – 3.02 (m, 2H), 2.37 (t, J = 7.4 Hz, 2H), 1.73 – 1.59 (m, 2H), 1.55 – 1.43 (m, 2H), 1.47 (s, 9H), 1.42 – 1.32 (m, 4H).

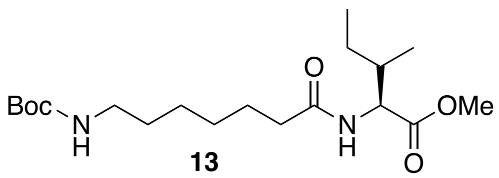

13 **Methyl (7-((*tert*-butoxycarbonyl)amino)heptanoyl)-*L*-isoleucinate (13).** Prepared from **5** (53.2 mg, 0.293 mmol) and **11** (71.8 mg, 0.293 mmol) following the general procedure D. Purification by flash chromatography over silica gel (hexane:EtOAc, 8:2 to 6:4) affords the product as a clear oil (65.6 mg, 0.176 mmol, 60% yield) with some small impurities. The product was taken on to subsequent synthetic steps during

the course of which, the impurities were removed. ^{1}H-NMR (400 MHz, $CDCl_3$) δ, ppm: 6.08 (bs, 1H), 4.68 – 4.50 (m, 2H), 3.74 (s, 3H), 3.17 – 3.05 (m, 2H), 2.24 (t, *J* = 7.5 Hz, 2H), 1.06 – 1.82 (m, 1H), 1.69 – 1.61 (m, 2H), 1.50 – 1.43 (m, 3H), 1.45 (s, 9H), 1.37 – 1.32 (m, 4H), 1.23 – 1.12 (m, 1H), 0.96 – 0.87 (m, 6H). ^{13}C-NMR (100 MHz, $CDCl_3$) δ, ppm: 172.9, 172.8, 156.0, 79.0, 56.2, 52.1, 40.4, 37.9, 36.4, 29.9, 28.7, 28.4, 26.4, 25.5, 25.2, 15.4, 11.6. HRMS (ESI-MS) *m/z* calculated for $C_{19}H_{36}N_2O_5$ $[M+Na]^+$ 395.2516, found 395.2530.

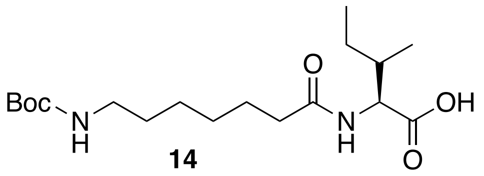

14

(7-((*tert*-butoxycarbonyl)amino)heptanoyl)-*L*-isoleucine (14). Prepared from **13** (65.5 mg, 0.176 mmol) following the general procedure E. The product was obtained as a colorless solid (41.6 mg, 0.116 mmol, 66% yield). ^{1}H-NMR (400 MHz, CD_3OD) δ, ppm: 4.38 (d, *J* = 5.8 Hz, 1H), 3.04 (t, *J* = 7.0 Hz, 2H), 2.29 (t, *J* = 7.3 Hz, 2H), 1.96 – 1.83 (m, 1H), 1.67 – 1.58 (m, 2H), 1.49 – 1.42 (m, 3H), 1.45 (s, 9H), 1.38 (m, 4H), 1.31 – 1.22 (m, 1H), 0.97 (d, *J* = 6.9 Hz, 3H), 0.95 (t, *J* = 7.4 Hz, 3H). ^{13}C-NMR (100 MHz, CD_3OD) δ, ppm: 175.0, 173.6, 157.2, 78.4, 56.7, 39.9, 36.8, 35.2, 29.4, 28.5, 27.4, 26.1, 25.5, 24.9, 14.7, 10.4. HRMS (ESI-MS) *m/z* calculated for $C_{18}H_{34}N_2O_5$ $[M-H]^-$ 357.2395, found 357.2388.

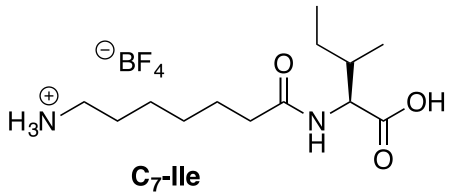

C_7-Ile

7-(((*1S,2S*)-1-carboxy-2-methylbutyl)amino)-7-oxoheptan-1-aminium tetrafluoroborate (C_7-Ile). Prepared from **14** (41.6 mg, 0.116 mmol) following the general procedure G. The product was obtained as a colorless solid (21.0 mg, 0.061 mmol, 52% yield). ^{1}H-NMR (400 MHz, CD_3CN) δ, ppm: 6.74 (d, *J* = 8.0 Hz, 1H), 6.34 (br-s, 3H), 4.30 (dd, *J* = 8.0 Hz, 5.8 Hz, 1H), 2.96 (t, *J* = 7.6 Hz, 2H), 2.24 (t, *J* = 7.3 Hz, 2H), 1.92 – 1.82 (m, 1H), 1.66 – 1.57 (m, 4H), 1.55 – 1.47 (m, 1H), 1.39 – 1.32 (m, 4H), 1.28 – 1.18 (m, 1H), 0.95 (d, *J* = 6.9 Hz, 3H), 0.93 (t, *J* = 7.4 Hz, 3H). ^{13}C-NMR (100 MHz, $CDCl_3$) δ, ppm: 173.8, 172.6, 56.8, 40.2, 36.5, 35.2, 27.8, 26.3, 25.3, 25.0, 24.9, 15.0, 10.8. HRMS (ESI-MS) *m/z* calculated for $C_{13}H_{26}N_2O_3$ $[M-H]^-$ 257.1871, found 257.1863.

4.5. Bz-Ile (5)

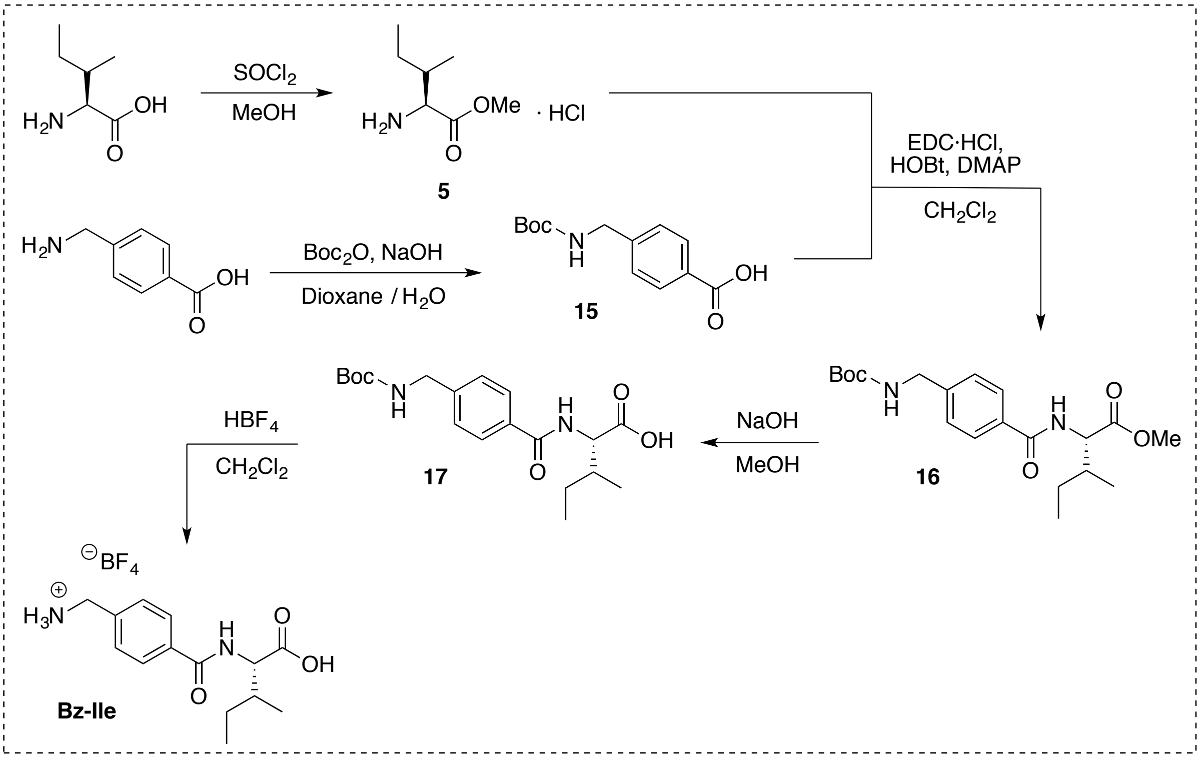

Scheme S6. Synthetic pathway for the synthesis of Bz-Ile (Figure 5, **5**).

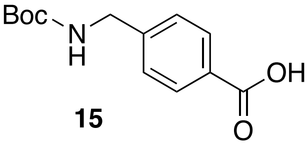

15

4-(((*tert*-butoxycarbonyl)amino)methyl)benzoic acid (15). Prepared from 4-(aminomethyl)benzoic acid (3.00 g, 19.8 mmol) following the general procedure B. The product was obtained as a colorless solid (4.97 g, 19.8 mmol, quantiative). Spectral data match those reported in literature.[22] ^{1}H-NMR (400 MHz, $CDCl_3$) δ, ppm: 8.09 (d, *J* = 8.3 Hz, 2H), 7.41 (d, *J* = 8.1 Hz, 2H), 4.98 (bs, 1H), 4.42 (d, *J* = 6.2 Hz), 1.49 (s, 9H).

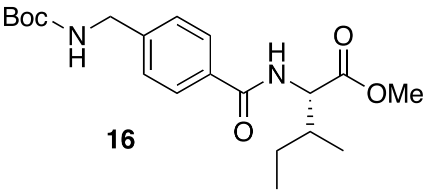

16

Methyl (4-(((*tert*-butoxycarbonyl)amino)methyl)benzoyl)-*L*-isoleucinate (16). Prepared from **5** (474.4 mg, 2.61 mmol) and **15** (656.2 mg, 2.61 mmol) following the general procedure D. Purification by flash chromatography over silica gel (hexane:EtOAc, 6:4) affords the product as a colorless solid (342.9 mg, 0.906 mmol, 35% yield). ^{1}H-NMR (400 MHz, $CDCl_3$) δ, ppm: 7.78 (d, *J* = 8.1 Hz, 2H), 7.37 (d, *J* = 8.1 Hz, 2H), 6.66 (d, *J* = 8.5 Hz, 1H), 4.96 (bs, 1H), 4.83 (dd, *J* = 8.5, 4.9 Hz, 1H), 4.38 (d, *J* = 6.1 Hz, 2H), 3.79 (s,

3H), 2.14 – 1.93 (m, 1H), 1.70 – 1.64 (m, 1H), 1.48 (s, 9H), 1.33 – 1.22 (m, 1H), 0.99 (d, *J* = 6.9 Hz, 3H), 0.98 (t, *J* = 7.4 Hz, 3H). ^{13}C-NMR (100 MHz, $CDCl_3$) δ, ppm: 172.6, 166.7, 155.9, 143.0, 133.1, 127.5, 127.4, 79.8, 56.8, 52.2, 44.3, 38.3, 28.4, 25.4, 15.5, 11.6. HRMS (ESI-MS) *m/z* calculated for $C_{20}H_{30}N_2O_5$ $[M+Na]^+$ 401.2047, found 401.2045.

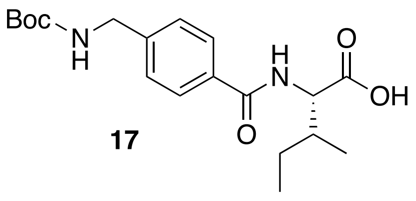

17

(4-(((*tert*-butoxycarbonyl)amino)methyl)benzoyl)-*L*-isoleucine (17). Prepared from **16** (103.7 mg, 0.303 mmol) following the general procedure E. The product was obtained as a colorless solid (35.6 mg, 0.098 mmol, 36% yield). ^{1}H-NMR (400 MHz, CD_3OD) δ, ppm: 7.82 (d, *J* = 8.1 Hz, 2H), 7.39 (d, *J* = 8.1 Hz, 2H), 4.56 (d, *J* = 6.3 Hz, 1H), 4.30 (s, 2H), 2.12 – 1.97 (m, 1H), 1.73 – 1.55 (m, 1H), 1.47 (s, 9H), 1.38 – 1.30 (m, 1H), 1.04 (d, *J* = 6.9 Hz, 3H), 0.99 (t, *J* = 7.4 Hz, 3H). ^{13}C-NMR (100 MHz, CD_3OD) δ, ppm: 174.0, 168.8, 157.2, 143.7, 132.7, 127.3, 126.7, 79.0, 57.7, 43.3, 36.9, 27.4, 25.2, 14.7, 10.3. HRMS (ESI-MS) *m/z* calculated for $C_{19}H_{28}N_2O_5$ $[M-H]^-$ 363.1925, found 363.1919.

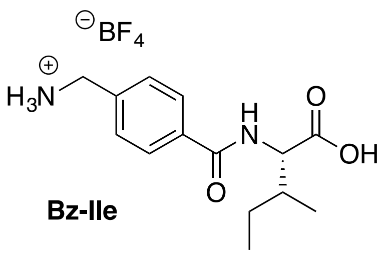

Bz-Ile

(4-(((*1S,2S*)-1-carboxy-2-methylbutyl)carbamoyl)phenyl) methanaminium tetrafluoroborate (Bz-Ile). Prepared from **17** (35.6 mg, 0.098 mmol) following the general procedure G. The product was obtained as a colorless solid (25.9 mg, 0.074 mmol, 75% yield). ^{1}H-NMR (400 MHz, CD_3CN) δ, ppm: 7.82 (d, *J* = 8.3 Hz, 2H), 7.51 (d, *J* = 8.3 Hz, 2H), 6.86 (br-s 3H), 4.51 (dd, *J* = 8.0, 6.1 Hz, 1H), 4.22 (s, 2H), 2.07 – 1.99 (m, 1H), 1.67 – 1.53 (m, 1H), 1.40 – 1.26 (m, 1H), 1.02 (d, *J* = 6.8 Hz, 3H), 0.97 (t, *J* = 7.4 Hz, 3H). ^{13}C-NMR (100 MHz, $CDCl_3$) δ, ppm: 172.3, 167.1, 135.6, 134.7, 129.3, 128.0, 57.4, 43.3, 36.6, 26.2, 15.0, 10.7. HRMS (ESI-MS) *m/z* calculated for $C_{14}H_{20}N_2O_3$ $[M-H]^-$ 263.1401, found 263.1404.

4.6. C_6-Pro (6)

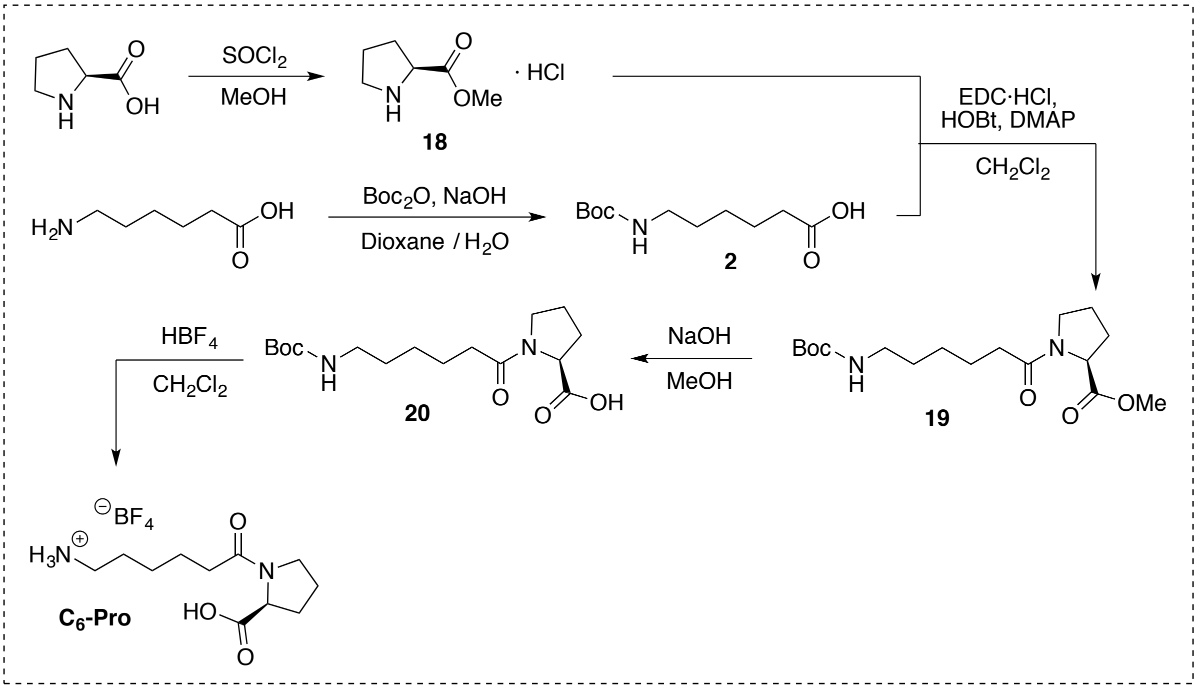

Scheme S7. Synthetic pathway for the synthesis of C_6-Pro (Figure 5, **6**).

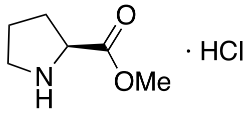

18

Methyl *L*-prolinate hydrochloride (18). Prepared from *L*-Proline (1.07 g, 9.11 mmol) following the general procedure A. The product was obtained as a clear oil (1.50 g, 9.06 mmol, 99% yield). Spectral data match those reported in literature.[23] ^{1}H-NMR (400 MHz, $CDCl_3$) δ, ppm: 4.46 (dd, J = 8.4, 6.7 Hz, 1H), 3.85 (s, 3H), 3.46 – 3.36 (m, 2H), 2.49 – 2.35 (m, 1H), 2.23 – 2.00 (m, 3H).

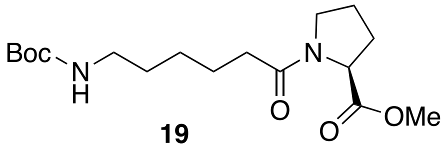

19

Methyl (6-((*tert*-butoxycarbonyl)amino)hexanoyl)-*L*-prolinate (19). Prepared from **2** (753.3 mg, 2.91 mmol) and **18** (529.4 mg, 2.91 mmol) following the general procedure D. Purification by flash chromatography over silica gel (hexane:EtOAc, 4:6) affords the product as a clear oil (399.8 mg, 1.17 mmol, 40% yield). The product is found as a mixture of cis- and trans- isomers.[24] ^{1}H-NMR (400 MHz, $CDCl_3$) δ, ppm: 4.66 (bs, 1H), 4.46 (dd, J = 8.4, 4.1 Hz, 1H), 3.71 (s, 3H), 3.67 – 3.56 (m, 1H), 3.56 – 3.43 (m, 1H), 3.09 (t, J = 6.6 Hz, 2H), 2.39 – 2.22 (m, 2H), 2.21 – 2.11 (m, 1H), 2.10 – 2.01 (m, 1H), 2.01 – 1.90 (m, 2H), 1.65 (quint, J = 8.0 Hz, 2H), 1.51 – 1.33 (m, 4H), 1.42 (s, 9H). ^{13}C-NMR (100 MHz, $CDCl_3$) δ, ppm: 173.0 (major), 172.8 (minor), 171.8 (minor), 171.7 (major), 156.0, 78.9, 59.3 (minor), 58.6 (major), 52.5 (minor), 52.1 (major), 47.0 (major), 46.3 (minor), 40.4, 34.2

(major), 34.1 (minor), 29.8, 29.2, 28.4, 26.4, 24.8 (major), 24.1 (minor), 22.5. HRMS (ESI-MS) *m/z* calculated for $C_{17}H_{30}N_2O_5$ $[M+Na]^+$ 365.2047, found 365.2050.

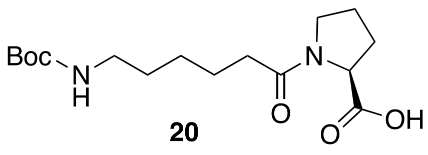

(6-((*tert*-butoxycarbonyl)amino)hexanoyl)-*L*-proline (20). Prepared from **19** (105.7 mg, 0.309 mmol) following the general procedure E. The product was obtained as a colorless solid (41.5 mg, 0.116 mmol, 41% yield). ^{1}H-NMR (400 MHz, CD_3OD) δ, ppm: 4.43 (dd, *J* = 8.7, 3.2 Hz, 1H), 3.70 – 3.51 (m, 2H), 3.05 (t, *J* = 6.9 Hz, 2H), 2.46 – 2.29 (m, 2H), 2.29 – 2.08 (m, 2H), 2.08 – 1.98 (m, 2H), 1.69 – 1.58 (m, 2H), 1.54 – 1.43 (m, 2H), 1.45 (s, 9H), 1.42 – 1.28 (m, 2H). ^{13}C-NMR (100 MHz, CD_3OD) δ, ppm: 174.4, 173.0, 157.1, 78.4, 58.8, 46.2, 39.8, 33.7, 29.3, 29.0, 27.4, 26.1, 24.3, 24.1. HRMS (ESI-MS) *m/z* calculated for $C_{16}H_{28}N_2O_5$ $[M-H]^-$ 327.1925, found 327.1916.

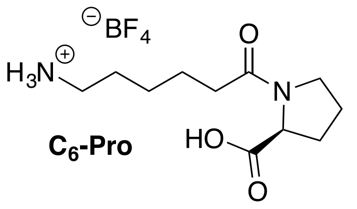

***(S)*-6-(2-carboxypyrrolidin-1-yl)-6-oxohexan-1-aminium tetrafluoroborate (C_6-Pro).** Prepared from **20** (39.5 mg, 0.123 mmol) following the general procedure G. The product was obtained as a colorless solid (37.3 mg, 0.118 mmol, 96% yield). ^{1}H-NMR (400 MHz, CD_3CN) δ, ppm: 6.40 (br-s, 3H), 4.42 (dd, *J* = 8.4, 4.0 Hz, 1H), 3.69 – 3.47 (m, 2H), 3.13 – 2.88 (m, 2H), 2.40 (td, *J* = 7.2, 2.1 Hz, 2H), 2.23 – 2.04 (m, 2H), 2.02 – 1.93 (m, 2H), 1.75 – 1.56 (m, 4H), 1.46 – 1.38 (m, 2H). ^{13}C-NMR (100 MHz, CD_3CN) δ, ppm: 137.7, 172.7, 59.2, 47.5, 40.0, 33.3, 28.4, 26.0, 25.0, 24.4, 23.2. HRMS (ESI-MS) *m/z* calculated for $C_{11}H_{20}N_2O_3$ $[M-H]^-$ 227.1401, found 227.1410.

4.7. C_6-Adam (7)

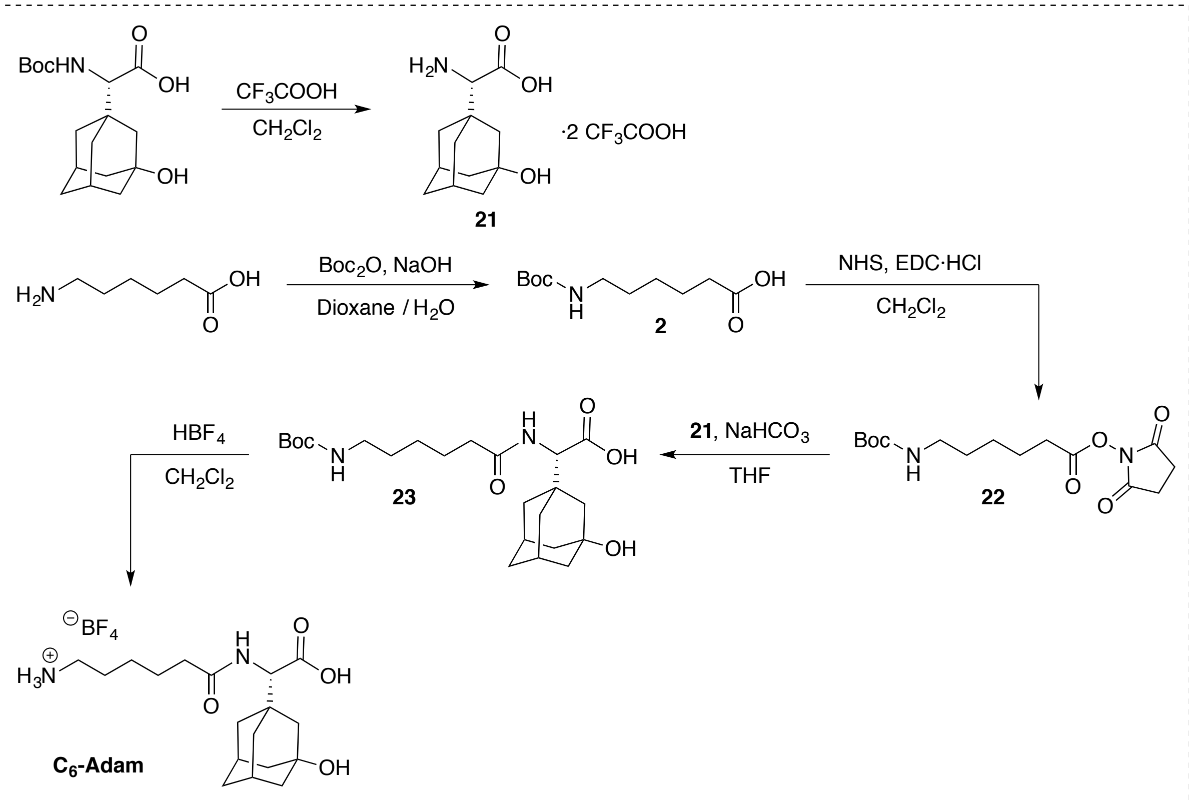

Scheme S8. Synthetic pathway for the synthesis of C_6-Adam (Figure 5, **7**).

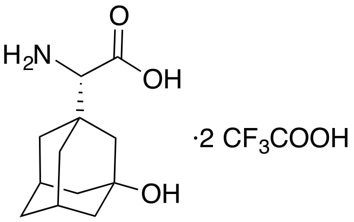

21

***(S)*-2-amino-2-((1*r*,3*R*,5*R*,7*S*)-3-hydroxyadamant-1-yl)acetic acid (27).** It was prepared according to a slightly modified reported procedure.[25] Boc-3-hydroxy-1-adamantyl-L-glycine (385.6 mg, 1.13 mmol) was dissolved in anhydrous CH_2Cl_2 (2.2 mL, 0.5M) under N_2 atmosphere. The mixture was cooled to 0°C and 22 equiv. of TFA (1.9 mL, 24.8 mmol) were added dropwise. The reaction was stirred at room temperature 3h, and after this time, the solvent was removed under reduced pressure. The residue was solved with EtOAc (10 mL) and the solvent was removed under reduced pressure. This washing-drying protocol was repeated three times. The product was obtained as a colorless solid (478.5 mg, 1.06 mmol, 94% yield) and used in the next step without further purification. ^{1}H-NMR (400 MHz, CD_3OD) δ, ppm: 3.60 (s, 1H), 2.28 (s, 2H), 1.78 – 1.53 (m, 12H). ^{13}C-NMR (100 MHz, CD_3OD) δ, ppm: 169.2, 67.4, 45.2, 43.4, 37.5, 36.9, 36.5, 34.5, 30.1. HRMS (ESI-MS) *m/z* calculated for $C_{12}H_{19}NO_3$ [M-H]$^-$ 224.1292, found 224.1289.

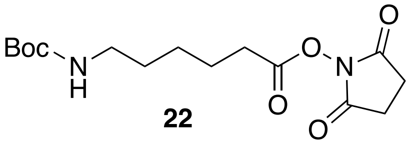

22

2,5-dioxopyrrolidin-1-yl-6-((*tert*-butoxycarbonyl)amino) hexanoate (22). Prepared from **2** (3.0 g, 13.0 mmol) following the general procedure C. Purification by flash chromatography over silica gel (hexane:EtOAc, 1:1) affords the product as a colorless solid (2.47 g, 7.52 mmol, 58% yield). Spectral data match those reported in literature.[21] ^{1}H-NMR (400 MHz, $CDCl_3$) δ, ppm: 4.60 (br-s, 1H), 3.14 (t, *J* = 6.8 Hz, 2H), 2.93 – 2.78 (m, 4H), 2.65 (t, *J* = 7.3 Hz, 2H), 1.79 (quint, *J* = 7.4 Hz, 2H), 1.60 – 1.44 (m, 4H), 1.46 (s, 9H).

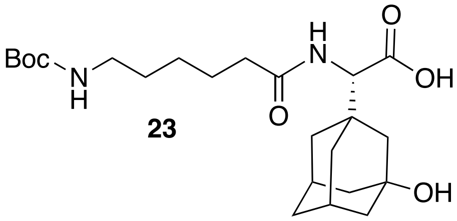

23

***(S)*-2-(6-((tert-butoxycarbonyl)amino)hexanamido-2-((1*r*,3*R,5R,7S)*-3-hydroxyadamant-1-yl)acetic acid (23).** Prepared from **22** (154.3 mg, 0.470 mmol) and **21** (212.1 mg, 0.470 mmol) following the general procedure F. The product was obtained as a colorless solid (46.8 mg, 0.106 mmol, 23% yield) with some small impurities. The product was taken on to subsequent synthetic steps during the course of which, the impurities were removed. ^{1}H-NMR (400 MHz, CD_3OD) δ, ppm: 4.27 (s, 1H), 3.04 (t, *J* = 7.0 Hz, 2H), 2.38 – 2.26 (m, 2H), 2.24 – 2.18 (m, 2H), 1.69 – 1.56 (m, 14H), 1.54 – 1.47 (m, 2H), 1.45 (s, 9H), 1.40 – 1.30 (m, 2H). ^{13}C-NMR (100 MHz, CD_3OD) δ, ppm: 174.8, 172.1, 157.2, 67.8, 60.6, 45.0, 43.8, 42.4, 39.8, 38.8, 37.3, 35.1, 35.0, 30.4, 30.3, 29.2, 27.4, 26.1, 25.3. HRMS (ESI-MS) *m/zz* calculated for $C_{23}H_{38}N_2O_6$ $[M-H]^-$ 437.2657, found 437.2659.

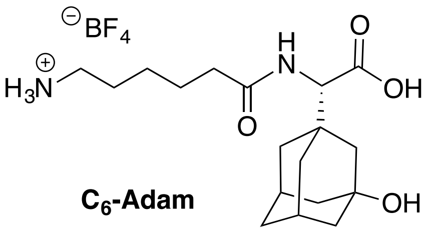

C$_6$-Adam

6-(((*S)*-carboxy((1*r*,3*R,5R,7S)*-3-hydroxyadamantan-1-yl)methyl)amino-6-oxohexan-1-aminium tetrafluoroborate (C$_6$-Adam). Prepared from **26** (18.2 mg, 0.042 mmol) following the general procedure G. The product was obtained as a colorless solid (11.9 mg, 0.028 mmol, 67% yield). ^{1}H-NMR (400 MHz, CD_3CN) δ, ppm: 6.66 (br-s, 1H), 6.29 (br-s, 3H), 4.16 (d, *J* = 8.8 Hz, 1H), 3.05 – 2.96 (m, 2H), 2.34 – 2.25 (m, 4H), 1.73 – 1.47 (m, 15H), 1.46 – 1.33 (m, 3H). ^{13}C-NMR (100 MHz, CD_3CN) δ, ppm: 173.2, 171.2, 67.6, 60.5, 46.1, 44.1, 44.1, 40.0, 38.6, 37.4, 37.1, 34.9, 34.8, 30.4, 30.3, 26.0, 25.1, 24.1. HRMS (ESI-MS) *m/z* calculated for $C_{18}H_{30}N_2O_4$ $[M-H]^-$ 337.2133, found 337.2125.

4.8. C_6-Hyp (8)

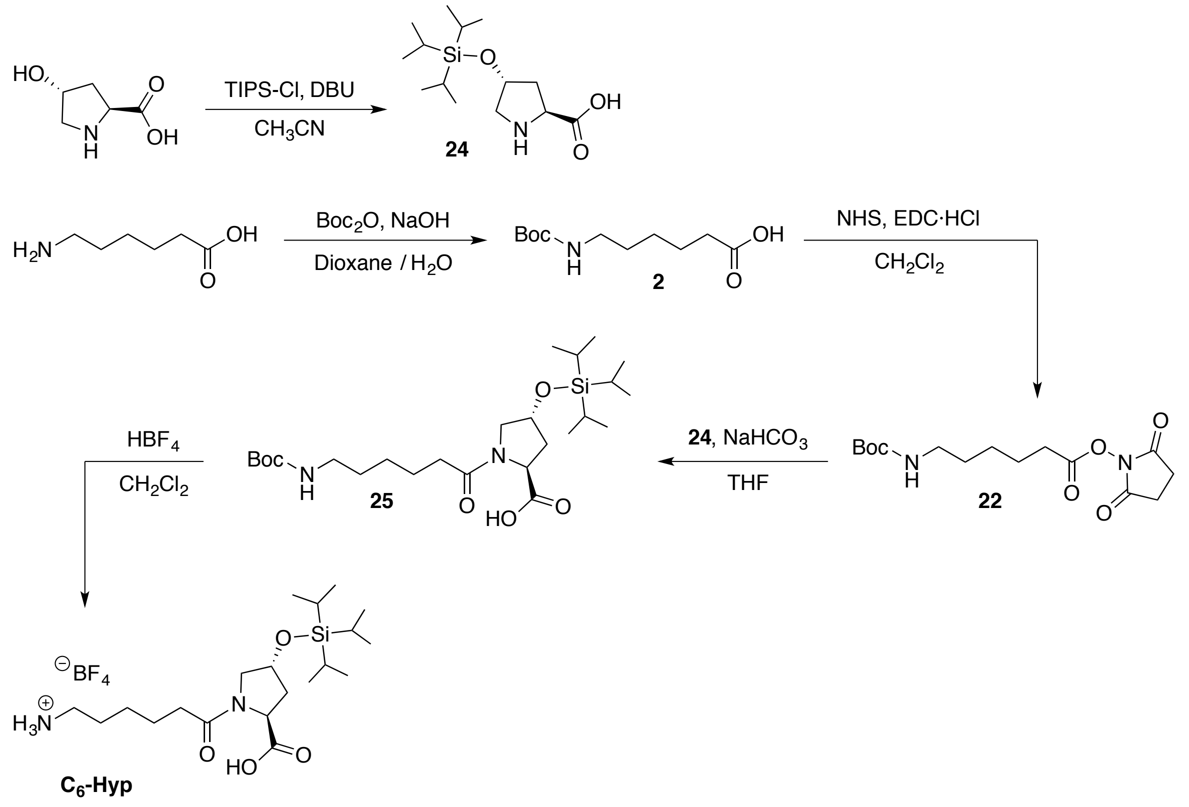

Scheme S9. Synthetic pathway for the synthesis of C_6-Hyp (Figure 5, **8**).

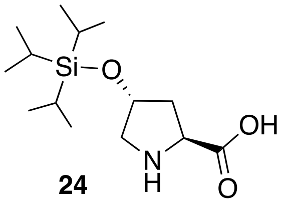

24

***(2S,4R)*-4-((triisopropylsilyl)oxy)pyrrolidine-2-carboxylic acid (24).** It was prepared according to slightly modified reported procedures.[26, 27] L-hydroxyproline (563.0 mg, 4.25 mmol, 1 equiv.) was suspended in CH_3CN (7 mL, 0.6M) and 3.5 equiv. of TIPS-Cl (3.3 mL, 14.9 mmol) were added. The mixture was cooled to 0°C and then 3.7 equiv. of DBU (2.4 mL, 15.7 mmol) were added dropwise. The reaction was stirred overnight at room temperature. After this time, the reaction was quenched with hexane (10 mL) and the phases were separated. The product was extracted with hexane (3 x 15 mL) and then, the solvent was removed under reduced pressure. Purification by flash chromatography over silica gel (CH_2Cl_2:MeOH:NH_3, 10:1:0.1) affords the product as a colorless solid (428.5 mg, 1.49 mmol, 35% yield). Spectral data match those reported in literature.[28] ^{1}H-NMR (400 MHz, $CDCl_3$) δ, ppm: 4.57 (quint, *J* = 4.6 Hz, 1H), 4.20 (t, *J* = 7.5 Hz, 1H), 3.52 (dd, *J* = 11.4, 5.1 Hz, 1H), 3.25 (dd, *J* = 11.4, 4.1 Hz, 1H), 2.32 (ddd, *J* = 12.8, 7.5, 4.1 Hz, 1H), 2.18 (ddd, *J* = 12.8, 7.5, 5.1 Hz, 1H), 1.28 – 0.88 (m, 21H).

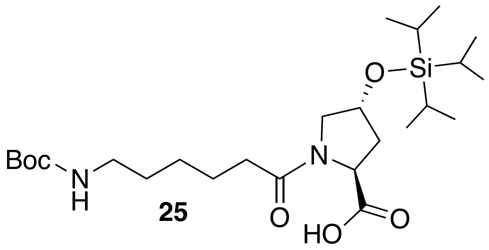

25

***(2S,4R)*-1-(6-((*tert*-butoxycarbonyl)amino)hexanoyl)-4-((triisopropylsilyl)oxy)pyrrolidine-2-carboxylic acid (25).** Prepared from **24** (124.6 mg, 0.433 mmol) and **22** (142.3 mg, 0.433 mmol) following the general procedure F. The product was obtained as a colorless solid (135.7 mg, 0.271 mmol, 63% yield) with some small impurities. The product was taken on to subsequent synthetic steps during the course of which, the impurities were removed. ^{1}H-NMR (400 MHz, CD_3OD) δ, ppm: 4.72 – 4.60 (m, 1H), 4.49 (t, *J* = 7.9 Hz, 1H), 3.80 (dd, *J* = 10.7, 4.2 Hz, 1H), 3.60 – 3.48 (m, 1H), 3.04 (td, *J* = 6.9, 4.5 Hz, 2H), 2.37 (t, *J* = 7.4 Hz, 2H), 2.34 – 2.25 (m, 1H), 2.17 – 2.06 (m, 1H), 1.65 (quint, *J* = 7.4 Hz, 2H), 1.52 – 1.36 (m, 4H), 1.45 (s, 9H), 1.10 (m, 21H). ^{13}C-NMR (100 MHz, CD_3OD) δ, ppm: 174.4, 173.2, 157.1, 78.4, 71.1, 57.7, 55.7, 39.8, 38.3, 33.8, 29.3, 27.5, 26.0, 24.3, 17.1, 11.9. HRMS (ESI-MS) *m/z* calculated for $C_{25}H_{48}N_2O_6Si$ [M-H]$^-$ 499.3209, found 499.3199.

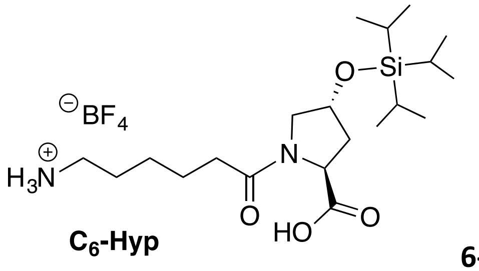

C_6-Hyp

6-*(2S,4R)*-2-carboxy-4-((triisopropylsilyl)oxy)pyrrolidin-1-yl)-6-oxohexan-1-aminium tetrafluoroborate (C_6-Hyp). Prepared from **25** (45.2 mg, 0.090 mmol) following the general procedure G. The product was obtained as a colorless solid (31.3 mg, 0.064 mmol, 71% yield). ^{1}H-NMR (400 MHz, CD_3CN) δ, ppm: 6.34 (br-s, 3H), 4.64 (q, *J* = 4.2 Hz, 1H), 4.48 (t, *J* = 7.6 Hz, 1H), 3.74 (dd, *J* = 10.8, 4.5 Hz, 1H), 3.48 (dd, *J* = 10.8, 3.2 Hz, 1H), 2.00 (q, *J* = 6.7 Hz, 2H), 2.37 (t, *J* = 7.1 Hz, 2H), 2.28 – 2.10 (m, 2H), 1.73 – 1.54 (m, 4H), 1.56 – 1.38 (m, 2H), 1.17 – 0.98 (m, 21H). ^{13}C-NMR (100 MHz, CD_3CN) δ, ppm: 173.6, 172.5, 69.3, 57.8, 55.0, 39.9, 36.8, 33.4, 25.9, 24.9, 23.0, 17.3, 11.8. HRMS (ESI-MS) *m/z* calculated for $C_{20}H_{40}N_2O_4Si$ [M-H]$^-$ 399.2685, found 399.2681.

4.9. C_6-cBut (9)

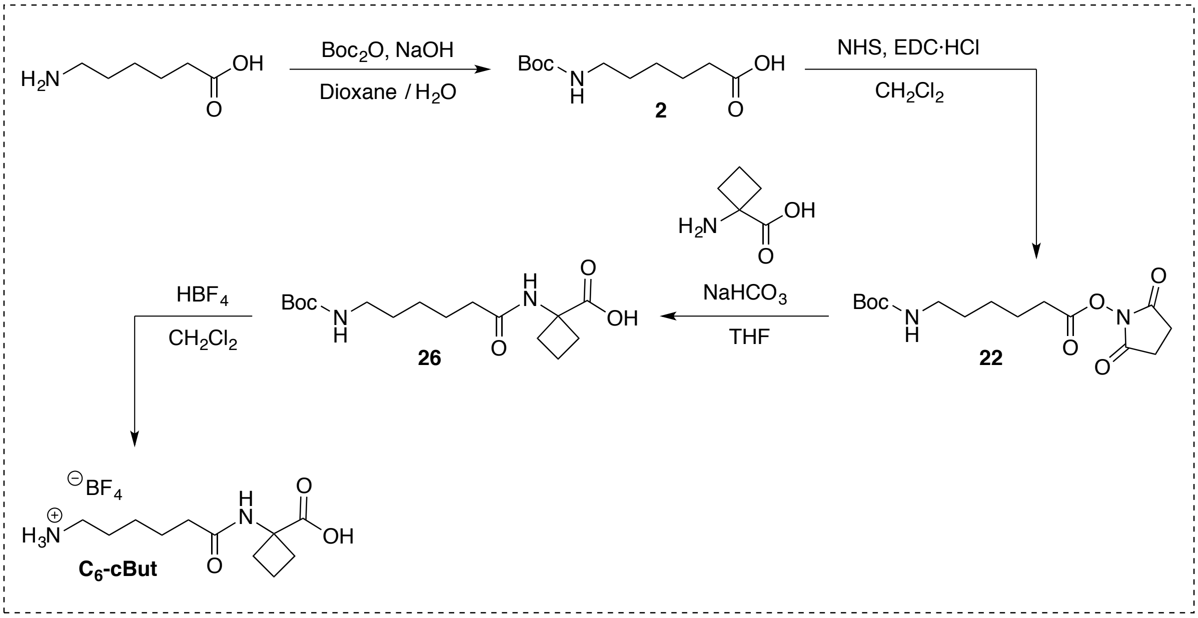

Scheme S10. Synthetic pathway for the synthesis of C_6-cBut (Figure 5, **9**).

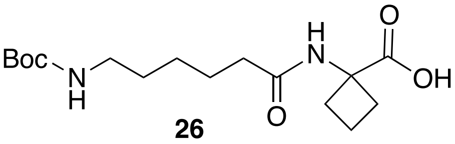

1-(6-((*tert*-butoxycarbonyl)amino)hexanamido) cyclobutane-1-carboxylic acid (26). Prepared from **22** (611.1 mg, 1.86 mmol) and 1-aminocyclobutane-1-carboxylic acid (220.9 mg, 1.86 mmol) following the general procedure F. The product was obtained as a colorless solid (251.4 mg, 0.766 mmol, 41% yield). ^{1}H-NMR (400 MHz, CD_3OD) δ, ppm: 3.04 (t, *J* = 7.0 Hz, 2H), 2.69 – 2.60 (m, 2H), 2.29 – 2.15 (m, 4H), 2.10 – 1.95 (m, 2H), 1.64 (quint, *J* = 7.4 Hz, 2H), 1.55 – 1.47 (m, 2H), 1.45 (s, 9H), 1.38 (m, 2H). ^{13}C-NMR (100 MHz, CD_3OD) δ, ppm: 175.5, 174.4, 157.1, 78.4, 58.0, 39.8, 35.0, 30.9, 29.2, 27.4, 25.9, 25.1, 15.0. HRMS (ESI-MS) *m/z* calculated for $C_{16}H_{28}N_2O_5$ $[M+Na]^+$ 351.1890, found 351.1900.

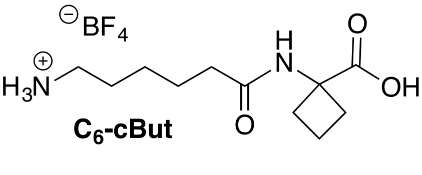

6-((1-carboxycyclobutyl)amino)-6-oxohexan-1-aminium tetrafluoroborate (C_6-cBut). Prepared from **26** (46.9 mg, 0.143 mmol) following the general procedure G. The product was obtained as a colorless solid (31.1 mg, 0.098 mmol, 69% yield). ^{1}H-NMR (400 MHz, CD_3CN) δ, ppm: 7.37 (bs, 1H), 6.36 (bs, 3H), 3.05 – 2.95 (m, 2H), 2.67 – 2.57 (m, 2H), 2.28 – 2.14 (m, 4H), 2.03 – 1.97 (m, 1H), 1.72 – 1.55 (m, 4H), 1.48 – 1.34 (m, 3H). ^{13}C-NMR (100 MHz, CD_3CN) δ, ppm: 174.5, 173.9, 58.4, 40.1, 34.6, 30.9, 26.1, 25.0, 24.2, 15.1. HRMS (ESI-MS) *m/z* calculated for $C_{11}H_{21}N_2O_3$ M^+ 229.1553, found 229.1547.

4.10. C_6-cLeu (10)

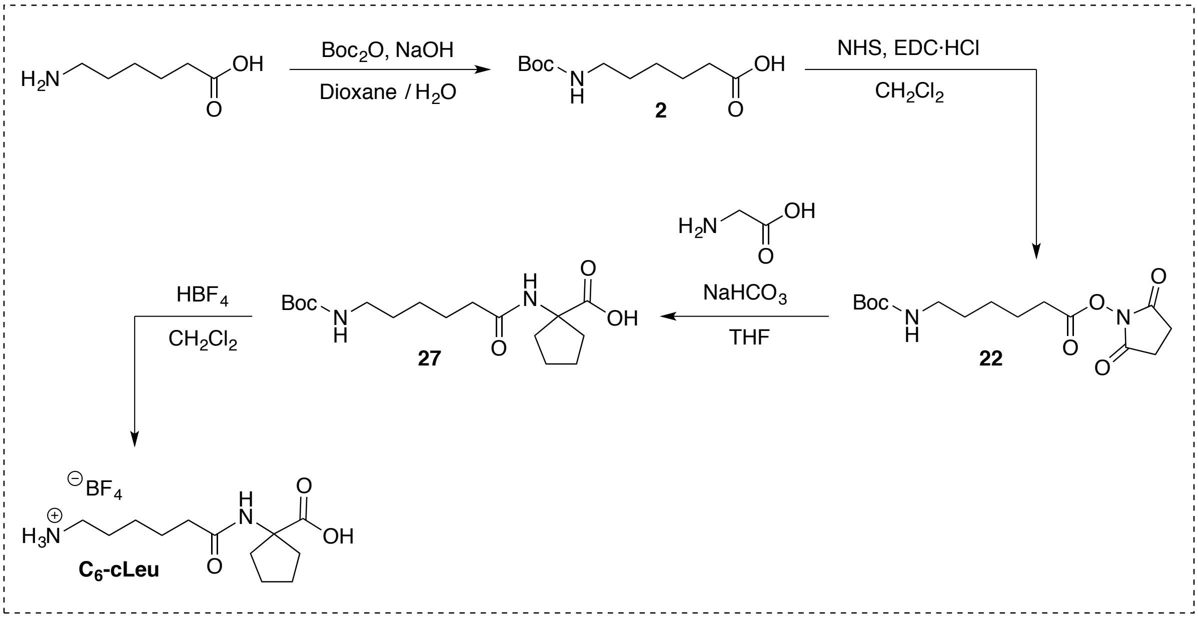

Scheme S11. Synthetic pathway for the synthesis of C_6-cBut (Figure 5, **10**).

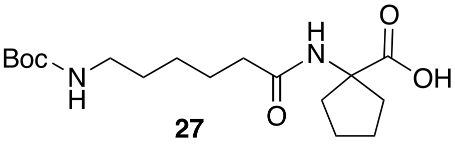

1-(6-((*tert*-butoxycarbonyl)amino)hexanamido)cyclo-pentane-1-carboxylic acid (27). Prepared from **22** (553.7 mg, 1.69 mmol) and 1-aminocyclopentane-1-carboxylic acid (220.0 mg, 1.69 mmol) following the general procedure F. The product was obtained as a colorless solid (89.0 mg, 0.250 mmol, 15% yield). ^{1}H-NMR (400 MHz, CD_3OD) δ, ppm: 3.04 (t, *J* = 7.0 Hz, 2H), 2.28 – 2.15 (m, 4H), 2.00 – 1.92 (m, 3H), 1.79 – 1.75 (m, 4H), 1.68 – 1.58 (m, 2H), 1.51 – 1.40 (m, 1H), 1.45 (s, 9H), 1.39 – 1.31 (m, 2H). ^{13}C-NMR (100 MHz, CD_3OD) δ, ppm: 176.8, 174.5, 157.1, 78.4, 65.7, 39.8, 36.6, 35.2, 29.3, 27.4, 26.0, 25.2, 24.1. HRMS (ESI-MS) *m/z* calculated for $C_{17}H_{30}N_2O_5$ $[M+Na]^+$ 365.2047, found 365.2049.

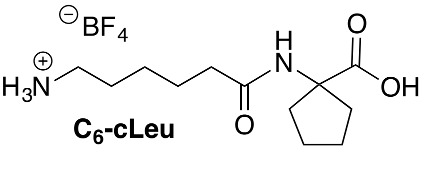

6-(1-carboxycyclopentyl)amino)-6-oxohexan-1-aminium tetrafluoroborate (C_6-cLeu). Prepared from **27** (80.9 mg, 0.236 mmol) following the general procedure G. The product was obtained as a colorless solid (76.5 mg, 0.231 mmol, 98% yield). ^{1}H-NMR (400 MHz, CD_3CN) δ, ppm: 6.91 (bs, 1H), 6.29 (bs, 3H), 3.00 – 2.95 (m, 2H), 2.19 (t, *J* = 6.8 Hz, 2H), 1.94 – 1.90 (m, 4H), 1.76 – 1.72 (m, 4H), 1.68 – 1.59 (m, 4H), 1.42 – 1.33 (m, 2H). ^{13}C-NMR (100 MHz, CD_3CN) δ, ppm: 1174.5, 174.1, 66.0, 40.0, 36.4, 34.7, 26.0, 24.9, 24.0, 23.8. HRMS (ESI-MS) *m/z* calculated for $C_{13}H_{25}$ N_2O_3 M^+ 243.1703, found 243.1703.

4.11. C_6-HcLeu (11)

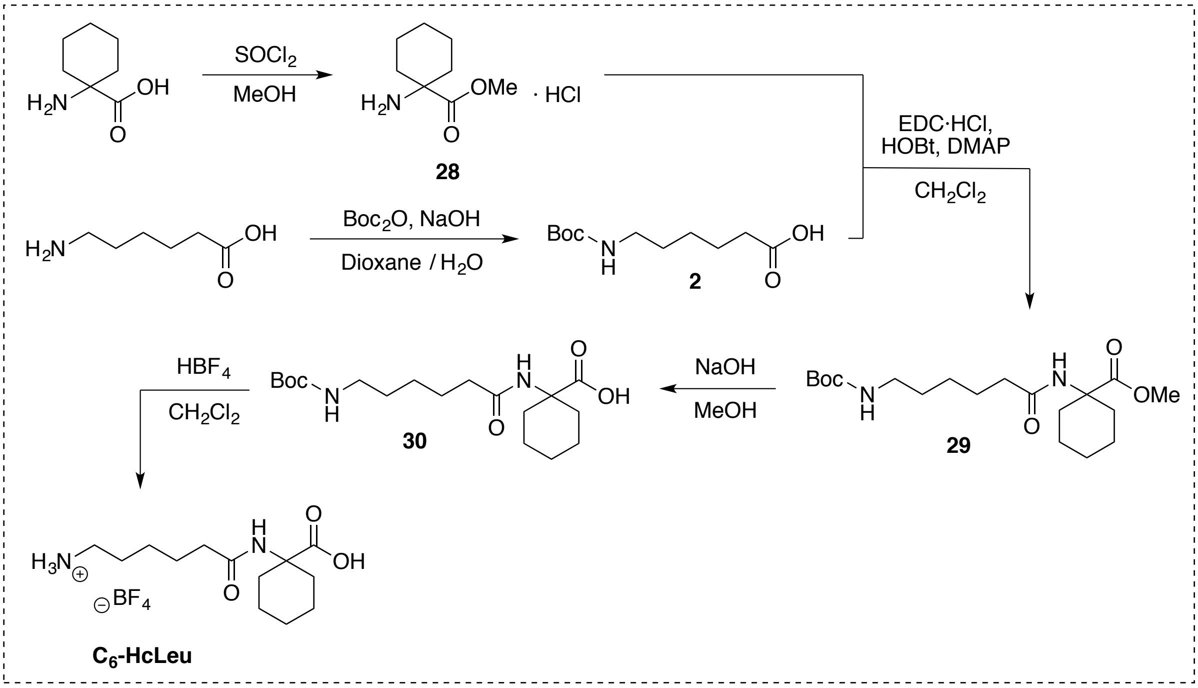

Scheme S12. Synthetic pathway for the synthesis of C_6-HcLeu (Figure 5, **11**).

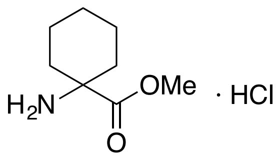

28

Methyl 1-aminocyclohexane-1-carboxylate hydrochloride (28). Prepared from 1-aminocyclohexane-1-carboxylic acid (1.40 g, 9.58 mmol) following the general procedure A. The product was obtained as a colorless solid (1.80 g, 9.29 mmol, 97% yield). Spectral data match those reported in literature.[29] ^{1}H-NMR (300 MHz, CD_3OD) δ, ppm: 3.87 (s, 3H), 2.20 – 2.05 (m, 2H), 1.86 – 1.57 (m, 8H).

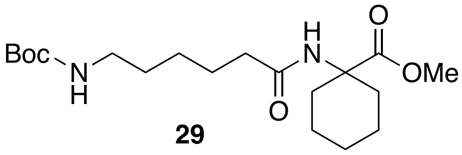

29

Methyl 1-(6-((*tert*-butoxycarbonyl)amino) hexanamido)cyclohexane-1-carboxylate (29). Prepared from **2** (850.1 mg, 3.28 mmol) and **28** (637.0 mg, 3.28 mmol) following the general procedure D. Purification by flash chromatography over silica gel (hexane:EtOAc, 6:4) affords the product as a colorless solid (494.3 mg, 1.33 mmol, 41% yield). ^{1}H-NMR (400 MHz, $CDCl_3$) δ, ppm: 5.66 (bs, 1H), 4.63 (bs, 1H), 3.71 (s, 3H), 3.13 (q, J = 6.7 Hz, 2H), 2.22 (t, J = 7.4 Hz, 2H), 2.13 – 1.98 (m, 2H), 1.94 – 1.79 (m, 2H), 1.76 – 1.57 (m, 5H), 1.55 – 1.47 (m, 2H), 1.45 (s, 9H), 1.42 – 1.27 (m, 5H). ^{13}C-NMR (100 MHz, $CDCl_3$) δ,

ppm: 174.6, 172.4, 156.0, 79.0, 58.7, 52.3, 40.3, 36.3, 32.4, 29.7, 28.4, 26.2, 25.2, 25.1, 21.5. HRMS (ESI-MS) *m/z* calculated for $C_{19}H_{34}N_2O_5$ $[M+Na]^+$ 393.2360, found 393.2348.

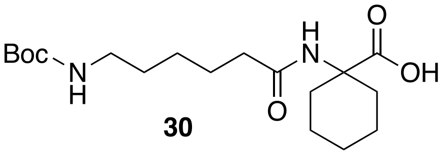

30

1-(6-((*tert*-butoxycarbonyl)amino)hexanamido)cyclo-hexane-1-carboxylic acid (20). Prepared from **19** (66.0 mg, 0.178 mmol) following the general procedure E. The product was obtained as a colorless solid (24.8 mg, 0.070 mmol, 39% yield). ^{1}H-NMR (400 MHz, CD_3OD) δ, ppm: 3.04 (t, *J* = 7.0 Hz, 2H), 2.25 (t, *J* = 7.4 Hz, 2H), 2.06 (dd, *J* = 13.5, 4.5 Hz, 2H), 1.83 (td, *J*= 13.6, 12.6, 3.4 Hz, 2H), 1.71 – 1.59 (m, 5H), 1.57 – 1.48 (m, 4H), 1.45 (s, 9H), 1.40 – 1.23 (m, 3H). ^{13}C-NMR (100 MHz, CD_3OD) δ, ppm: 177.2, 174.4, 157.1, 78.4, 58.8, 39.9, 35.3, 32.0, 29.3, 27.4, 26.0, 25.3, 25.2, 21.3. HRMS (ESI-MS) *m/z* calculated for $C_{18}H_{32}N_2O_5$ $[M-H]^-$ 355.2238, found 355.2243.

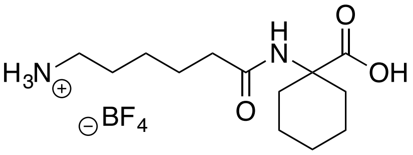

C_6-HcLeu

6-(1-carboxycyclohexyl)amino)-6-oxohexan-1-aminium tetrafluoroborate (C_6-HcLeu). Prepared from **20** (24.8 mg, 0.070 mmol) following the general procedure G. The product was obtained as a colorless solid (19.8 mg, 0.058 mmol, 83% yield). ^{1}H-NMR (400 MHz, CD_3CN) δ, ppm: 7.07 (br-s, 1H), 6.53 – 6.32 (br-s, 3H), 3.04 – 2.03 (m, 2H), 2.31 (t, *J* = 7.3 Hz, 2H), 2.08 – 1.99 (m, 2H), 1.87 – 1.75 (m, 2H), 1.71 – 1.56 (m, 7H), 1.56 – 1.46 (m, 2H), 1.44 – 1.29 (m, 3H). ^{13}C-NMR (100 MHz, CD_3CN) δ, ppm: 175.2, 174.7, 59.3, 40.0, 34.7, 31.7, 26.0, 25.0, 24.8, 24.6, 21.0. HRMS (ESI-MS) *m/z* calculated for $C_{13}H_{25}N_2O_3$ $[M-H]^-$ 255.1714, found 255.1715.

4.12. C_6-cHept (12)

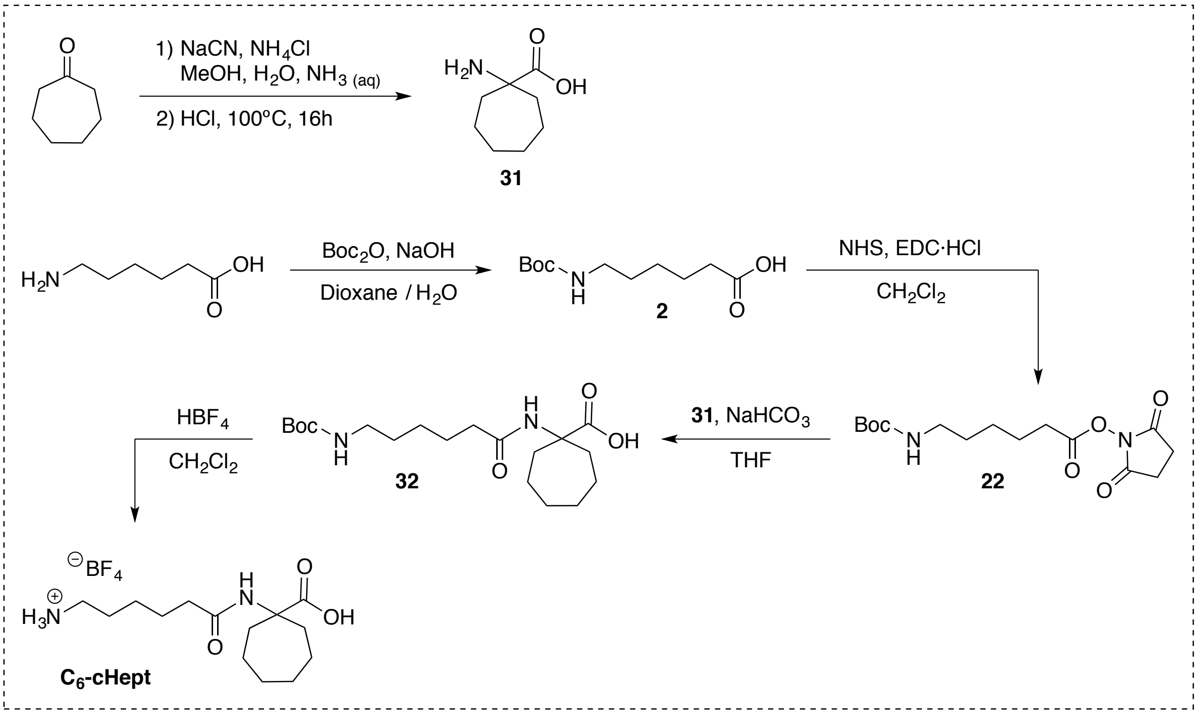

Scheme S13. Synthetic pathway for the synthesis of C_6-cHept (Figure 5, **12**).

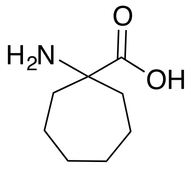

31

1-aminocycloheptane-1-carboxylic acid (31). It was prepared according to a reported procedure on a 21 mmol scale.[9] The product was obtained as a colorless solid (2.37 g, 15.1 mmol, 72% yield). Spectral data match those reported in literature.[9] ^{1}H-NMR (400 MHz, D_2O) δ, ppm: 2.18-2.03 (m, 2H), 1.80 – 1.62 (m, 4H), 1.55 – 1.49 (m, 6H).

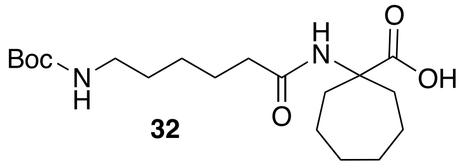

32

1-(6-((*tert*-butoxycarbonyl)amino)hexanamido)cycloheptane-1-carboxylic acid (32). Prepared from **22** (635.0 mg, 1.93 mmol) and **31** (304.0 mg, 1.93 mmol) following the general procedure F. The product was obtained as a colorless solid (90.0 mg, 0.242 mmol, 13% yield). ^{1}H-NMR (400 MHz, CD_3OD) δ, ppm: 3.08 – 3.00 (m, 2H), 2.24 – 2.15 (m, 4H), 2.04 – 1.99 (m, 2H), 1.63 – 1.57 (m, 12H), 1.54 – 1.42 (m, 1H), 1.45 (s, 9H), 1.39 – 1.33 (m, 1H). ^{13}C-NMR (100 MHz, CD_3OD) δ, ppm: 175.5, 174.1, 157.1, 78.4, 51.2, 39.8, 35.5, 29.6, 27.4, 25.9, 25.2, 22.4, 22.3, 21.1. HRMS (ESI-MS) *m/z* calculated for $C_{19}H_{34}N_2O_5$ $[M-H]^-$ 369.4825, found 369.4820.

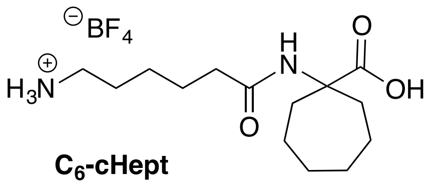

C$_6$-cHept

6-(1-carboxycyclopentyl)amino)-6-oxoheptan-1-aminium tetrafluoroborate (C$_6$-cLeu). Prepared from **33** (18.3 mg, 0.049 mmol) following the general procedure G. The product was obtained as a colorless solid (14.0 mg, 0.039 mmol, 79% yield). ^{1}H-NMR (400 MHz, CD_3CN) δ, ppm: 6.78 (bs, 1H), 6.23 (bs, 3H), 3.01 – 2.95 (m, 2H), 2.32 (t, J = 7.3 Hz, 2H), 2.21 (t, J = 6.9 Hz, 2H), 2.15 – 2.02 (m, 2H), 1.69 – 1.56 (m, 12H), 1.40 – 1.36 (m, 2H). ^{13}C-NMR (100 MHz, CD_3CN) δ, ppm: 175.3, 173.6, 62.3, 40.0, 35.7, 35.5, 34.8, 29.4, 26.0, 25.0, 22.4. HRMS (ESI-MS) m/z calculated for $C_{14}H_{27}N_2O_3$ M^+ 271.2016, found 271.2016.

4.13. C$_6$-cOct (13)

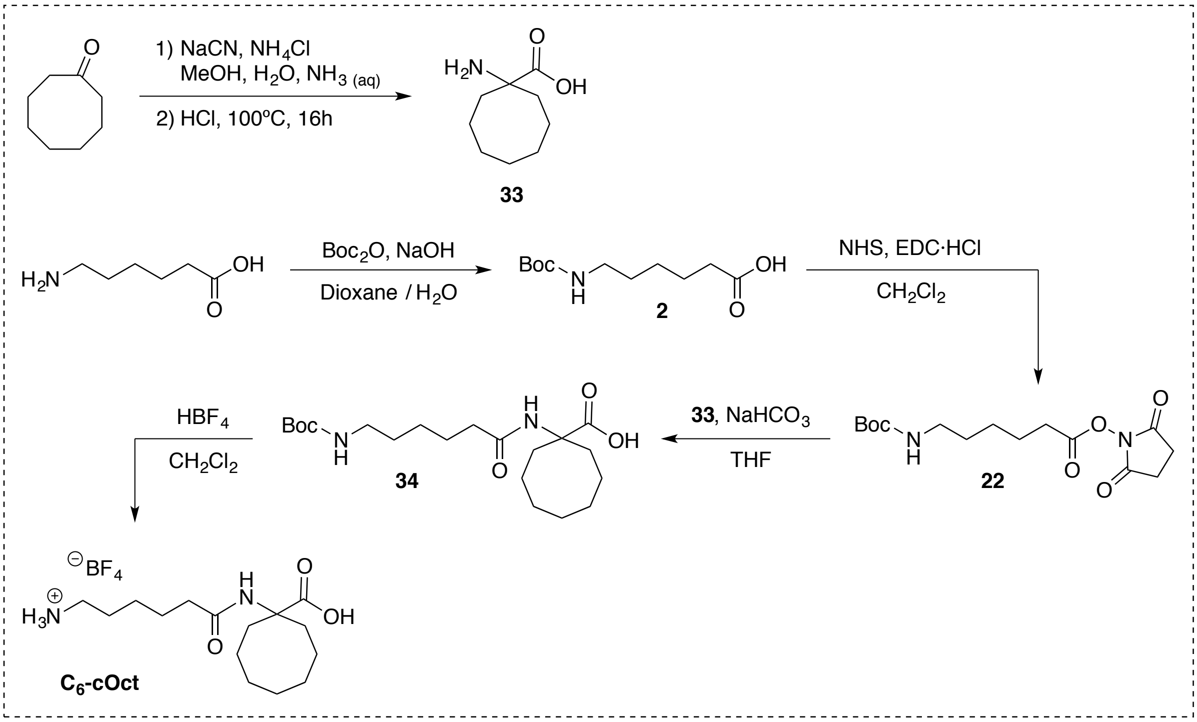

Scheme S14. Synthetic pathway for the synthesis of C$_6$-cOct (Figure 5, **13**).

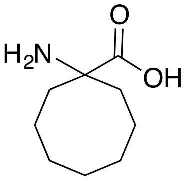

33

1-aminocyclooctane-1-carboxylic acid (33). It was prepared according to a reported procedure on a 19.6 mmol scale.[9] The product was obtained as a colorless solid (1.47 g, 8.58 mmol, 44% yield).[9] Spectral data match those reported in literature. ^{1}H-NMR (400 MHz, D_2O) δ, ppm: 2.15 – 2.04 (m, 2H), 1.81 – 1.75 (m, 2H), 1.65 – 1.56 (m, 2H), 1.51 – 1.43 (m, 8H).

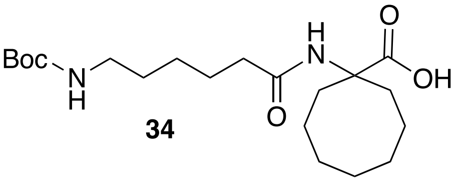

34

1-(6-((*tert*-butoxycarbonyl)amino)hexanamido)cyclooctane-1-carboxylic acid (34). Prepared from **22** (635.0 mg, 1.93 mmol) and **33** (304.0 mg, 1.93 mmol) following the general procedure F. The product was obtained as a colorless solid (90.0 mg, 0.242 mmol, 13% yield) and although not completely pure, it was used in the next step. ^{1}H-NMR (400 MHz, CD_3OD) δ, ppm: 3.03 (t, *J* = 7.0 Hz, 2H), 2.22 – 2.17 (m, 2H), 2.06 – 1.98 (m, 2H), 1.66 – 1.52 (m, 10H), 1.51 – 1.42 (m, 2H), 1.45 (s, 9H), 1.38 – 1.31 (m, 6H). ^{13}C-NMR (100 MHz, CD_3OD) δ, ppm: 173.6, 171.6, 157.1, 60.2, 39.8, 35.2, 29.8, 27.9, 27.4, 24.8, 21.6, 21.2, 19.5, 17.0, 13.1. HRMS (ESI-MS) *m/z* calculated for $C_{20}H_{36}N_2O_5$ $[M+Na]^+$ 407.2516, found 407.2524.

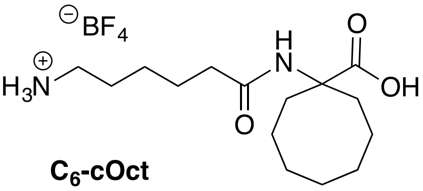

C_6-cOct

6-(1-carboxycyclopentyl)amino)-6-oxooctan-1-aminium tetrafluoroborate (C_6-cLeu). Prepared from **33** (18.3 mg, 0.049 mmol) following the general procedure G. The product was obtained as a colorless solid (14.0 mg, 0.039 mmol, 79% yield). ^{1}H-NMR (400 MHz, CD_3CN) δ, ppm: 6.65 (bs, 1H), 3.04 – 2.95 (m, 2H), 2.21 – 2.13 (m, 2H), 2.06 – 2.01 (m, 4H), 1.67 – 1.47 (m, 14H), 1.39 – 1.28 (m, 2H). ^{13}C-NMR (100 MHz, CD_3CN) δ, ppm: 176.3, 174.5, 62.2, 40.0, 34.8, 29.9, 27.8, 27.6, 26.0, 24.7, 24.0, 21.5. HRMS (ESI-MS) *m/z* calculated for $C_{15}H_{29}N_2O_3$ M^+ 285.2173, found 285.2179.

4.14. Piv-Lys(Gly) (14)

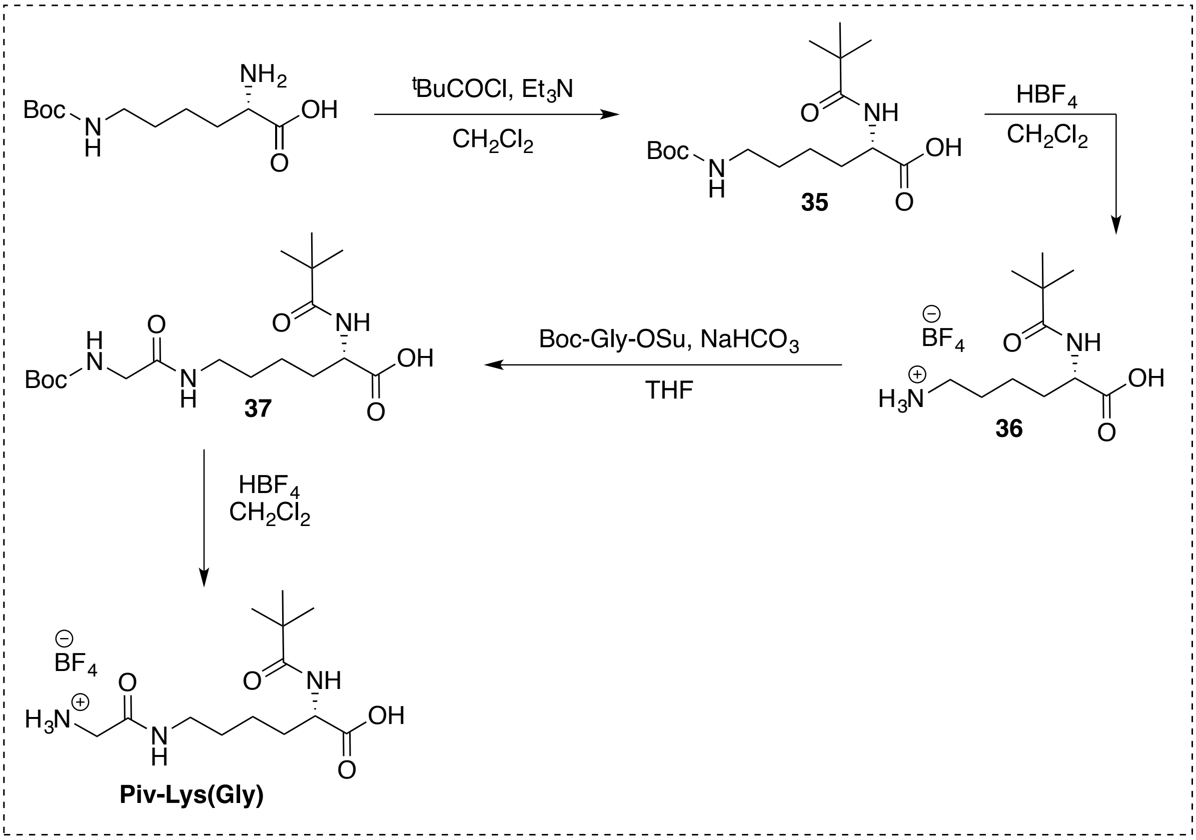

Scheme S15. Synthetic pathway for the synthesis of Piv-Lys(Gly) (Figure 5, **14**).

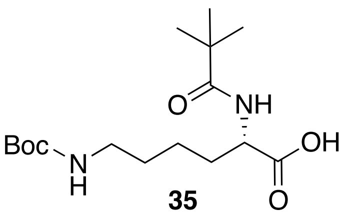

***N*⁶-(*tert*-butoxycarbonyl)-*N*²-pivaloyl-*L*-lysine (35).** H-Lys(Boc)-OH (886.0 mg, 3.60 mmol, 1equiv.) was dissolved in a mixture of methanol (4 mL) and 1M NaOH (20 mL). The reaction was cooled to 0°C and 1.1 equiv. of pivaloyl chloride were added dropwise (490 μL, 3.98 mmol). The mixture was stirred at room temperature overnight. Then, the mixture was diluted with 25 mL of EtOAc and the phases were separated. The aqueous layer was acidified with 1M HCl until pH = 2 and the product was extracted wih EtOAc (3 x 25 mL). The combined organic layers were dried over anhydrous $MgSO_4$, filtered and the solvent was evaporated under reduced pressure. The product was obtained as a clear oil (1.03 g, 3.3 mmol, 59% yield) and was used in the next step without further purification. ^{1}H-NMR (400 MHz, CD_3OD) δ, ppm: 4.39 (dd, *J* = 9.3, 4.9 Hz, 1H), 3.05 (t, *J* = 6.8 Hz, 2H), 1.98 – 1.87 (m, 1H), 1.82 – 1.71 (m, 1H), 1.54 – 1.38 (m, 4H), 1.45 (s, 9H), 1.23 (s, 9H). ^{13}C-NMR (100 MHz, CD_3OD) δ, ppm: 179.9, 174.4, 157.1, 78.4, 52.3, 39.7, 38.3, 30.7, 29.1, 27.5, 26.5, 22.9. HRMS (ESI-MS) *m/z* calculated for $C_{16}H_{30}N_2O_5$ $[M-H]^-$ 329.2082, found 329.2076.

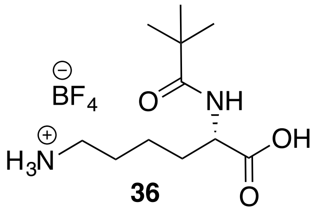

36

***(S)*-5-carboxy-5-pivalamidopentan-1-amiinum tetrafluoroborate (36).** Prepared from **35** (500.0 mg, 1.51 mmol) following the general procedure G. Crystallization with CH_2Cl_2/hexane affords the product as a colorless solid (440.0 mg, 1.38 mmol, 91% yield). ^{1}H-NMR (400 MHz, CD_3CN) δ, ppm: 6.68 (d, *J* = 7.5 Hz, 1H), 6.28 (br-s, 3H), 4.34 (ddd, *J* = 9.1, 7.5, 5.0 Hz, 1H), 3.02 – 2.91 (m, 2H), 1.94 – 1.82 (m, 1H), 1.82 – 1.73 (m, 1H), 1.73 – 1.62 (m, 2H), 1.49 – 1.37 (m, 2H), 1.19 (s, 9H). ^{13}C-NMR (100 MHz, CD_3CN) δ, ppm: 179.6, 172.8, 169.2, 52.1, 40.1, 30.2, 26.6, 26.0, 22.2. HRMS (ESI-MS) *m/z* calculated for $C_{11}H_{22}N_2O_3$ $[M-H]^-$ 229.1558, found 229.1558.

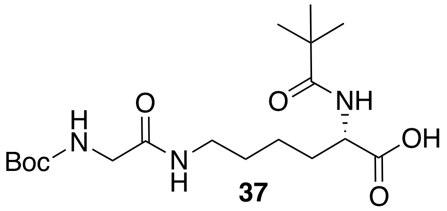

37

N

***N^6-((tert-butoxycarbonyl)glycyl)-N^2-pivaloyl-*L*-lysine (37).** Prepared from **36** (100.0 mg, 0.314 mmol) and Boc-Gly-OSu (85.6 mg, 0.314 mmol) following the general procedure F. Crystallization with CH_2Cl_2/hexane affords the product as a colorless solid (28.2 mg, 0.073 mmol, 23% yield). ^{1}H-NMR (400 MHz, $CDCl_3$) δ, ppm: 6.93 (br-s, 1H), 6.56 (br-s, 1H), 4.50 (td, *J* = 7.4, 5.4 Hz, 1H), 3.80 (d, *J* = 5.8 Hz, 2H), 3.32 – 3.18 (m, 2H), 1.89 (dt, *J* = 13.7, 6.5 Hz, 1H), 1.76 (dt, *J* = 14.1, 7.2 Hz, 1H), 1.63 – 1.53 (m, 2H), 1.52 – 1.28 (m, 2H), 1.44 (s, 9H), 1.22 (s, 9H). ^{13}C-NMR (100 MHz, $CDCl_3$) δ, ppm: 179.3, 174.6, 170.5, 156.5, 80.4, 52.1, 39.1, 38.7, 31.5, 28.6, 28.3, 27.4, 25.5, 22.3. HRMS (ESI-MS) *m/z* calculated for $C_{18}H_{33}N_3O_6$ $[M-H]^-$ 386.2297, found 386.2302.

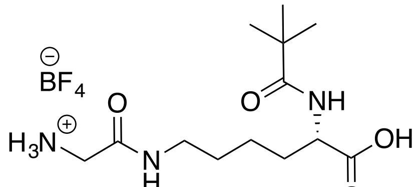

Piv-Lys(Gly)

***(S)*-2-((5-carboxy-5-pivalamidopentyl)amino)-2-oxoethan-1-aminium tetrafluoroborate (Piv-Lys(Gly)).** Prepared from **37** (14.1 mg, 0.036 mmol) following the general procedure G. The product was obtained as a colorless solid (11.0 mg, 0.029 mmol, 81% yield). ^{1}H-NMR (400 MHz, CD_3CN) δ, ppm: 6.83 (br-s, 1H), 6.54 (br-s, 3H), 4.31 (ddd, *J* = 9.2, 7.6, 5.0 Hz, 1H), 3.67 – 3.53 (m, 2H), 3.33 – 3.12 (m, 2H), 1.93 – 1.80 (m, 1H), 1.80 – 1.67 (m, 1H), 1.62 – 1.48 (m, 2H), 1.47 – 1.33 (m, 2H), 1.19 (s, 9H). ^{13}C-NMR (100 MHz, CD_3CN) δ, ppm: 179.0, 173.0, 164.6, 52.1, 40.7, 29.1, 38.3, 30.5, 28.1, 26.7, 22.5. HRMS (ESI-MS) *m/z* calculated for $C_{13}H_{25}N_3O_4$ $[M-H]^-$ 286.1772, found 286.1771.

4.15. Npha-Lys(Gly)

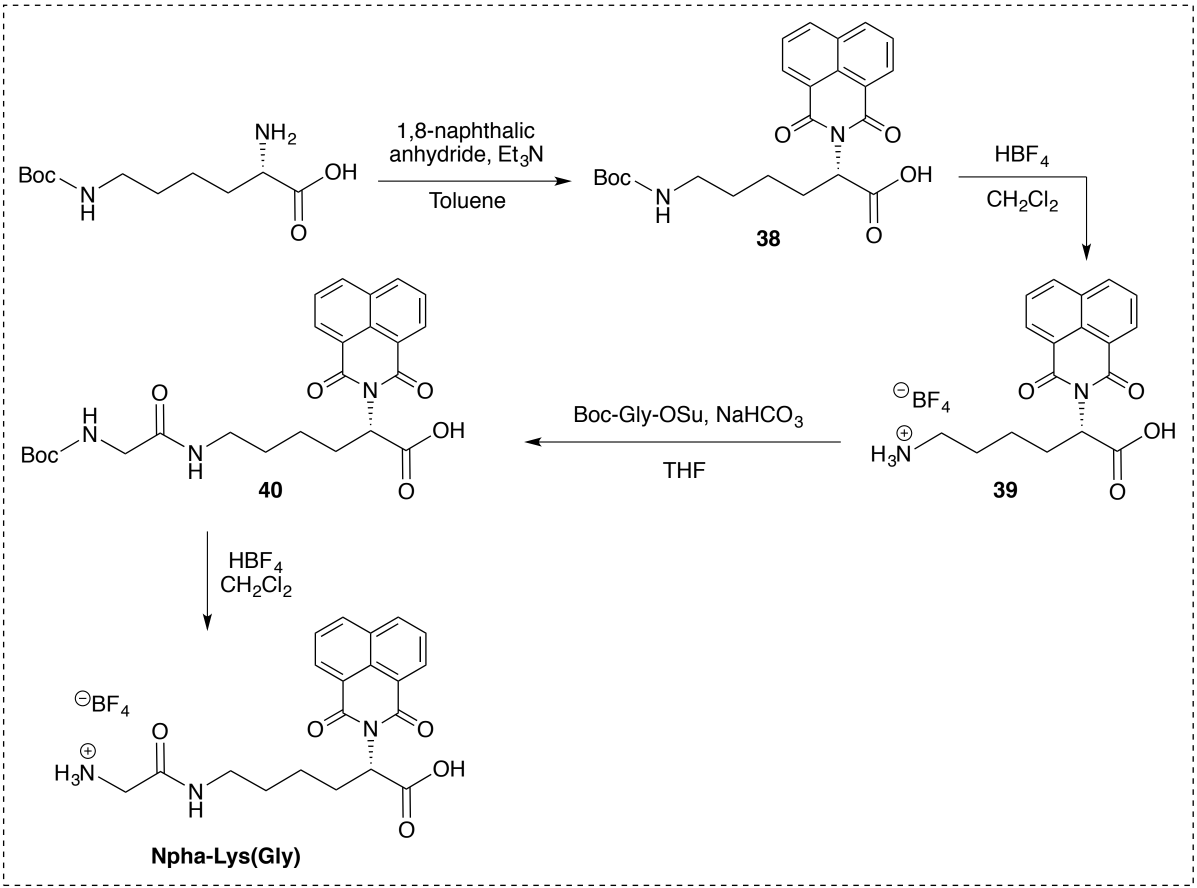

Scheme S16. Synthetic pathway for the synthesis of Piv-Lys(Npha) (Figure 5, **15**).

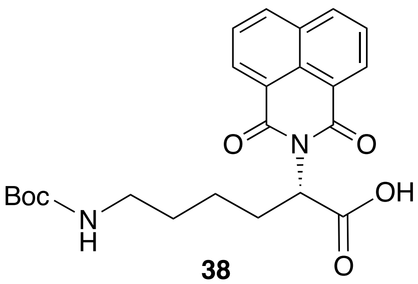

38

***(S)*-6-((*tert*-butoxycarbonyl)amino)-2-(1,3-dioxo-*1H*-benzo [de]isoquinolin-2(*3H)*-yl)hexanoic acid (38).** It was prepared according to a slightly modified reported procedure.[9] H-Lys(Boc)-OH (2.00 g, 8.13 mmol, 1 equiv.) and 1,8-naphthalic anhydride (1.61 g, 8.13 mmol, 1 equiv.) were dissolved in toluene (8 mL, 1M) and 0.1 equiv. of Et_3N were added (113 μL, 0.813 mmol). The mixture was refluxed at 130°C overnight equipped with a Dean-Stark system. After this time, the reaction was cooled down to room temperature and the solvent was removed under vacuum. The crude product was then dissolved in CH_2Cl_2 (10 mL) and washed with aqueous 1M HCl (10 mL). The combined organic layers were dried over anhydrous $MgSO_4$, filtered and evaporated under reduced pressure. Purification by flash chromatography over silica gel (hexane:EtOAc, 1:1) affords the product as a colorless solid (1.52 g, 3.56 mmol, 44% yield). ^{1}H-NMR (400 MHz, CD_3CN) δ, ppm: 8.59

(td, J = 7.2 Hz, 1.1 Hz, 4H), 8.47 (dd, J = 8.3, 1.1 Hz, 2H), 8.39 (dd, J = 8.3 Hz, 0.5 Hz, 2H), 7.03 – 7.81 (m, 4H), 5.68 (dd, J = 9.7, 4.9 Hz, 1H), 5.17 (br-s), 2.96 (q, J = 6.4 Hz, 2H), 2.31 – 2.20 (m, 1H), 2.19 – 2.09 (m, 1H), 1.56 – 1.31 (m, 4H), 1.29 (s, 9H). ^{13}C-NMR (100 MHz, CD_3CN) δ, ppm: 163.8, 161.0, 155.8, 135.6, 134.6, 132.9, 131.3, 127.5, 127.2, 77.8, 52.8, 39.7, 29.5, 28.3, 27.5, 23.3. HRMS (ESI-MS) *m/z* calculated for $C_{23}H_{26}N_2O_6$ $[M-H]^-$ 425.1718, found 425.1709.

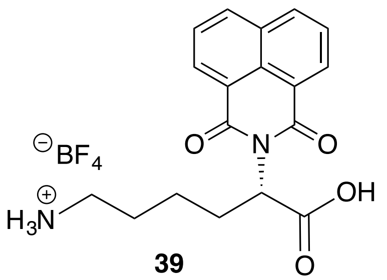

39

***(S)*-5-carboxy-5-(1,3-dioxo-1*H*-benzo[de]isoquinolin-2(*3H)*-yl)pentan-1-aminium tetrafluoroborate (39).** Prepared from **38** (600.0 mg, 1.41 mmol) following the general procedure G. Crystallization with CH_2Cl_2/hexane affords the product as a colorless solid (510.0 mg, 1.23 mmol, 87% yield). ^{1}H-NMR (400 MHz, CD_3CN) δ, ppm: 8.59 (dd, J = 7.2, 14. Hz, 2H), 8.49 – 8.38 (m, 2H), 7.91 – 7.83 (m, 2H), 6.17 (br-s, 3H), 5.69 (dd, J = 9.4, 5.0 Hz, 1H), 2.93 (t, J = 7.5 Hz, 2H), 2.39 – 2.25 (m, 1H), 2.21 – 2.07 (m, 1H), 1.80 – 1.58 (m, 2H), 1.54 – 1.31 (m, 2H). ^{13}C-NMR (100 MHz, CD_3CN) δ, ppm: 170.6, 163.9, 135.6, 134.7, 132.9, 131.4, 127.3, 122.1, 52.5, 40.1, 28.0, 26.3, 22.8. HRMS (ESI-MS) *m/z* calculated for $C_{18}H_{18}N_2O_4$ $[M-H]^-$ 325.1194, found 325.1202.

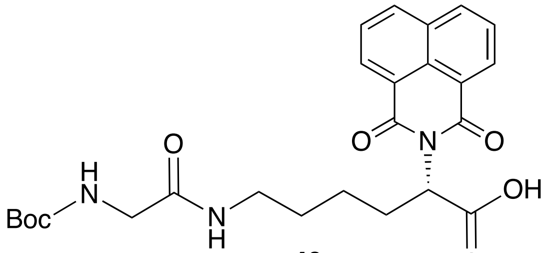

40

***(S)*-6-(2-((*tert*-butoxycarbonyl)amino)acetamido) -2-(1,3-dioxo-*1H*-benzo[de]isoquinolin-2*(3H)*-yl)hexanoic acid (40).** Prepared from **39** (46.0 mg, 0.111 mmol) and Boc-Gly-OSu (30.2 mg, 0.111 mmol) following the general procedure F. Crystallization with CH_2Cl_2/hexane affords the product as a colorless solid (12.1 mg, 0.025 mmol, 23% yield). ^{1}H-NMR (400 MHz, $CDCl_3$) δ, ppm: 8.59 (d, J = 7.2 Hz, 2H), 8.24 (d, J = 8.2 Hz, 2H), 7.77 (t, J = 7.7 Hz, 2H), 6.61 (br-s, 1H), 5.71 (dd, J = 9.0, 5.1 Hz, 1H), 5.50 (br-s, 1H), 3.78 (s, 2H), 3.24 (d, J = 6.5 Hz, 2H), 2.48 – 2.29 (m, 1H), 2.26 – 2.07 (m, 1H), 1.70 – 1.54 (m, 2H), 1.48 – 1.27 (m, 2H), 1.42 (s, 9H). ^{13}C-NMR (100 MHz, $CDCl_3$) δ, ppm: 173.4, 172.1, 168.7, 164.0, 134.4, 131.8, 131.6, 128.3, 127.0, 122.1, 53.0, 39.3, 28.6, 28.3, 25.6, 25.4, 23.7. HRMS (ESI-MS) *m/z* calculated for $C_{25}H_{29}N_3O_7$ $[M-H]^-$ 482.1933, found 482.1934.

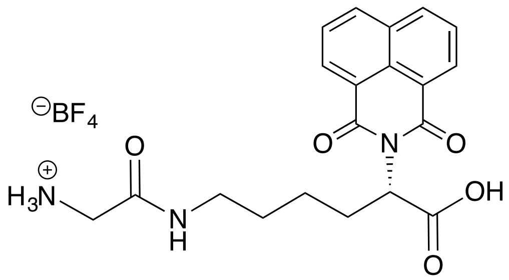

Npha-Lys(Gly)

(*S)*-2-((5-carboxy-5-(1,3-dioxo-*1H*-benzo[de] isoquinolin-2(*3H)*-yl)pentyl)amino)-2-oxoethan-1-aminium tetrafluoroborate (Npha-Lys-Gly). Prepared from **40** (12.1 mg, 0.025 mmol) following the general procedure G. The product was obtained as a colorless solid (9.3 mg, 0.020 mmol, 79% yield). ^{1}H-NMR (400 MHz, CD_3CN) δ, ppm: 8.60 (dd, *J* = 7.4, 1.2 Hz, 2H), 8.43 (dd, *J* = 8.3, 1.2 Hz, 2H), 7.88 (dd, *J* = 8.3, 7.3 Hz, 2H), 6.70 (br-s, 1H), 6.52 (br-s, 3H), 5.68 (dd, *J* = 9.3, 5.0 Hz, 1H), 3.61 – 3.49 (m, 2H), 3.28 – 3.11 (m, 2H), 2.36 – 2.11 (m, 4H), 1.63 – 1.48 (m, 2H), 1.36 – 1.28 (m, 2H). ^{13}C-NMR (100 MHz, CD_3CN) δ, ppm: 172.0, 170.6, 163.9, 134.7, 131.8, 131.4, 28.1, 127.3, 122.1, 52.7, 39.1, 28.5, 28.2, 25.2, 23.2. HRMS (ESI-MS) *m/z* calculated for $C_{20}H_{21}N_3O_5$ $[M-H]^-$ 382.1408, found 382.1414.

5. NMR analysis of the binding

5.1. Binding of 1 to *(S,S)*-^{CR,TIPS}Zn

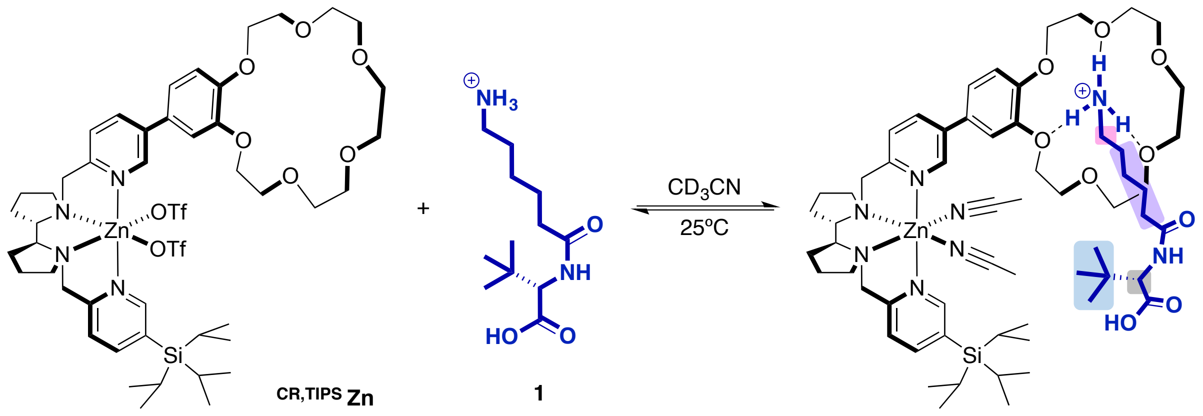

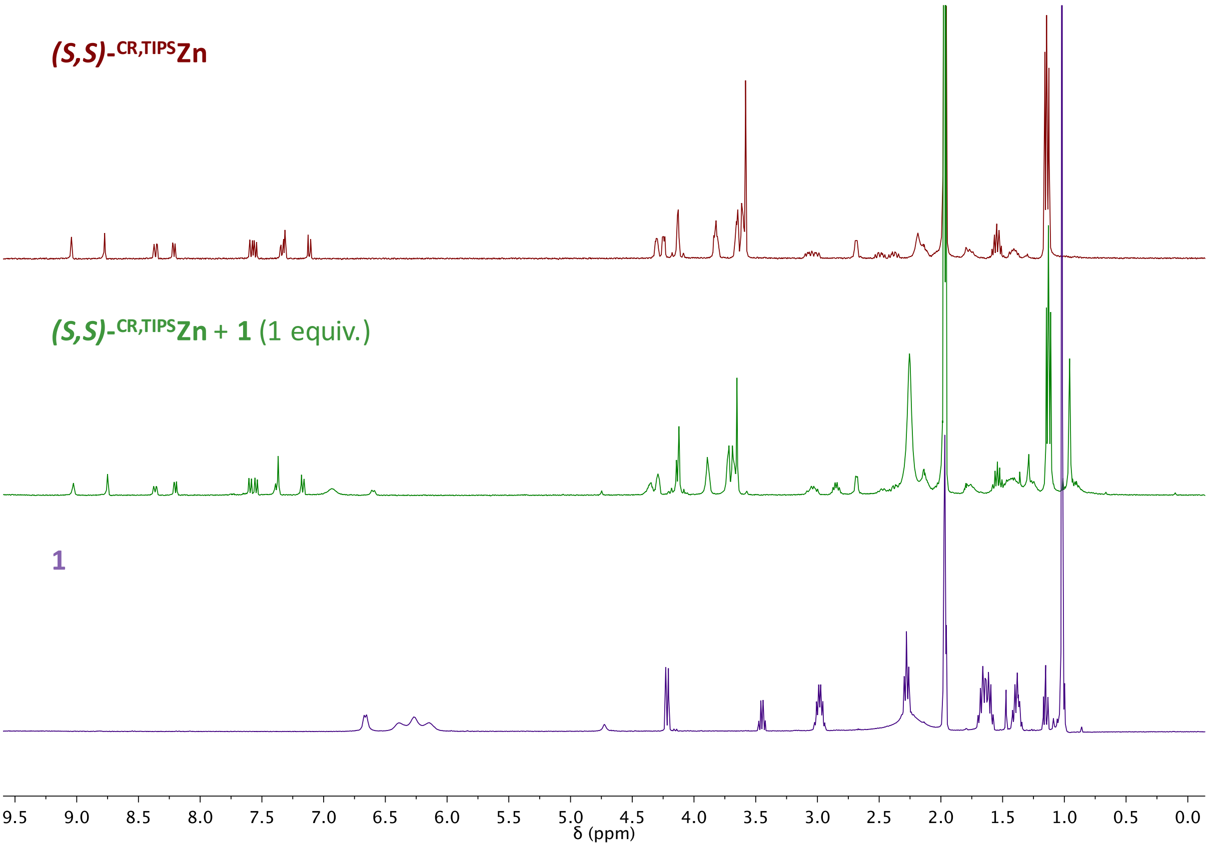

Figure S1. ^{1}H-NMR spectra of *(S,S)*-^{CR,TIPS}Zn (red spectrum, top), **1** : *(S,S)*-^{CR,TIPS}Zn (1:1 mixture, green spectrum, middle) and **1** (purple spectrum, bottom) in CD_3CN (2 mM) at 25°C.

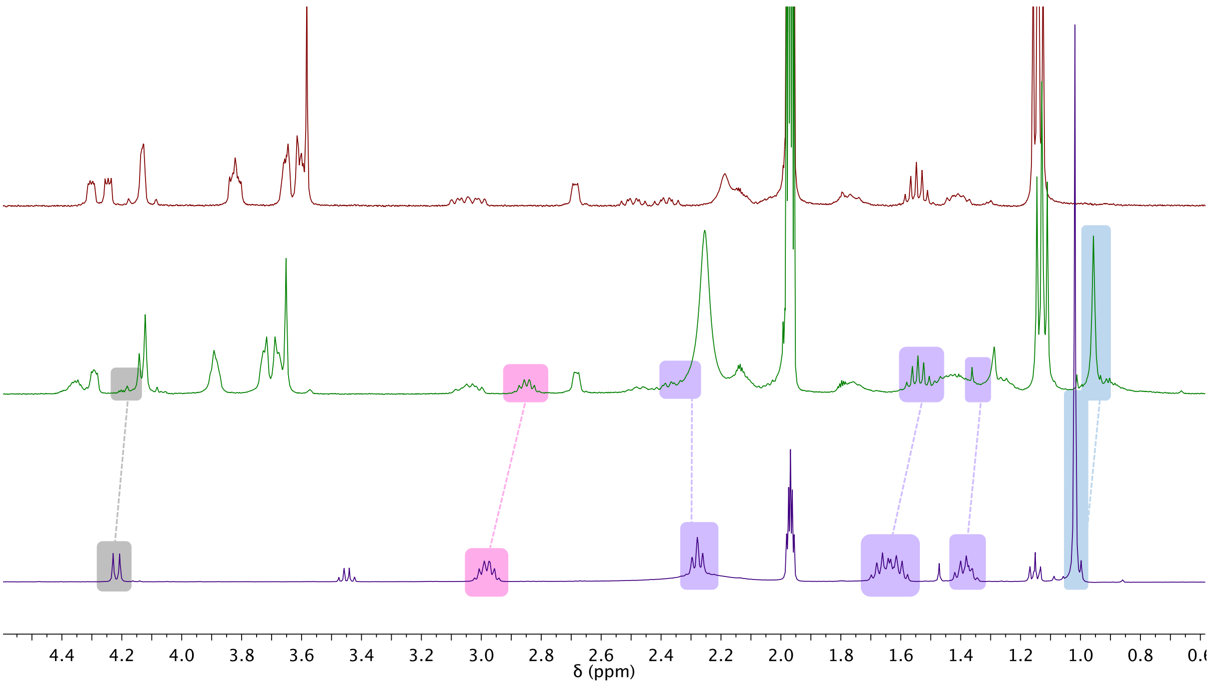

Figure S2. Enlargement of the aliphatic region of the above spectra (Figure S1). The shift of the signals of **1** is evidenced.

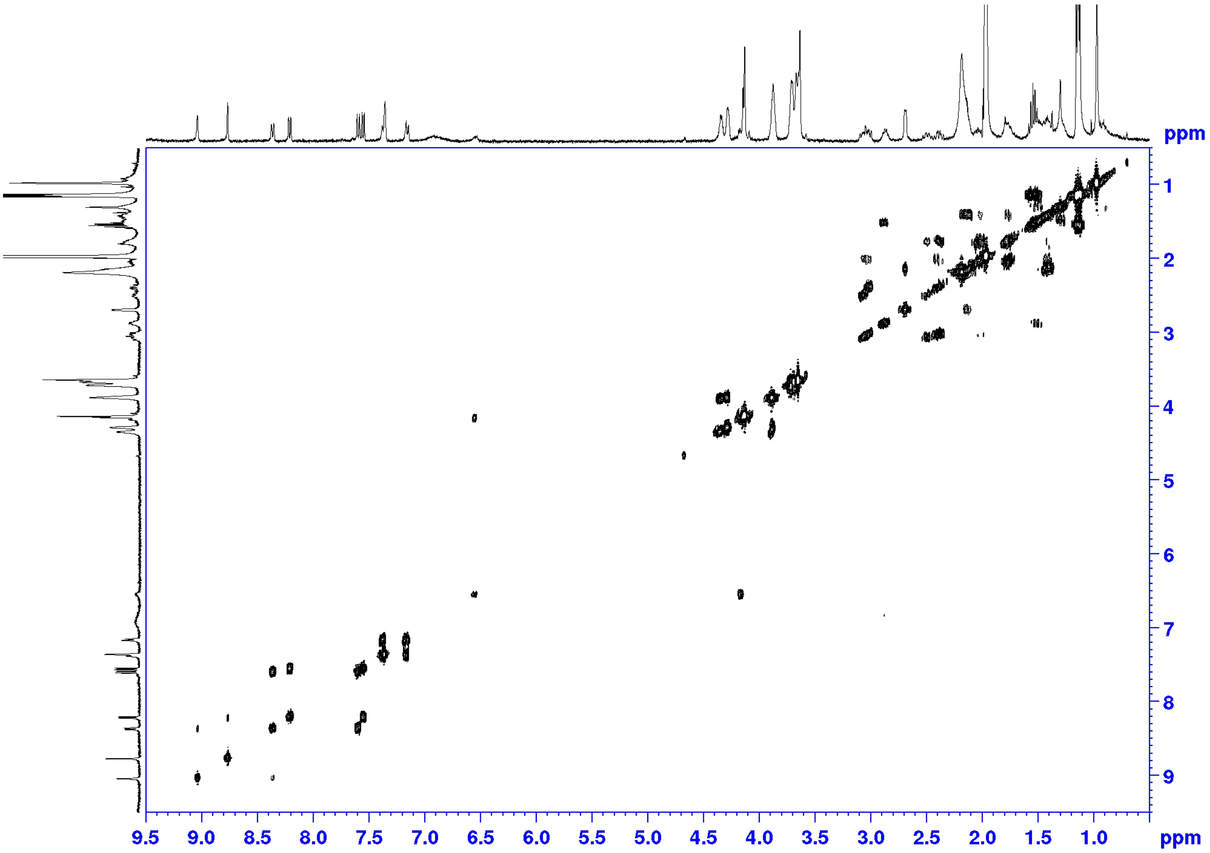

Figure S3. COSY spectrum of **1** : *(S,S)*-^{CR,TIPS}Zn (1:1 mixture) in CD_3CN (2 mM) at 25°C.

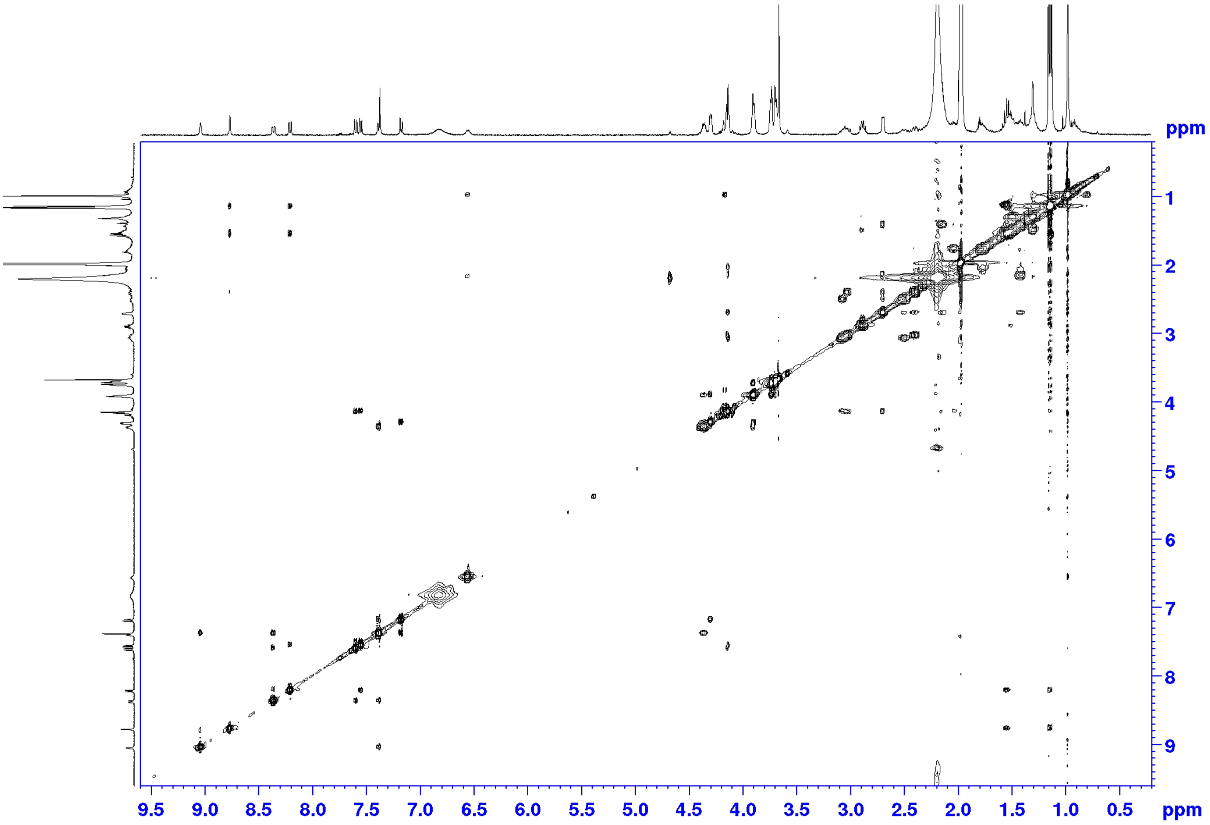

Figure S4. NOESY spectrum of **1** : *(S,S)*-^{CR,TIPS}Zn (1:1 mixture) in CD_3CN (2 mM; mixing time d_8=0.8s) at 25°C.

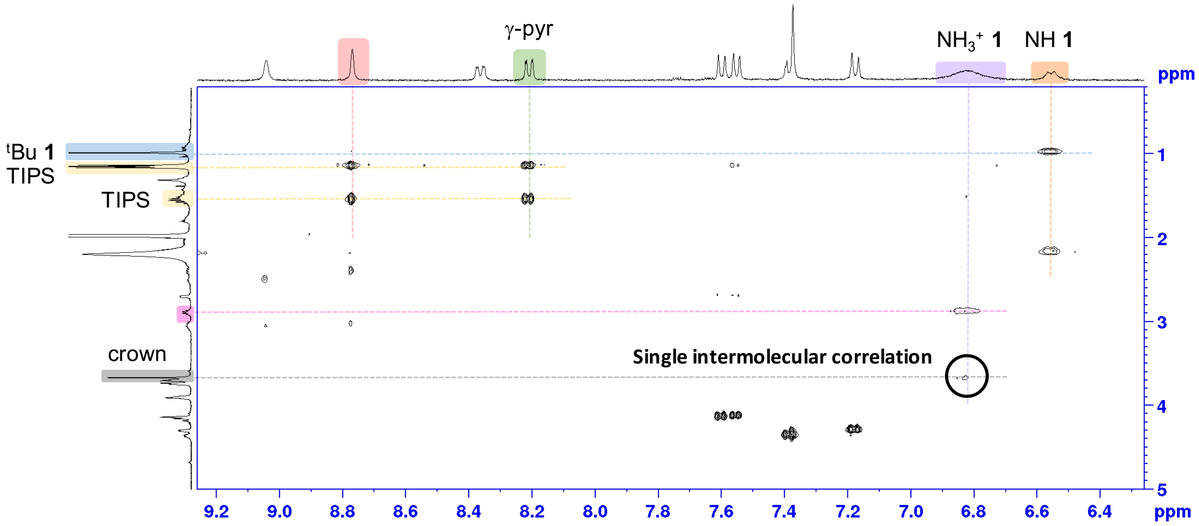

Figure S5. Enlargement of the aromatic-aliphatic cross peaks of the above NOESY spectrum (Figure S4). Note the absence of intermolecular NOESY correlations. The only intermolecular correlation is between the NH_3^+ of **1** and the crown signals (see also the scheme at right).

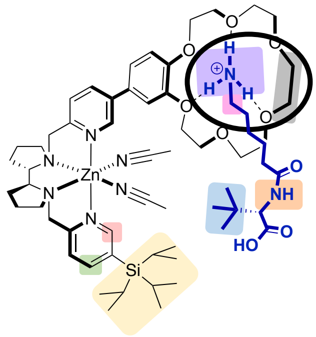

5.2. Titration of *(S,S)*-^{CR,TIPS}Zn with 1

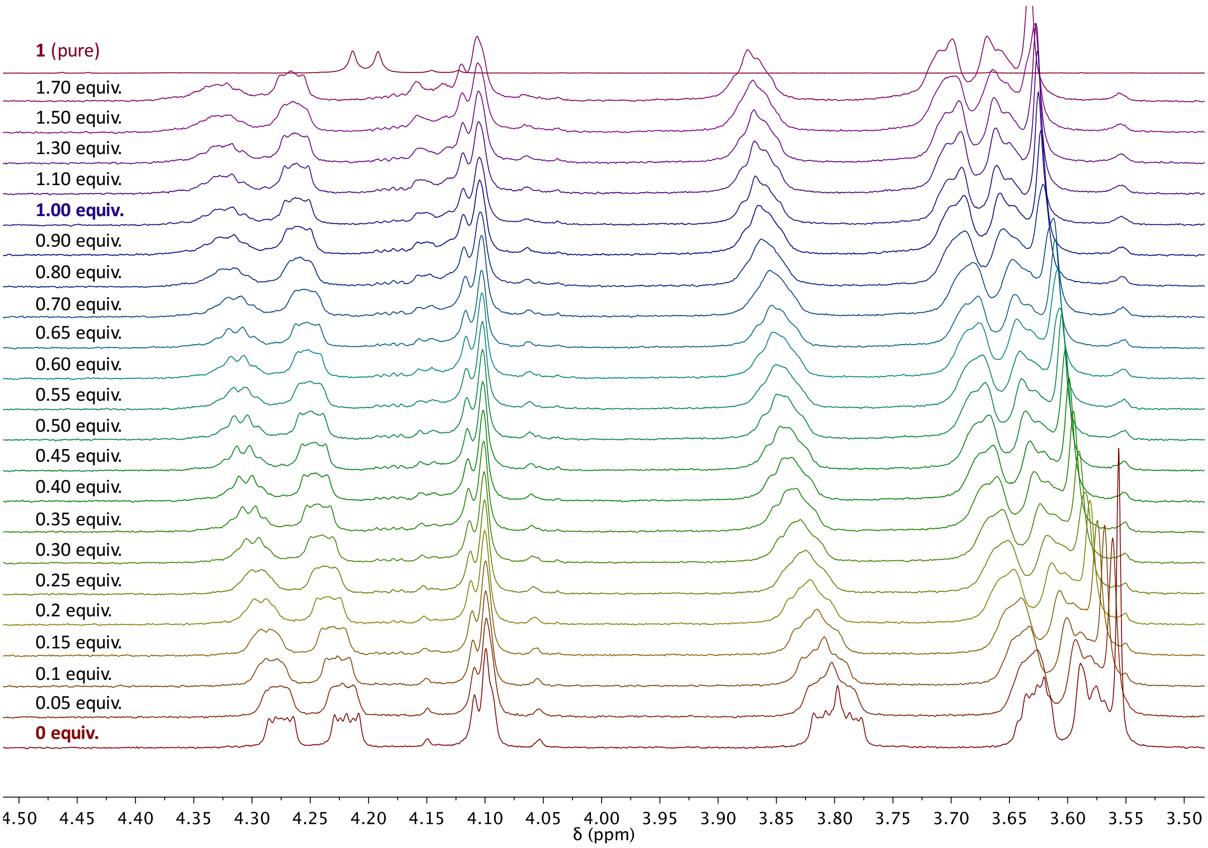

Figure S6. Downfield shift of the crown signals during the titration of ***(S,S)***-$^{\mathbf{CR,TIPS}}$**pdpZn** (2mM) with **1** in CD_3CN at 25°C (enlargement of crown region).

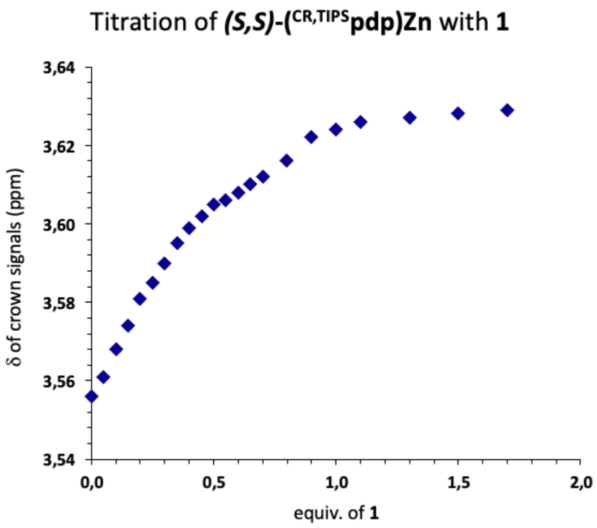

Figure S7. Tritration curve of the above NMR expermients (Figure S6). The behavior at ca 0.5 equivalents suggests the formation of higher stoichiometry adducts in defect of **1**, which do not allow to draw a reliable binding constant. Anyway, this curve is consistent with the formation of 1:1 adducts at millimolar concentrations.

5.3. Addition of a base to favor carboxylate coordination to Zn

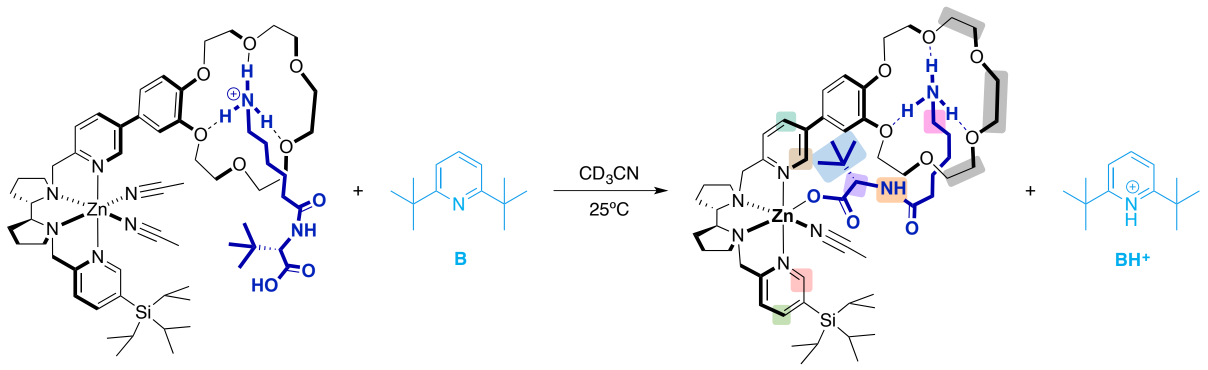

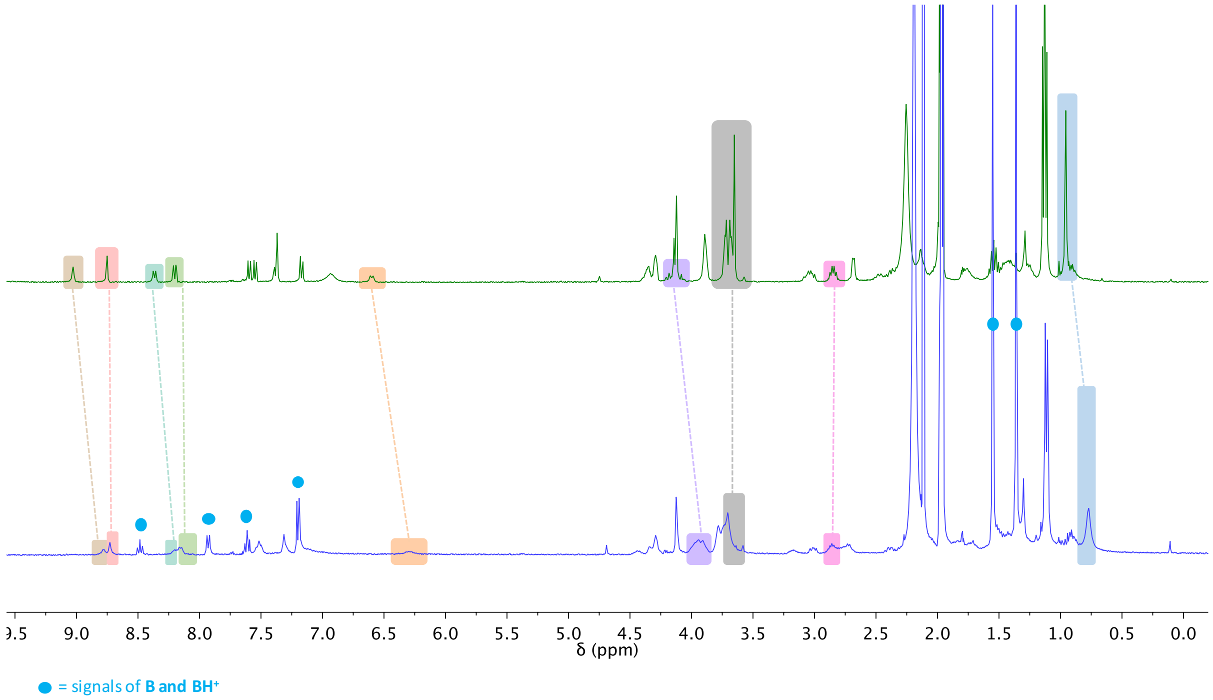

Figure S8. ^{1}H-NMR spectra of a 1:1 mixture of **1** : *(S,S)*-^{CR,TIPS}Zn in CD$_3$CN (2 mM) at 25°C before (green spectrum, top) and after addition of 1.5 equiv. of 2,6-lutidine (blue spectrum, bottom; signals of **B** are denoted with a light blue circle). The main signal shifts are evidenced.

<u>Comments</u>: The signals of the carboxylic acid containing part of **1** (α-C-H in purple, *tert*-butyl in blue and NH in orange) are all shifted upfield, suggesting removal of the acidic proton. Moreover, the signals of the crown-containing pyridine are also shifted upfield, in particular the α-proton (light brown). On the other hand, the signals of the crown ether (grey) and of the $CH_2NH_3^+$ lateral chain (pink) are essentially unaltered, which indicate that these moieties (and their binding) are unaffected by the addition of a base. Also, the TIPS-containing pyridine is essentially not shifted (i.e. in particular, the α-C-H, red), suggesting the lack of close interactions with the carboxylate.

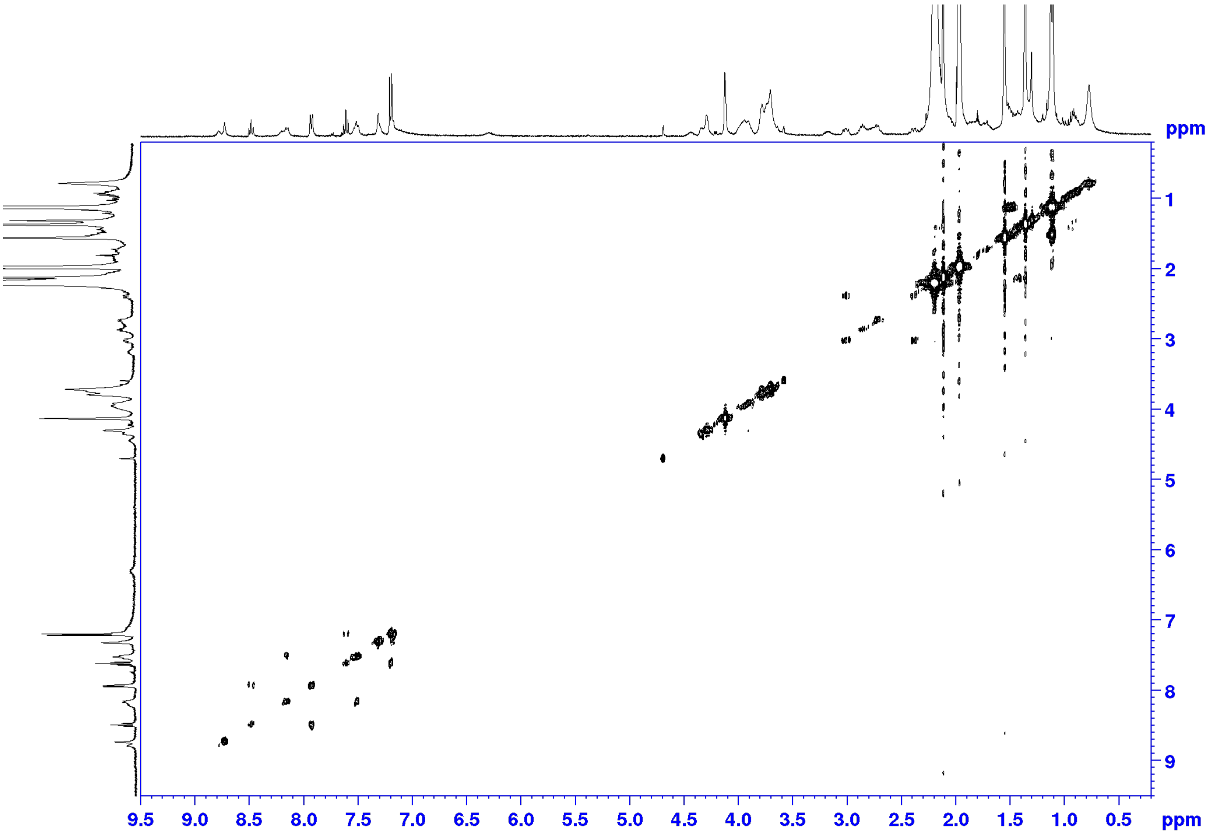

Figure S9. COSY spectrum of a 1:1 mixture of **1** : *(S,S)*-^{CR,TIPS}Zn (1:1 mixture) in CD_3CN (2 mM) at 25°C after addition of 1.5 equivalents of **B**.

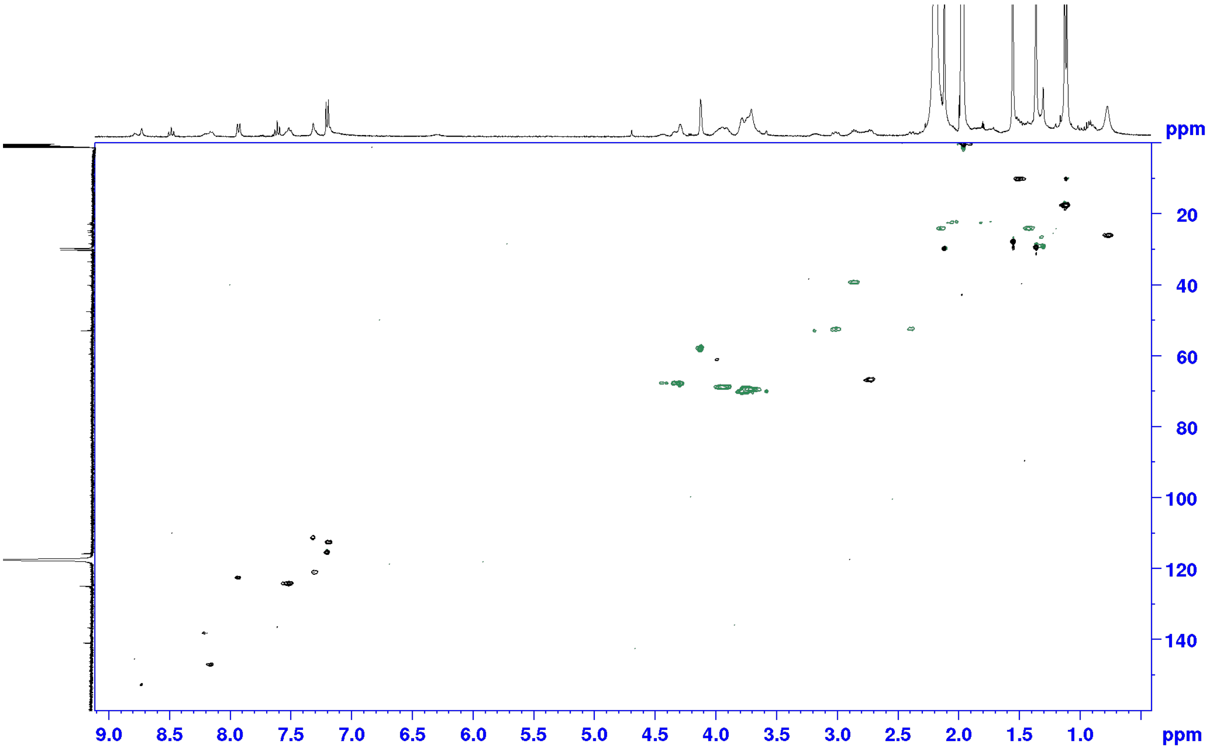

Figure S10. Phase-edited HSQC spectrum of a 1:1 mixture of **1** : *(S,S)*-^{CR,TIPS}Zn (1:1 mixture) in CD_3CN (2 mM) at 25°C after addition of 1.5 equivalents of **B** (CH and CH_3 signals are in positive phase, black signals; while CH_2 signals are in negative phase, green signals).

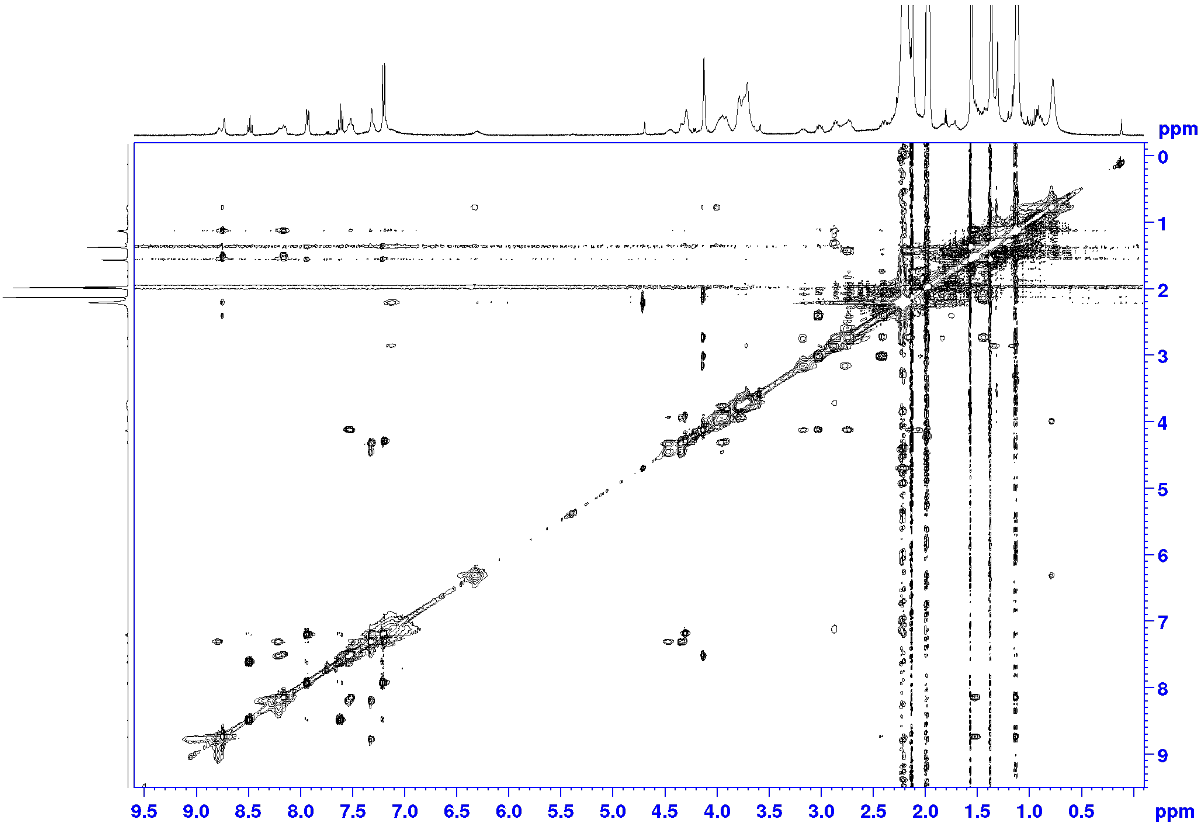

Figure S11. NOESY spectrum of a 1:1 mixture of **1** : *(S,S)*-^{CR,TIPS}Zn (1:1 mixture) in CD_3CN (2 mM) at 25°C after addition of 1.5 equivalents of **B** (mixing time d_8=0.8s).

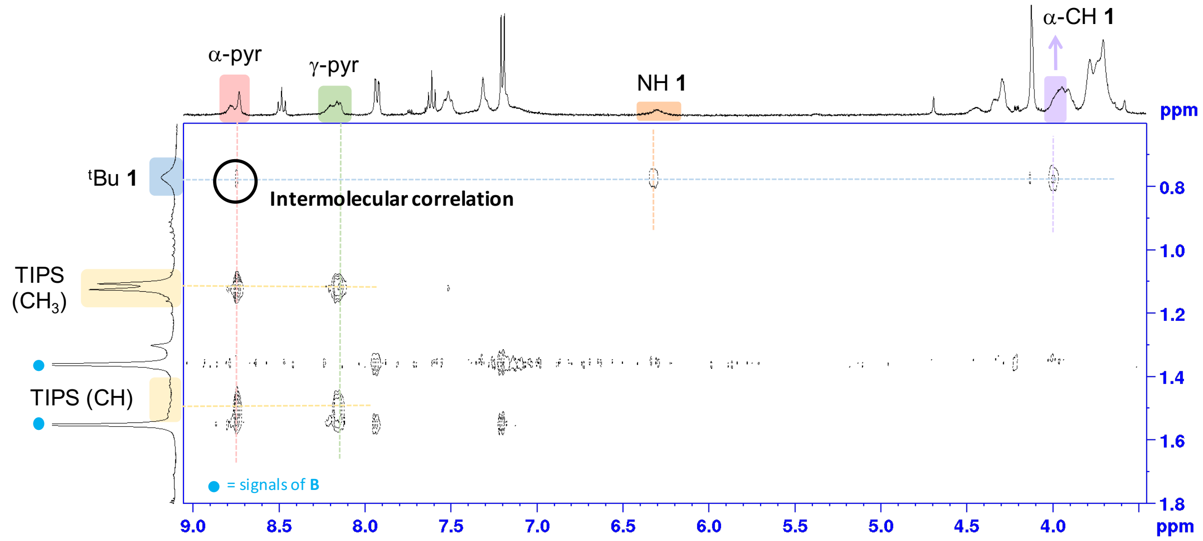

Figure S12. Enlargement of the aromatic-aliphatic cross peaks regions of the above NOESY spectrum (Figure S11). Note the intermolecular NOESY correlation between the *tert*-butyl signals of **1** and the α-pyridine proton single of ***(S,S)*-^{CR,TIPS}Zn** (see also the scheme at right). Peaks marked with a blue circle are *tert*-butyl signals of **B** (protonated and not protonated).

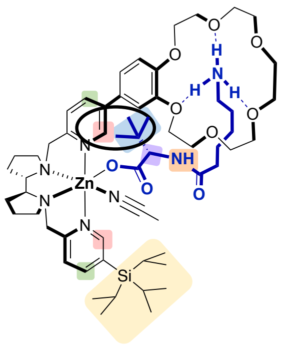

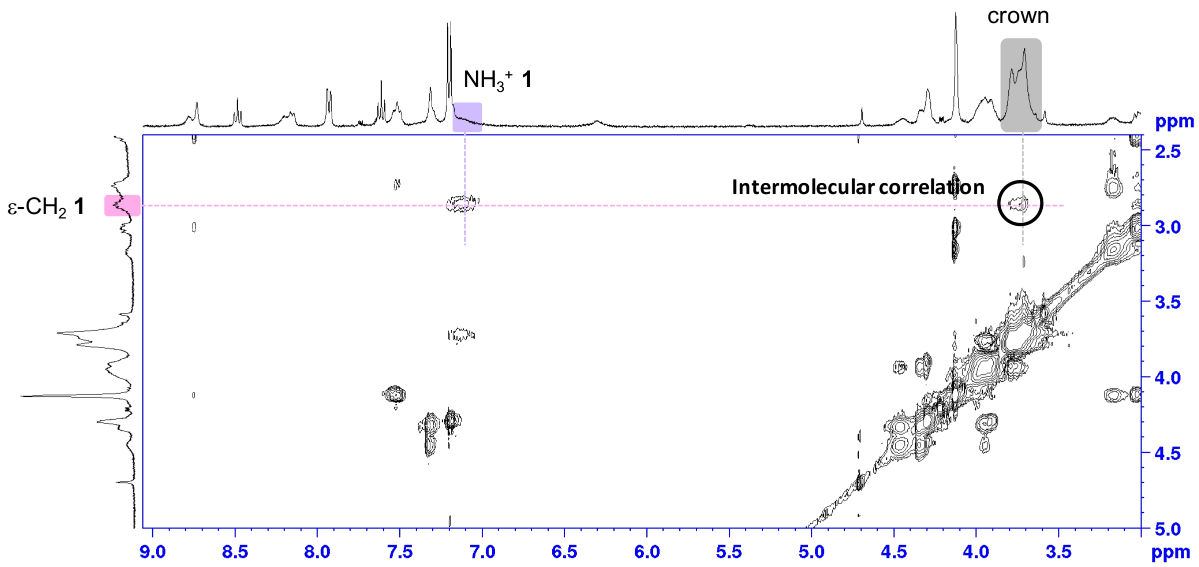

Figure S13. Enlargement of the above NOESY spectrum (S11). Note the intermolecular NOESY correlation between the $CH_2NH_3^+$ of **1** and the crown ether signals suggesting that the ammonium remains bound to the host after treatment with base.

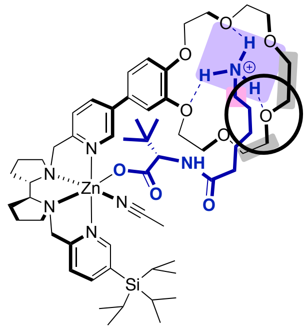

5.4. Control experiment: Binding of 1 to *(S,S)*-^{H}Zn

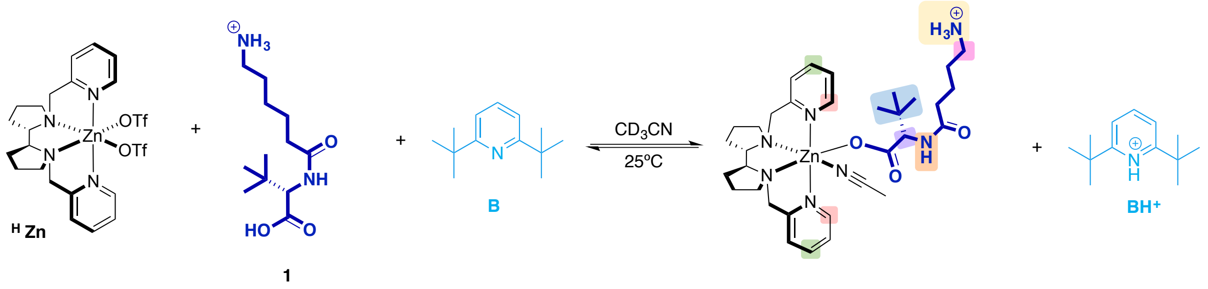

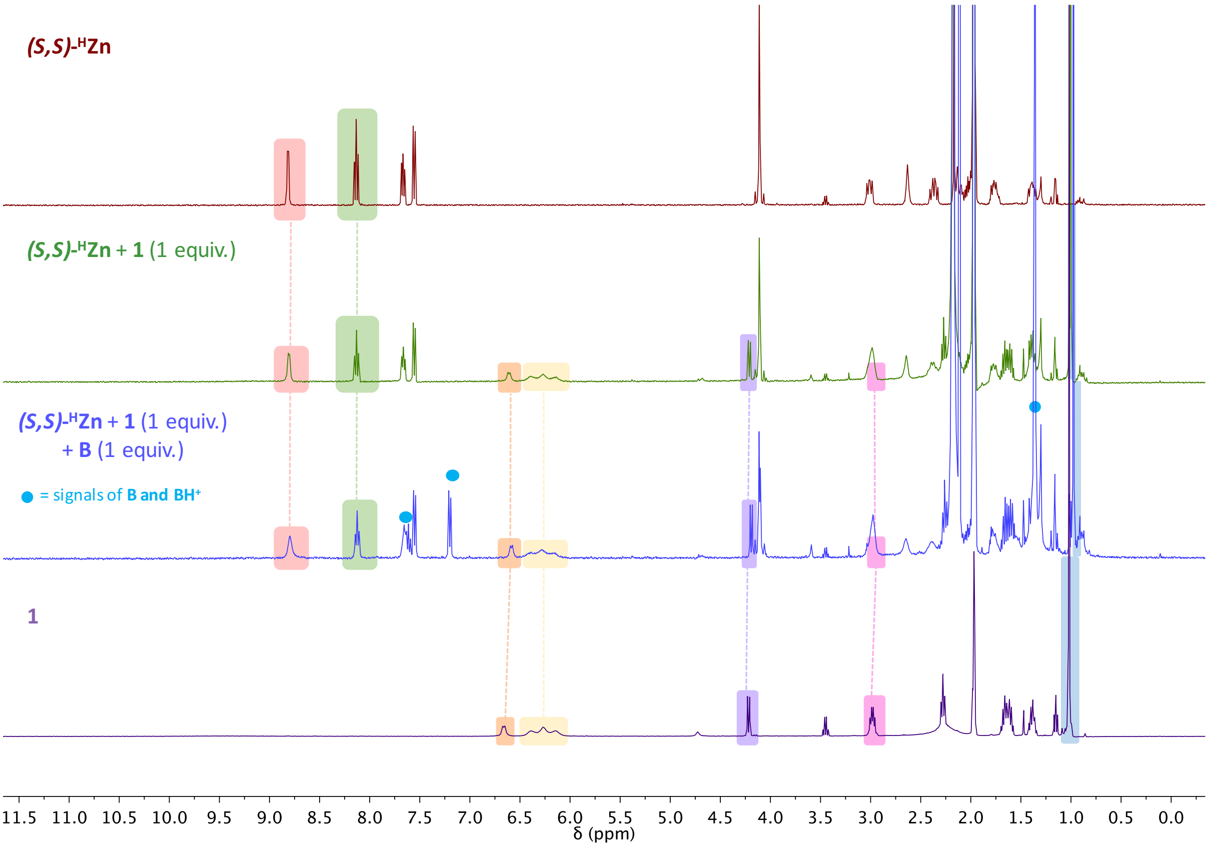

Figure S14. ^{1}H-NMR spectra of *(S,S)*-^{H}Zn (red spectrum, top), a 1:1 mixture of **1** and *(S,S)*-^{H}Zn before (green spectrum) and after addition of 1 equiv. of **B** (blue spectrum) and **1** (purple spectrum, bottom) in CD_3CN (2 mM) at 25°C.

<u>Comments</u>: Note that the signals of the three components undergo very small shifts, if any, upon combination, suggesting lack of significant interactions between the Zn complex and the carboxylate or base **B**. This observation highlights the importance of anchoring the carboxylic acid in the second coordination sphere to observe any appreciable interaction.

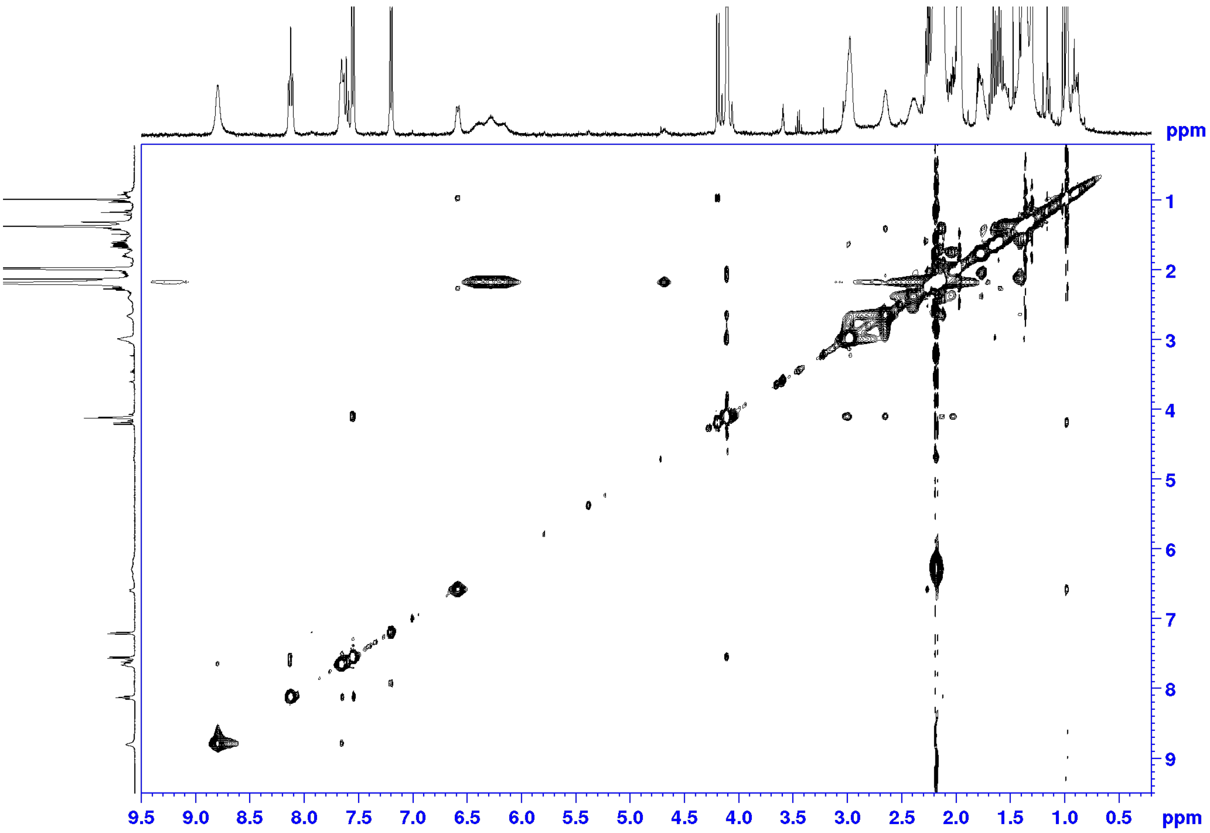

Figure S15. NOESY of a 1:1:1 mixture of *(S,S)*-^{H}Zn, **1** and base **B** (mixing time d_8=0.8s). There are no intermolecular correlations, indicating that carboxylate binding, if occurs, is weak and/or exchanges at a fast timescale, contrarily to what observed with *(S,S)*-^{CR,TIPS}Zn.

6. HRMS analysis of the binding

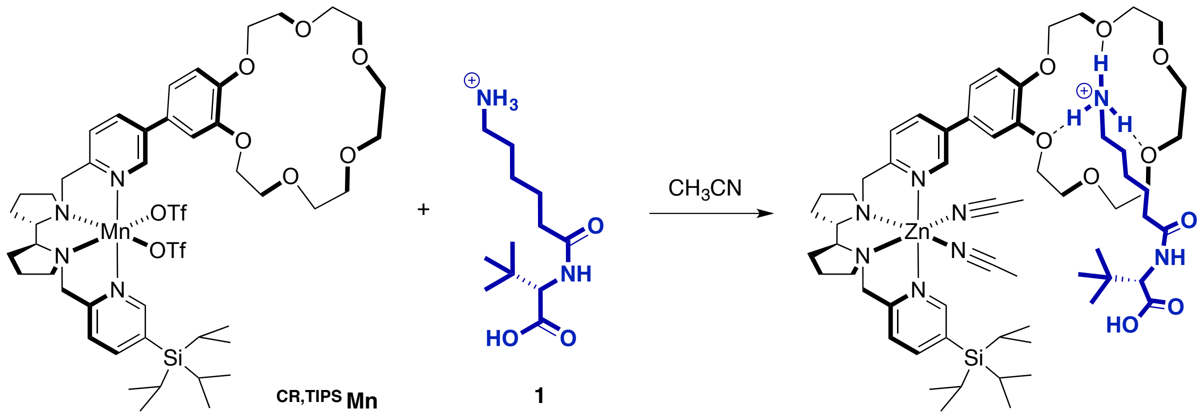

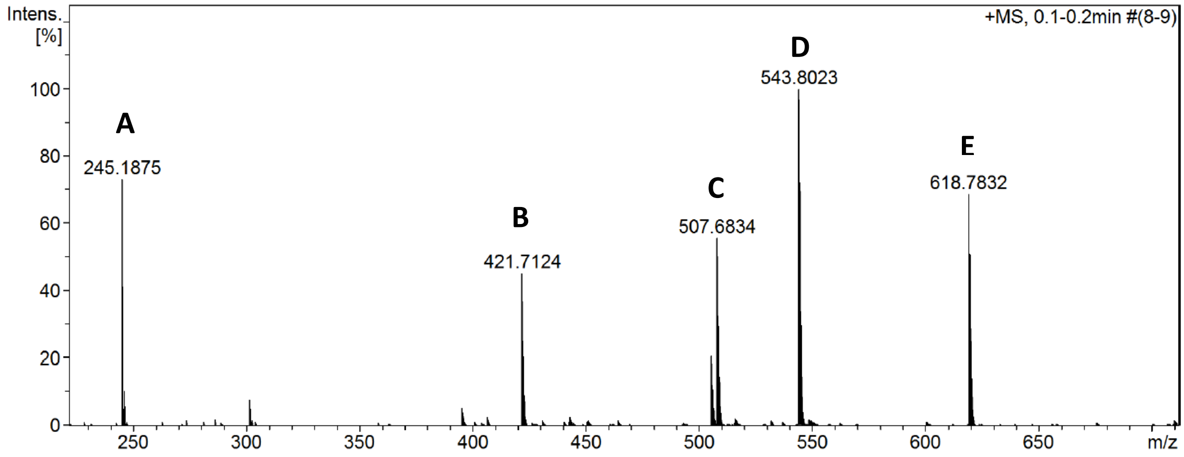

Figure S16. HRMS of the mixture of ^{CR,TIPS}**Mn** and **1** (1:1)

A

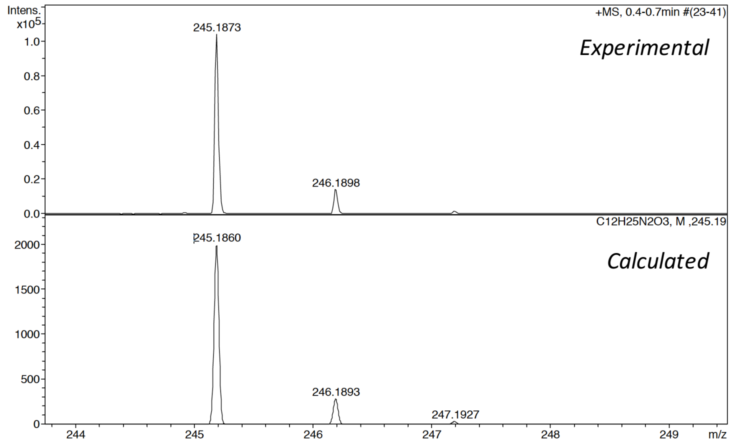

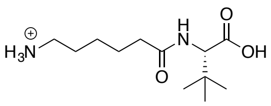

Chemical Formula: $C_{12}H_{25}N_2O_3^+$
Exact Mass: 245,1860

Figure S17. Isotopic pattern obtained (top) and calculated (bottom) for *m/z* = 245 (Figure S16).

B

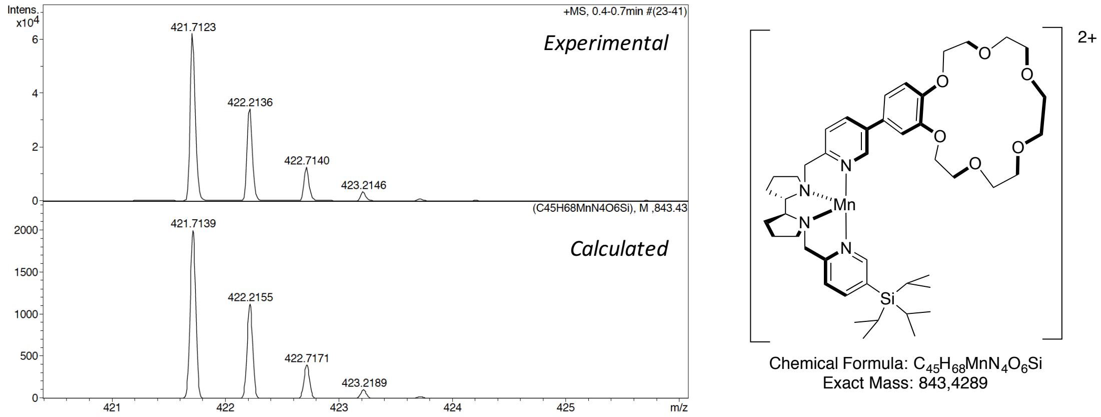

Chemical Formula: $C_{45}H_{68}MnN_4O_6Si$
Exact Mass: 843,4289

Figure S18. Isotopic pattern obtained (top) and calculated (bottom) for *m/z* = 421 (Figure S16).

C

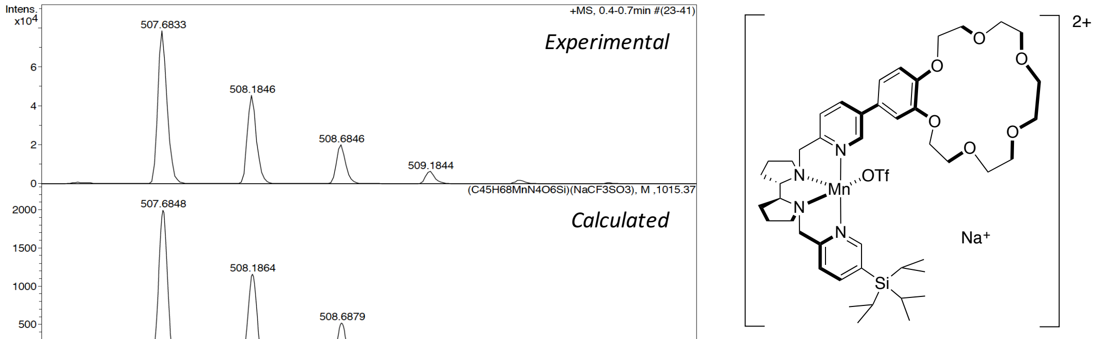

Chemical Formula: $C_{46}H_{68}F_3MnN_4NaO_9SSi^+$
Exact Mass: 1015,3701

Figure S19. Isotopic pattern obtained (top) and calculated (bottom) for *m/z* = 507 (Figure S16).

D

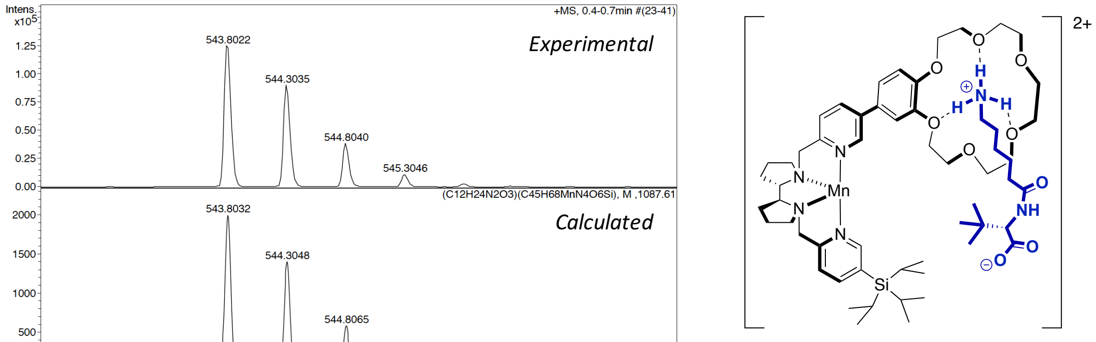

Chemical Formula: $C_{57}H_{92}MnN_6O_9Si$
Exact Mass: 1087,6076

Figure S20. Isotopic pattern obtained (top) and calculated (bottom) for *m/z* = 543 (Figure S16).

E

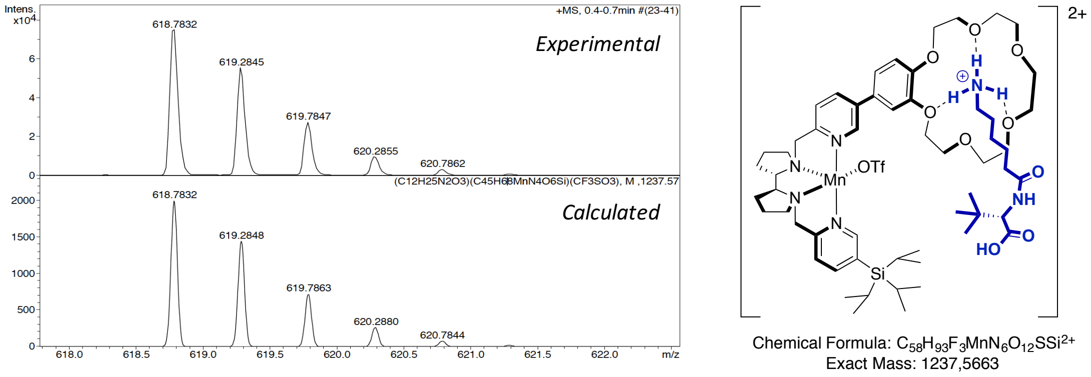

Figure S21. Isotopic pattern obtained (top) and calculated (bottom) for *m/z* = 618 (Figure S16).

7. Reaction conditions for the epoxidation of olefins

A CH_3CN solution (200 μL, 0.1M) of substrate (22 μmol, 1 equiv.) and *(R,R)*-[Mn(OTf)$_2$(^{TIPS,CR}pdp)] (0.22 μmol, 1 mol%) was prepared in a 2 mL vial equipped with a stirring bar. 1.5 mol% of carboxylic acid (0.33 μmol) was added and the resulting mixture was cooled at -40 °C with an acetonitrile/N_2 bath. 150 μL (44 μmol, 2 equiv.) of a 0.3M H_2O_2 solution in CH_3CN (diluted from 50% in water, Aldrich) were added by syringe pump over 30 minutes. Then, 0.5 equiv. of internal standard (biphenyl) was added to the solution and it was filtered through a small plug containing silica, basic alumina and $MgSO_4$, which was subsequently rinsed with 2 mL of EtOAc. The mixture was directly analyzed by GC.

8. Control experiments

8.1. Selected experiments using 1 equiv. of amino acid

These experiments were done following the standard reaction conditions (SI, 7), but adding different amounts of amino acid **3**.

Table S1. *Epoxidation of cis-2-octene using different amount of amino acid **3**.*

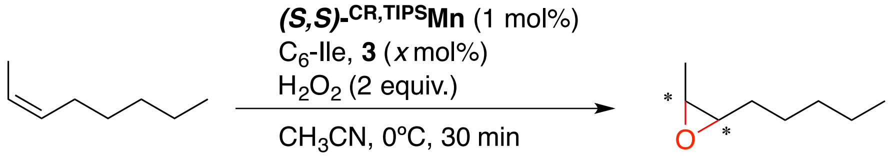

Entry	3 (*x* mol%)	Conv. (%)[a]	Yield (%)[a]	ee (%)[a]
1	1 mol%	87	57	3
2	1.5 mol%	99	85	3

[a] *Conversions, yields and ee determined by GC. The results are expressed as an average of 2 runs with an error <5%.*

Table S2. *Epoxidation of styrene using different amount of amino acid **3**.*

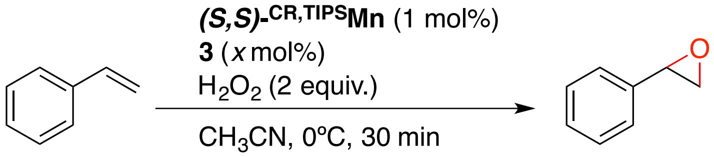

Entry	3 (*x* mol%)	Conv. (%)[a]	Yield (%)[a]	ee (%)[a]
1	1 mol%	42	33	39
2	1.5 mol%	64	53	40

[a] *Conversions, yields and ee determined by GC. The results are expressed as an average of 2 runs with an error <5%.*

8.2. Synthesis of the co-ligands

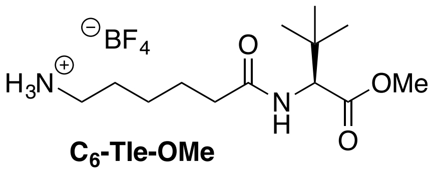

C$_6$-Tle-OMe

***(S)*-6-((1-methoxy-3,3,-dimethyl-1-oxobutan-2-yl)amino)-6-oxohexan-1-aminium tetrafluoroborate**. Prepared from **16** (25.8 mg, 0.072 mmol) following the general procedure G. The product was obtained as a colorless solid (4.0 mg, 0.012 mmol, 16% yield). ^{1}H-NMR (400 MHz, CD_3CN) δ, ppm: 6.66 (d, *J* = 8.4 Hz, 1H), 6.24 (t, *J* = 49.7 Hz, 3H), 4.27 (d, *J* = 8.7 Hz, 1H), 3.69 (s, 3H), 3.07 – 2.86 (m, 2H), 2.32 – 2.25 (m, 2H), 1.71 – 1.52 (m, 4H), 1.47 – 1.29 (m, 2H), 0.99 (s, 9H). ^{13}C-NMR (100 MHz, CD_3CN) δ, ppm: 172.9, 171.8, 60.6, 51.2, 40.0, 34.8, 33.6, 26.0, 25.9, 25.1, 24.1. HRMS (ESI-MS) *m/z* calculated for $C_{13}H_{26}N_2O_3$ $[M+H]^+$ 259.2016, found 259.2017.

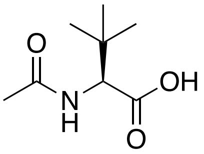

Ac-Tle-OH

***(S)*-2-acetamido-3,3-dimethylbutanoic acid**. It was prepared according to a slightly modified reported procedure.[30] NaOH (2.80 g, 69.3 mmol, 3 equiv.) were dissolved in 70 mL of distilled H_2O and the resulting solution was cooled to 0ºC in an ice bath. L-tertleucine (3.09 g, 23.1 mmol, 1 equiv.) was added and the solution was stirred until it was homogeneous. Then, 1.1 equiv. of acetyl chloride (1.84 mL, 25.4 mmol) in dioxane (20 mL) were added dropwise with an addition funnel. The reaction mixture was stirred at room temperature overnight. After this time, the solution was washed with Et_2O (3 x 20 mL) and the aqueous phases were acidified until pH = 2 using HCl 2M. The product was extracted with EtOAc (2 x 20 mL) and CH_2Cl_2 (2 x 20 mL). The combined organic phases were dried over anhydrous $MgSO_4$, filtered and the solvent was evaporated under reduced pressure. The product was obtained as a colorless solid (1.84 g, 10.6 mmol, 46% yield). Spectral data match those reported in literature.[30] ^{1}H-NMR (400 MHz, CD_3OD) δ, ppm: 4.32 (s, 1H), 2.02 (s, 3H), 1.05 (s, 9H).

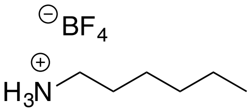

C_6-NH_3^+

Hexan-1-aminium tetrafluoroborate. It was prepared according to a reported procedure.[2] Spectral data match those reported in literature. [2] ^{1}H-NMR (400 MHz, CD_3CN) δ, ppm: 6.63 (br-s, 3H), 2.91 (q, *J* = 6.4 Hz, 2H), 1.67 – 1.51 (m, 2H), 1.40 – 1.18 (m, 6H), 0.93 (t, *J* = 7.1 Hz, 3H).

8.3. Reaction conditions for each type of binding (Figure 4C)

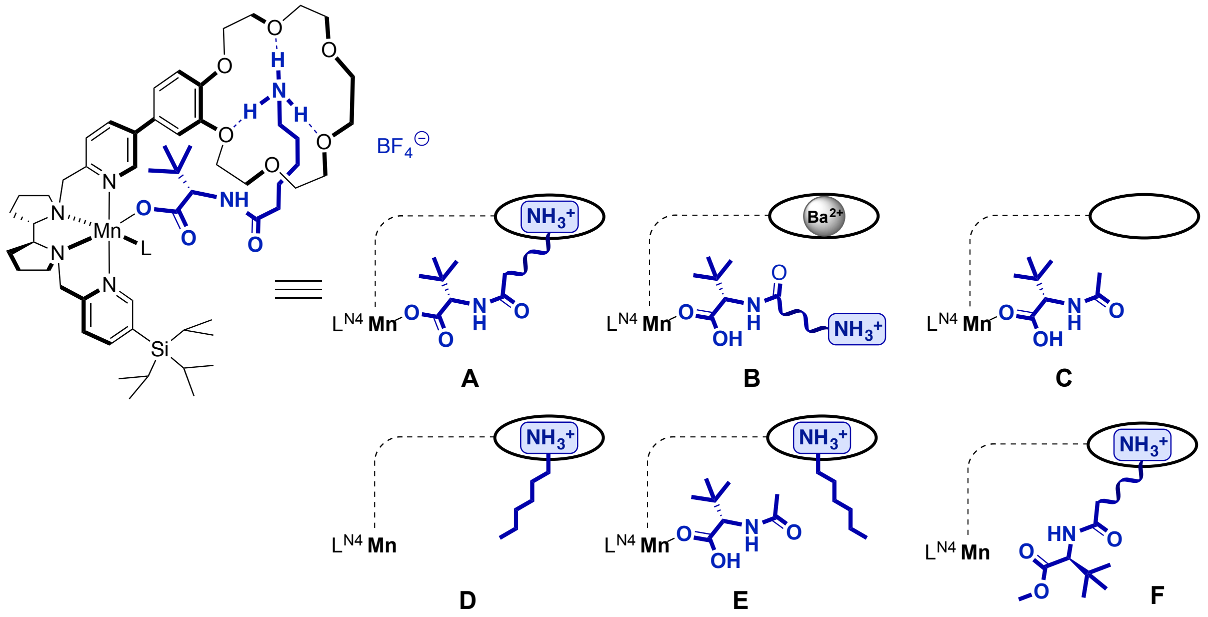

Figure S22. Schematic representation of the different type of bindings.

These experiments were done following the standard reaction conditions (SI, 6), but applying the following modifications:

Binding B: 2 mol% of $Ba(ClO_4)_2$ were added in the reaction mixture.
Binding C: 1.5 mol% of Ac-Tle-OH were used instead of **1**.
Binding D: 1.5 mol% of hexylammonium tetrafluoroborate were used instead of **1**.
Binding E: 1.5 mol% of Ac-Tle-OH and 1.5 mol% of hexylammonium tetrafluoroborate were used instead of **1**.
Binding F: 1.5 mol% of C_6-Tle-OMe were used instead of **1**.

8.4. Selected control experiments in the epoxidation of styrene

Table S3. *Epoxidation of styrene using different carboxylic acids.*

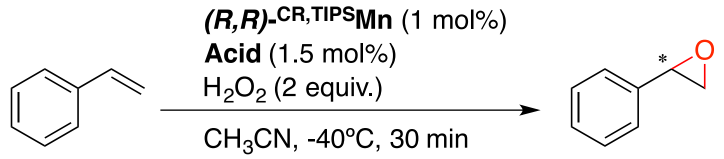

Entry	Acid	Binding[a]	Conv. (%)[b]	Yield (%)[b]	ee (%)[b]
1	C_6-Tle (**1**)	A	93	81	44
2[c]	C_6-Tle (**1**)	B	29	29	18
3	Ac-Tle-OH	E	27	23	16

[a] See Figure S22 [b] Conversions, yields and ee determined by GC. The results are expressed as an average of 2 runs with an error <5%. [c] 2 mol% of $Ba(ClO_4)_2$ were added.

Table S4. *Epoxidation of styrene using different carboxylic acids.*

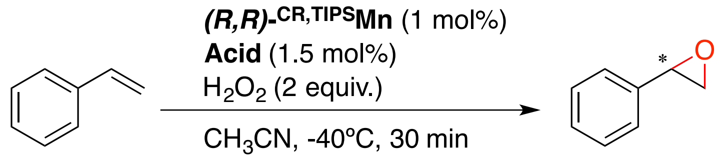

Entry	AcOH	Conv. (%)[a]	Yield (%)[a]	ee (%)[a]
1	AcOH	20	7	2
2	C_6-Tle (**1**)	93	80	44
3	AcOH + **1**	77	76	44

[a] Conversions, yields and ee determined by GC. The results are expressed as an average of 2 runs with an error <5%.

8.5. Competitive reactions using an external carboxylic acid and 1 (C_6-Tle)

Table S5. *Epoxidation of 1-chloro-3-methylbut-2-ene (**S1**) using **1** and different amounts of AcOH.*

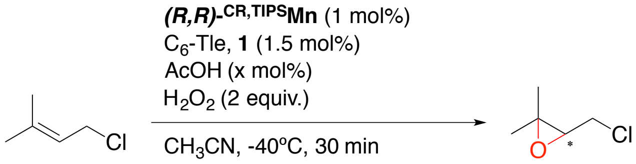

Entry	AcOH	Conv. (%)[a]	Yield (%)[a]	ee (%)[a]
1	Only AcOH	25	10	44
2	0 mol%	88	65	67
3	1.5 mol%	99	77	67
4	3 mol%	98	78	67
5	0.1 equiv.	93	77	67
6	0.5 equiv.	95	75	67
7	1 equiv.	98	79	67
8	5 equiv.	93	78	67

[a] Conversions, yields and ee determined by GC. The results are expressed as an average of 2 runs with an error <5%.

Table S6. *Epoxidation of 1-chloro-3-methylbut-2-ene (**S1**) using **1** and other carboxylic acid in equimolar amounts.*

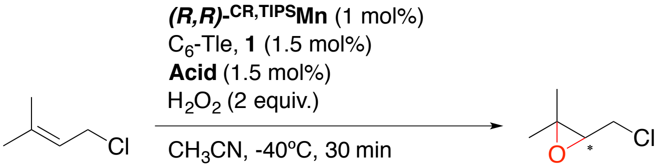

Entry	Acid		Conv. (%)[a]	Yield (%)[a]	ee (%)[a]
1	-	-	88	65	67
2	AcOH	*Blank*[b]	25	10	44
3	AcOH	*1.5 mol%*	99	77	67
4	2-eha[c]	*Blank*[b]	28	10	52
5	2-eha[c]	*1.5 mol%*	83	65	67
6	cPr-OH[d]	*Blank*[b]	38	14	45
7	cPr-OH[d]	*1.5 mol%*	85	69	67

*[a] Conversions, yields and ee determined by GC. The results are expressed as an average of 2 runs with an error <5%. [b] No **1** was added in the reaction [c] 2-ethylhexanoic acid [d] cyclopropanecarboxylic acid*

8.6. Competitive reactions using an external carboxylic acid and 11 (C_6-HcLeu)

Table S7. *Epoxidation of 1-chloro-3-methylbut-2-ene (**S1**) using **11** and different amounts of AcOH.*

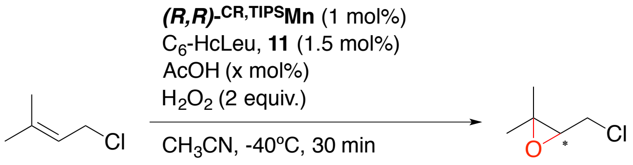

Entry	AcOH	Conv. (%)[a]	Yield (%)[a]	ee (%)[a]
1	Only AcOH	25	10	44
2	0 mol%	99	61	69
3	1.5 mol%	99	78	69
4	3 mol%	99	75	69
5	0.1 equiv.	99	75	69
6	0.5 equiv.	99	75	69
7	1 equiv.	99	77	69
8	5 equiv.	99	79	69

[a] *Conversions, yields and ee determined by GC. The results are expressed as an average of 2 runs with an error <5%.*

Table S8. *Epoxidation of 1-chloro-3-methylbut-2-ene (**S1**) using **11** and other carboxylic acid in equimolar amounts.*

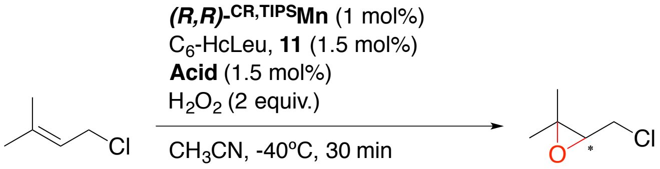

Entry	Acid		Conv. (%)[a]	Yield (%)[a]	ee (%)[a]
1	-	-	99	61	69
2	AcOH	*Blank*[b]	25	10	44
3	AcOH	*1.5 mol%*	99	78	68
4	2-eha[c]	*Blank*[b]	28	10	52
5	2-eha[c]	*1.5 mol%*	92	67	69
6	cPr-OH[d]	*Blank*[b]	38	14	45
7	cPr-OH[d]	*1.5 mol%*	94	66	69

[a] *Conversions, yields and ee determined by GC. The results are expressed as an average of 2 runs with an error <5%.* [b] *No **1** was added in the reaction* [c] *2-ethylhexanoic acid* [d] *cyclopropanecarboxylic acid*

8.7. Determination of the consumed H_2O_2 in the reaction using AcOH

It was done according to a modified reported procedure.[31] The catalysis was run according to the experimental procedure detailed on SI.6. When the addition was finished, a 100 μL aliquot was taken and added to a solution containing 2 mL of distilled water and 0.5 mL of Ti(IV)OSO_4. The solution took a yellow color, which indicates the formation of $[TiO_2]^{2+}$ due to the presence of H_2O_2.[32] The UV-vis spectrum was then recorded, and the absorbance at λ = 406 nm was used to determine the concentration of H_2O_2 according to a calibration curve previously prepared.

9. Optimization of the catalytic system

The optimization of the system was done with two other substrates apart from **S1** (depicted in the manuscript): trans-2-octene and styrene.

9.1. Optimization of catalyst structure

These experiments were done following the standard reaction conditions (SI, 7), but changing the structure of the catalyst (catalysts depicted in Figure 2 of the manuscript) and using amino acid **3** instead of amino acid **1**.

Table S9. *Influence of catalyst structure on the epoxidation of trans-2-octene.*

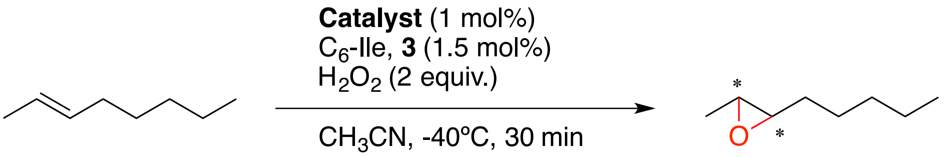

Entry	Catalyst	Conv. (%)[a]	Yield (%)[a]	ee (%)[a]
1	(*S,S*)-^{CR,TIPS}Mn	100	>99	14
2	(*S,S*)-^{CR,TMS}Mn	100	91	17
3	(*S,S*)-^{CR,DMM}Mn	100	81	15

[a] *Conversions, yields and ee determined by GC. The results are expressed as an average of 2-3 runs with an error <5%.*

Table S10. *Influence of catalyst structure on the epoxidation of styrene.*

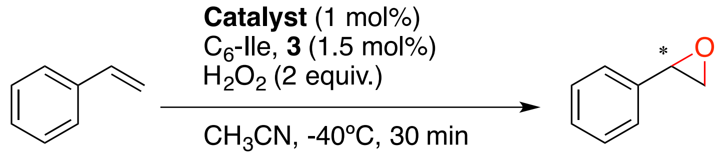

Entry	Catalyst	Conv. (%)[a]	Yield (%)[a]	ee (%)[a]
1	(*S*,*S*)-^{CR,TIPS}Mn	91	64	48
2	(*S*,*S*)-^{CR,TMS}Mn	25	21	44
3	(*S*,*S*)-^{CR,DMM}Mn	99	81	46

[a] *Conversions, yields and ee determined by GC. The results are expressed as an average of 2-3 runs with an error <5%.*

9.2. Optimization of the amino acid structure

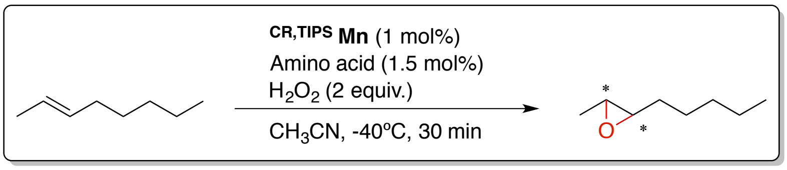

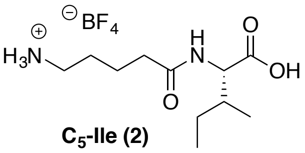

C$_5$-Ile (2)

(S,S): 93% yield, 12% ee
(R,R): 78% yield, 24% ee

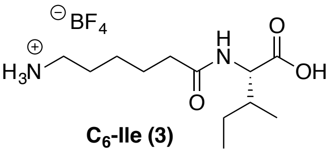

C$_6$-Ile (3)

(S,S): >99% yield, 14% ee
(R,R): 75% yield, 27% ee

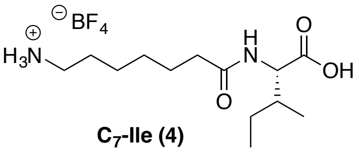

C$_7$-Ile (4)

(S,S): 64% yield, 17% ee
(R,R): 33% yield, 21% ee

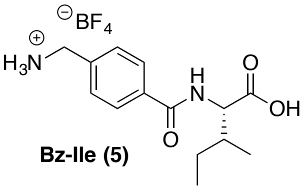

Bz-Ile (5)

(S,S): 11% yield, 9% ee
(R,R): 78% yield, 27% ee

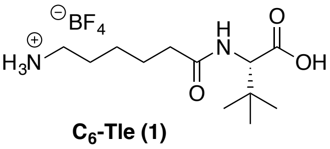

C$_6$-Tle (1)

(S,S): 80% yield, 23% ee
(R,R): 81% yield, 34% ee

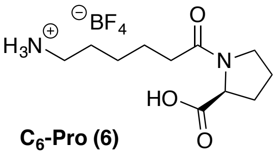

C$_6$-Pro (6)

(S,S): 78% yield, 19% ee
(R,R): 56% yield, 21% ee

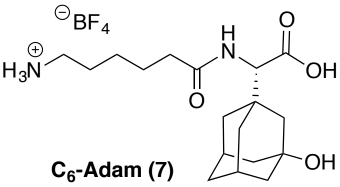

C$_6$-Adam (7)

(S,S): 63% yield, 16% ee
(R,R): 60% yield, 25% ee

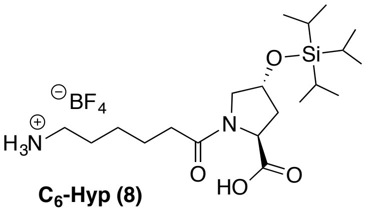

C$_6$-Hyp (8)

(S,S): 72% yield, 21% ee
(R,R): 44% yield, 20% ee

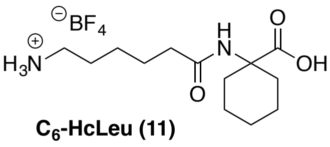

C$_6$-HcLeu (11)

(S,S): 49% yield, 32% ee
(R,R): 50% yield, 32% ee

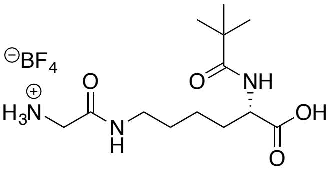

Piv-Lys(Gly) (14)

(S,S): 41% yield, 50% ee
(R,R): 27% yield, 43% ee

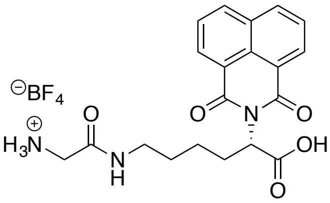

Npha-Lys(Gly) (15)

(S,S): 8% yield, 25% ee
(R,R): 8% yield, 12% ee

Figure S23. Optimization of the amino acid chain in the epoxidation of trans-2-octene.

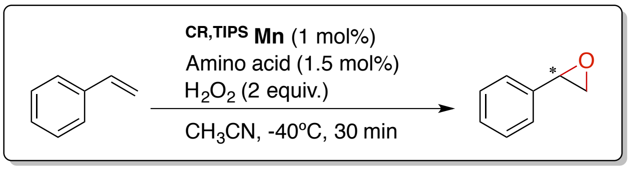

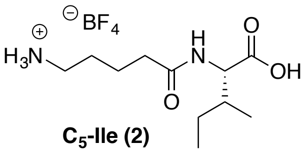

C_5-Ile (2)

(S,S): 29% yield, 43% ee
(R,R): 51% yield, 38% ee

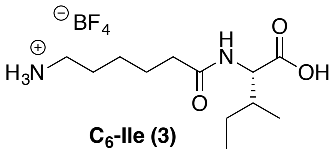

C_6-Ile (3)

(S,S): 64% yield, 48% ee
(R,R): 92% yield, 50% ee

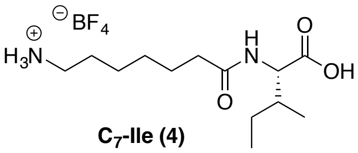

C_7-Ile (4)

(S,S): 52% yield, 34% ee
(R,R): 14% yield, 33% ee

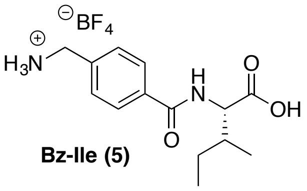

Bz-Ile (5)

(S,S): 13% yield, 49% ee
(R,R): 36% yield, 47% ee

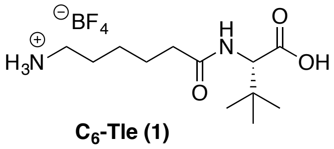

C_6-Tle (1)

(S,S): 48% yield, 23% ee
(R,R): 80% yield, 34% ee

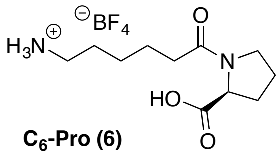

C_6-Pro (6)

(S,S): 33% yield, 27% ee
(R,R): 41% yield, 46% ee

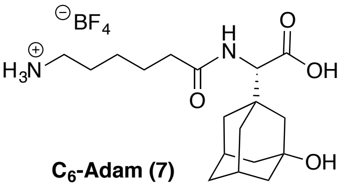

C_6-Adam (7)

(S,S): 36% yield, 38% ee
(R,R): 16% yield, 42% ee

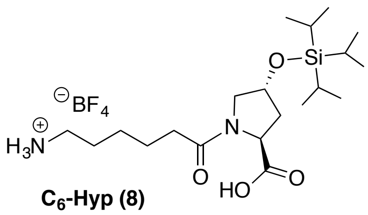

C_6-Hyp (8)

(S,S): 29% yield, 21% ee
(R,R): 33% yield, 44% ee

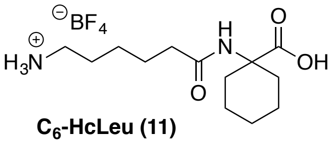

C_6-HcLeu (11)

(S,S): 32% yield, 24% ee
(R,R): 34% yield, 24% ee

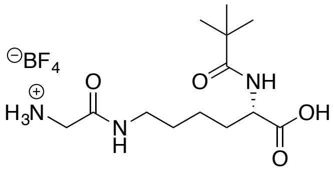

Piv-Lys(Gly) (14)

(S,S): 57% yield, 14% ee
(R,R): 30% yield, 26% ee

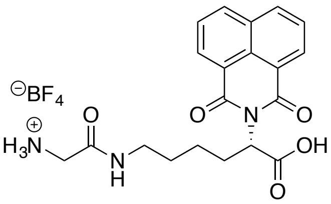

Npha-Lys(Gly) (15)

(S,S): 15% yield, 22% ee
(R,R): 7% yield, 20% ee

Figure S24. Optimization of the amino acid chain in the epoxidation of styrene.

10. Time-course analysis of the epoxidation of S1

These experiments were done following the standard reaction conditions (SI, 7), but quenching the reaction at different times.

10.1. Time-course analysis using *(R,R)*-^{CR,TIPS}Mn and C_6-Tle (1)

Table S11. *Time-course analysis of the epoxidation of 1-chloro-3-methylbut-2-ene* ***(S1)*** *using (R,R)-*^{CR,TIPS}*Mn and* C_6*-Tle* ***(1)****.*

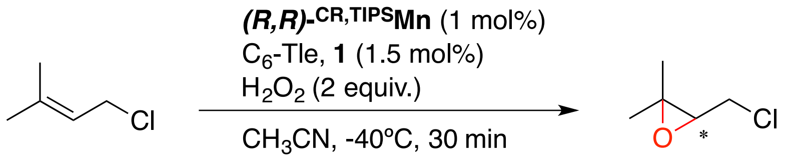

Entry	Time	Conv. (%)[a]	Yield (%)[a]	ee (%)[a]
1	1 min	18	0	-
2	5 min	20	12	60
3	10 min	36	18	60
4	15 min	35	25	61
5	20 min	46	36	63
6	25 min	70	55	67
7	30 min	88	65	67

[a] *Conversions, yields and ee determined by GC. The results are expressed as an average of 2-3 runs with an error <5%.*

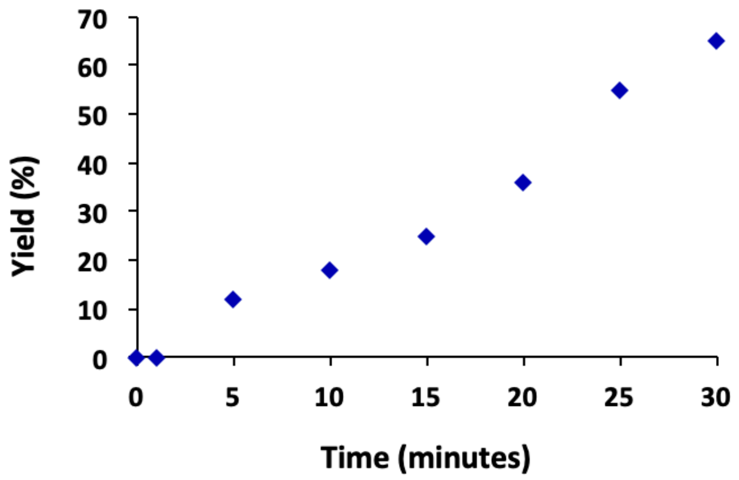

Figure S25. Graphical representation of the yield of the reaction from the data on Table S11.

Mass spectrometry analysis

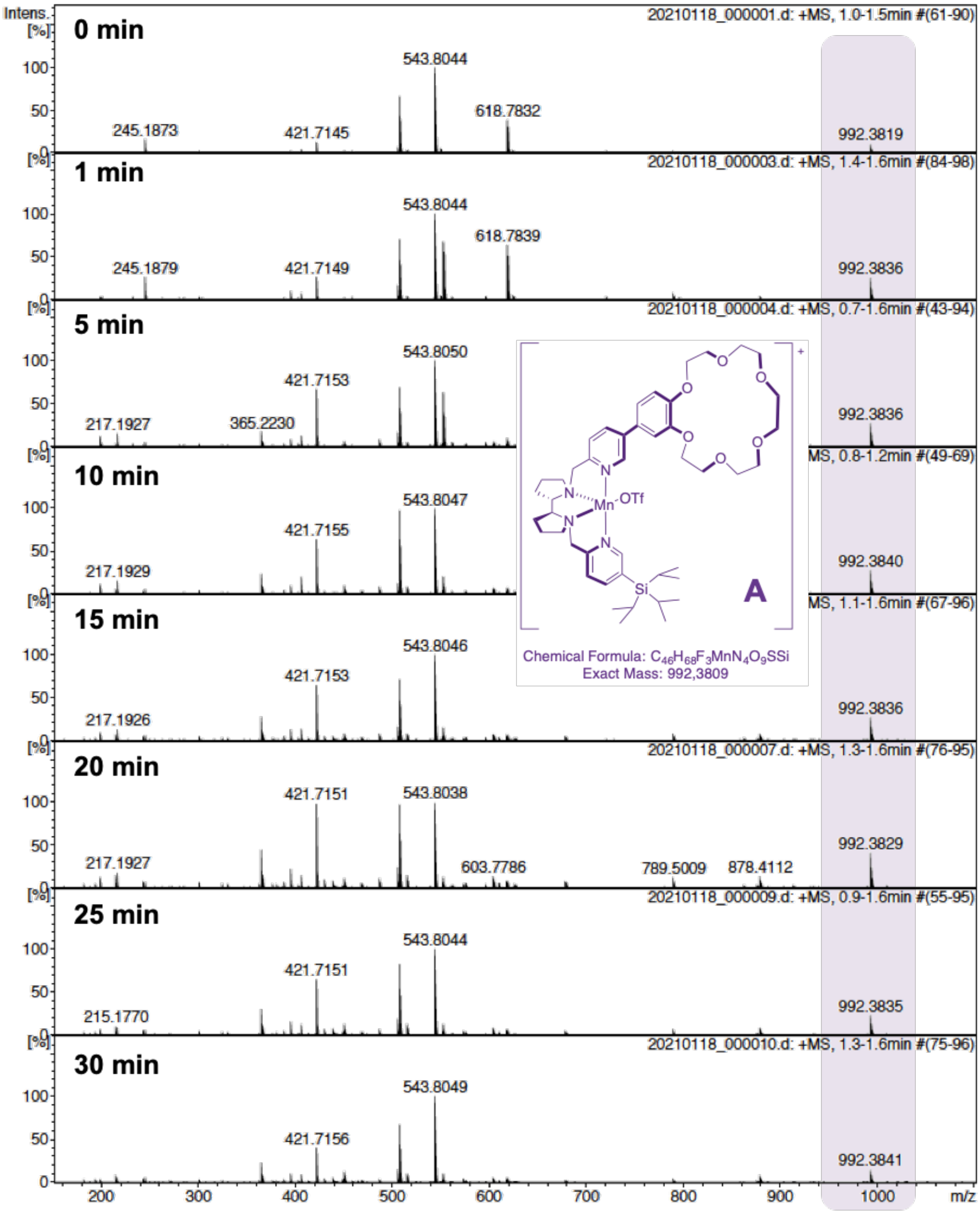

Figure S25. HRMS over time of the reaction mixture of the epoxidation of **S1** using *(R,R)*-^{CR,TIPS}Mn and C_6-Tle **(1)**.

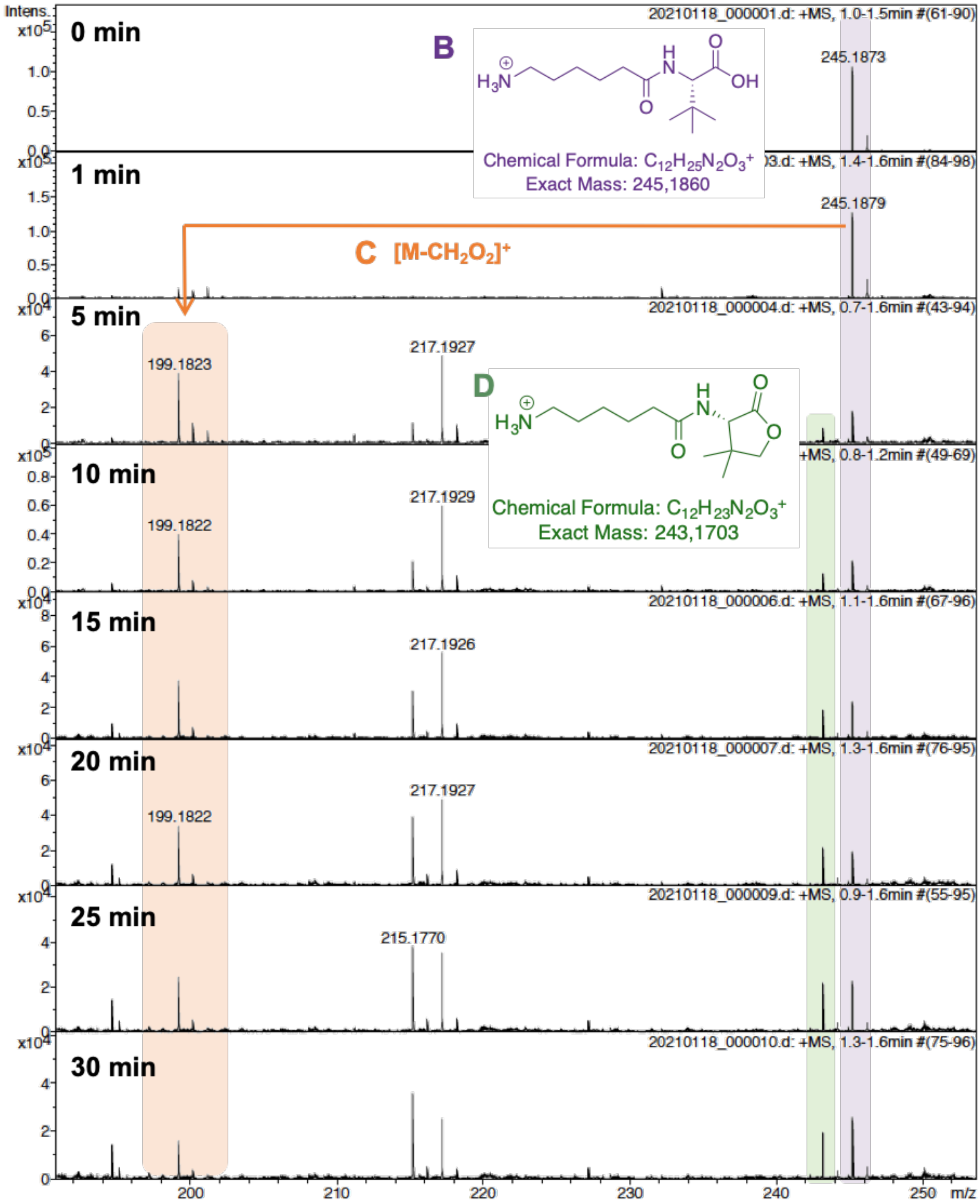

Figure S27. HRMS (from m/z =150 to m/z = 250) over time of the reaction mixture in the epoxidation of **S1** using *(R,R)*-^{CR,TIPS}Mn and C_6-Tle **(1)**.

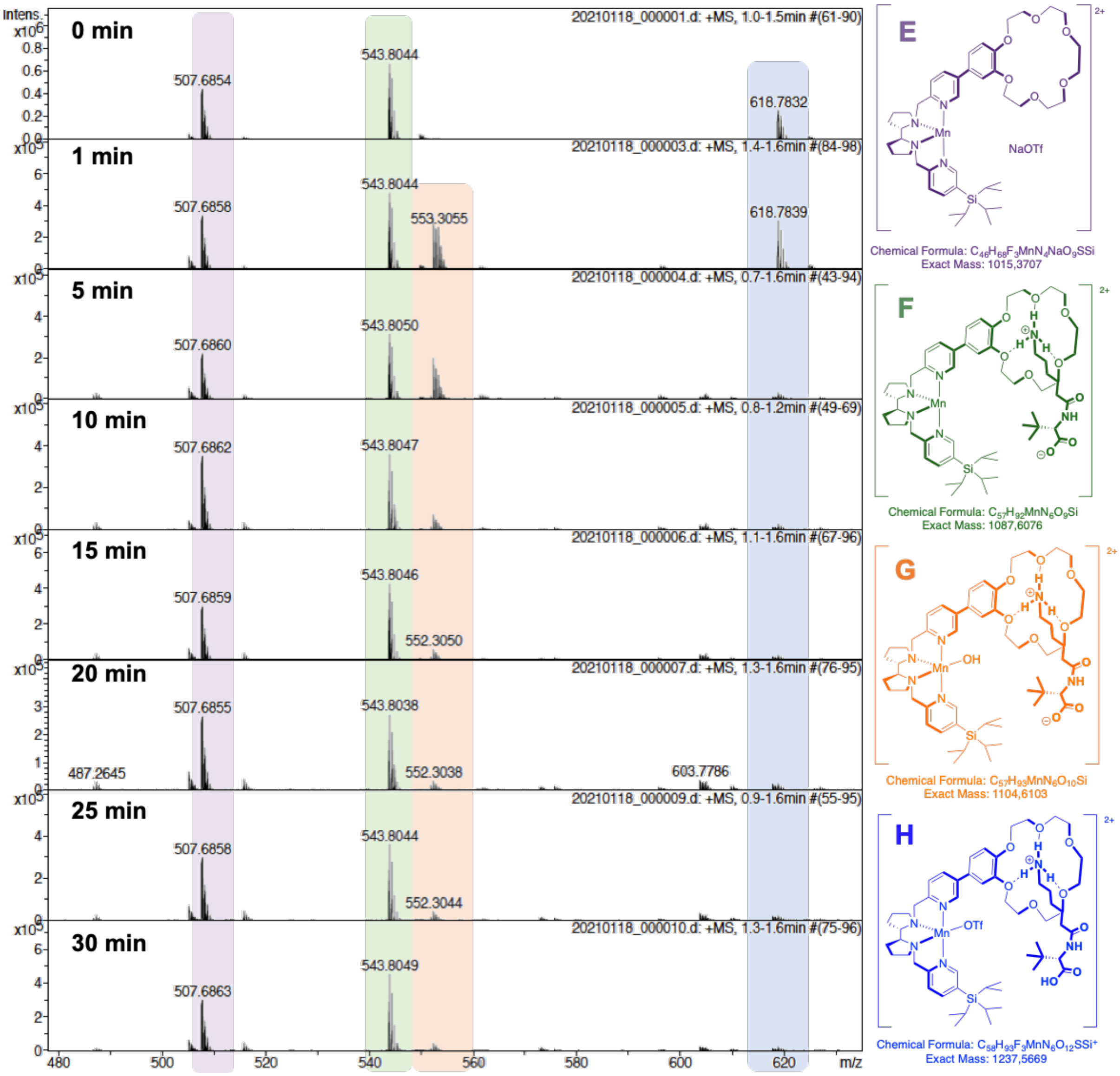

Figure S28. HRMS (from m/z = 480 to m/z = 630) over time of the reaction mixture in the epoxidation *of **S1*** using *(R,R)*-^{CR,TIPS}Mn and C_6-Tle **(1)**.

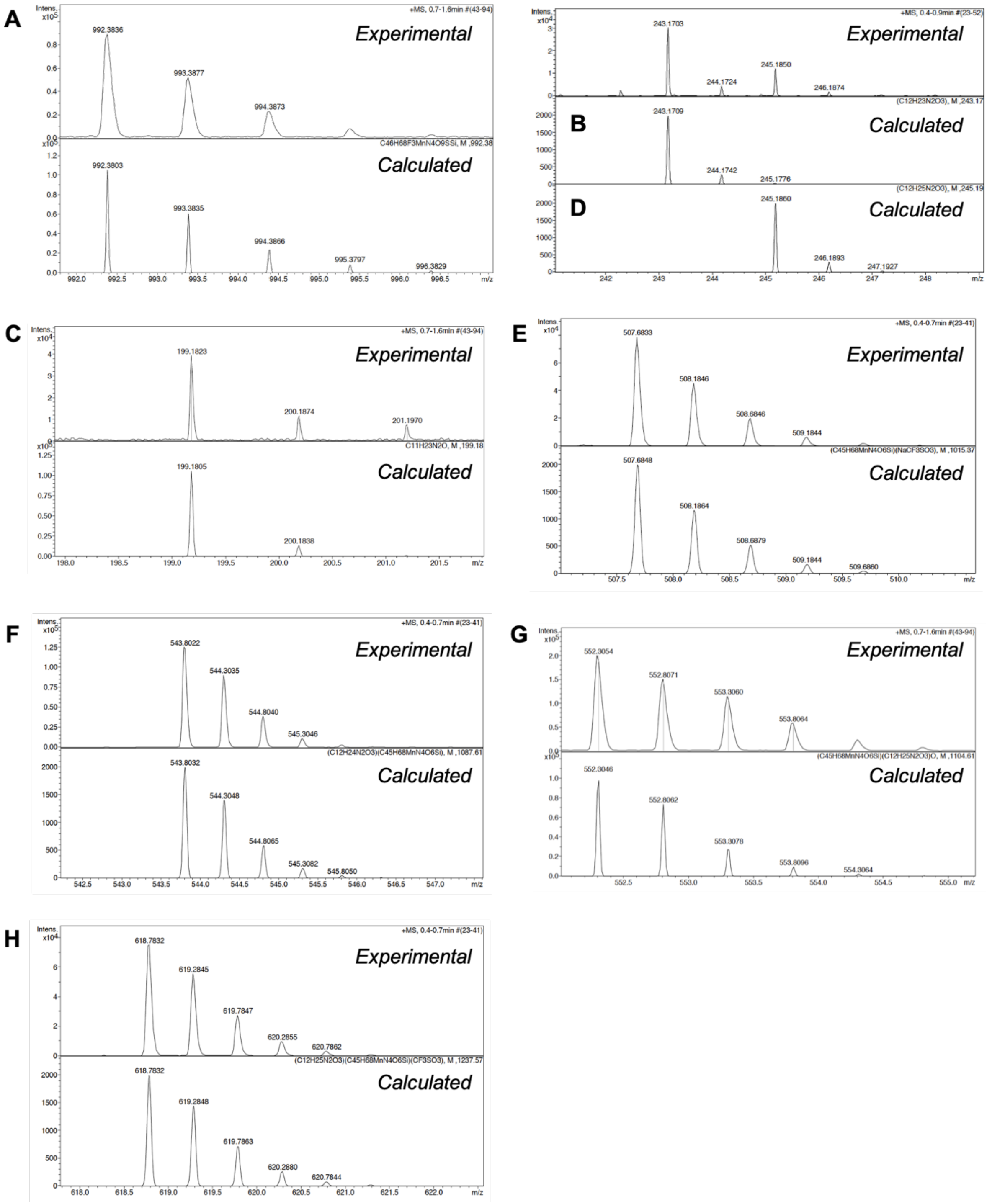

Figure S29. *Isotopic pattern obtained and calculated for the different species identified during the time-course analysis (Figures S26-S28) of the epoxidation of* ***S1*** *using (R,R)-^{CR,TIPS}Mn and C_6-Tle* ***(1)****.*

10.2. Time-course analysis using *(R,R)*-^{CR,TIPS}Mn and C_6-HcLeu (11)

Table S12. *Time-course analysis of the epoxidation of 1-chloro-3-methylbut-2-ene using (R,R)-^{CR,TIPS}Mn and C_6-HcLeu **(11)**.*

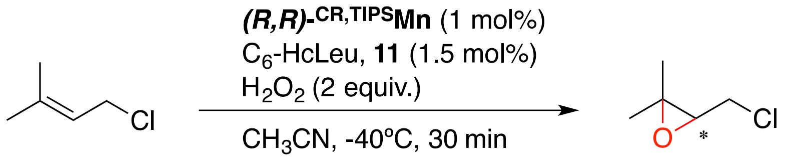

Entry	Time	Conv. (%)[a]	Yield (%)[a]	ee (%)[a]
1	1 min	11	<1	-
2	5 min	32	15	62
3	10 min	44	31	65
4	15 min	55	44	66
5	20 min	76	59	67
6	25 min	95	61	68
7	30 min	99	61	69

[a] *Conversions, yields and ee determined by GC. The results are expressed as an average of 2-3 runs with an error <5%.*

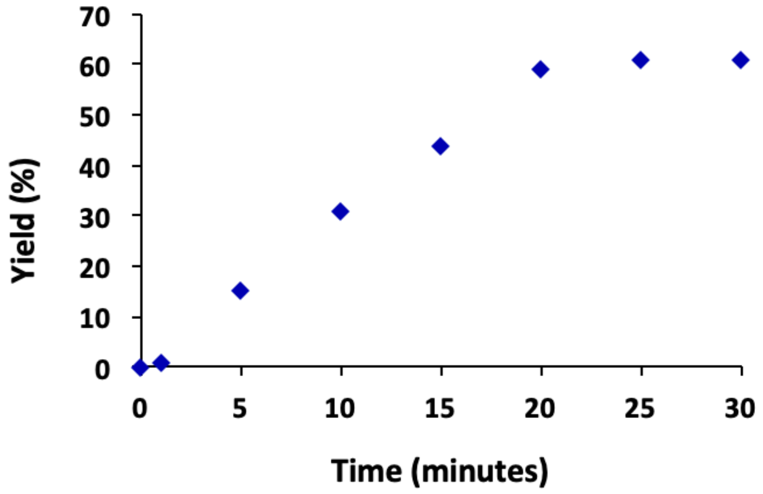

Figure S30. Graphical representation of the yield of the reaction from the data on Table S12.

Mass spectrometry analysis

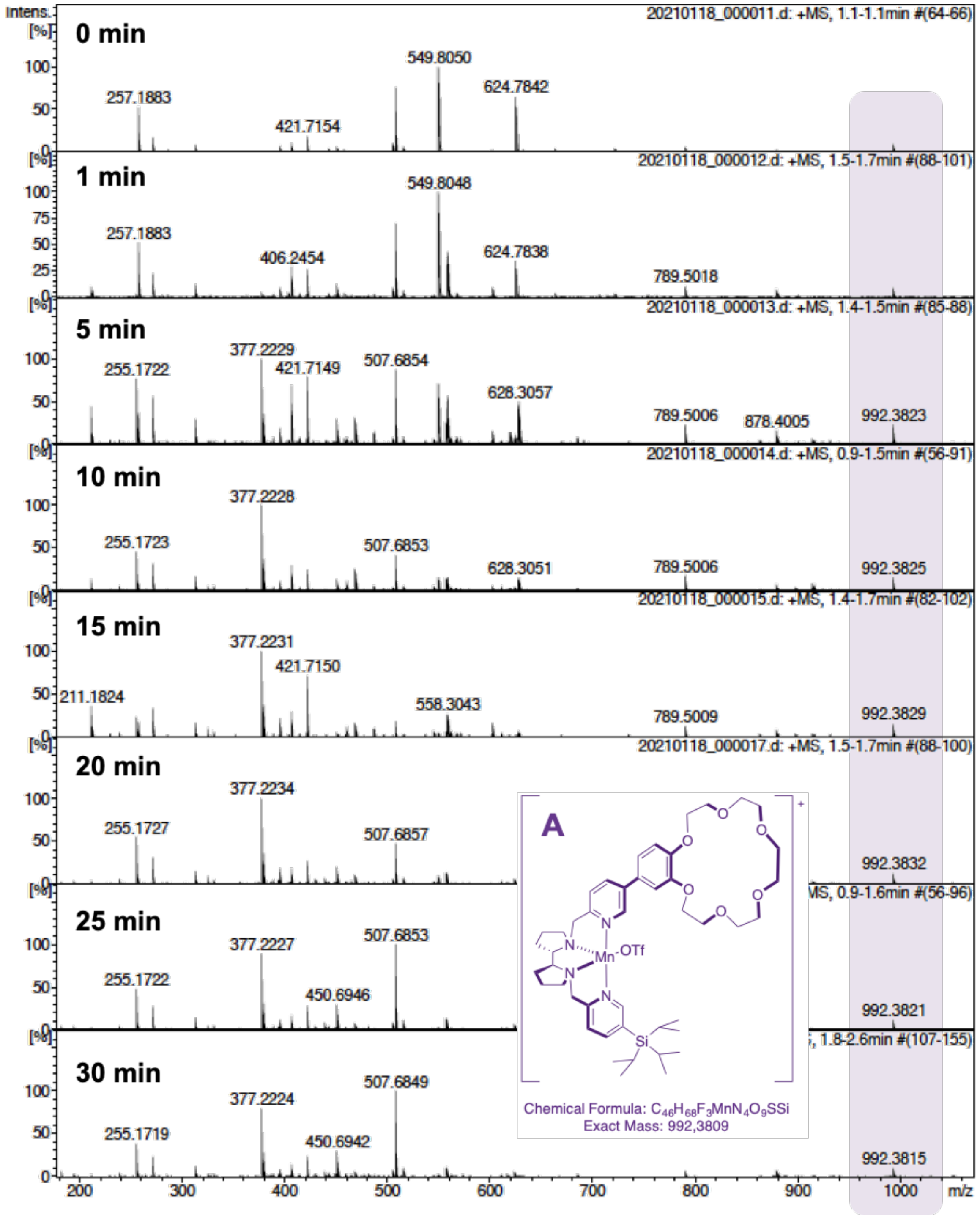

Figure S31. HRMS over time of the reaction mixture of the epoxidation of **S1** using *(R,R)*-^{CR,TIPS}Mn and C_6-HcLeu **(11)**.

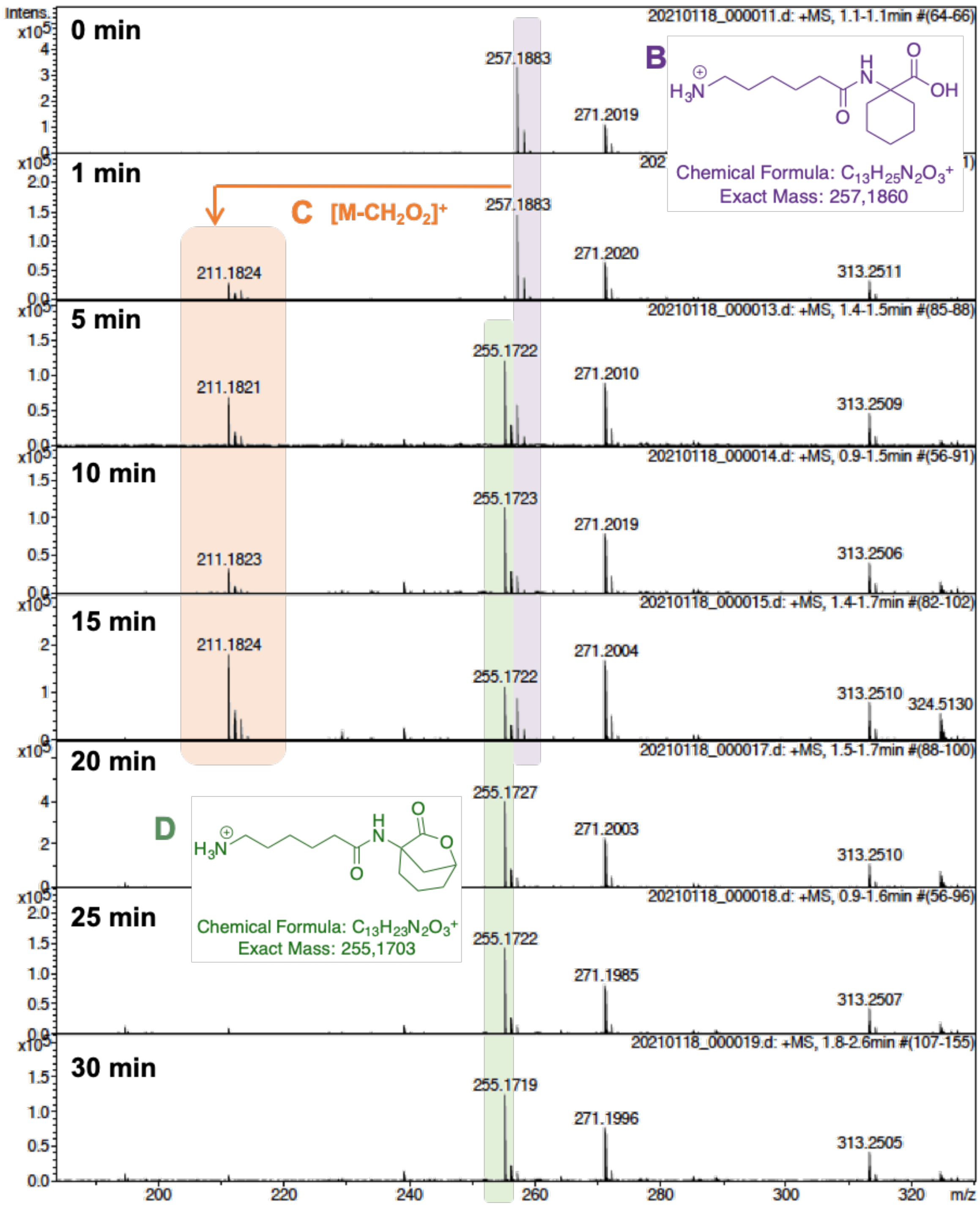

Figure S32. HRMS (from m/z =190 to m/z = 320) over time of the reaction mixture in the epoxidation of **S1** using *(R,R)*-^{CR,TIPS}Mn and C_6-HcLeu **(11)**.

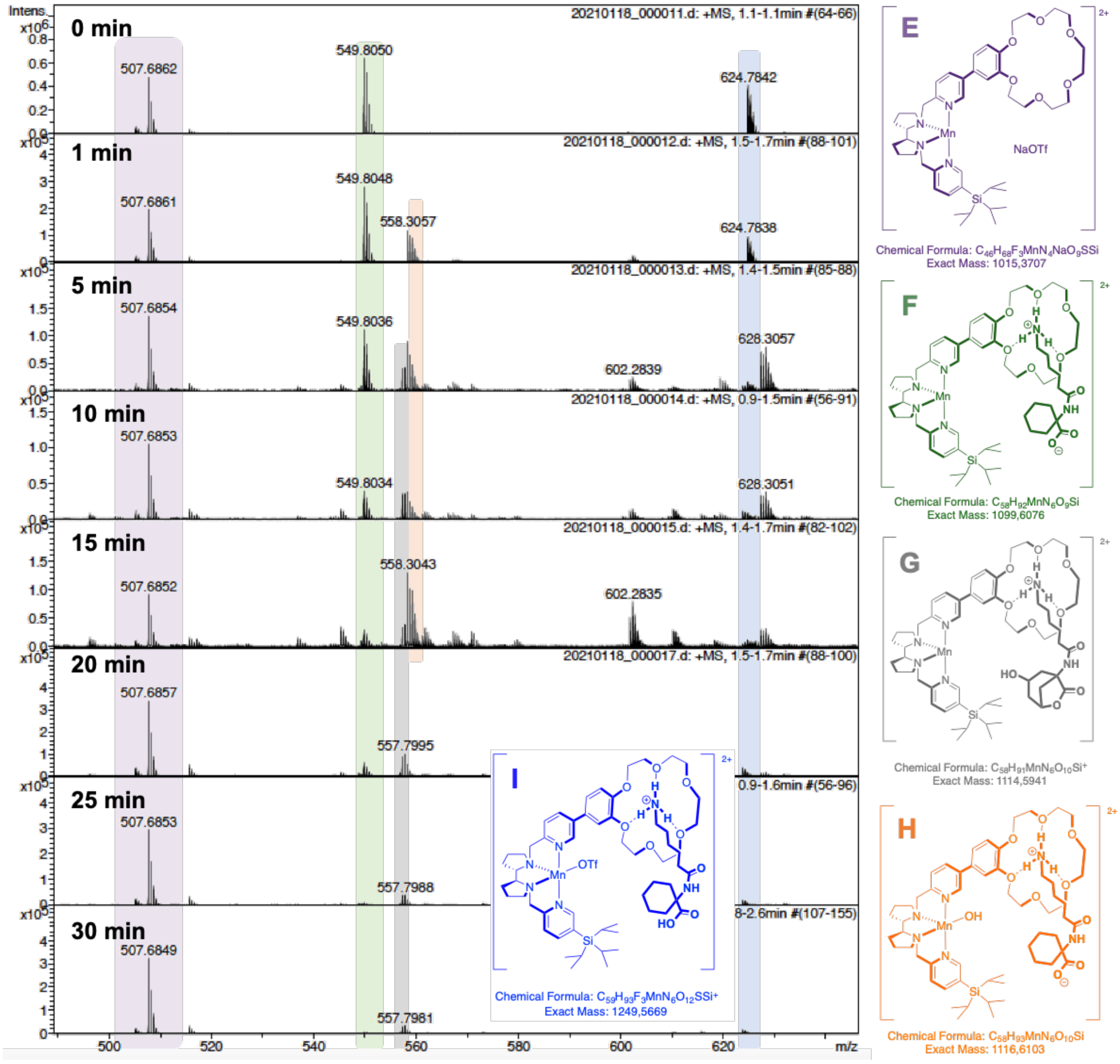

Figure S33. HRMS (from m/z = 490 to m/z = 640) over time of the reaction mixture in the epoxidation *of* **S1** using *(R,R)*-^{CR,TIPS}Mn and C_6-HcLeu **(11)**.

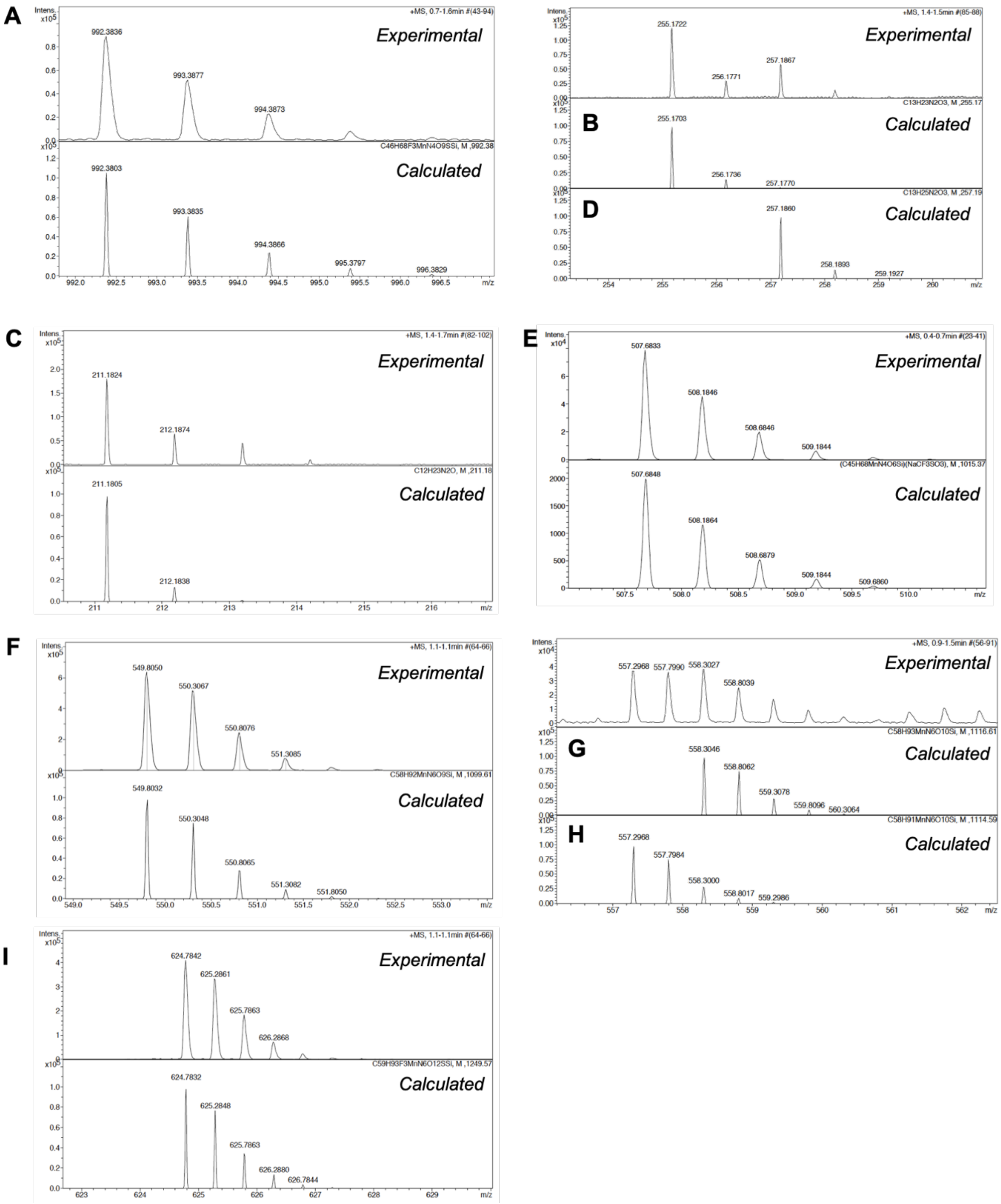

Figure S34. Isotopic pattern obtained and calculated for the different species identified during the time-course analysis (Figures S31-S33) of the epoxidation of **S1** using *(R,R)*-^{CR,TIPS}Mn and C_6-HcLeu **(11)**.

11. Substrate scope

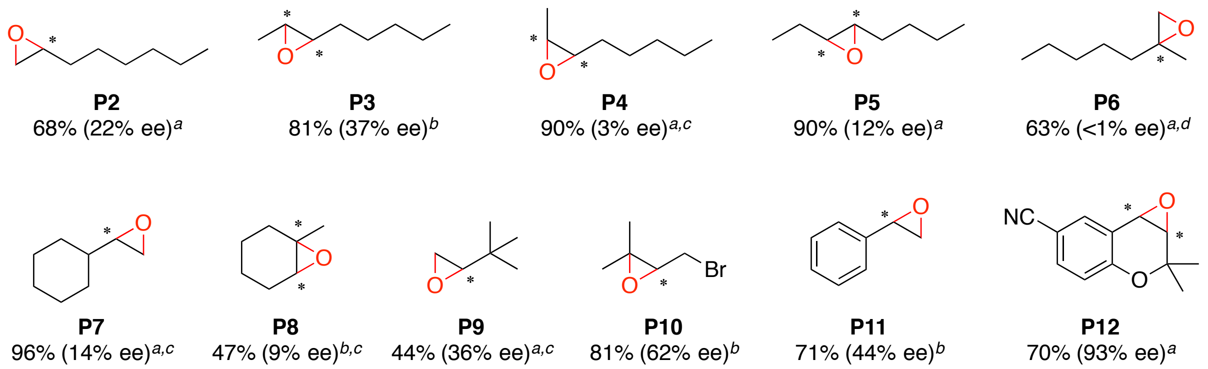

Figure S35. *Epoxidation of aliphatic olefins by supramolecular system* ^{CR,TIPS}*Mn/**1**.* *<u>Reaction conditions</u>: Substrate (22 μmol, 1 equiv.), catalyst (0.22 μmol, 1 mol%), amino acid (0.33 μmol, 1.5 mol%),* H_2O_2 *(44 μmol, 2 equiv.),* CH_3CN *(200 μL, 0.1M), -40°C, 30 min. Yields and ee analyzed by GC.* [a] *(S,S)-*^{CR,TIPS}*Mn as catalyst.* [b] *(R,R)-*^{CR,TIPS}*Mn as catalyst.* [c] ***3** was used instead of **1**.* [d] ***11** was used instead of **1**.*

11.1. Synthesis and characterization of racemic epoxides

Styrene oxide (**P11**) was purchased from Sigma-Aldrich. The synthesis of non-commercially available racemic epoxides was done following a slightly modified reported procedure.[33]

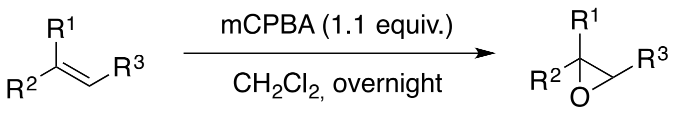

The olefin (1 equiv.) was solved in CH_2Cl_2 (0.5M) and cooled to 0°C with an ice bath. Then, 1.1 equiv. of mCPBA were added in different portions and the mixture was stirred at room temperature overnight. After this time, the solution was washed with 2M NaOH (x2) and 1M HCl (x2). The combined organic layers were dried over anhydrous $MgSO_4$, filtered and the solvent was removed under reduced pressure.

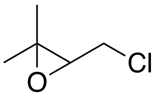

3-(chloromethyl)-2,2-dimethyloxirane (P1). Prepared from 1-chloro-3-methylbut-2-ene (1 mL, 8.43 mmol) according to the general procedure. ^{1}H-NMR (400 MHz, $CDCl_3$) δ, ppm: 3.70 (dd, *J* = 11.5, 6.0 Hz, 1H), 3.49 (dd, *J* = 11.5, 7.0 Hz, 1H), 3.07 (dd, *J* = 7.0, 6.0 Hz, 1H), 1.39 (s, 3H), 1.36 (s, 3H). ^{13}C-NMR (100 MHz, $CDCl_3$) δ, ppm: 62.3, 59.8, 42.3, 24.5, 18.4.

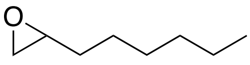 **2-hexyloxirane (P2)**. Prepared from 1-octene (254.1 mg, 2.24 mmol) according to the general procedure. Spectral data match those reported in literature.[34] ^{1}H-NMR (400 MHz, $CDCl_3$) δ, ppm: 2.93 (tdd, *J* = 5.6, 4.0, 2.7 Hz, 1H), 2.76 (dd, *J* = 5.0, 4.0 Hz, 1H), 2.48 (dd, *J* = 5.0, 2.7 Hz, 1H), 1.58 – 1.51 (m, 2H), 1.51 – 1.43 (m, 2H), 1.42 – 1.23 (m, 6H), 0.90 (t, *J* = 6.8 Hz, 3H). ^{13}C-NMR (100 MHz, $CDCl_3$) δ, ppm: 52.4, 47.1, 32.5, 31.8, 29.1, 25.9, 22.6, 14.0.

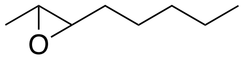 **2-methyl-3-pentyloxirane (P3)**. Prepared from trans-2-octene (187.2 mg, 1.63 mmol) according to the general procedure. Spectral data match those reported in literature.[35] ^{1}H-NMR (400 MHz, $CDCl_3$) δ, ppm: 2.77 (qd, *J* = 5.3, 2.3 Hz, 1H), 2.65 (td, *J* = 5.5, 2.3 Hz, 1H), 1.56 – 1.48 (m, 2H), 1.47 – 1.36 (m, 2H), 1.35 – 1.27 (m, 7H), 0.89 (t, *J* = 7.0 Hz, 3H). ^{13}C-NMR (100 MHz, $CDCl_3$) δ, ppm: 60.1, 54.8, 31.9, 31.6, 25.6, 22.6, 17.6, 14.0.

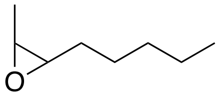 **2-methy-3-pentyloxirane (P4)**. Prepared from cis-2-octene (120.5 mg, 1.07 mmol) according to the general procedure. Spectral data match those reported in literature.[35] ^{1}H-NMR (400 MHz, $CDCl_3$) δ, ppm: 3.11 – 3.00 (m, 1H), 2.94 – 2.86 (m, 1H), 1.53 – 1.28 (m, 8H), 1.32 (d, *J* = 5.4 Hz, 3H), 0.91 (t, *J* = 7.0 Hz, 3H). ^{13}C-NMR (100 MHz, $CDCl_3$) δ, ppm: 57.0, 52.4, 31.7, 27.5, 26.0, 22.4, 14.1, 13.2.

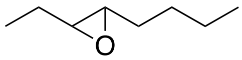 **2-butyl-3-ethyloxirane (P5)**. Prepared from trans-3-octene (159.8 mg, 1.38 mmol) according to the general procedure. ^{1}H-NMR (400 MHz, $CDCl_3$) δ, ppm: 2.72 – 2.59 (m, 2H), 1.58 – 1.48 (m, 4H), 1.43 – 1.32 (m, 4H), 0.97 (t, *J* = 7.5 Hz, 3H), 0.89 (t, *J* = 7.1 Hz, 4H). ^{13}C-NMR (100 MHz, $CDCl_3$) δ, ppm: 60.1, 58.8, 31.7, 28.1, 25.1, 22.5, 13.9, 9.8.

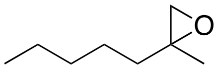 **2-methy-2-pentyloxirane (P6)**. Prepared from 2-methylhept-1-ene (155.8 mg, 1.37 mmol) according to the general procedure. Spectral data match those reported in literature.[36] ^{1}H-NMR (400 MHz, $CDCl_3$) δ, ppm: 2.63 (d, *J* = 4.9 Hz, 1H), 2.59 (d, *J* = 4.9 Hz, 1H), 1.65 – 1.56 (m, 1H), 1.54 – 1.46 (m, 1H), 1.47 – 1.36 (m, 2H), 1.37 – 1.24 (m, 4H), 1.32 (s, 3H), 0.90 (t, *J* = 6.8 Hz, 3H). ^{13}C-NMR (100 MHz, $CDCl_3$) δ, ppm: 57.2, 54.0, 36.7, 31.8, 24.9, 22.6, 20.9, 14.0.

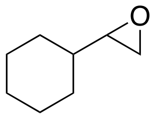

2-cyclohexyloxirane (P7). Prepared from vinylcyclohexane (105.6 mg, 0.930 mmol) according to the general procedure. Spectral data match those reported in literature.[35] ^{1}H-NMR (400 MHz, $CDCl_3$) δ, ppm: 2.77 – 2.68 (m, 2H), 2.59 – 2.51 (m, 1H), 1.94 – 1.83 (m, 1H), 1.82 – 1.73 (m, 2H), 1.73 – 1.61 (m, 2H), 1.30 – 1.03 (m, 6H). ^{13}C-NMR (100 MHz, $CDCl_3$) δ, ppm: 56.7, 46.0, 40.4, 29.7, 28.8, 26.3, 25.7, 25.5.

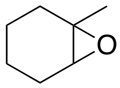

1-methyl-7-oxabicyclo[4.1.0]heptane (P8). Prepared from 1-methylyclohex-1-ene (165.4 mg, 424.0 mmol) according to the general procedure. Spectral data match those reported in literature.[37] ^{1}H-NMR (400 MHz, $CDCl_3$) δ, ppm: 3.00 (d, *J* = 3.7 Hz, 1H), 1.94 – 1.84 (m, 3H), 1.71 – 1.63 (m, 1H), 1.47 – 1.37 (m, 2H), 1.31 (s, 3H), 1.30 – 1.15 (2H). ^{13}C-NMR (100 MHz, $CDCl_3$) δ, ppm: 59.8, 58.0, 298, 24.7, 23.9 20.0, 19.6.

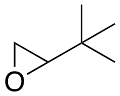

2-(*tert*-butyl)oxirane (P9). Prepared from 3,3,-dimethylbut-1-ene (340.7 mg, 3.92 mmol) according to the general procedure. Spectral data match those reported in literature.[38] ^{1}H-NMR (400 MHz, $CDCl_3$) δ, ppm: 2.71 – 2.62 (m, 1H), 2.49 – 2.41 (m, 2H), 0.99 (s, 9H)- ^{13}C-NMR (100 MHz, $CDCl_3$) δ, ppm: 59.8, 43.7, 29.7, 24.3.

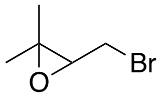

3-(bromomethyl)-2,2-dimethyloxirane (P10). Prepared from 1-bromo-3-methylbut-2-ene (156.8 mg, 1.05 mmol) according to the general procedure. Spectral data match those reported in literature.[39] ^{1}H-NMR (400 MHz, $CDCl_3$) δ, ppm: 3.54 (ddd, *J* = 10.5, 6.0, 0.8 Hz, 1H), 3.27 (ddd, *J* = 10.4, 7.6, 0.7 Hz, 1H), 3.11 (ddd, *J* = 7.3, 6.0, 1.0 Hz, 1H), 1.38 (s, 3H), 1.34 (s, 3H). ^{13}C-NMR (100 MHz, $CDCl_3$) δ, ppm: 62.2, 60.7, 29.8, 24.5, 18.2.

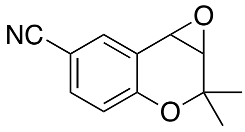

2,2-dimethyl-1a,7b-dihydro-2*H*-oxireno[2,3-c]-chromene-6-carbonitrile (P12). Prepared from 2,2-dimethyl-2*H*-chromene-6-carbonitrile (102.0 mg, 0.534 mmol) according to the general procedure. Spectral data match those reported in literature.[7] ^{1}H-NMR (400 MHz, $CDCl_3$) δ, ppm: 7.65 (d, *J* = 2.1 Hz, 1H), 7.52 (dd, *J* = 8.5, 2.1 Hz, 1H), 6.87 (d, *J* = 8.5 Hz, 1H), 3.92 (d, *J* = 4.0 Hz, 1H), 3.55 (d, *J* = 4.0 Hz, 1H), 1.60 (s, 3H), 1.30 (s, 3H). ^{13}C-NMR (100 MHz, $CDCl_3$) δ, ppm: 156.5, 134.4, 133.8, 121.1, 119.0, 118.7, 104.2, 74.7, 62.3, 49.9, 25.5, 23.0.

11.2. Epoxidation of geranyl acetate

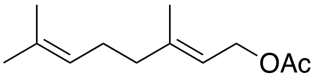

Geranyl acetate (**S13**) was prepared according to a reported procedure.[40] Spectral data match those reported in literature. ^{1}H-NMR (400 MHz, $CDCl_3$) δ, ppm: 5.36 (tq, *J* = 7.2, 1.4 Hz, 1H), 5.19 – 5.02 (m, 1H), 4.61 (d, *J* = 7.1 Hz, 2H), 2.19 – 1.99 (m, 4H), 2.07 (s, 3H), 1.72 (d, *J* = 1.3 Hz, 3H), 1.70 (d, *J* = 1.4 Hz, 4H), 1.62 (s, 3H). ^{13}C-NMR (100 MHz, $CDCl_3$) δ, ppm: 171.1, 142.3, 131.8, 123.7, 118.2, 61.4, 39.5, 26.3, 25.7, 21.1, 17.7, 16.5.

Identification of the products

Epoxidation of geranyl acetate was done following a procedure reported in the literature.[41] The products observed in the GC could be identified by comparing the yields obtained with those reported. Assignation was confirmed by GC-MS analysis of each product.

Table S13. *Selectivity obtained in the epoxidation of geranyl acetate (**S2**) using Mn(II)/Picolinic acid catalyst system.*

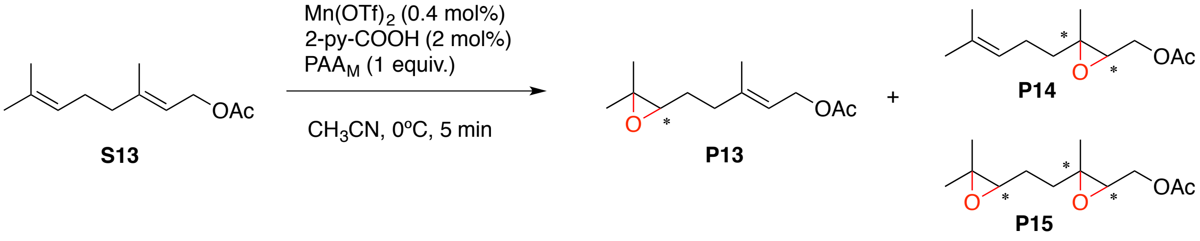

Entry		Yield P2 (%)[a]	Yield P3 (%)[a]	Yield P4 (%)[a]
1	Obtained	78	6	16
2	Reported	67	6	27

[a] *Normalized yields (·100)*

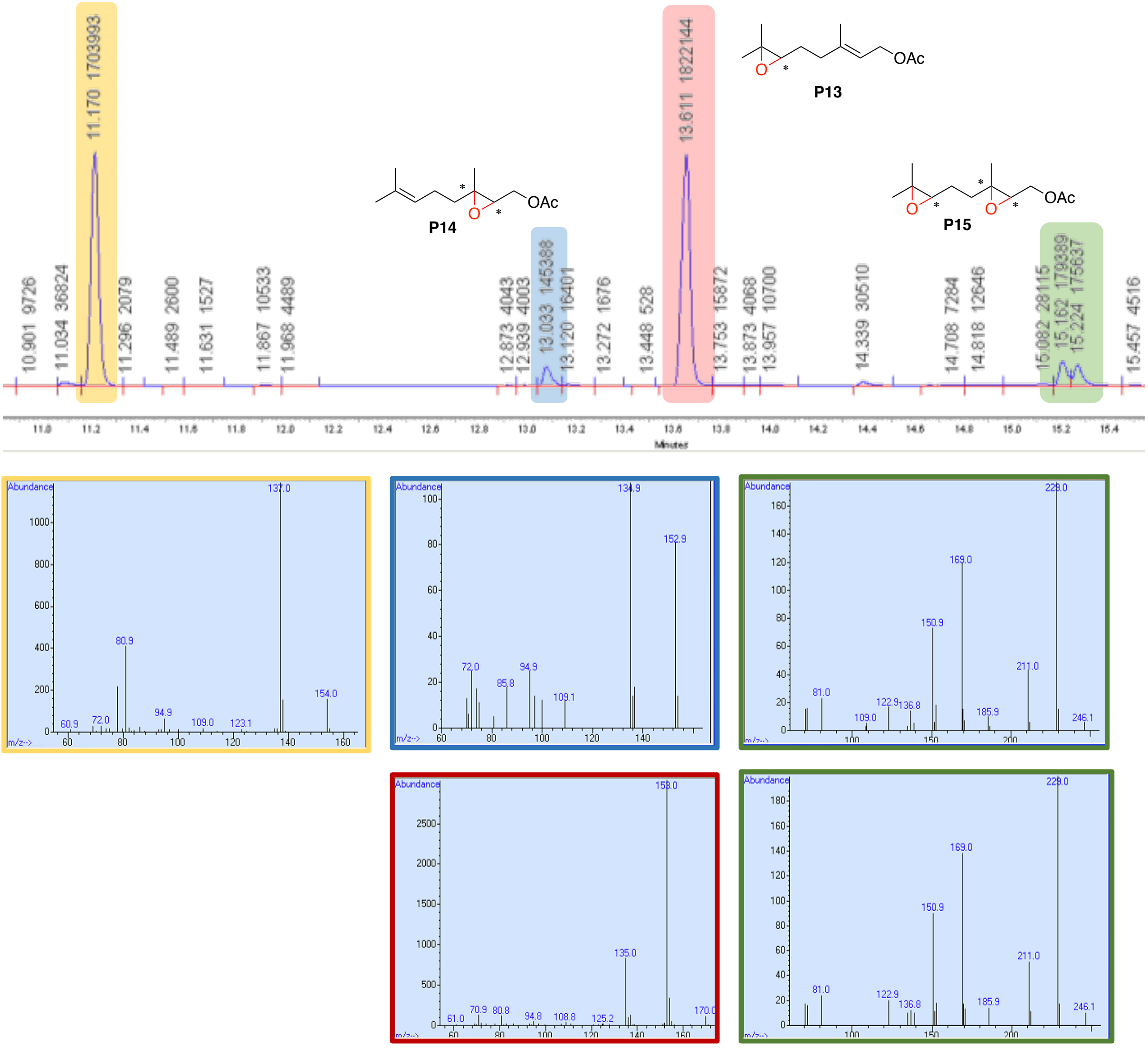

Figure S36. GC spectrum of the reaction crude (top) and GC-MS spectrum for each compound (bottom).

Catalytic experiments

Table S14. *Epoxidation of geranyl acetate (**S13**).*

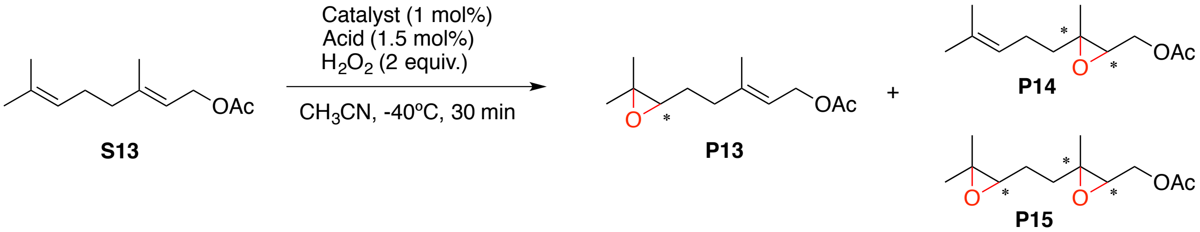

Entry	Catalyst	Acid	Conv. (%)[a]	Yield P13 (%) [ee, %][a]	Yield P14 (%)[a]	Yield P15 (%)[a]
1	*(R,R)*-CR,TIPSMn	C_6-Tle (**1**)	**68**	**55 [38% ee]**	**4**	-
2	*(R,R)*-CR,TIPSMn	C_6-HcLeu (**11**)	97	77 [26% ee]	-	13
3	*(S,S)*-CR,TIPSMn	C_6-Tle (**1**)	75	61 [32 % ee]	4	-
4	*(S,S)*-CR,Me2NMn	C_6-Tle (**1**)	93	67 [26% ee]	-	19
5	*(R,R)*-TIPSMn	C_6-Tle (**1**)	10	4 [13% ee]	1	-
6[b]	*(R,R)*-TIPSMn	Ac-Tle-OH	20	12 [12% ee]	2	-
7[b]	*(S,S)*-TIPSMn	Ac-Tle-OH	30	25 [20% ee]	3	-

*<u>Reaction conditions</u>: **S13** (22 μmol, 1 equiv.), catalyst (0.22 μmol, 1 mol%), acid (0.33 μmol, 1.5 mol%), H_2O_2 (44 μmol, 2 equiv.), CH_3CN (200 μL, 0.1M), -40°C, 30 min.* [a] *Conversions, yields and ee analyzed by GC. The results are expressed as an average of 2-3 runs with an error <5%.* [b] *15 equiv. of acid were used.*

<u>Discussion</u>:

The best supramolecular systems were applied in the epoxidation of a natural product derivative, geranyl acetate **S13**. This molecule contains two C=C bonds that compete for epoxidation, differing in their electronic properties. Irrespective of catalyst and reaction conditions, we observed that the most electron-rich, remote double bond is preferentially oxidized to furnish **P13**, consistently with the electrophilic character of the oxidizing species.[42, 43] **P13** is interesting because known directed epoxidation methods typically operate on the proximal C=C bond of geraniol derivatives with high ee (>90%) (for examples in allylic alcohols[44-47] or amides,[48, 49]), but very few methods to epoxidize the terminal olefin in moderate to good enantioselectivities have been reported so far.[47, 50, 51]

The two best combinations of catalyst and amino acid chain were tested (Table S14, entries 1-2), with the highest enantioselectivity (38% ee) achieved with *(R,R)*-CR,TIPSMn and **1**. Comparable yields and slightly lower enantioselectivity were obtained with **1** and the enantiomeric *(S,S)*-CR,TIPSMn catalyst (entry 3) or *(S,S)*-CR,Me2NMn (Entry 4). It is interesting to note that *(R,R)*-CR,TIPSMn/**11** and *(S,S)*-CR,Me2NMn/**1** systems (entries 2 and 4 respectively) furnished higher yields of remote epoxide **P13** together with considerable amount of diepoxide **P15**, suggesting the formation of more active catalytic systems.

While yields and selectivities obtained with the supramolecular catalyst are still far from satisfactory from a synthetic perspective, the comparison with the results of a non-supramolecular catalysts highlight the benefits of the former; oxidation of geranyl acetate **S13** with the non-supramolecular catalyst *(R,R)*-^{TIPS}Mn again provided a very modest 4% yield of the remote epoxide **P13** in a much lower enantioselectivity (13% ee, Entry 5). Higher amounts of amino acid co-catalyst (15 equiv.) permit to increase product yield only to 30% and ee's up to 20% (entries 6-7), still far from the outcome of the supramolecular system.

12. ^{1}H and ^{13}C{H} NMR spectra of the ligands and complexes

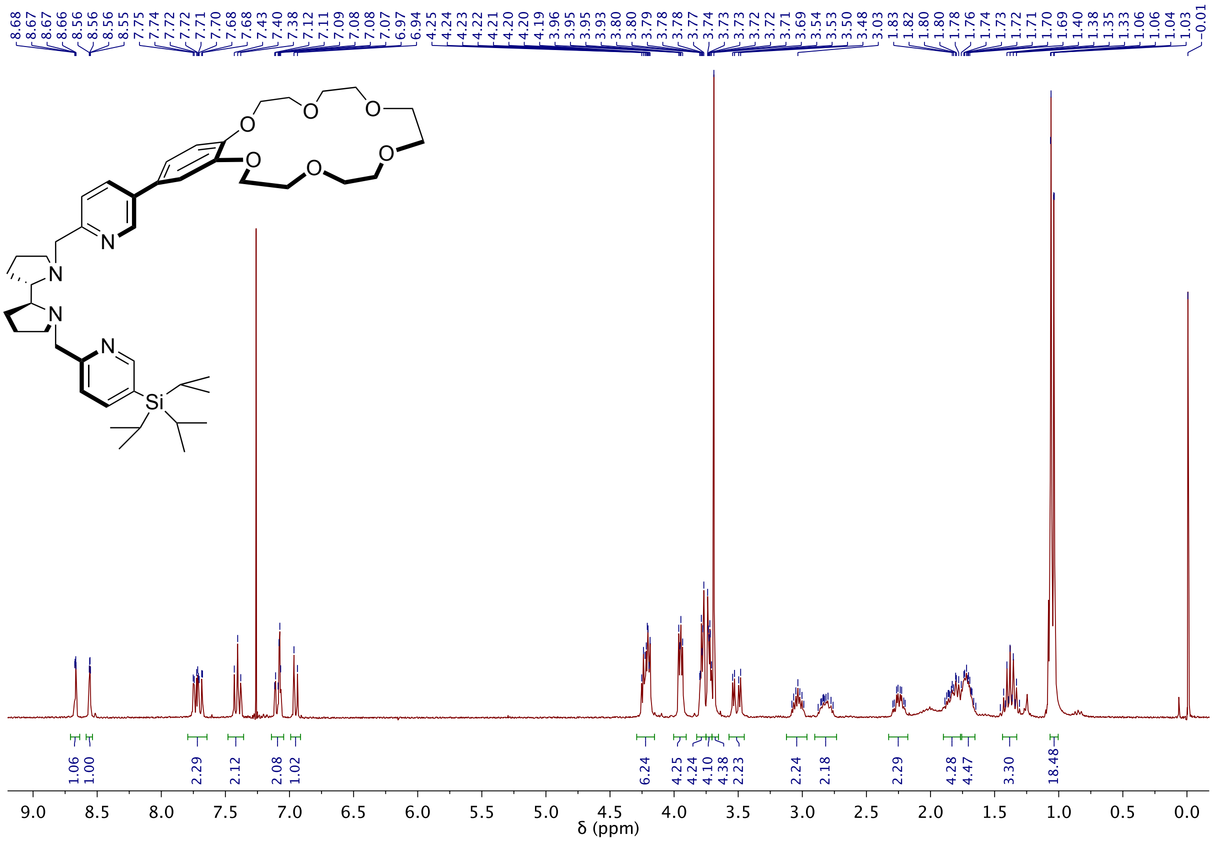

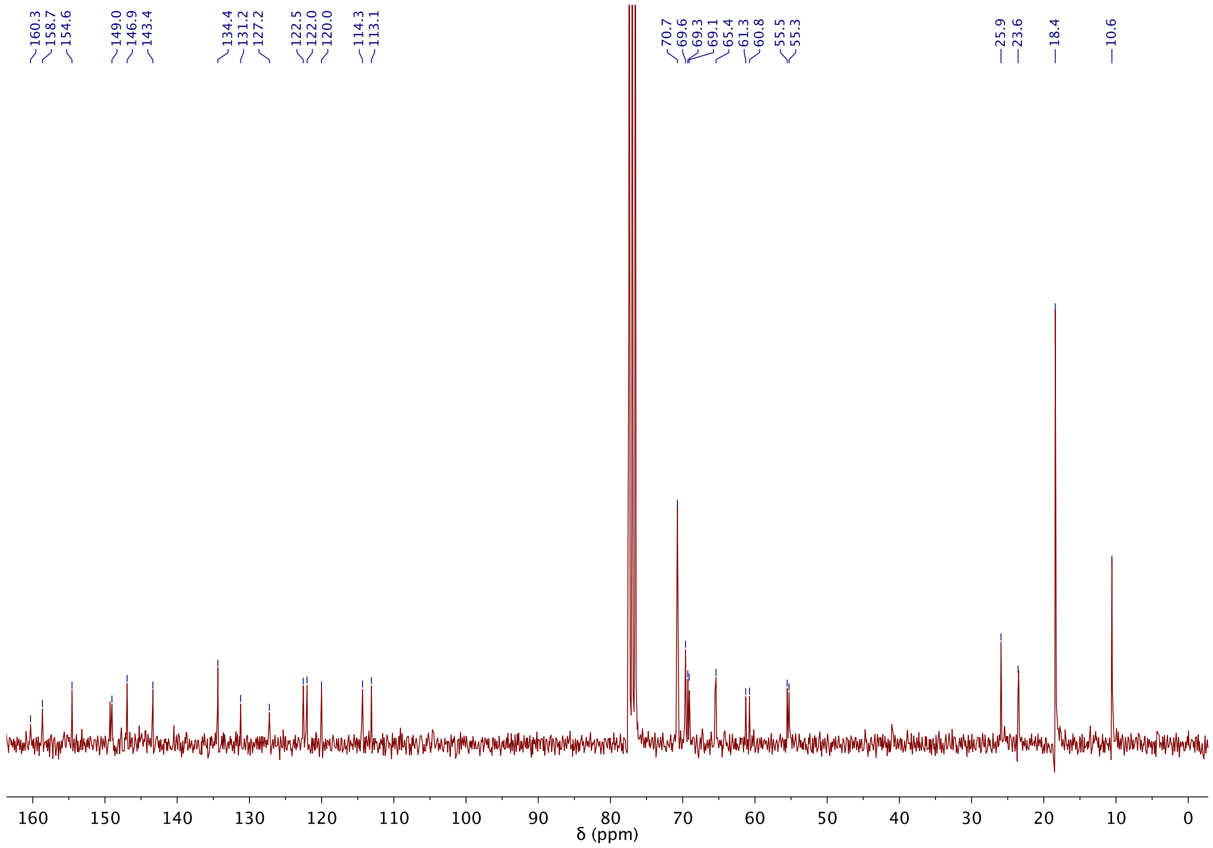

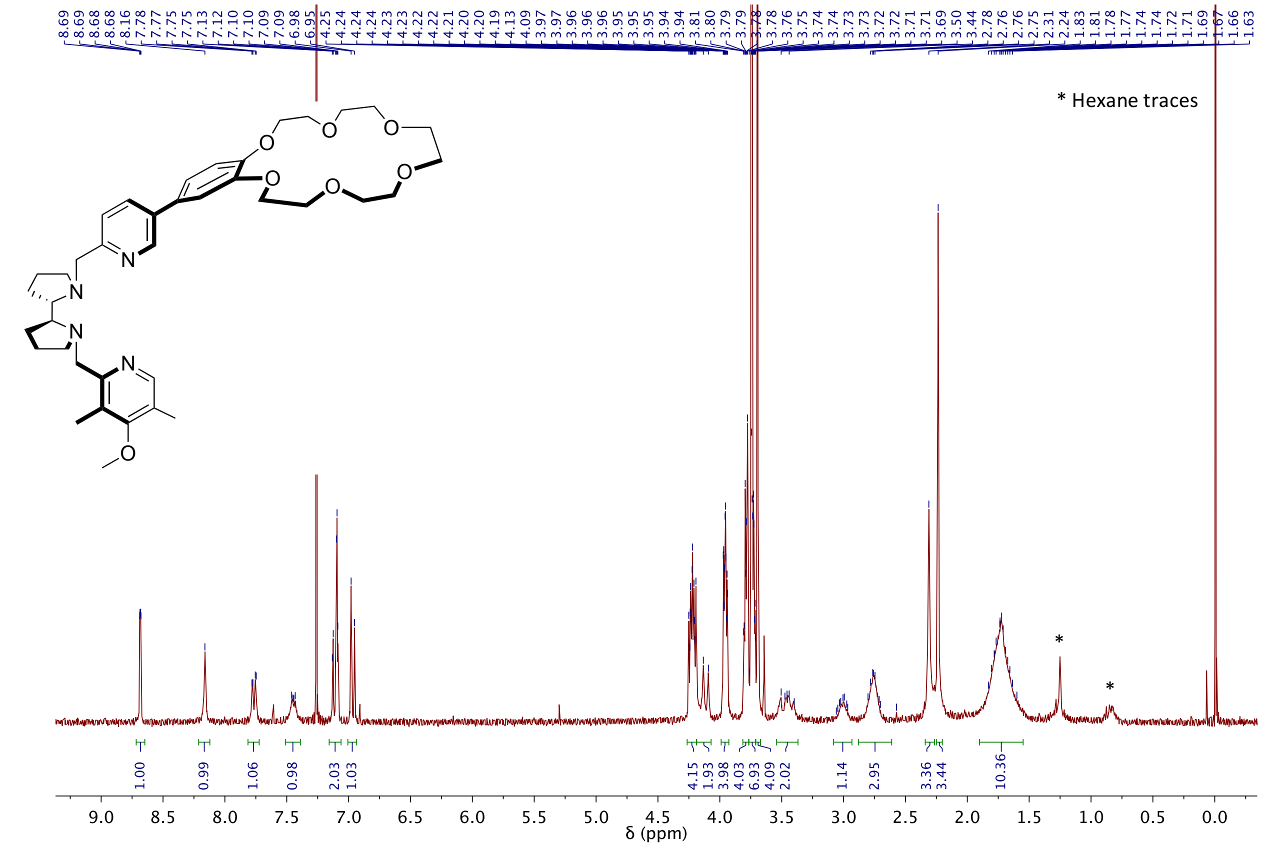
8.69 8.69 8.68 8.68 8.16 7.78 7.77 7.75 7.75 7.13 7.12 7.10 7.10 7.09 6.98 6.95 4.25 4.24 4.24 4.23 4.23 4.22 4.21 4.20 4.20 4.19 4.13 4.09 3.97 3.97 3.96 3.96 3.95 3.95 3.95 3.94 3.81 3.80 3.79 3.79 3.78 3.76 3.75 3.74 3.74 3.73 3.73 3.72 3.72 3.71 3.71 3.69 3.50 3.44 2.78 2.76 2.76 2.75 2.31 2.24 1.83 1.81 1.78 1.77 1.74 1.72 1.71 1.69 1.67 1.66 1.63
* Hexane traces
O
O
O
O
O
O
N
N
N
N
O
1.00 0.99 1.06 0.98 2.03 1.03 4.15 1.93 3.98 4.03 6.93 4.09 2.02 1.14 2.95 3.36 3.44 10.36
9.0 8.5 8.0 7.5 7.0 6.5 6.0 5.5 5.0 4.5 4.0 3.5 3.0 2.5 2.0 1.5 1.0 0.5 0.0
δ (ppm)

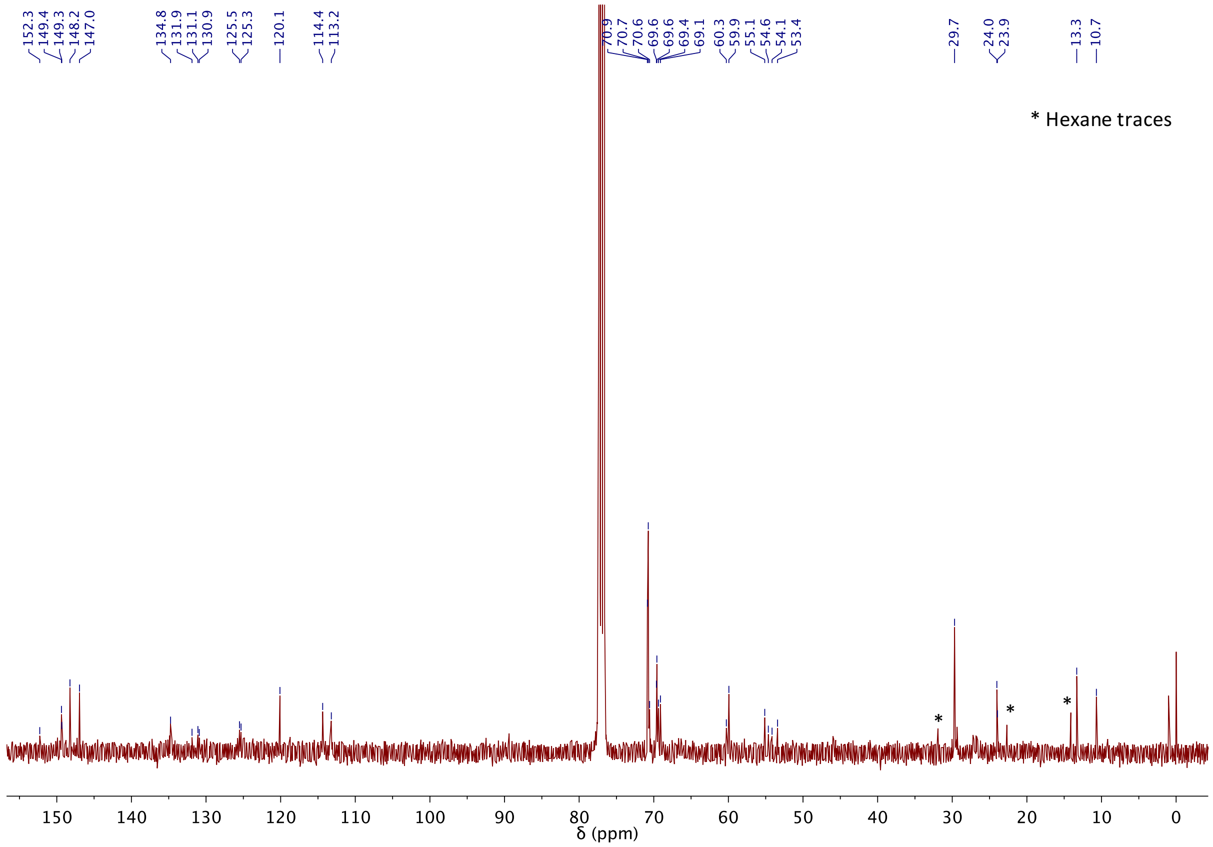
152.3 149.4 149.3 148.2 147.0 134.8 131.9 131.1 130.9 125.5 125.3 120.1 114.4 113.2 70.9 70.7 70.6 69.6 69.4 69.1 60.3 59.9 55.1 54.6 54.1 53.4 29.7 24.0 23.9 13.3 10.7
* Hexane traces
150 140 130 120 110 100 90 80 70 60 50 40 30 20 10 0
δ (ppm)

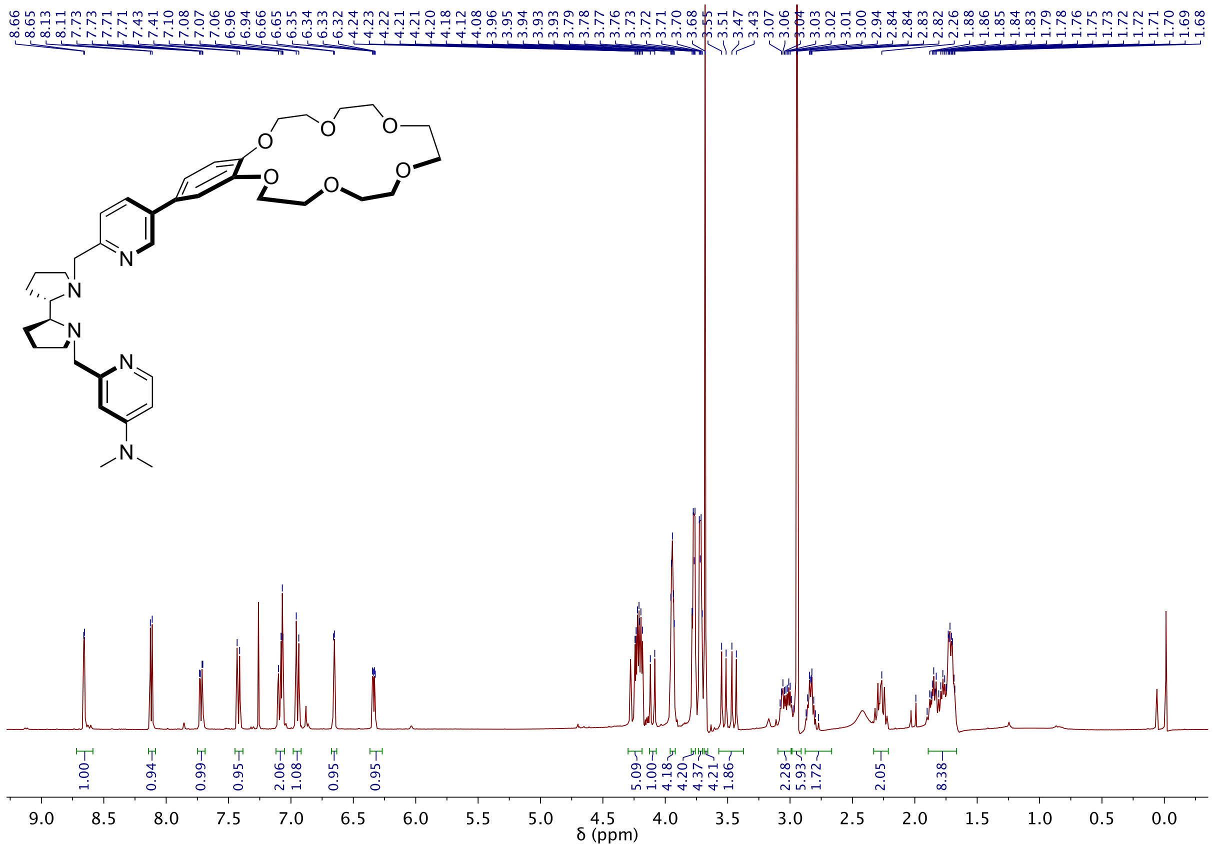
δ (ppm)

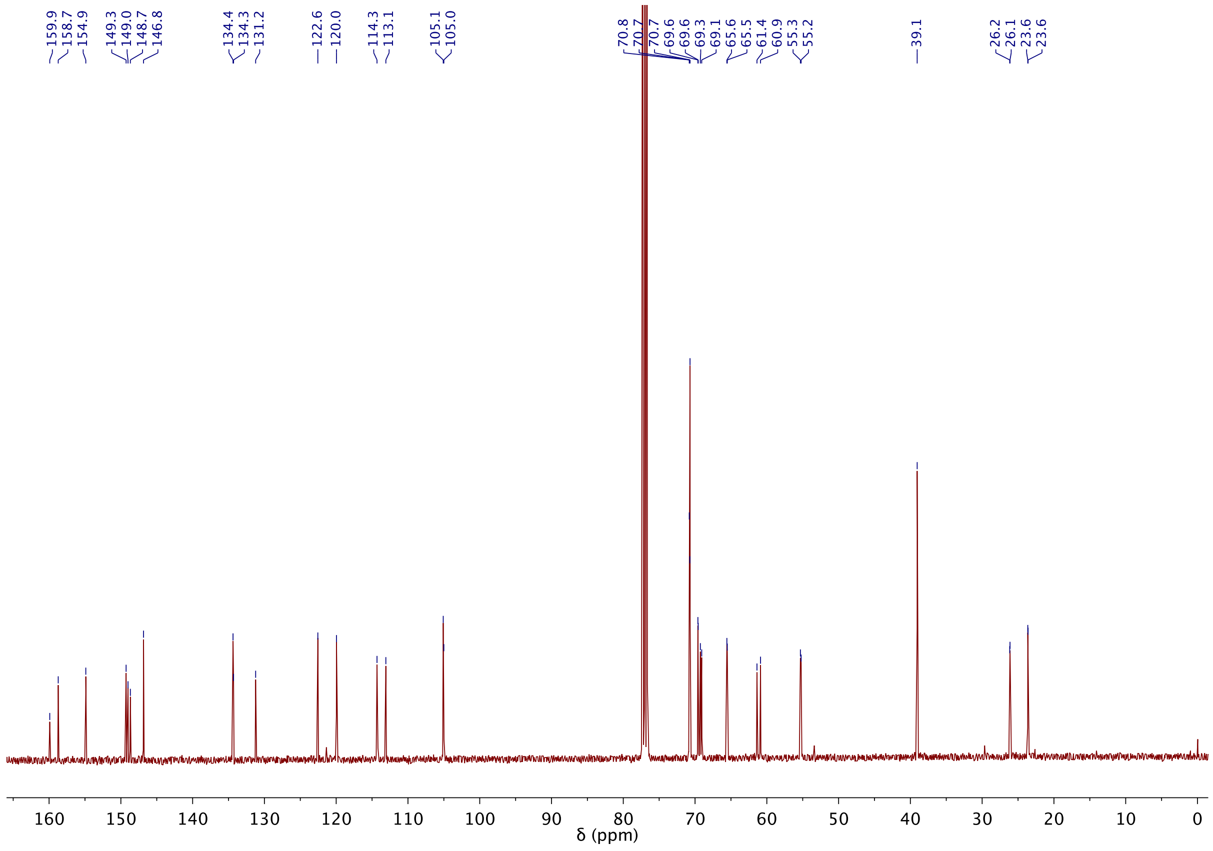
δ (ppm)

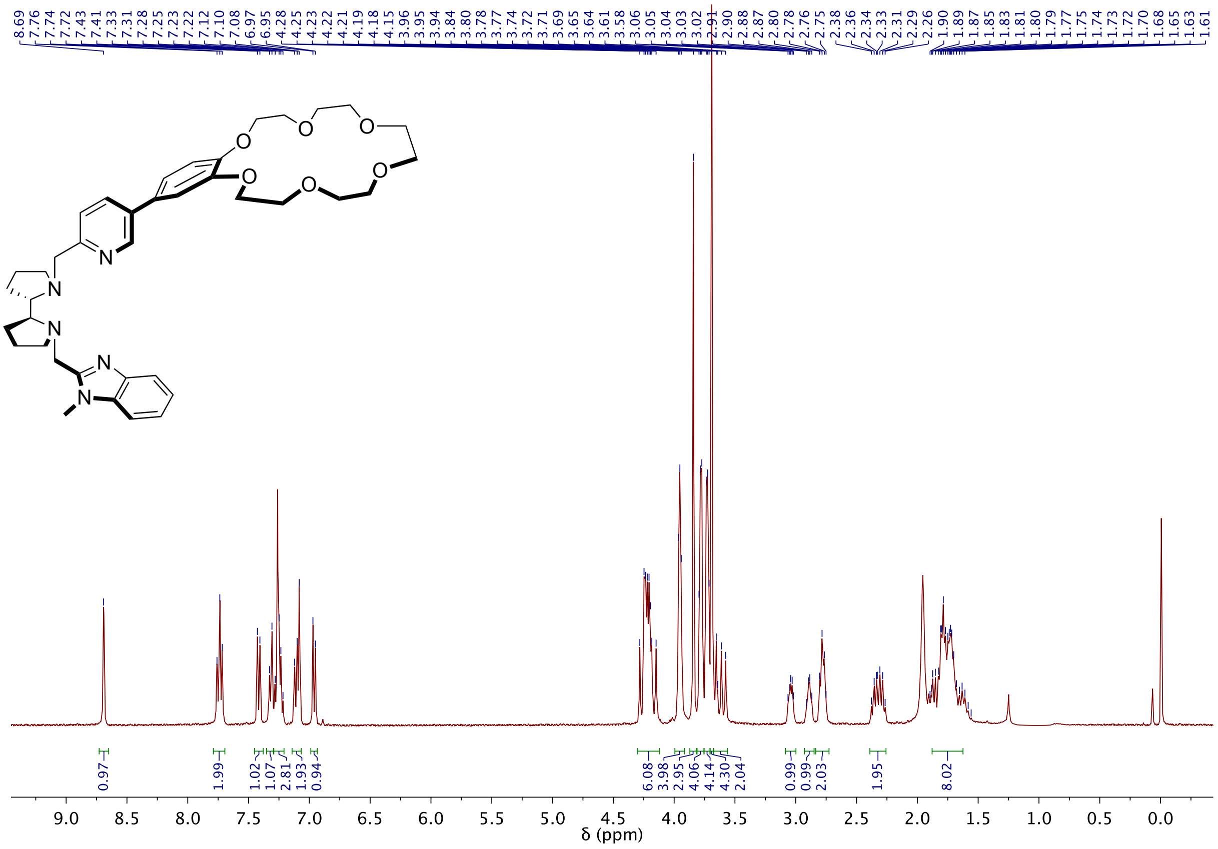
8.69 7.76 7.74 7.72 7.43 7.41 7.33 7.31 7.28 7.25 7.23 7.22 7.12 7.10 7.08 6.97 6.95 4.28 4.25 4.23 4.22 4.21 4.19 4.18 4.15 3.96 3.95 3.94 3.84 3.80 3.78 3.77 3.74 3.72 3.71 3.69 3.65 3.64 3.61 3.58 3.06 3.05 3.04 3.03 3.02 2.91 2.90 2.88 2.87 2.80 2.78 2.76 2.75 2.38 2.36 2.34 2.33 2.31 2.29 2.26 1.90 1.89 1.87 1.85 1.83 1.81 1.80 1.79 1.77 1.75 1.74 1.73 1.70 1.68 1.65 1.63 1.61
0.97 1.99 1.02 1.07 2.81 1.93 0.94 6.08 3.98 2.95 4.06 4.14 4.30 2.04 0.99 0.99 2.03 1.95 8.02
9.0 8.5 8.0 7.5 7.0 6.5 6.0 5.5 5.0 4.5 4.0 3.5 3.0 2.5 2.0 1.5 1.0 0.5 0.0
δ (ppm)

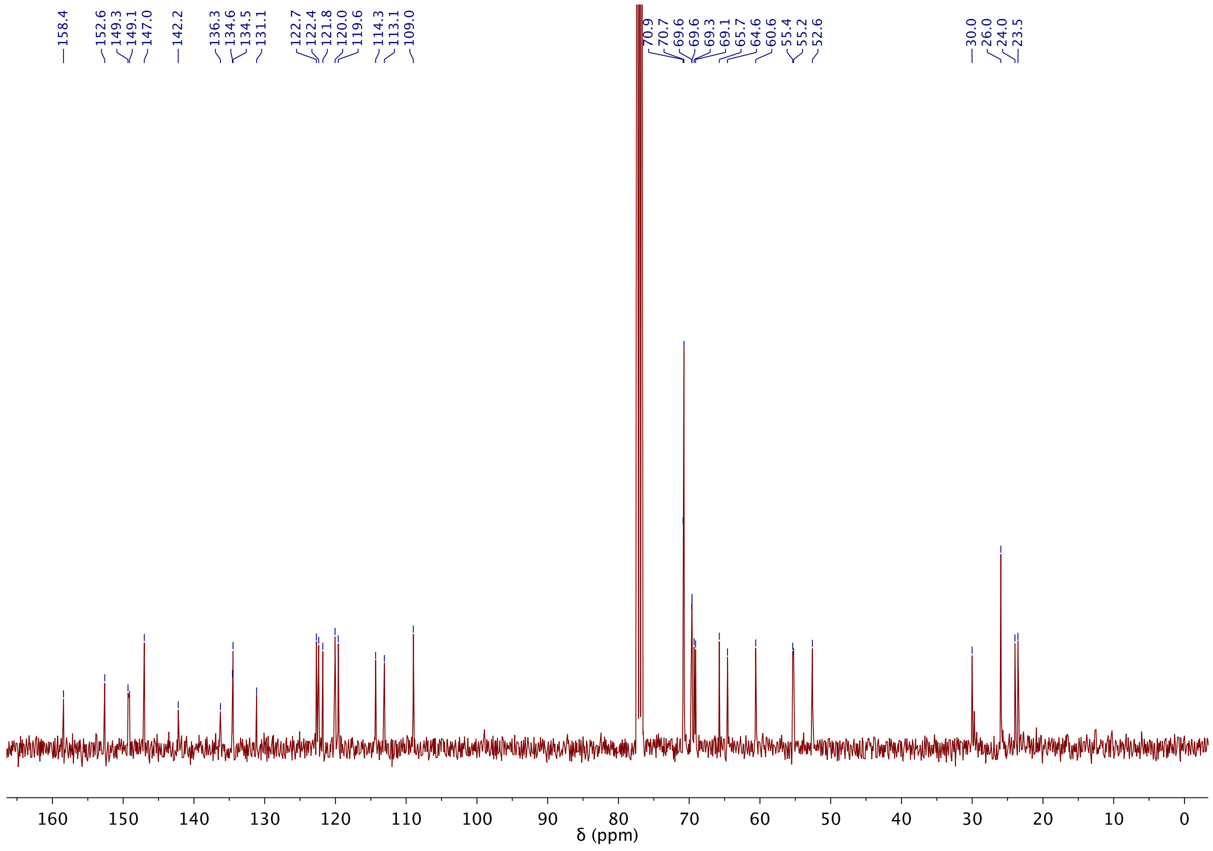
158.4 152.6 149.3 149.1 147.0 142.2 136.3 134.6 134.5 131.1 122.7 122.4 121.8 120.0 119.6 114.3 113.1 109.0 70.9 70.7 69.6 69.3 69.1 65.7 64.6 60.6 55.4 55.2 52.6 30.0 26.0 24.0 23.5
160 150 140 130 120 110 100 90 80 70 60 50 40 30 20 10 0
δ (ppm)

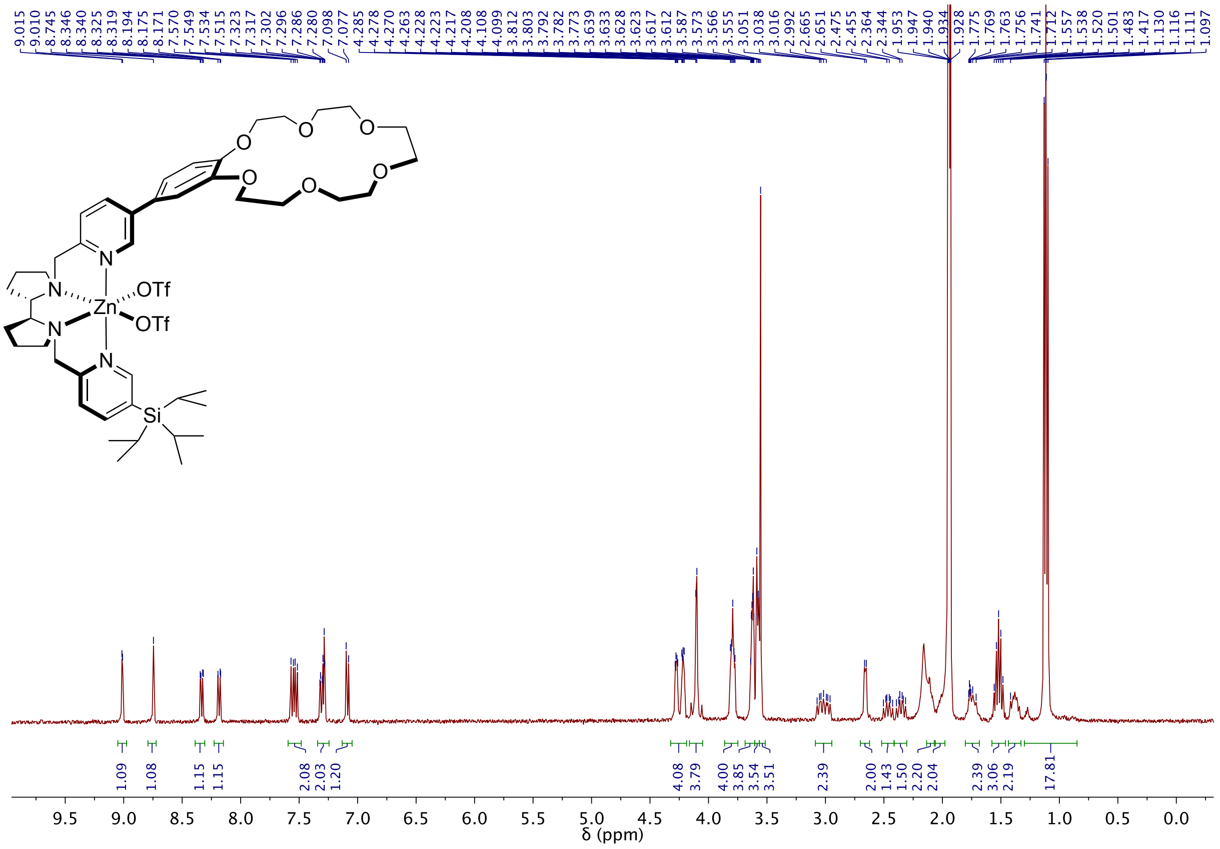
9.015
9.010
8.745
8.346
8.340
8.325
8.319
8.194
8.175
8.171
7.570
7.549
7.534
7.515
7.323
7.317
7.302
7.296
7.286
7.280
7.098
7.077
4.285
4.270
4.263
4.228
4.223
4.217
4.208
4.108
4.099
3.812
3.803
3.792
3.782
3.773
3.639
3.633
3.628
3.623
3.617
3.612
3.587
3.573
3.566
3.555
3.551
3.051
3.038
3.016
2.992
2.665
2.651
2.475
2.455
2.364
2.344
1.953
1.947
1.940
1.934
1.928
1.775
1.769
1.763
1.756
1.741
1.712
1.557
1.538
1.520
1.501
1.483
1.417
1.130
1.116
1.111
1.097
O
O
O
O
O
O
N
N
N
Zn
N
OTf
OTf
Si
1.09
1.08
1.15
1.15
2.08
2.03
1.20
4.08
3.79
4.00
3.85
3.54
3.51
2.39
2.00
1.43
1.50
2.20
2.04
2.39
3.06
2.19
17.81
9.5 9.0 8.5 8.0 7.5 7.0 6.5 6.0 5.5 5.0 4.5 4.0 3.5 3.0 2.5 2.0 1.5 1.0 0.5 0.0
δ (ppm)

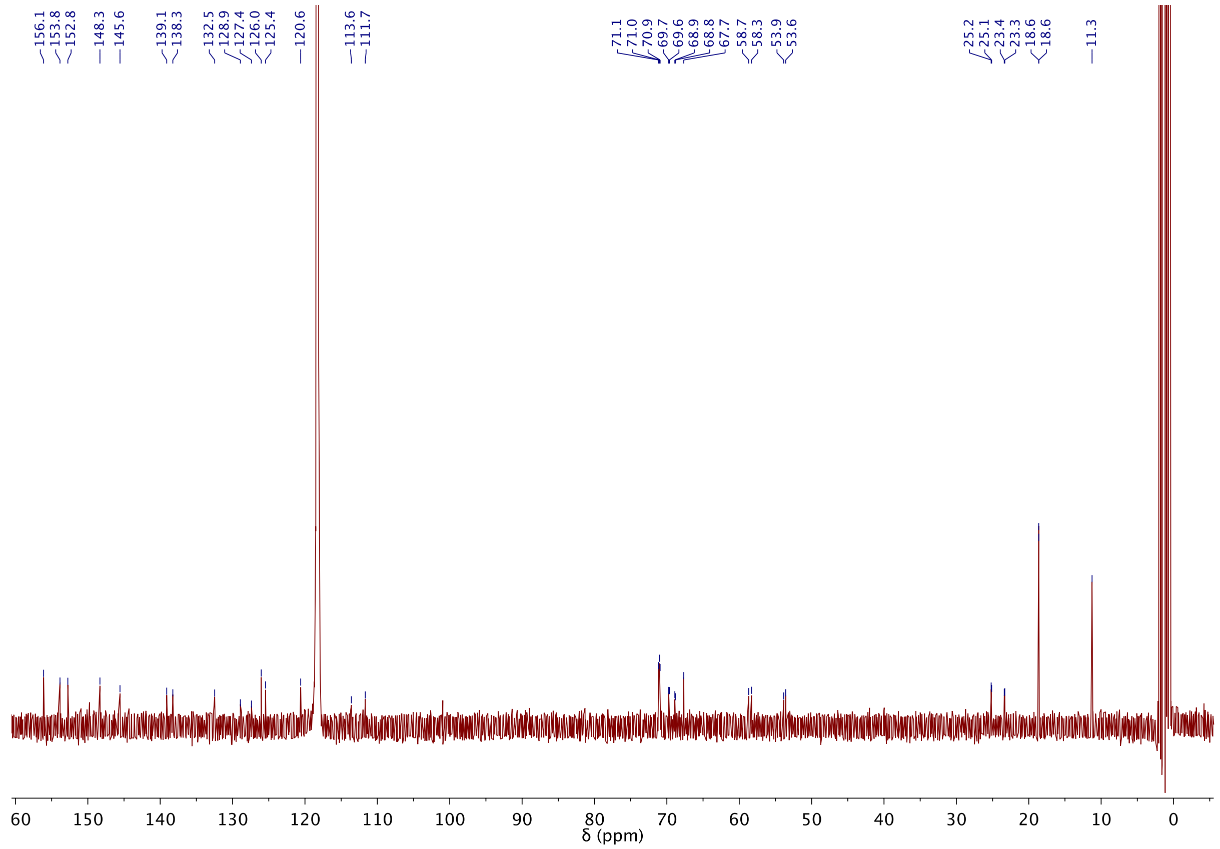
156.1
153.8
152.8
148.3
145.6
139.1
138.3
132.5
128.9
127.4
126.0
125.4
120.6
113.6
111.7
71.1
71.0
70.9
69.7
69.6
68.9
68.8
67.7
58.7
58.3
53.9
53.6
25.2
25.1
23.4
23.3
18.6
18.6
11.3
60 150 140 130 120 110 100 90 80 70 60 50 40 30 20 10 0
δ (ppm)

13. ¹H and ¹³C{H} NMR spectra of the α,ω-amino acids

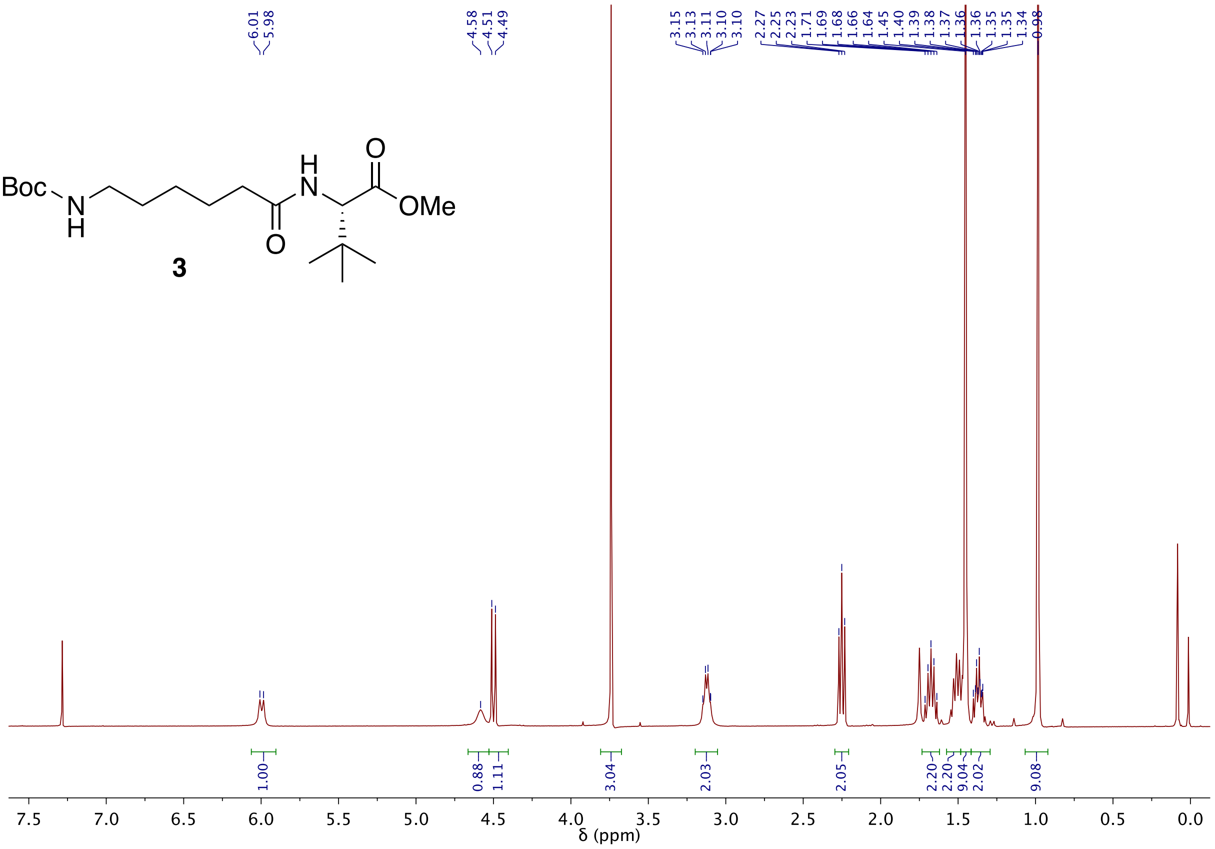

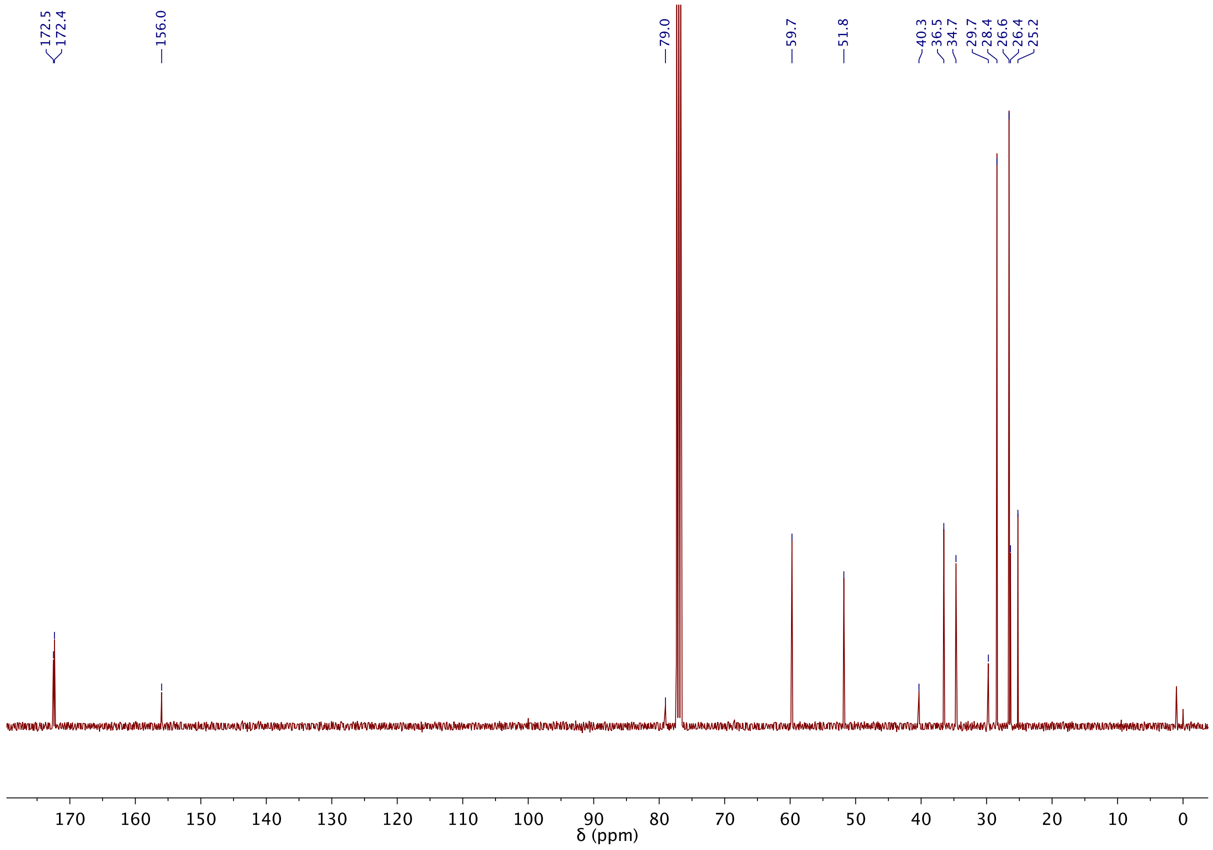

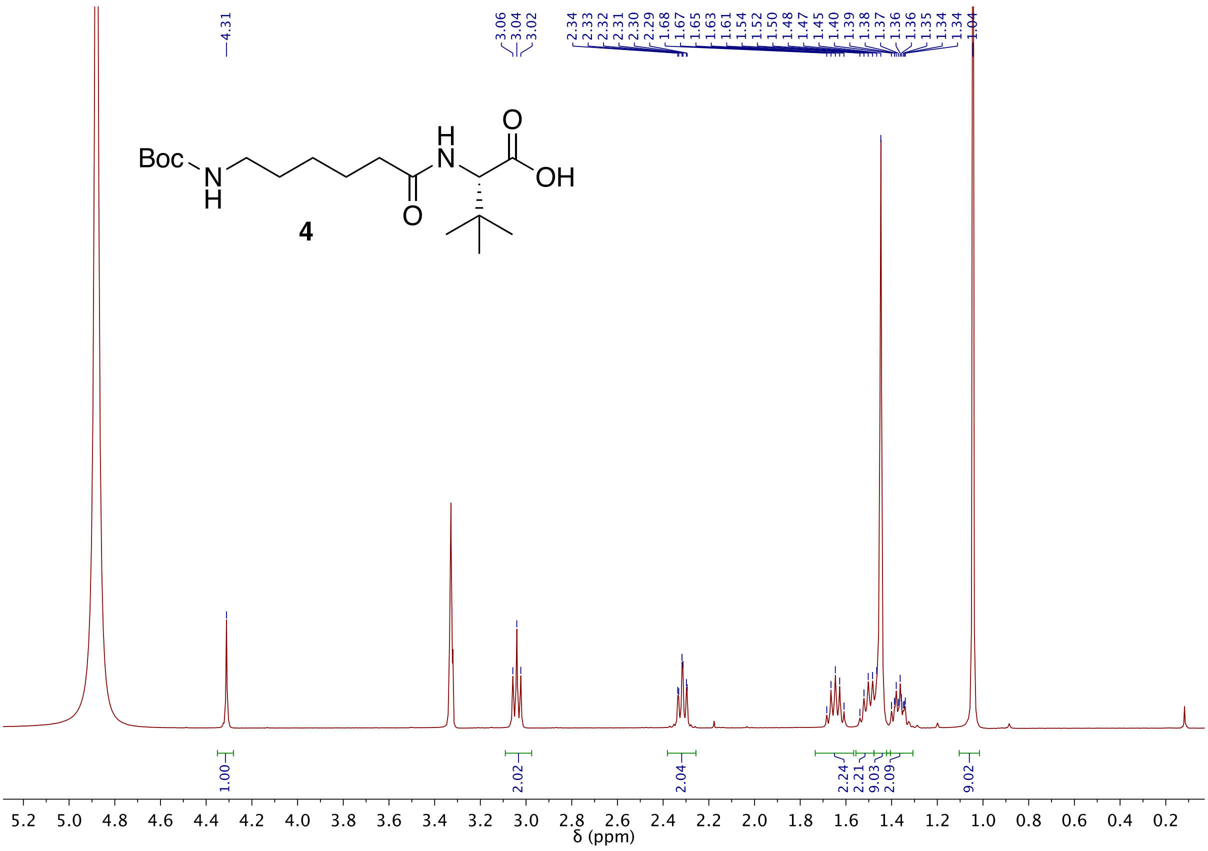
4.31
3.06
3.04
3.02
2.34 2.33 2.32 2.31 2.30 2.29 1.68 1.67 1.65 1.63 1.61 1.54 1.52 1.50 1.48 1.47 1.45 1.40 1.39 1.38 1.37 1.36 1.36 1.35 1.34 1.34 1.04
Boc
N
H
H
N
O
OH
O
4
1.00
2.02
2.04
2.24
2.21
9.03
2.09
9.02
5.2 5.0 4.8 4.6 4.4 4.2 4.0 3.8 3.6 3.4 3.2 3.0 2.8 2.6 2.4 2.2 2.0 1.8 1.6 1.4 1.2 1.0 0.8 0.6 0.4 0.2
δ (ppm)

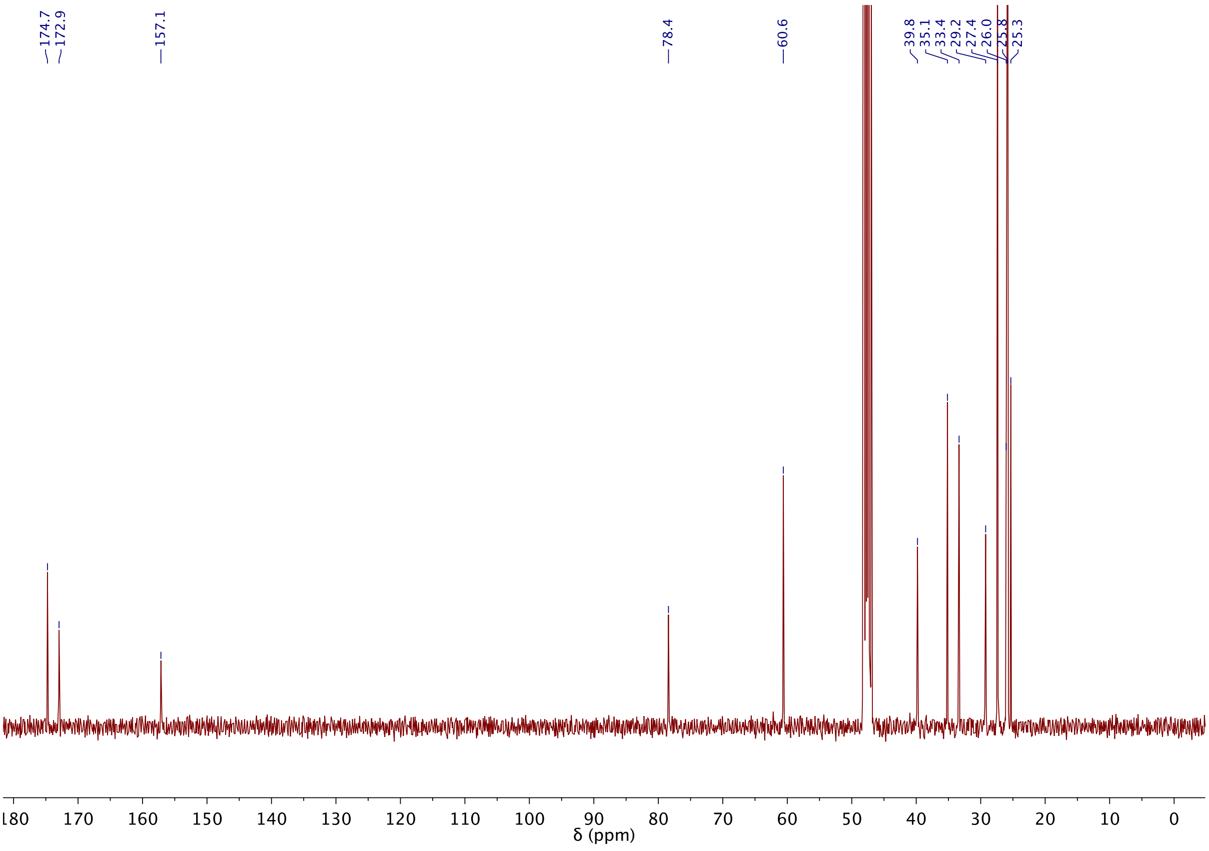
174.7
172.9
157.1
78.4
60.6
39.8 35.1 33.4 29.2 27.4 26.0 25.8 25.3
180 170 160 150 140 130 120 110 100 90 80 70 60 50 40 30 20 10 0
δ (ppm)

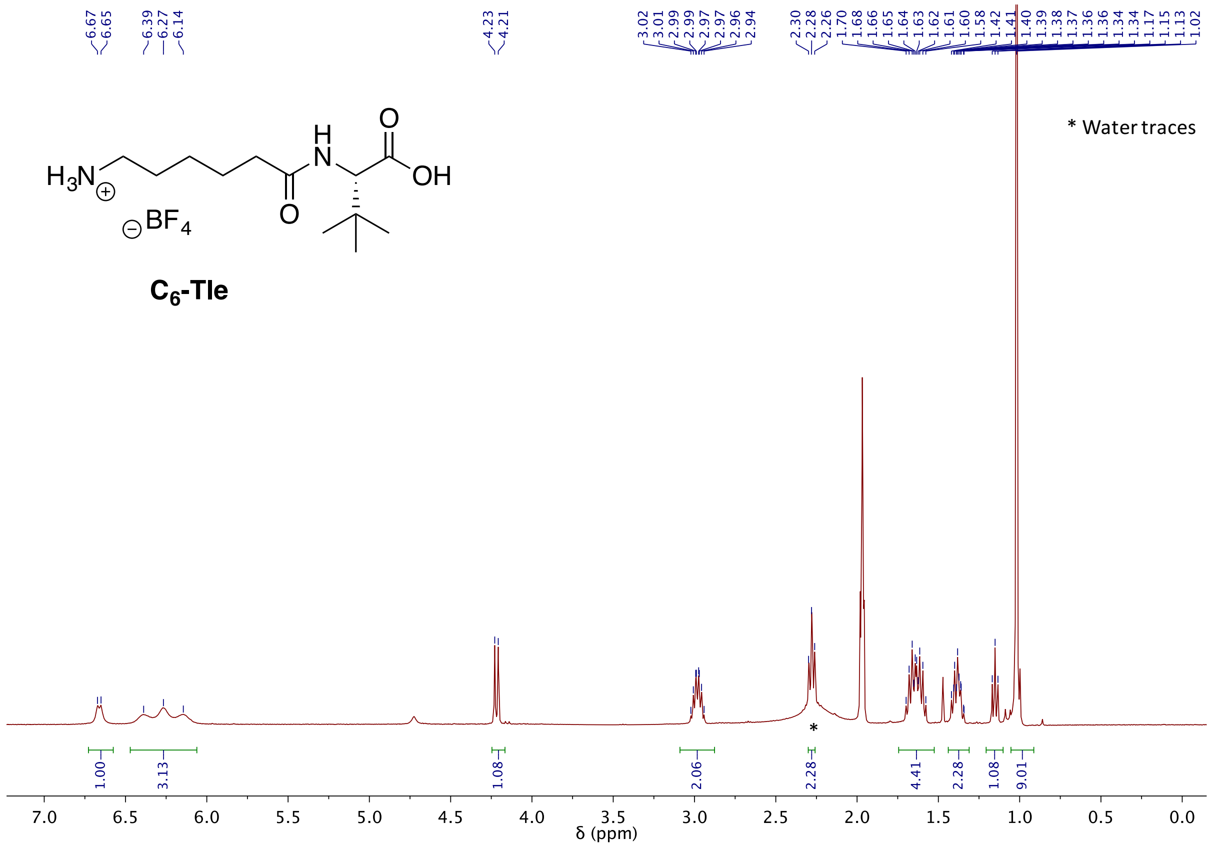

H3N
BF4
O
H
N
O
OH
O
C6-Tle
* Water traces
δ (ppm)

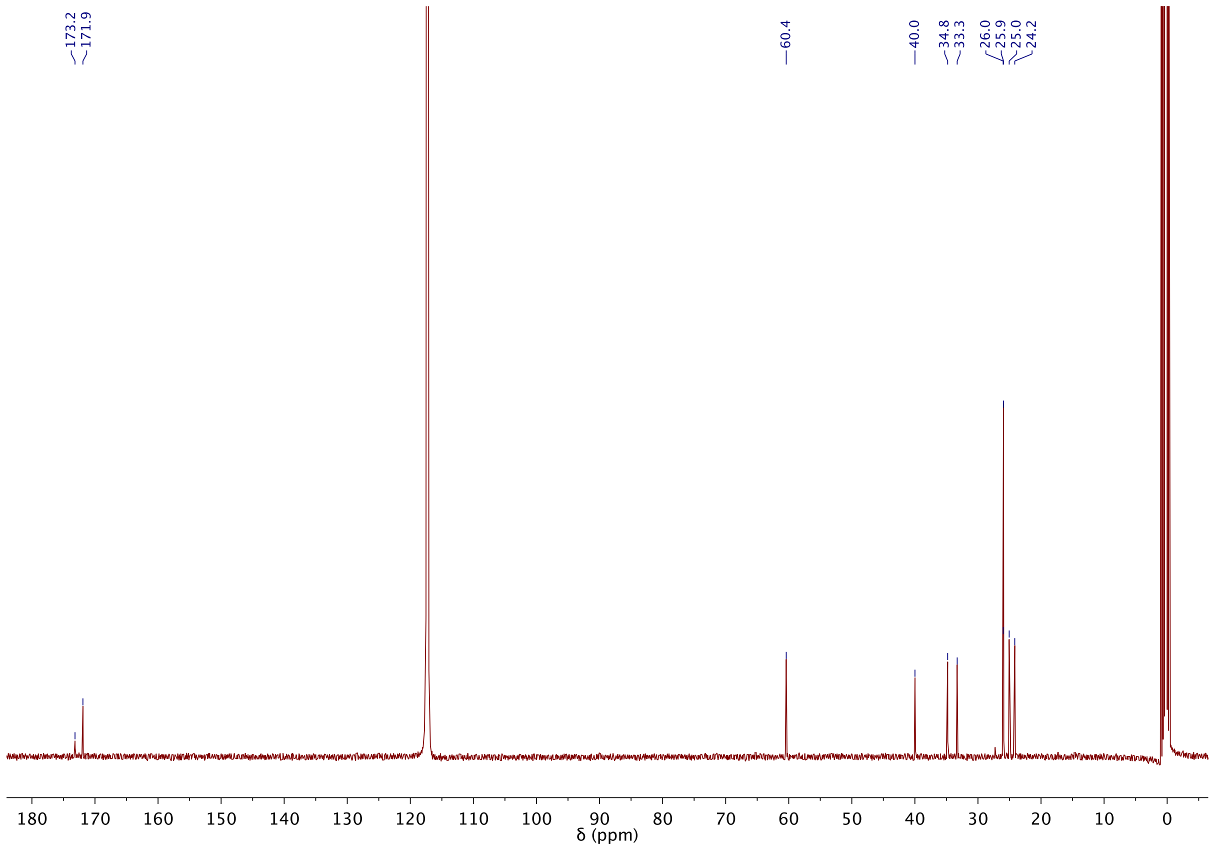

δ (ppm)

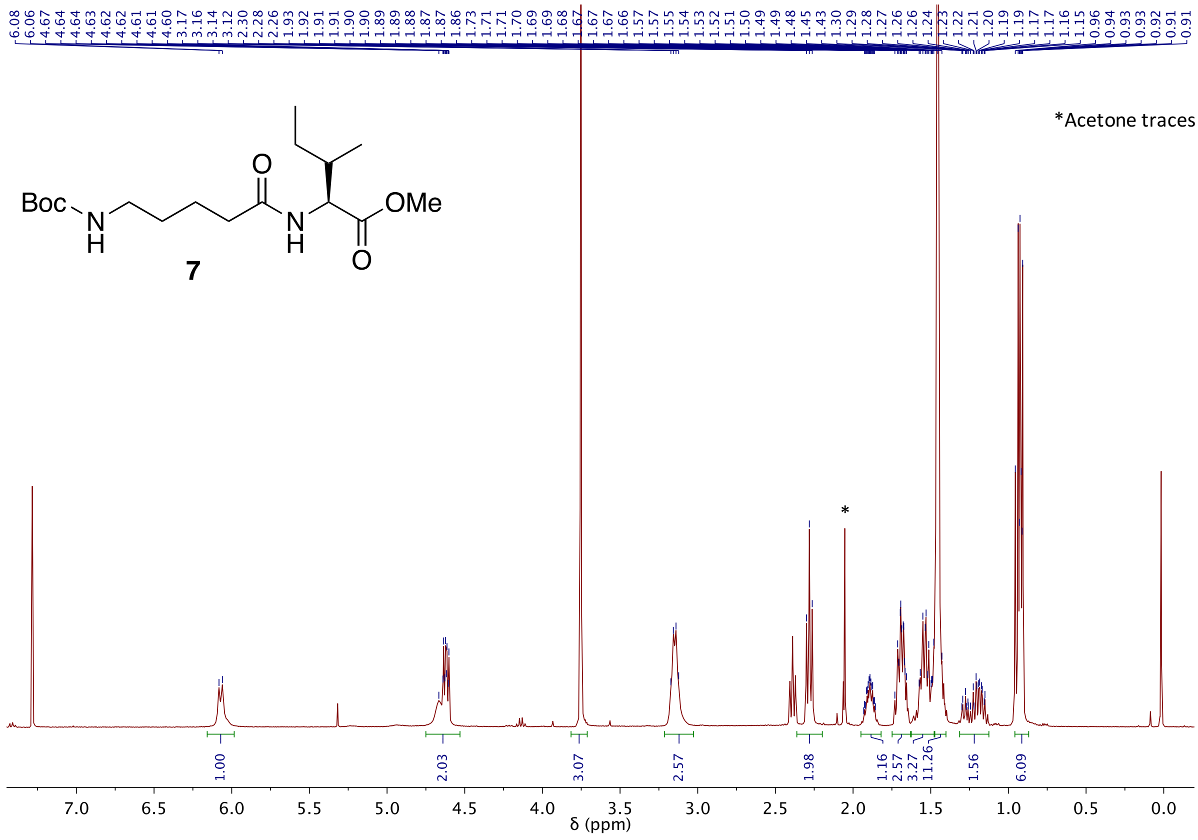
*Acetone traces
Boc
N
H
O
N
H
OMe
O
7
δ (ppm)

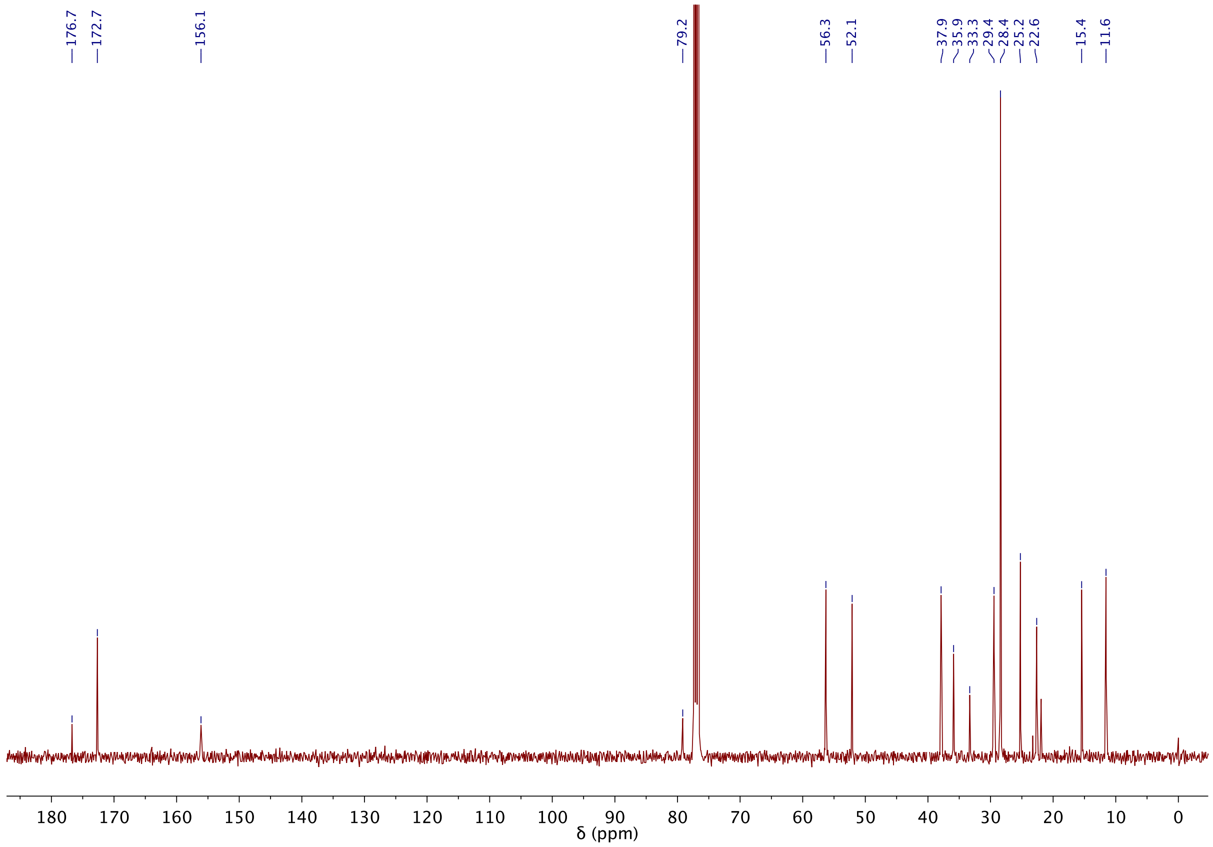
δ (ppm)

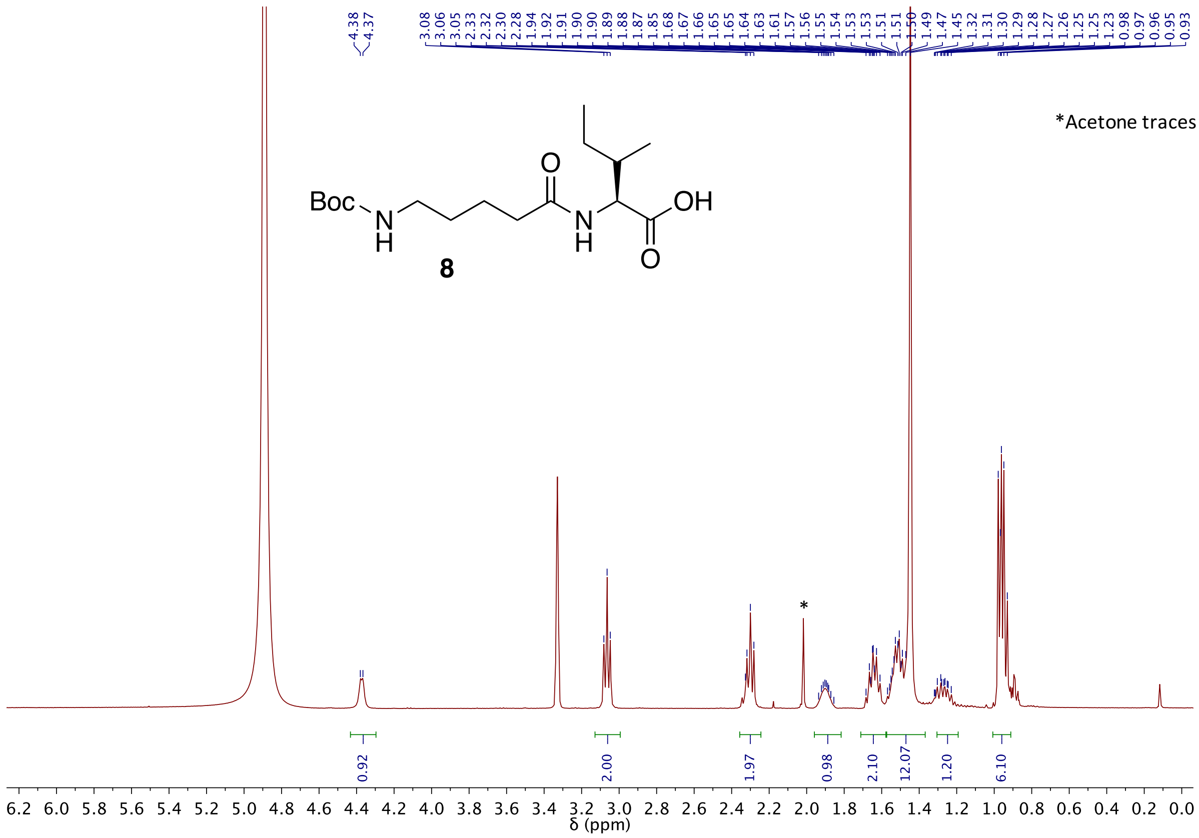
*Acetone traces
Boc
N
H
O
N
H
OH
O
8
δ (ppm)

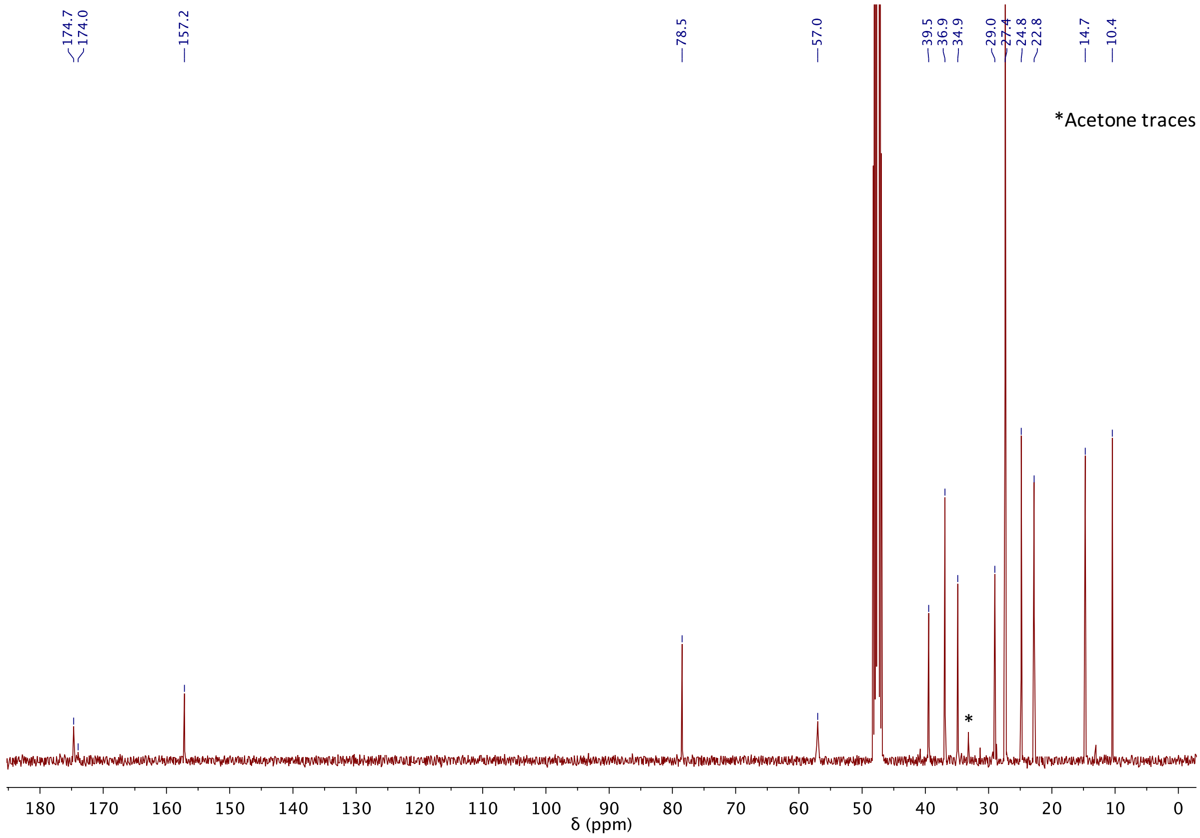
*Acetone traces
δ (ppm)

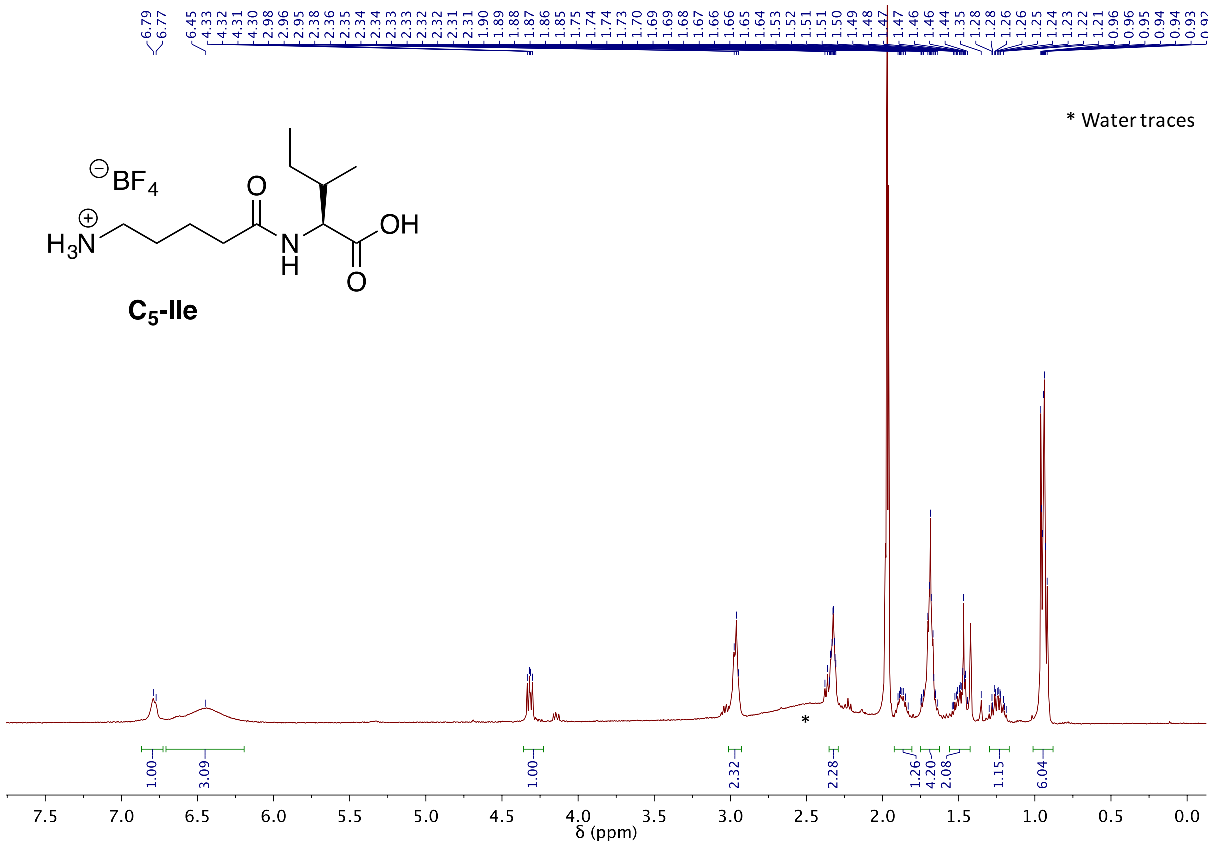
6.79
6.77
6.45
4.33
4.32
4.31
4.30
2.98
2.96
2.95
2.38
2.36
2.35
2.34
2.34
2.33
2.33
2.32
2.32
2.31
2.31
1.90
1.89
1.88
1.87
1.86
1.85
1.75
1.74
1.73
1.70
1.69
1.69
1.68
1.67
1.66
1.65
1.64
1.53
1.52
1.51
1.51
1.50
1.49
1.48
1.47
1.47
1.46
1.46
1.44
1.35
1.28
1.28
1.26
1.26
1.25
1.24
1.23
1.22
1.21
0.96
0.96
0.95
0.94
0.94
0.93
* Water traces
BF4
O
OH
H3N
N
H
O
C5-Ile
*
1.00
3.09
1.00
2.32
2.28
1.26
4.20
2.08
1.15
6.04
δ (ppm)

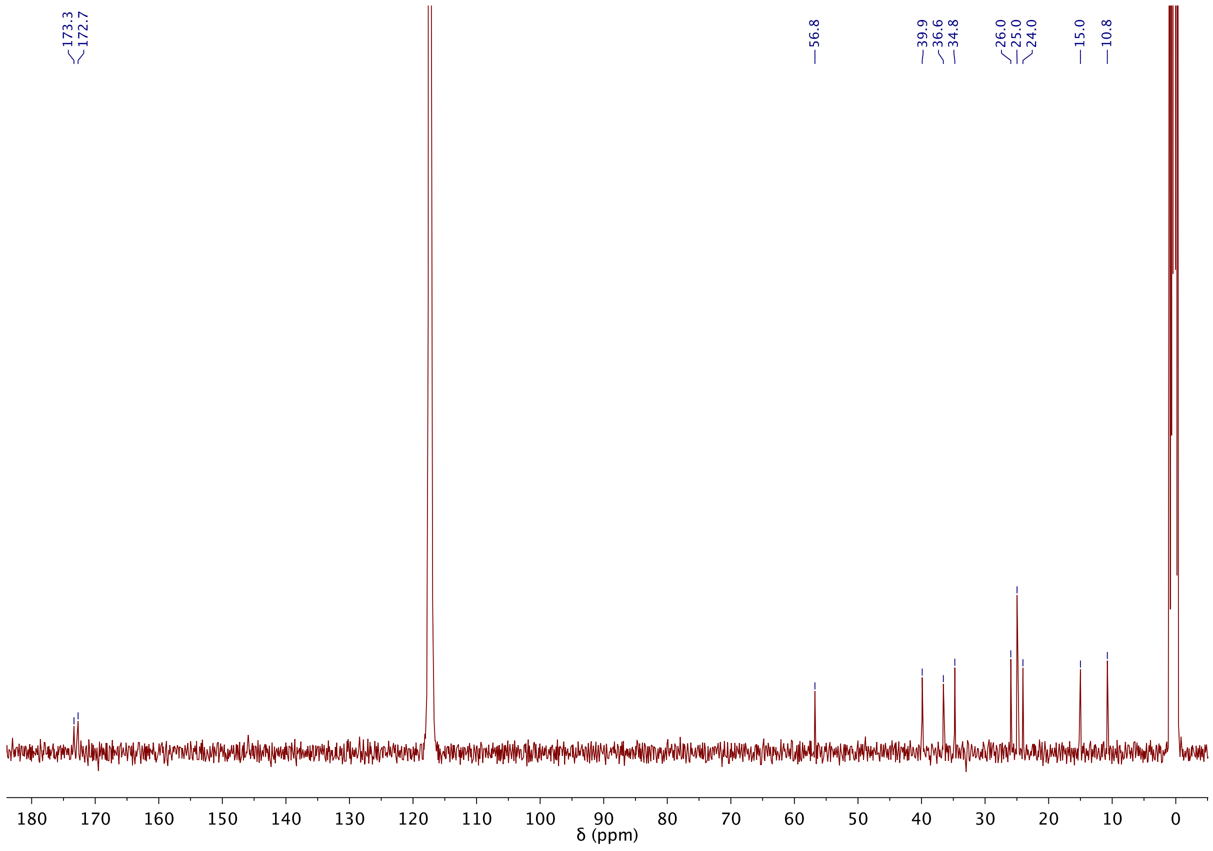
173.3
172.7
56.8
39.9
36.6
34.8
26.0
25.0
24.0
15.0
10.8
δ (ppm)

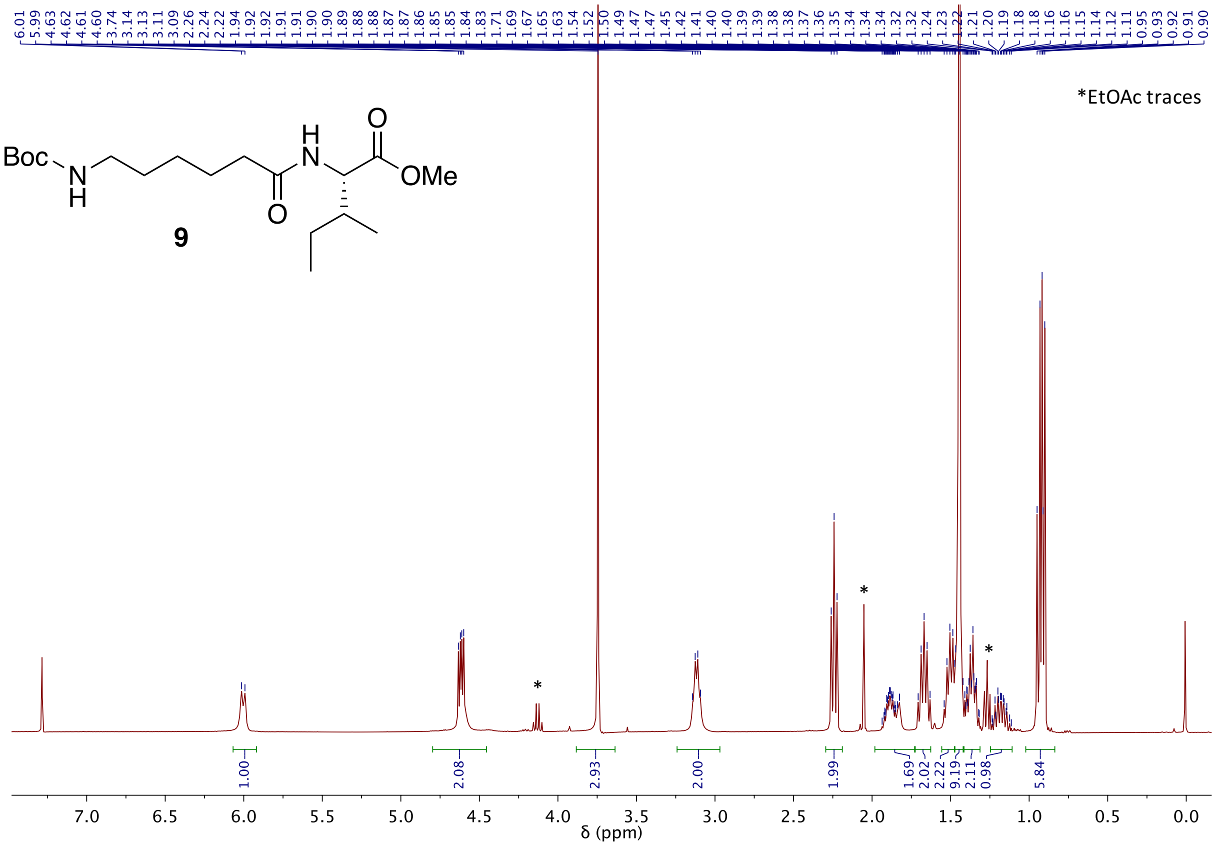
*EtOAc traces
Boc
N
H
N
H
O
O
OMe
9
δ (ppm)

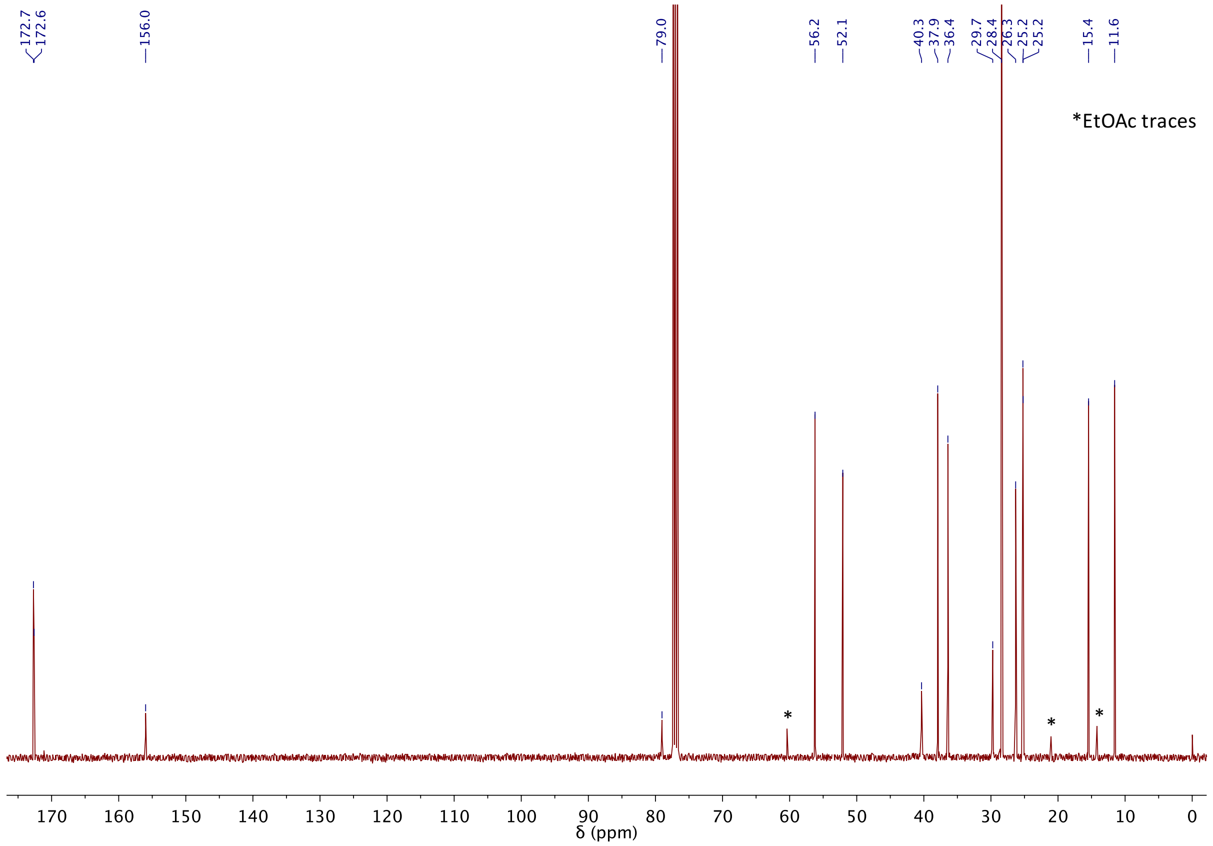
172.7
172.6
156.0
79.0
56.2
52.1
40.3
37.9
36.4
29.7
28.4
26.3
25.2
25.2
15.4
11.6
*EtOAc traces
δ (ppm)

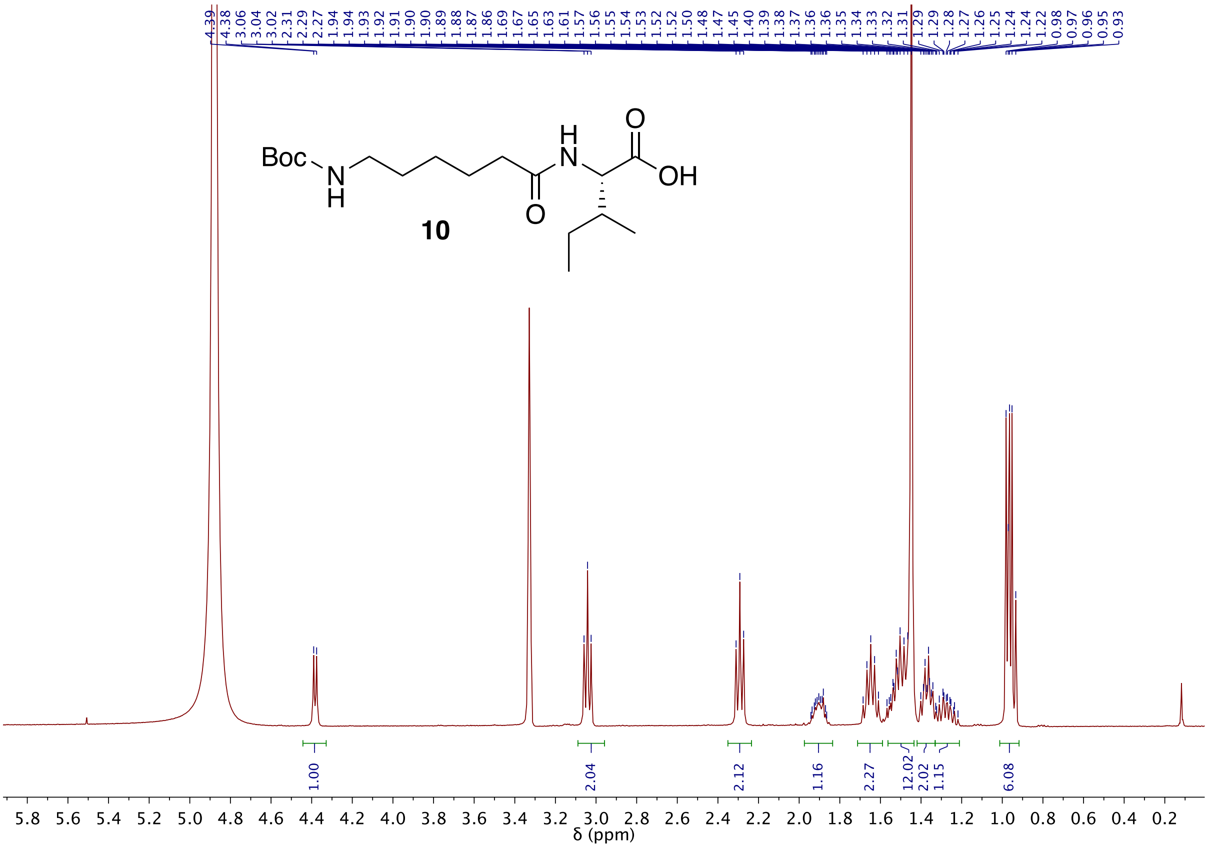
4.39 4.38 3.06 3.04 3.02 2.31 2.29 2.27 1.94 1.94 1.93 1.92 1.91 1.90 1.90 1.89 1.88 1.87 1.86 1.69 1.67 1.65 1.63 1.61 1.57 1.56 1.55 1.54 1.53 1.52 1.52 1.50 1.48 1.47 1.45 1.40 1.39 1.38 1.37 1.36 1.36 1.35 1.34 1.33 1.32 1.31 1.29 1.29 1.28 1.28 1.27 1.26 1.25 1.24 1.24 1.22 0.98 0.97 0.96 0.95 0.93
Boc
N
H
H
N
O
OH
O
10
1.00
2.04
2.12
1.16
2.27
12.02
2.02
1.15
6.08
5.8 5.6 5.4 5.2 5.0 4.8 4.6 4.4 4.2 4.0 3.8 3.6 3.4 3.2 3.0 2.8 2.6 2.4 2.2 2.0 1.8 1.6 1.4 1.2 1.0 0.8 0.6 0.4 0.2
δ (ppm)

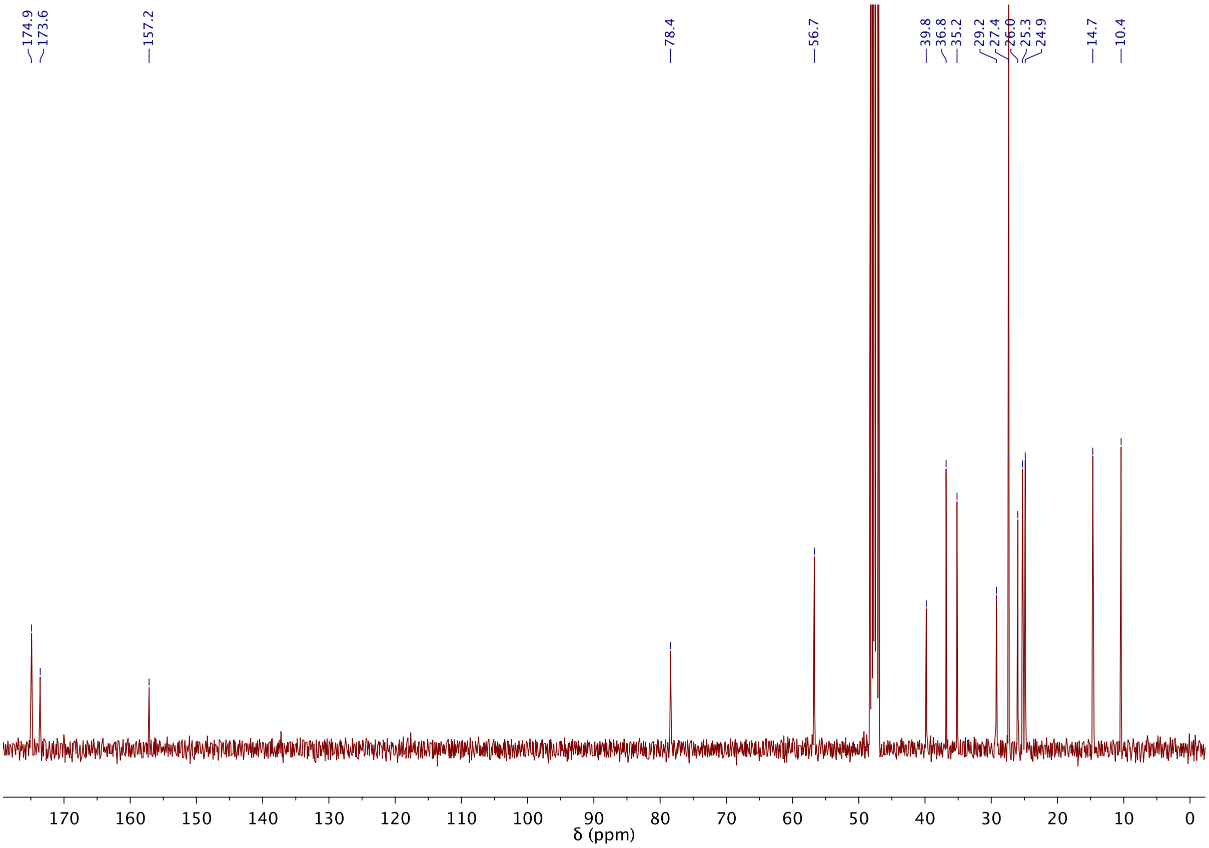
174.9 173.6 157.2 78.4 56.7 39.8 36.8 35.2 29.2 27.4 26.0 25.3 24.9 14.7 10.4
170 160 150 140 130 120 110 100 90 80 70 60 50 40 30 20 10 0
δ (ppm)

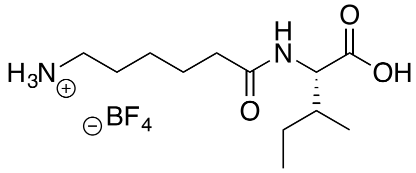
H3N
BF4
H
N
O
OH
O

C$_6$-Ile

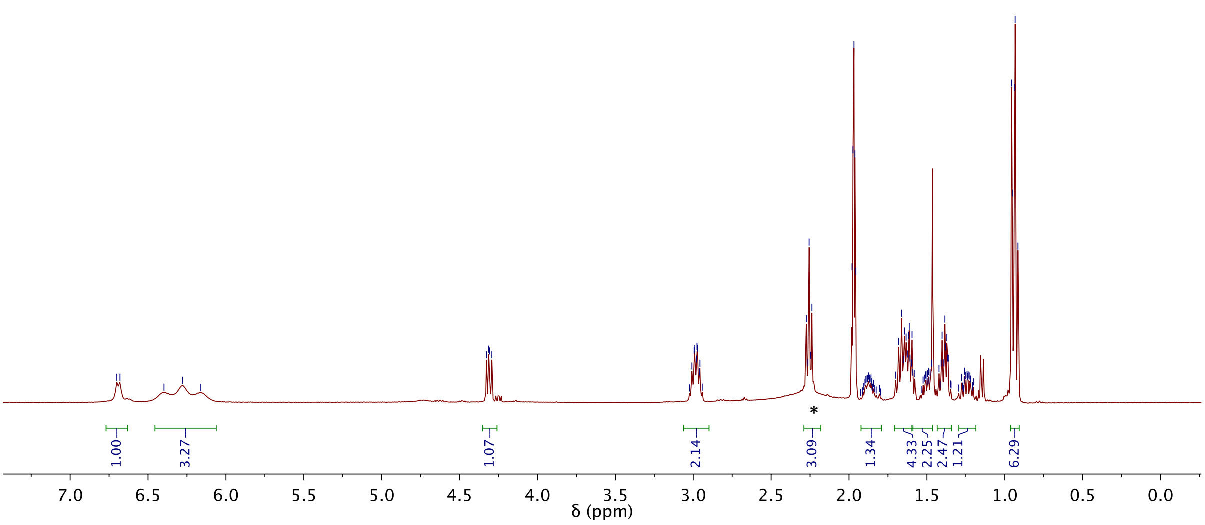
* Water traces
1.00
3.27
1.07
2.14
3.09
1.34
4.33
2.25
2.47
1.21
6.29
δ (ppm)

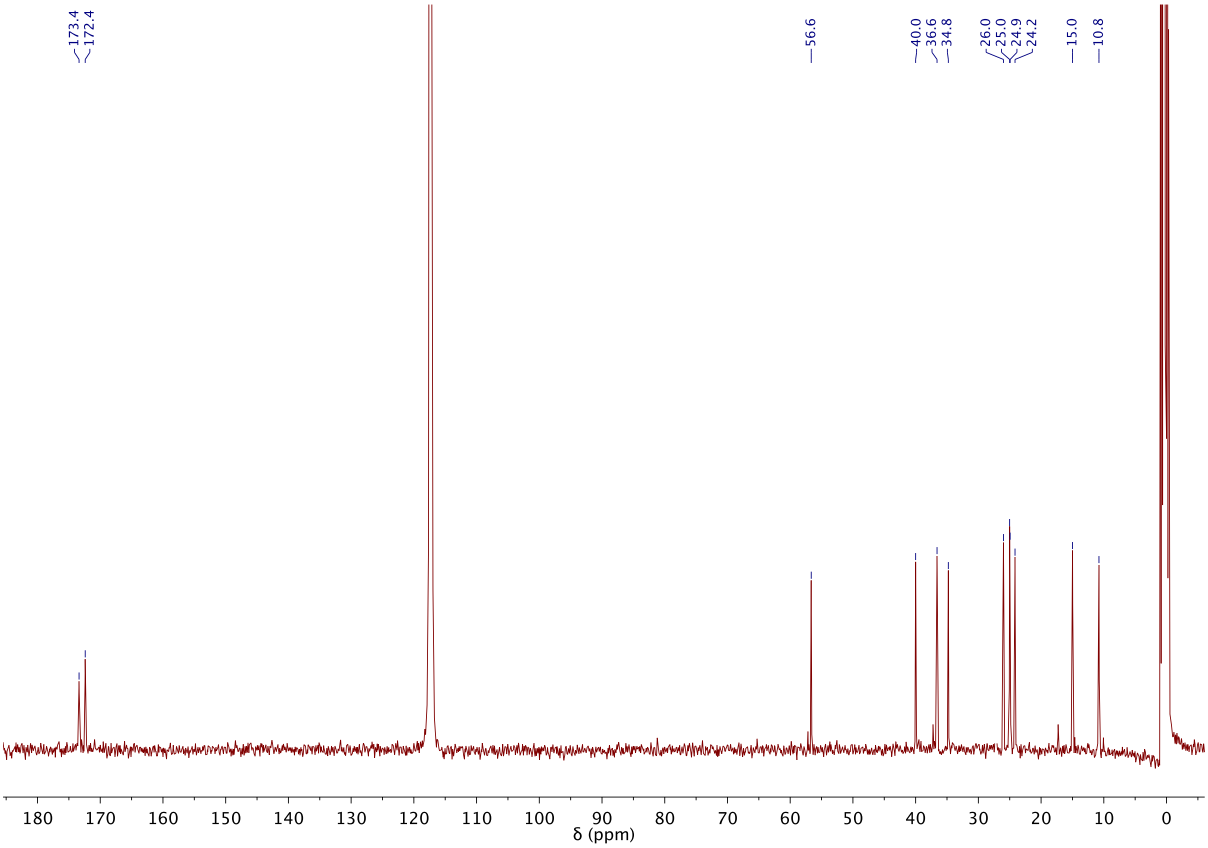
173.4
172.4
56.6
40.0
36.6
34.8
26.0
25.0
24.9
24.2
15.0
10.8
δ (ppm)

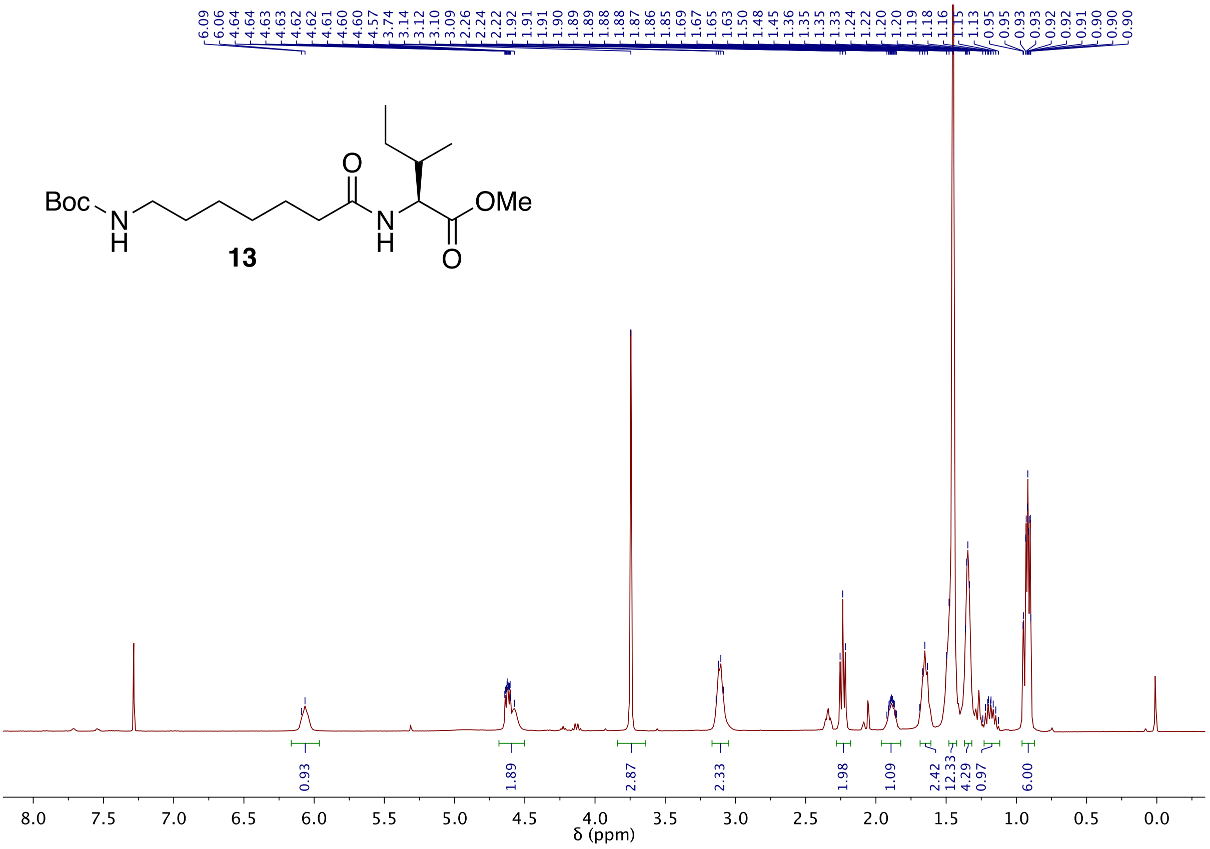
Boc
N
H
O
N
H
OMe
O
6.09 6.06 4.64 4.64 4.63 4.63 4.62 4.62 4.61 4.60 4.57 3.74 3.14 3.12 3.10 3.09 2.26 2.24 2.22 1.92 1.91 1.91 1.90 1.89 1.89 1.88 1.87 1.86 1.85 1.69 1.67 1.65 1.63 1.50 1.48 1.45 1.36 1.35 1.35 1.33 1.24 1.22 1.20 1.20 1.19 1.18 1.16 1.15 1.13 0.95 0.95 0.93 0.93 0.92 0.92 0.91 0.90 0.90 0.90
0.93
1.89
2.87
2.33
1.98
1.09
2.42
12.33
4.29
0.97
6.00
8.0 7.5 7.0 6.5 6.0 5.5 5.0 4.5 4.0 3.5 3.0 2.5 2.0 1.5 1.0 0.5 0.0
δ (ppm)

13

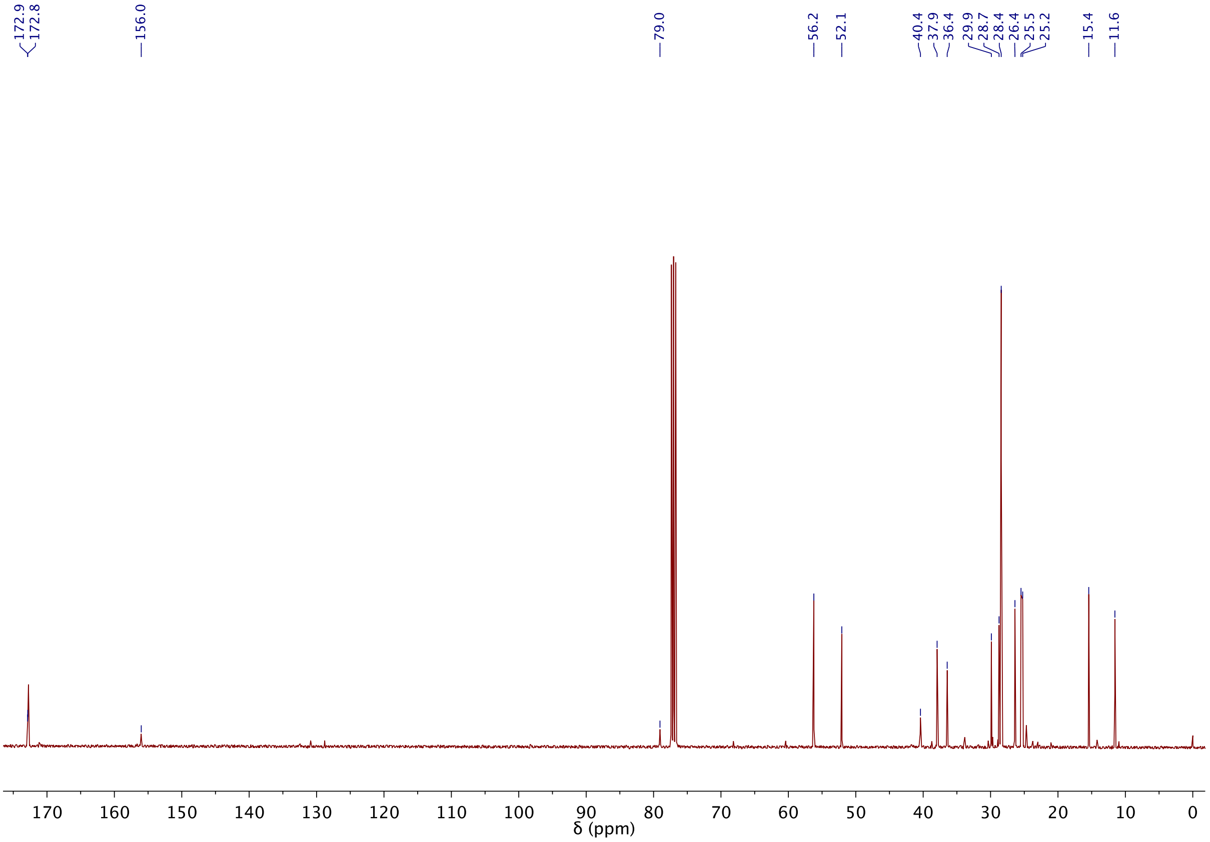
172.9 172.8
156.0
79.0
56.2
52.1
40.4 37.9 36.4 29.9 28.7 28.4 26.4 25.5 25.2
15.4
11.6
170 160 150 140 130 120 110 100 90 80 70 60 50 40 30 20 10 0
δ (ppm)

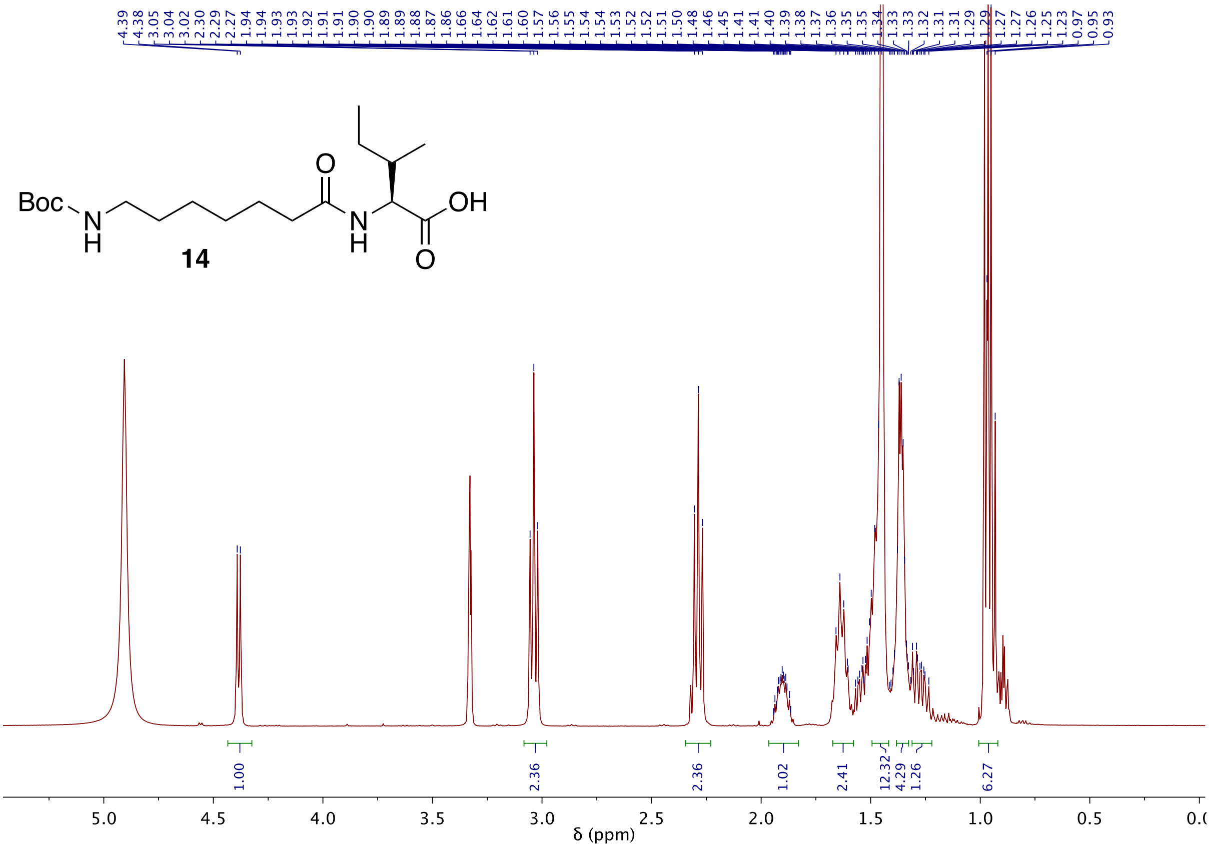

Boc
N
H
14
O
N
H
OH
O
δ (ppm)

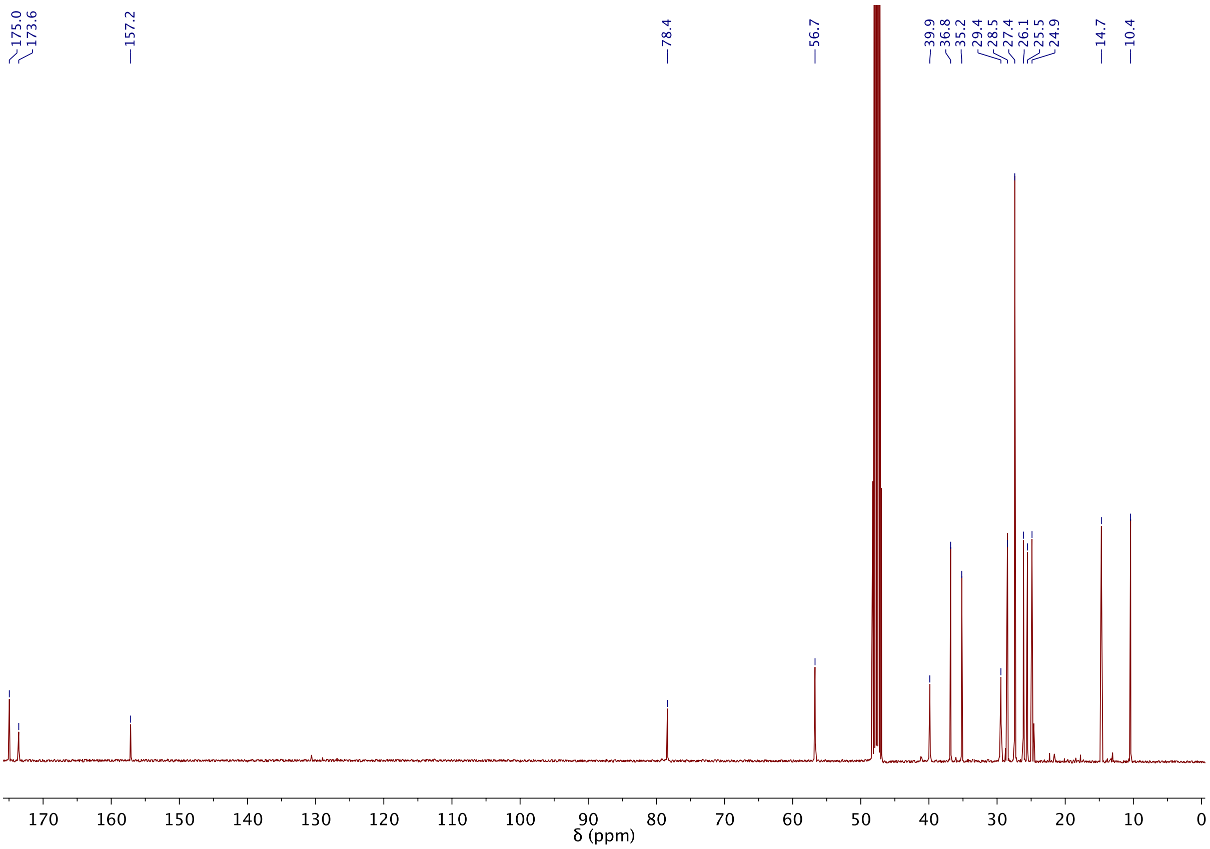

δ (ppm)

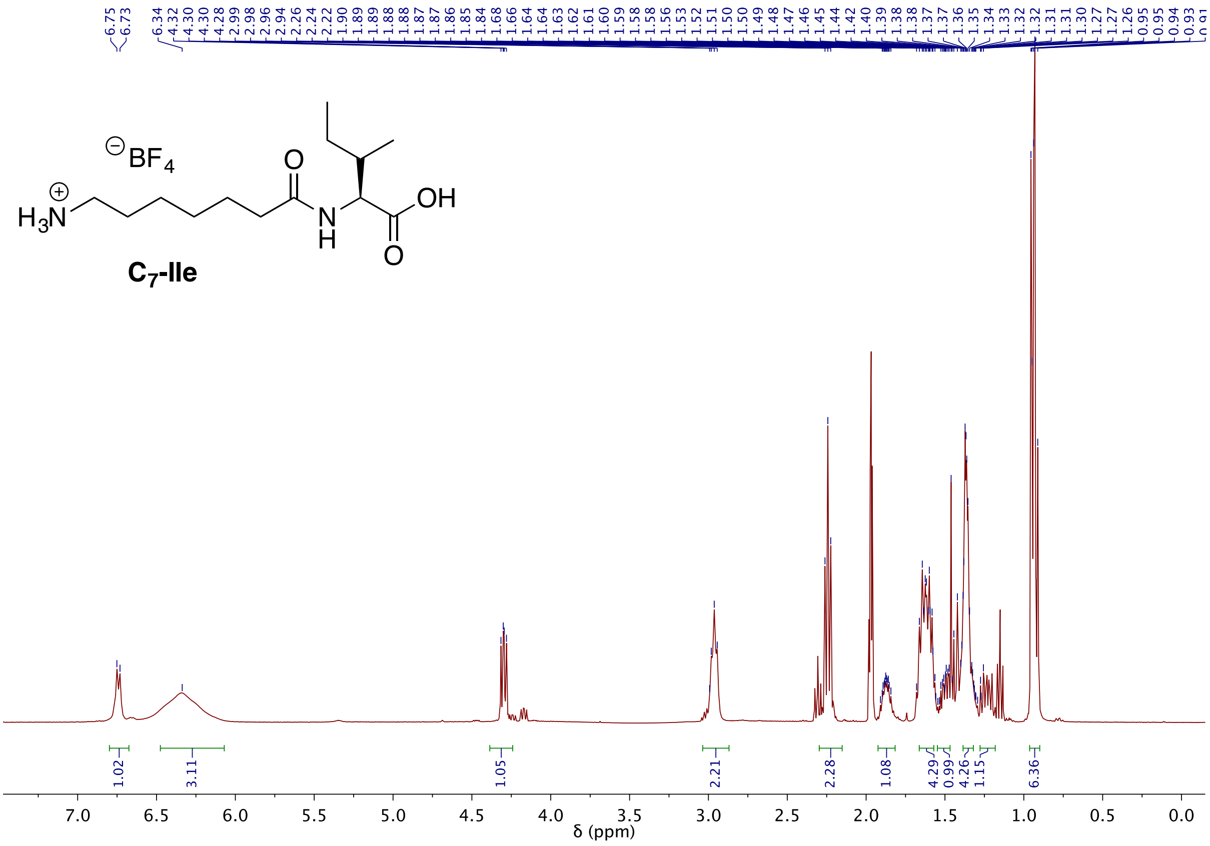

BF4
H3N
O
N
H
OH
O
C7-Ile
1.02
3.11
1.05
2.21
2.28
1.08
4.29
0.99
4.26
1.15
6.36
δ (ppm)

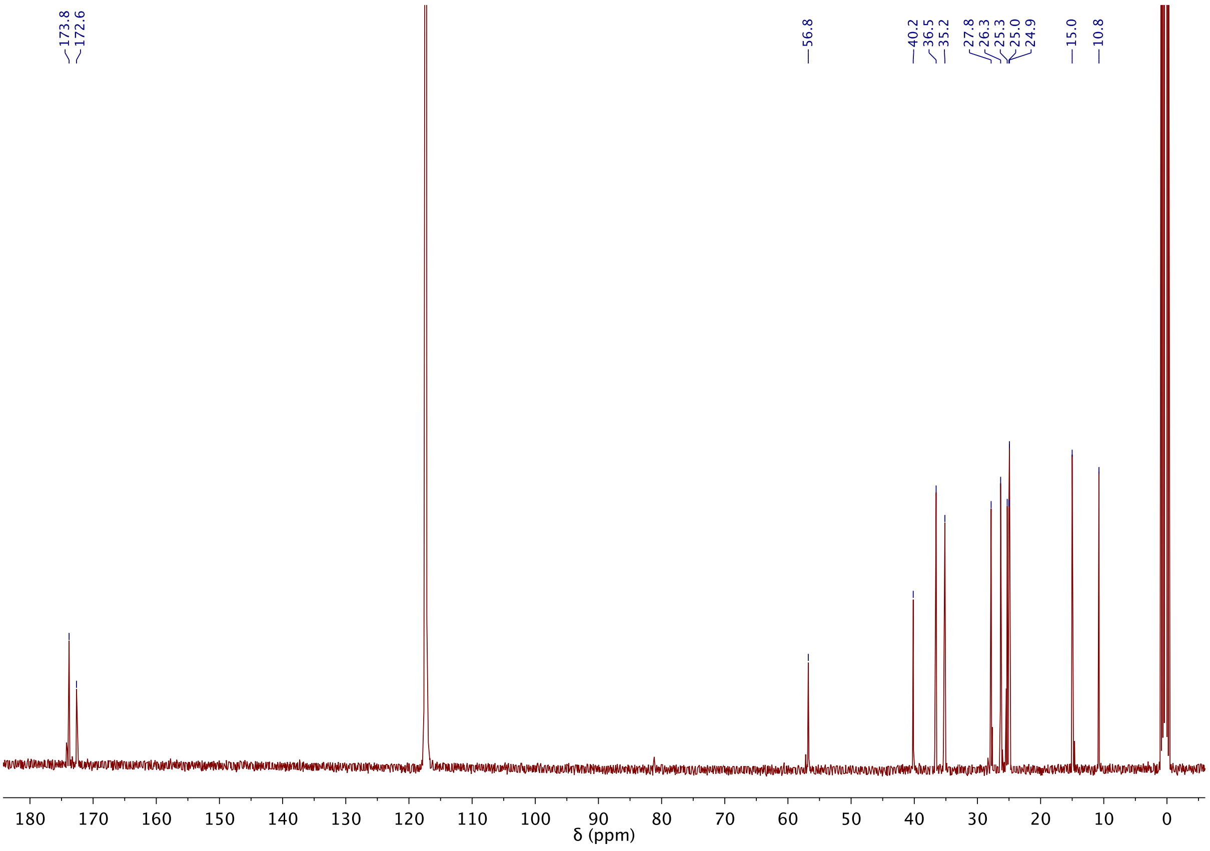

173.8
172.6
56.8
40.2
36.5
35.2
27.8
26.3
25.3
25.0
24.9
15.0
10.8
δ (ppm)

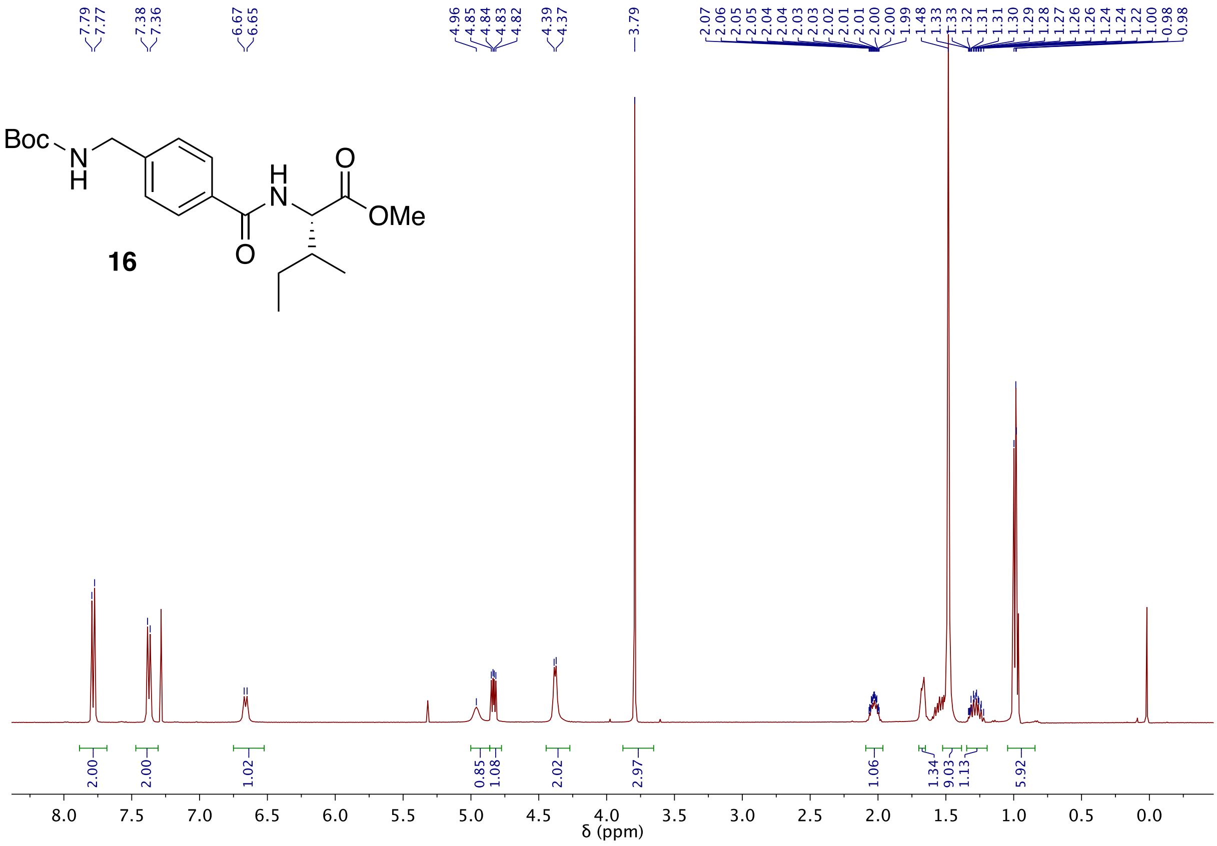

16
7.79
7.77
7.38
7.36
6.67
6.65
4.96
4.85
4.84
4.83
4.82
4.39
4.37
3.79
2.07
2.06
2.05
2.05
2.04
2.04
2.03
2.03
2.02
2.01
2.00
2.00
1.99
1.48
1.33
1.33
1.32
1.31
1.31
1.30
1.29
1.28
1.27
1.26
1.26
1.24
1.24
1.22
1.00
0.98
0.98
2.00
2.00
1.02
0.85
1.08
2.02
2.97
1.06
1.34
9.03
1.13
5.92
8.0 7.5 7.0 6.5 6.0 5.5 5.0 4.5 4.0 3.5 3.0 2.5 2.0 1.5 1.0 0.5 0.0
δ (ppm)

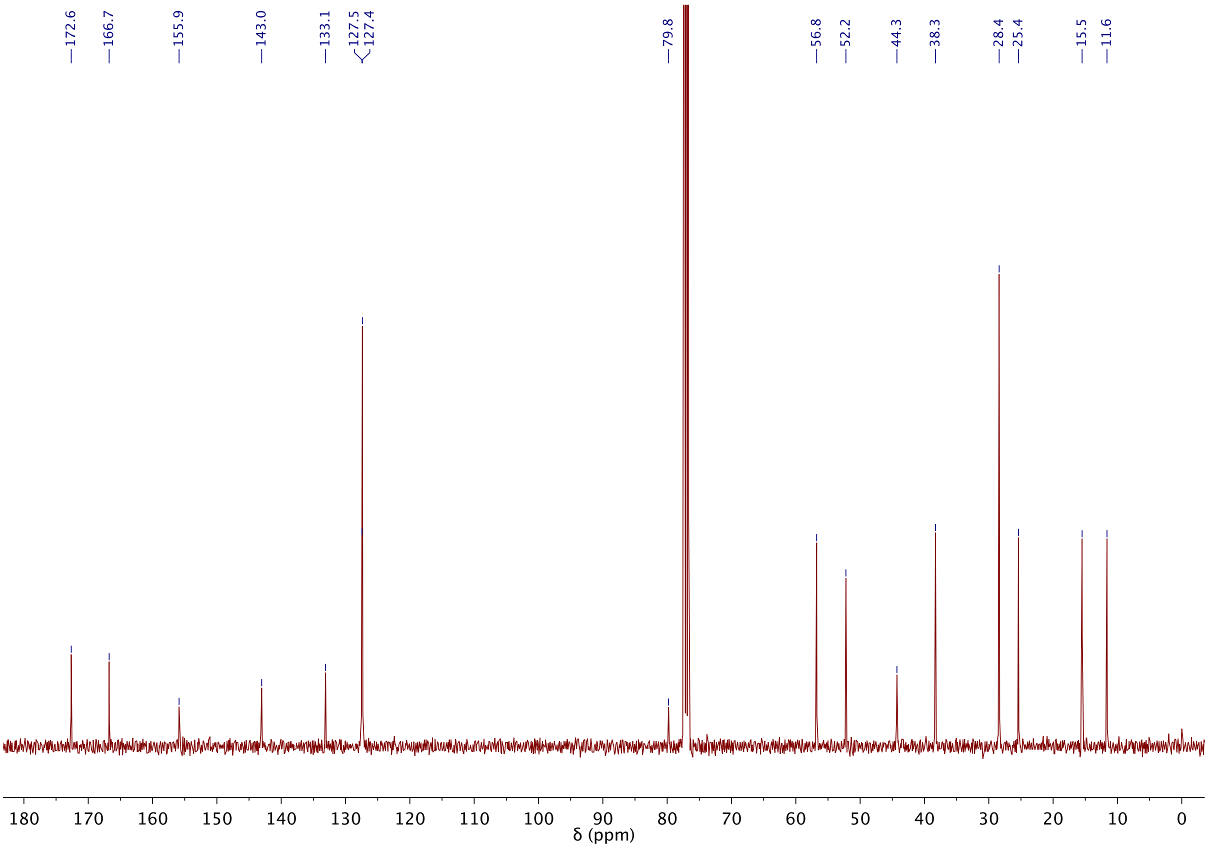
172.6
166.7
155.9
143.0
133.1
127.5
127.4
79.8
56.8
52.2
44.3
38.3
28.4
25.4
15.5
11.6
180 170 160 150 140 130 120 110 100 90 80 70 60 50 40 30 20 10 0
δ (ppm)

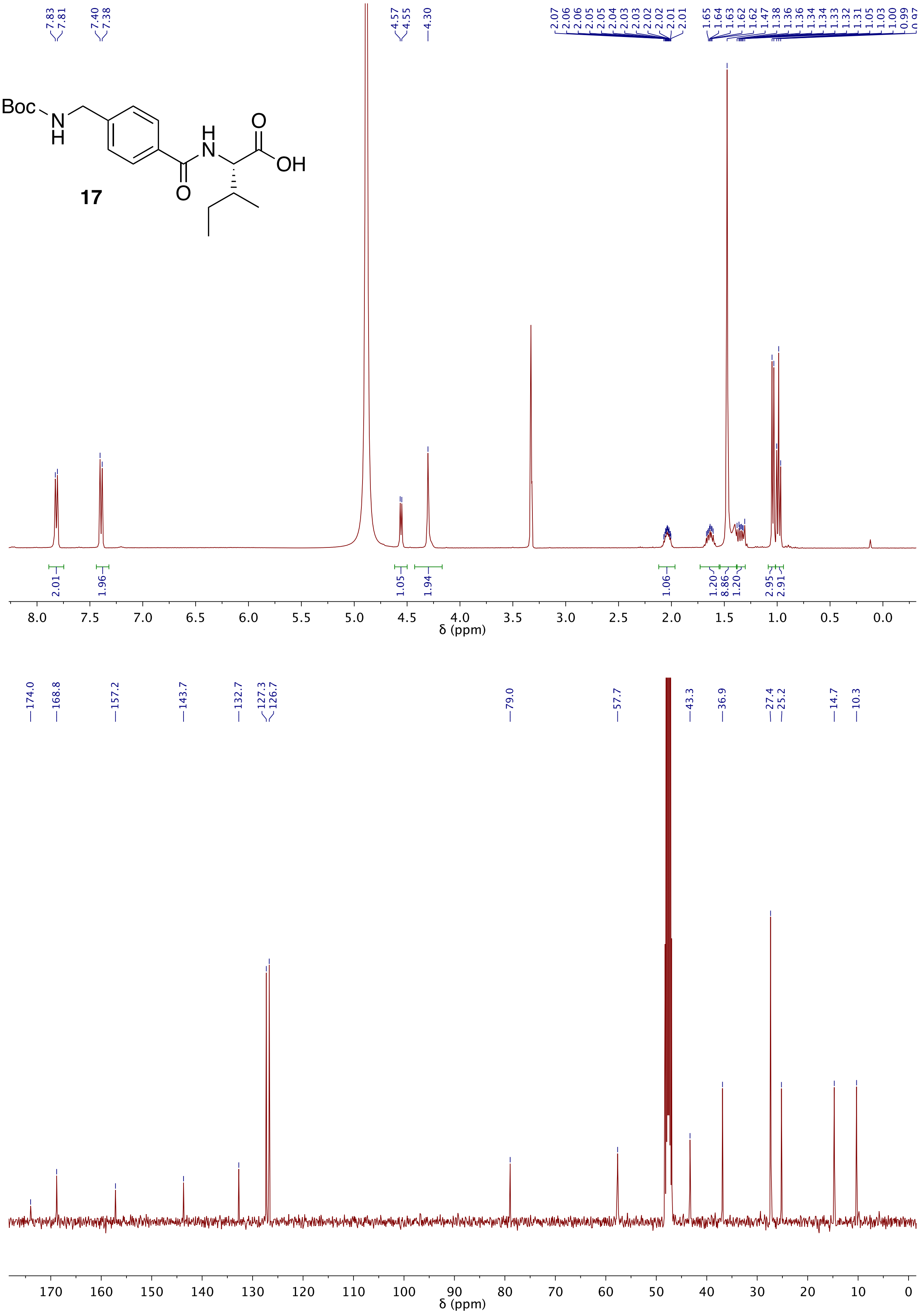
Boc
N
H
H
N
O
OH
O
17
7.83
7.81
7.40
7.38
4.57
4.55
4.30
2.07
2.06
2.05
2.05
2.04
2.03
2.03
2.02
2.02
2.01
2.01
1.65
1.64
1.63
1.62
1.62
1.47
1.38
1.36
1.36
1.34
1.34
1.33
1.32
1.31
1.05
1.03
1.00
0.99
2.01
1.96
1.05
1.94
1.06
1.20
8.86
1.20
2.95
2.91
8.0 7.5 7.0 6.5 6.0 5.5 5.0 4.5 4.0 3.5 3.0 2.5 2.0 1.5 1.0 0.5 0.0
δ (ppm)
174.0
168.8
157.2
143.7
132.7
127.3
126.7
79.0
57.7
43.3
36.9
27.4
25.2
14.7
10.3
170 160 150 140 130 120 110 100 90 80 70 60 50 40 30 20 10 0
δ (ppm)

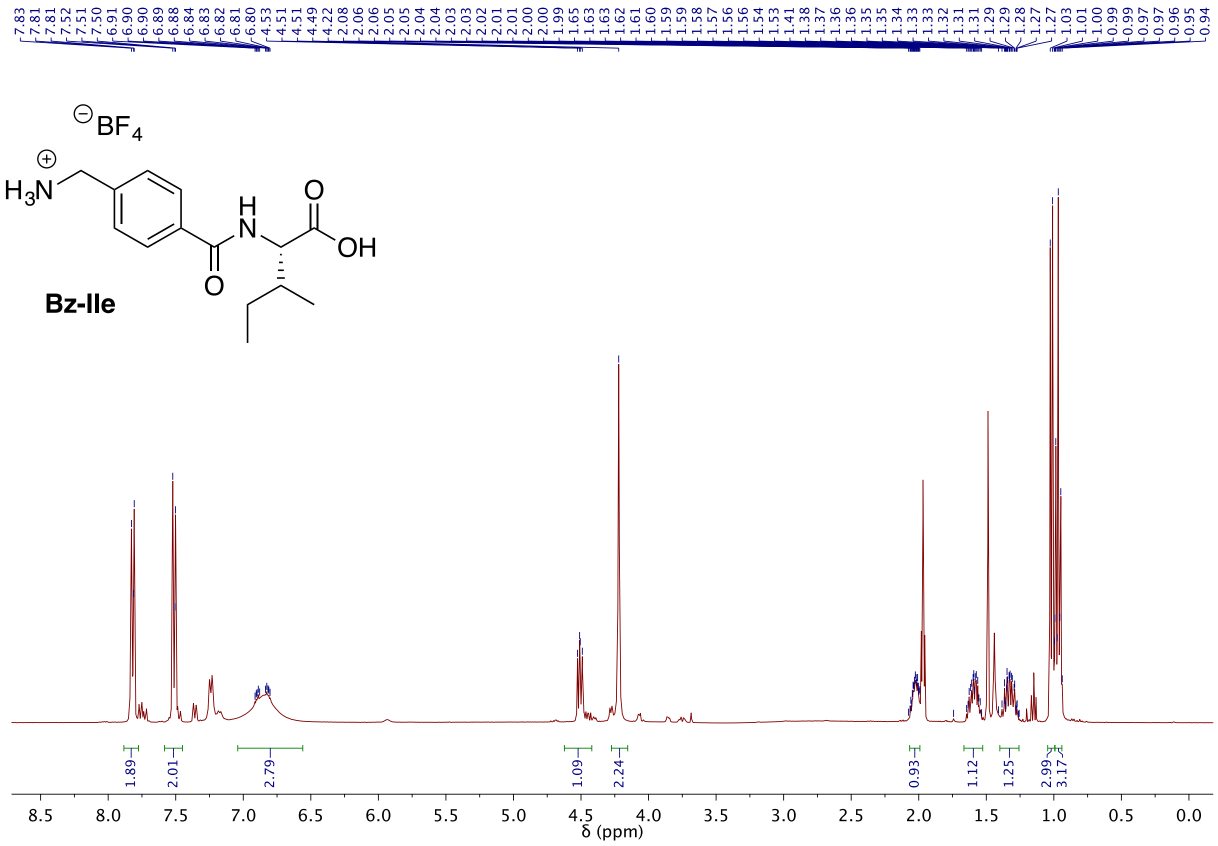
7.83 7.81 7.81 7.52 7.51 7.50 6.91 6.90 6.90 6.89 6.88 6.84 6.83 6.82 6.81 6.80 4.53 4.51 4.51 4.49 4.22 2.08 2.06 2.06 2.05 2.05 2.04 2.04 2.03 2.03 2.02 2.01 2.01 2.00 2.00 1.99 1.65 1.63 1.63 1.62 1.61 1.60 1.59 1.59 1.58 1.57 1.56 1.56 1.54 1.53 1.41 1.38 1.37 1.36 1.36 1.35 1.35 1.34 1.33 1.33 1.33 1.32 1.31 1.31 1.31 1.29 1.29 1.28 1.27 1.27 1.03 1.01 1.01 1.00 0.99 0.99 0.97 0.97 0.96 0.95 0.94
BF4
H3N
H
N
O
OH
Bz-Ile
1.89 2.01 2.79 1.09 2.24 0.93 1.12 1.25 2.99 3.17
8.5 8.0 7.5 7.0 6.5 6.0 5.5 5.0 4.5 4.0 3.5 3.0 2.5 2.0 1.5 1.0 0.5 0.0
δ (ppm)

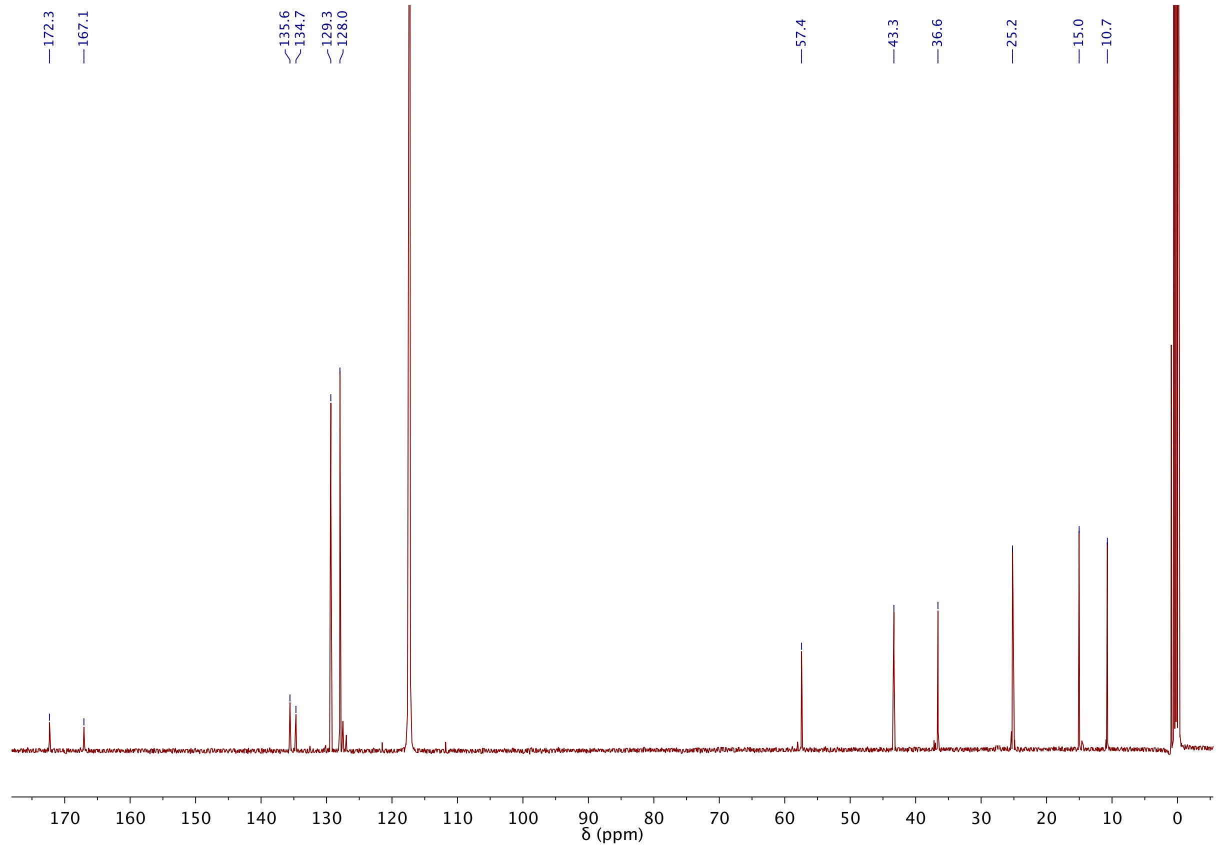
172.3 167.1 135.6 134.7 129.3 128.0 57.4 43.3 36.6 25.2 15.0 10.7
170 160 150 140 130 120 110 100 90 80 70 60 50 40 30 20 10 0
δ (ppm)

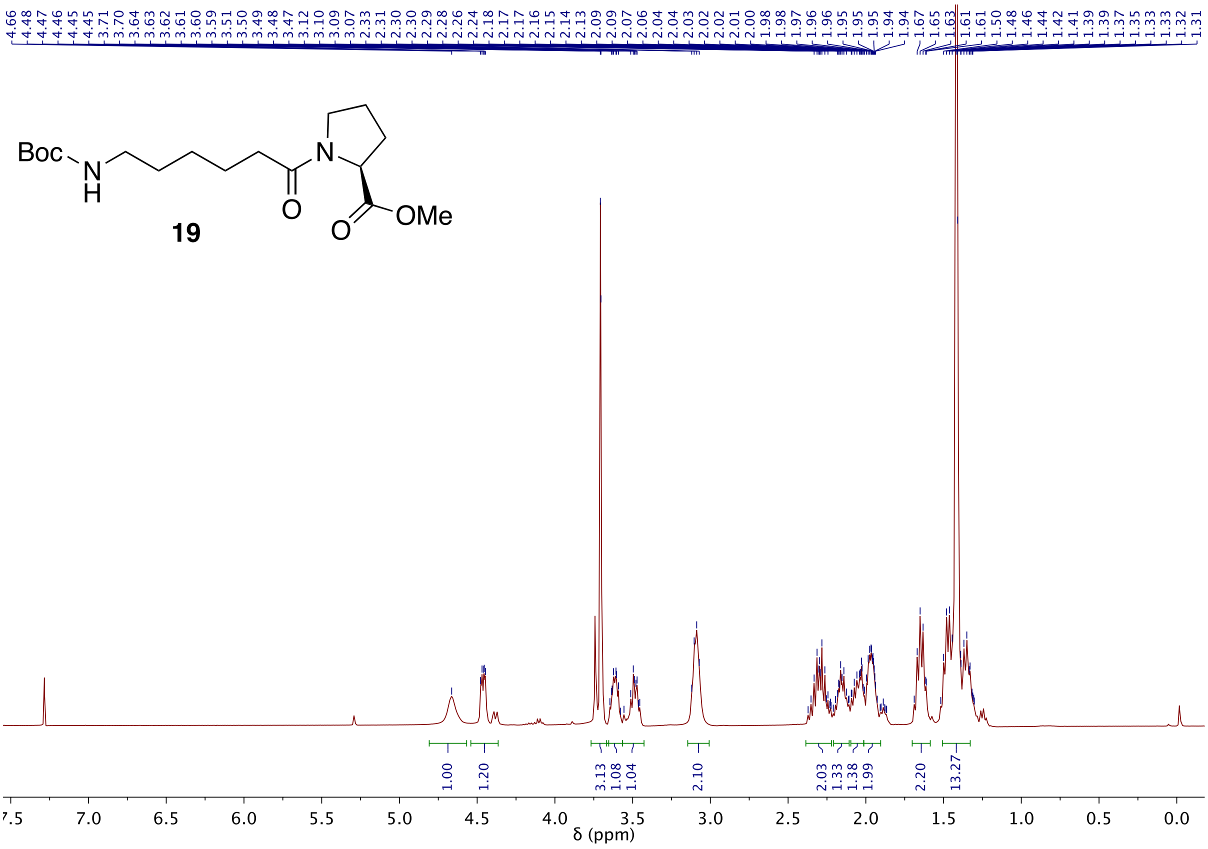
Boc
N
H
O
OMe
19
δ (ppm)

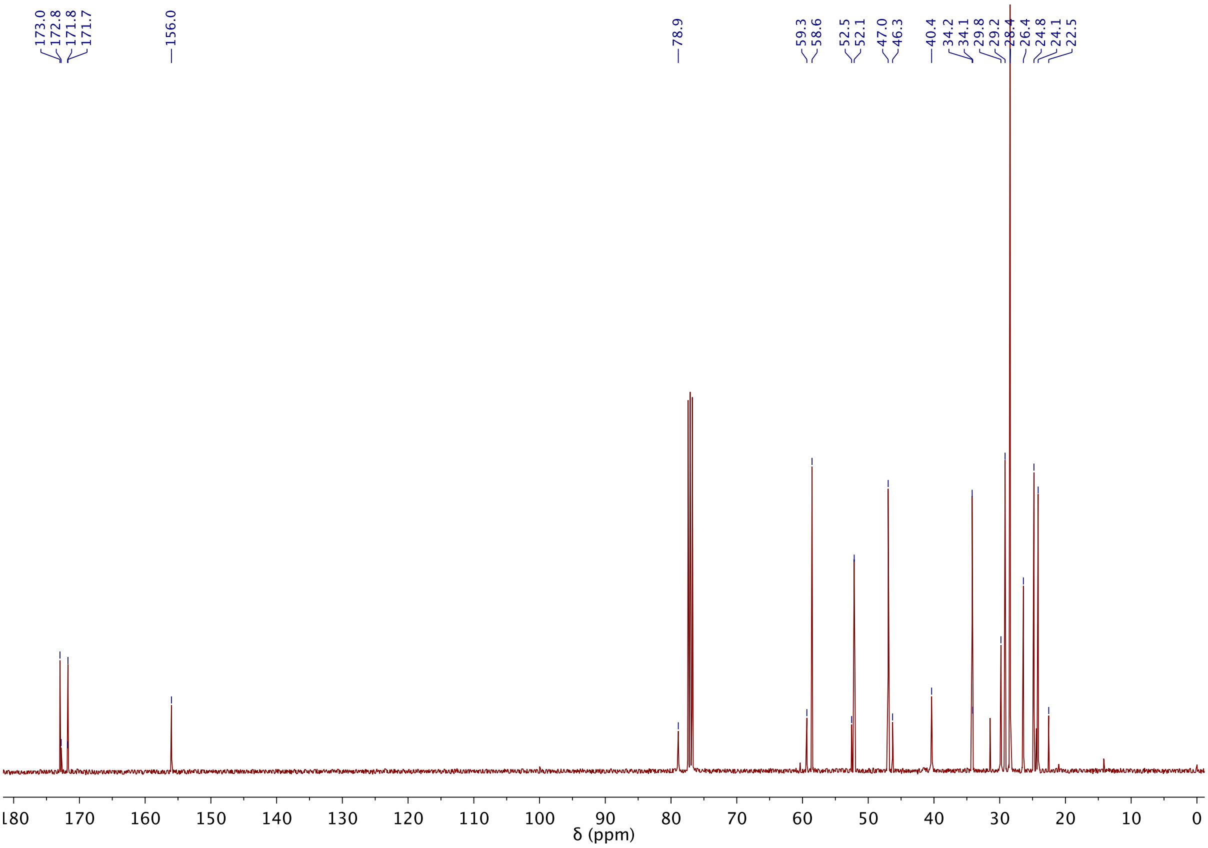
173.0
172.8
171.8
171.7
156.0
78.9
59.3
58.6
52.5
52.1
47.0
46.3
40.4
34.2
34.1
29.8
29.2
28.4
26.4
24.8
24.1
22.5
δ (ppm)

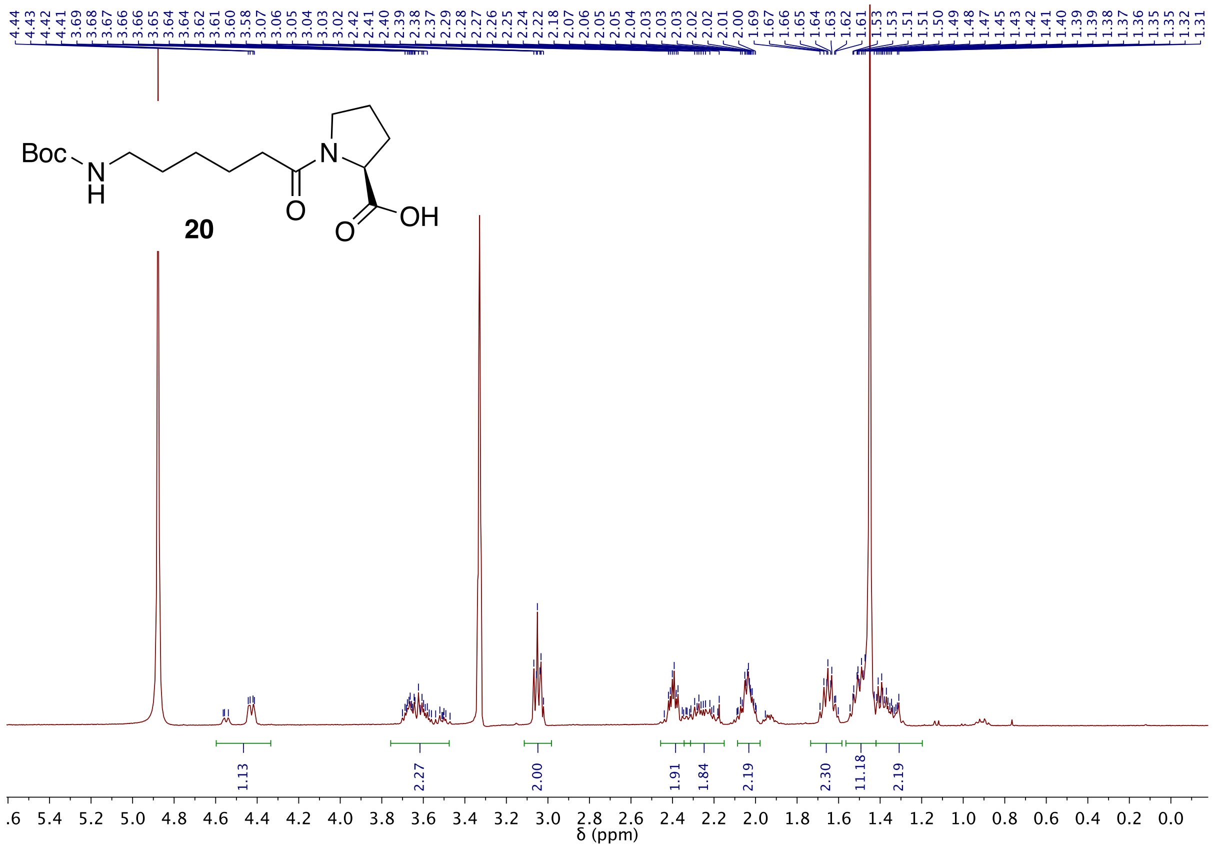

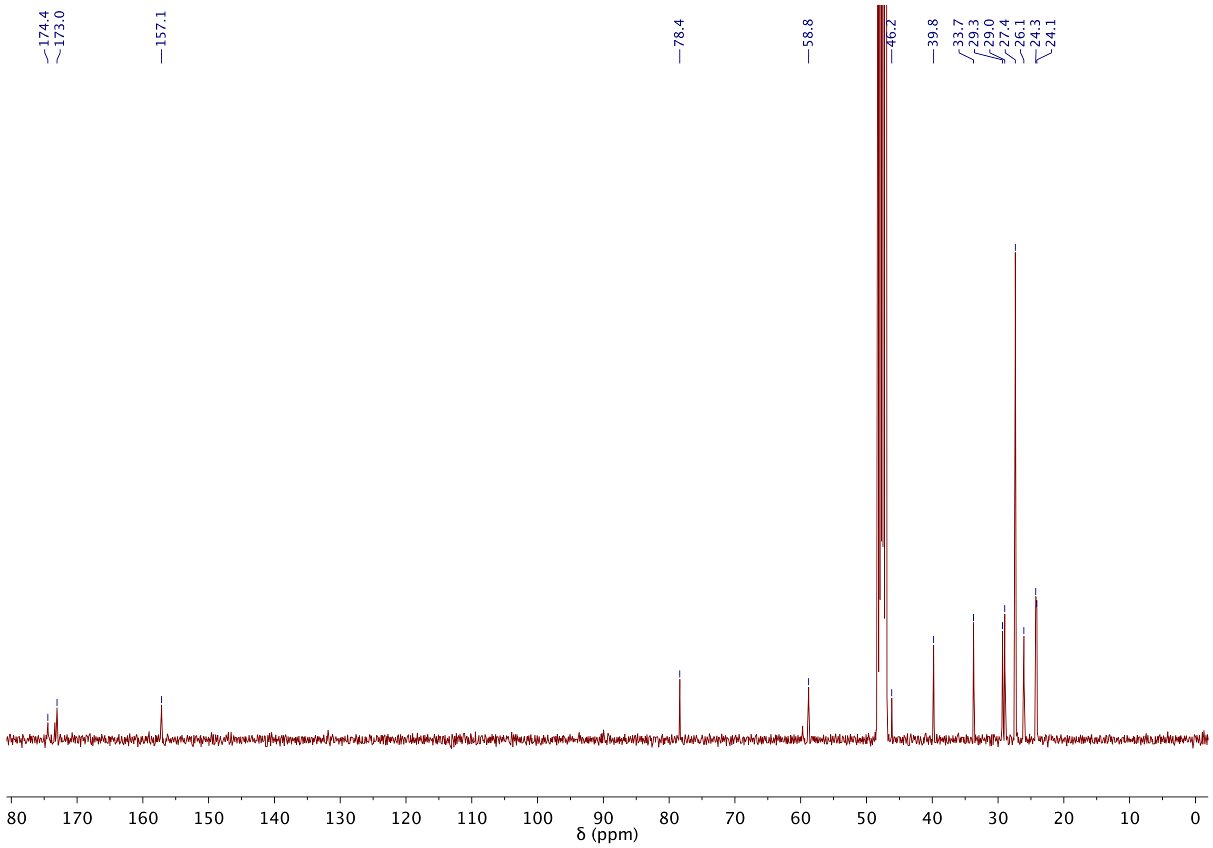

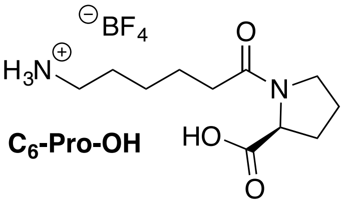

C$_6$-Pro-OH

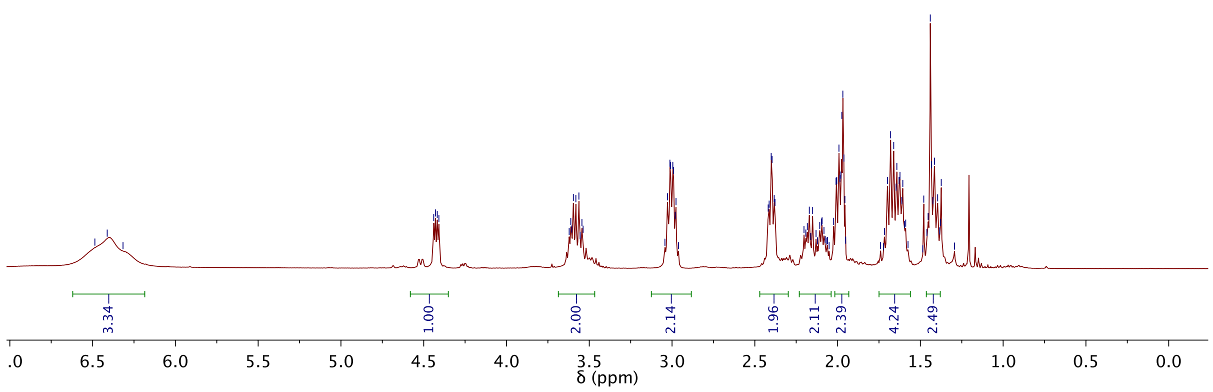

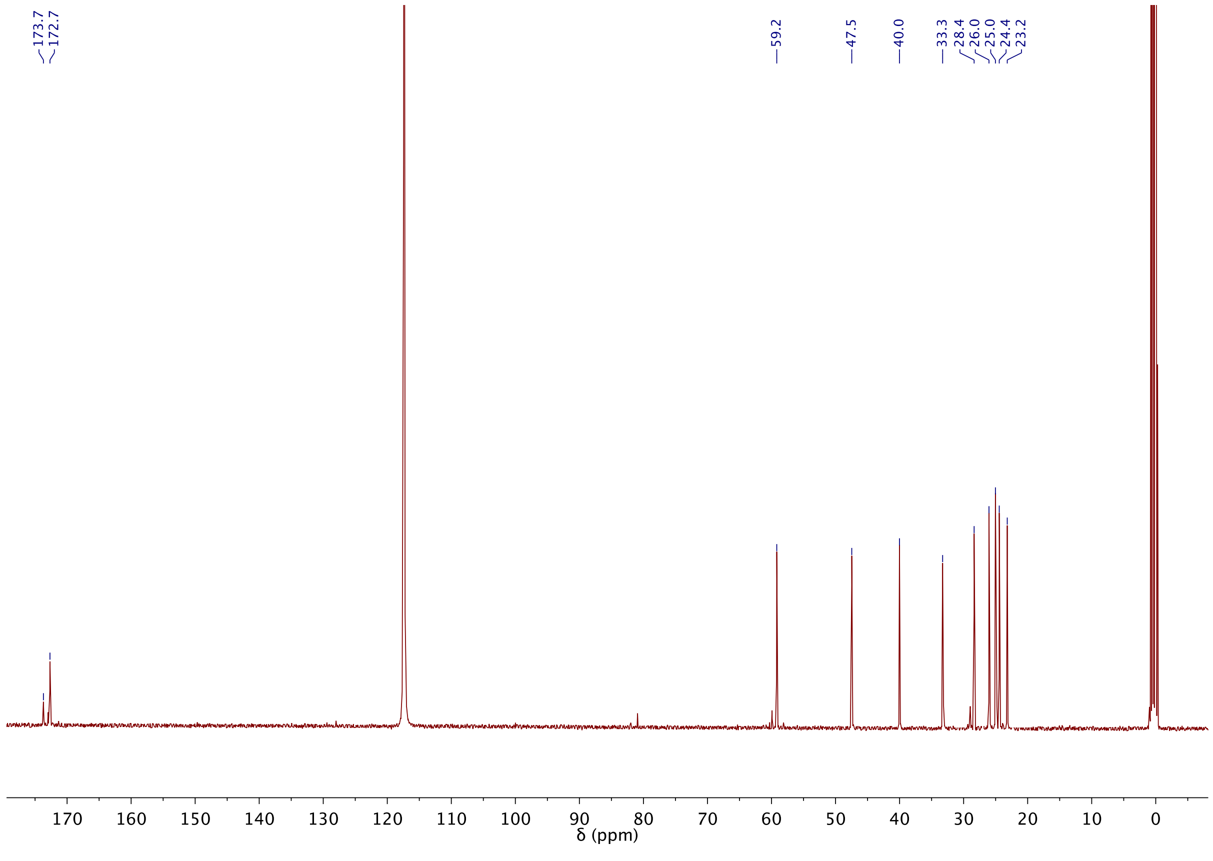

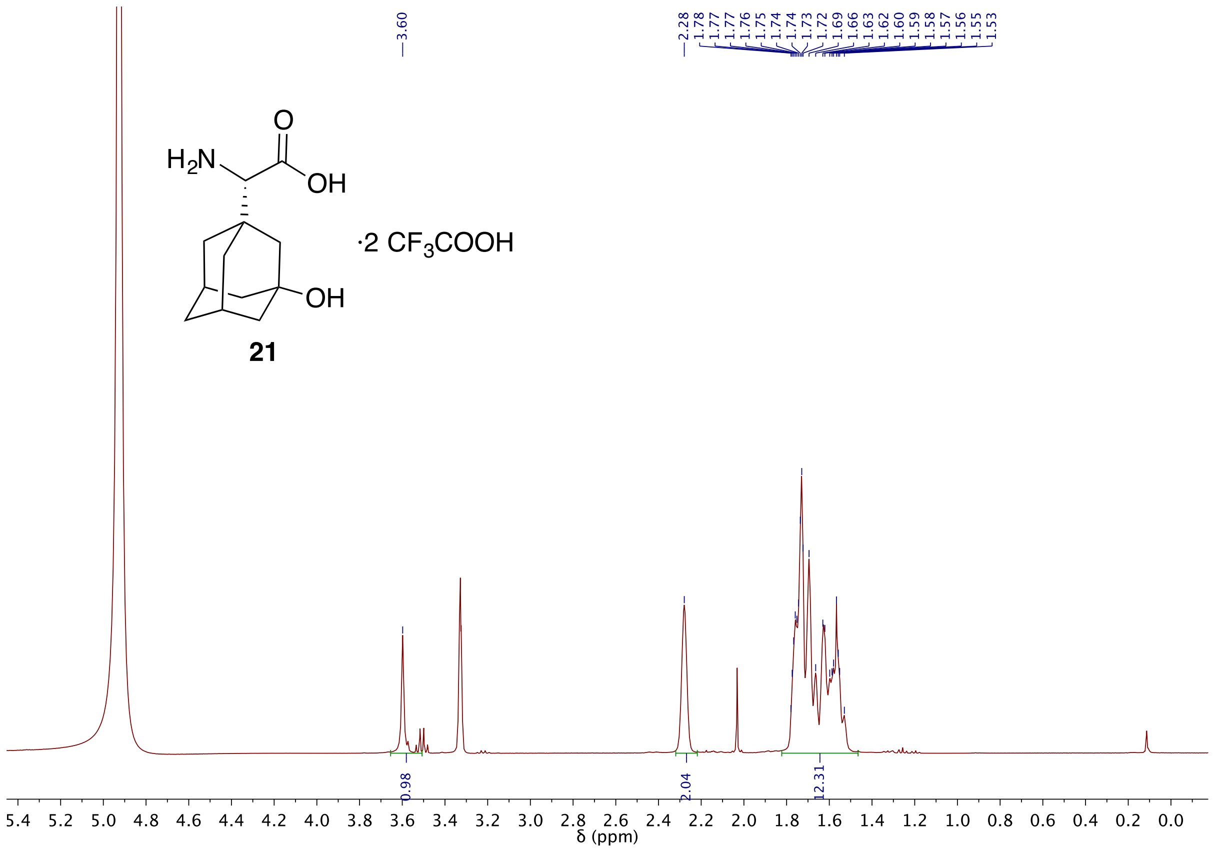
3.60
2.28
1.78
1.77
1.77
1.76
1.75
1.74
1.74
1.73
1.72
1.69
1.66
1.63
1.62
1.60
1.59
1.58
1.57
1.56
1.55
1.53
O
H2N
OH
·2 CF3COOH
OH
21
0.98
2.04
12.31
5.4 5.2 5.0 4.8 4.6 4.4 4.2 4.0 3.8 3.6 3.4 3.2 3.0 2.8 2.6 2.4 2.2 2.0 1.8 1.6 1.4 1.2 1.0 0.8 0.6 0.4 0.2 0.0
δ (ppm)

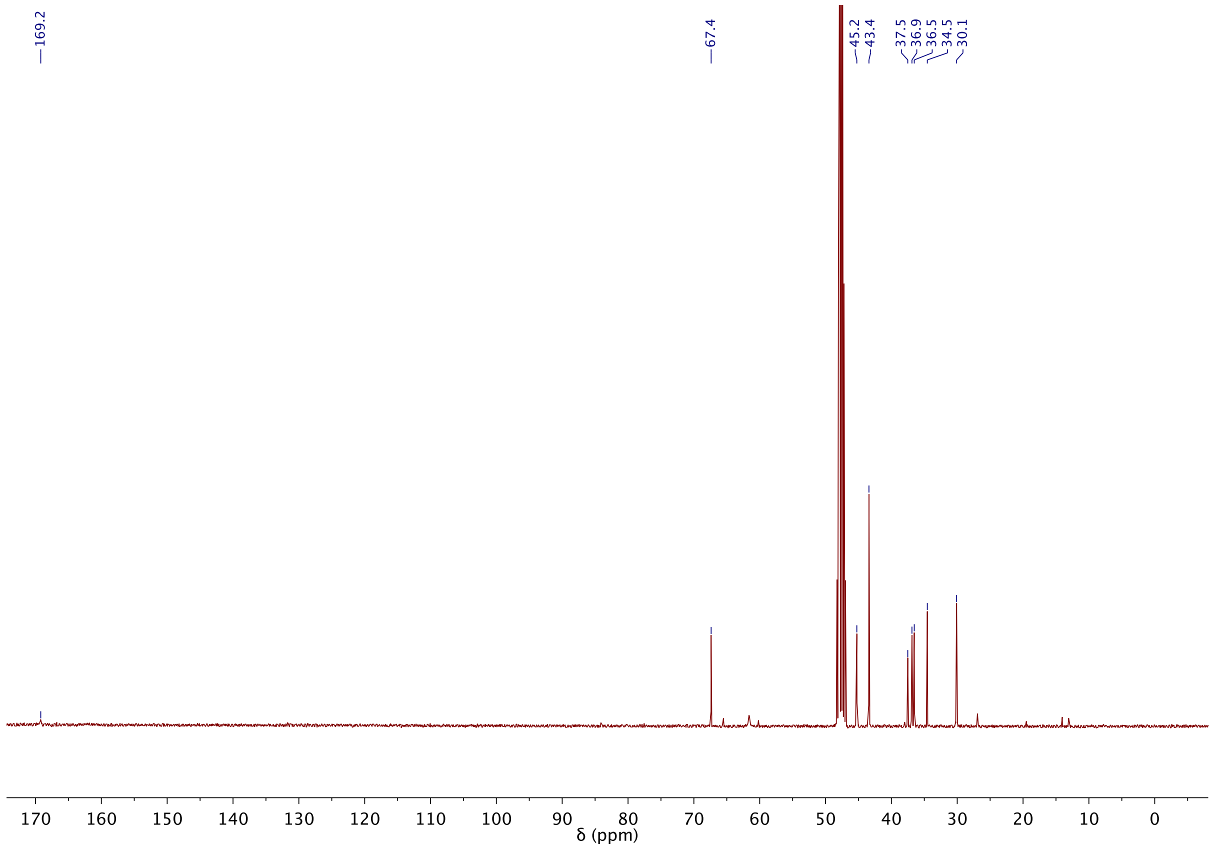
169.2
67.4
45.2
43.4
37.5
36.9
36.5
34.5
30.1
170 160 150 140 130 120 110 100 90 80 70 60 50 40 30 20 10 0
δ (ppm)

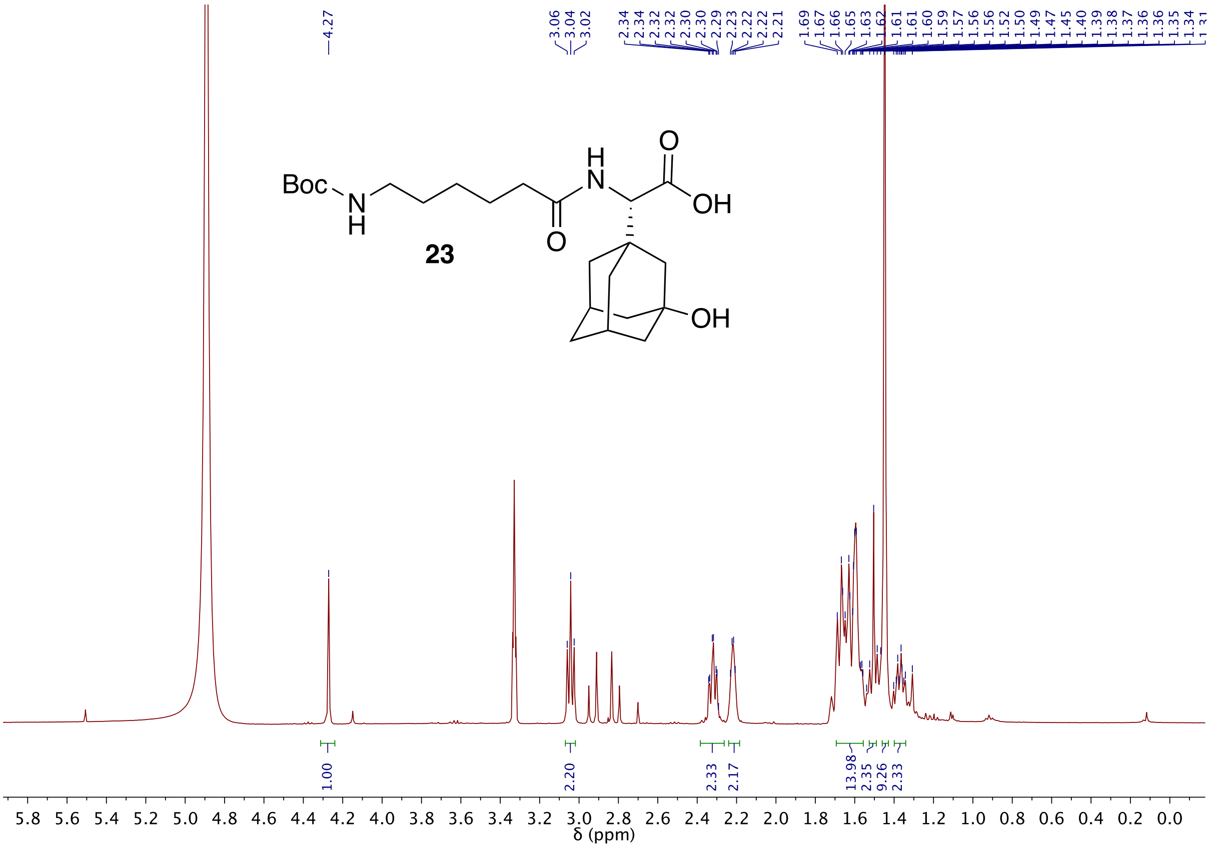
Boc
N
H
H
N
O
O
OH
OH
23
δ (ppm)

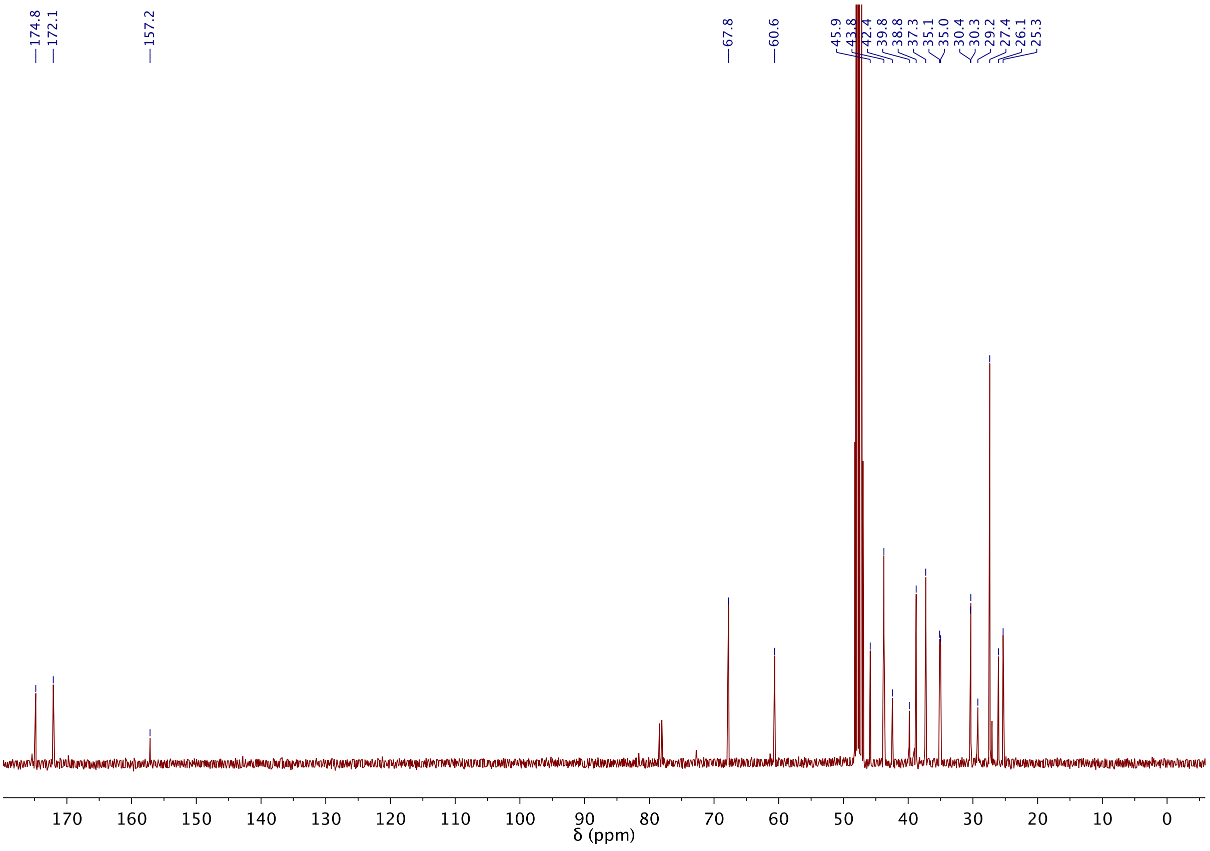
δ (ppm)

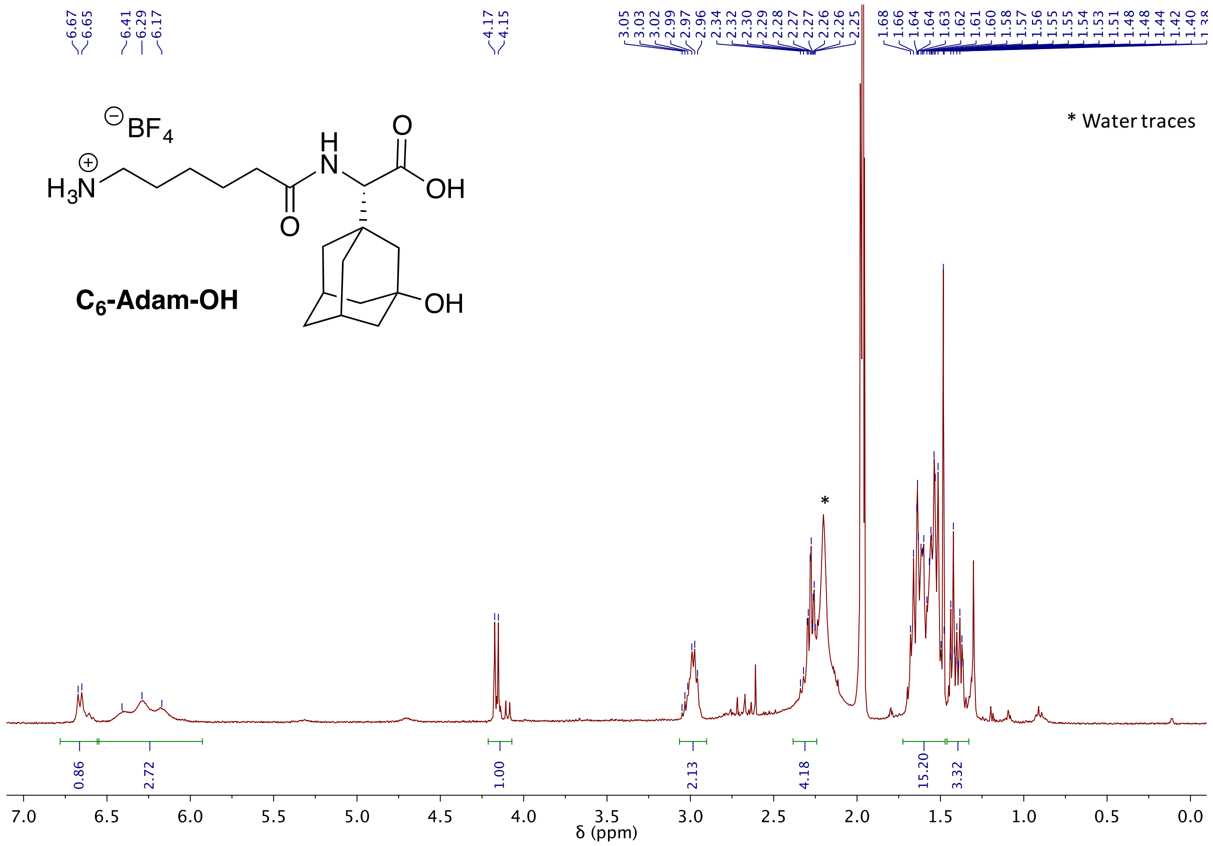
C6-Adam-OH
BF4
H3N
OH
* Water traces
6.67
6.65
6.41
6.29
6.17
4.17
4.15
0.86
2.72
1.00
2.13
4.18
15.20
3.32
δ (ppm)

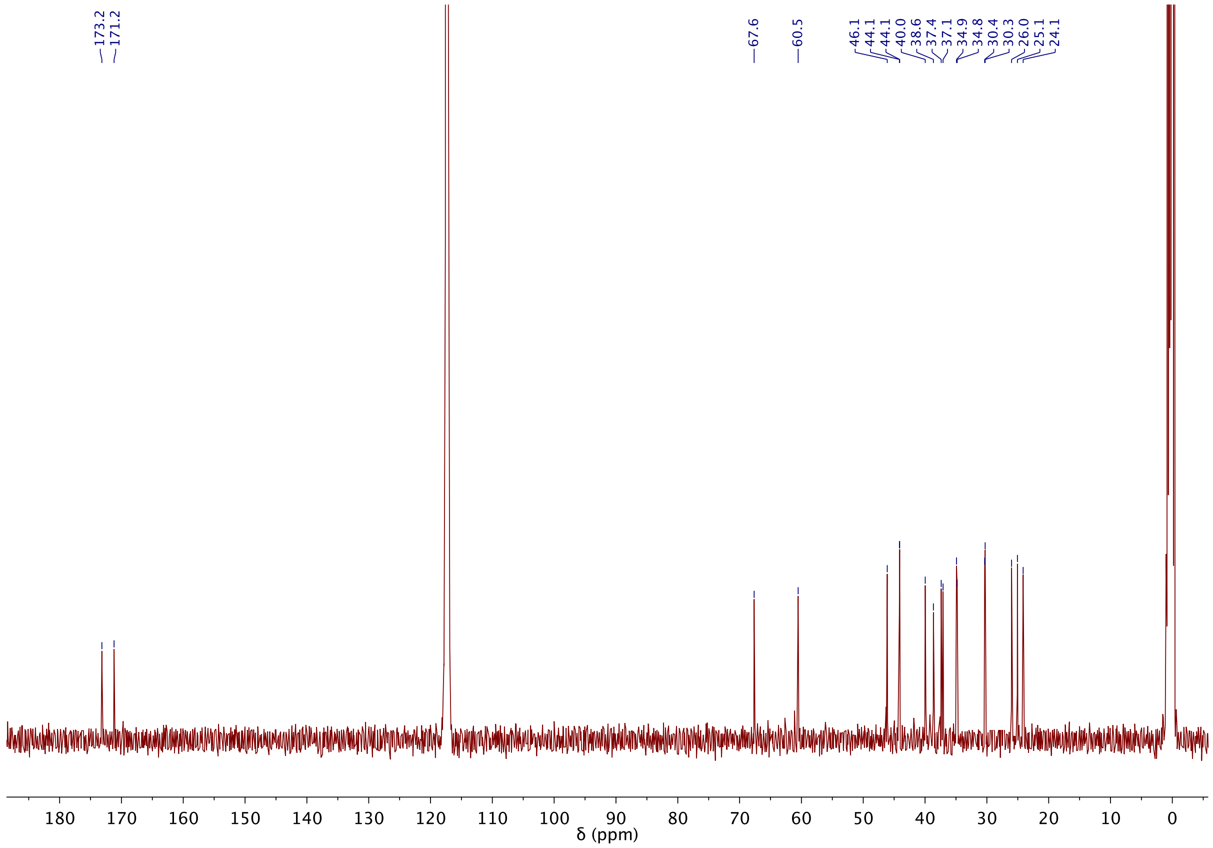
173.2
171.2
67.6
60.5
46.1
44.1
44.1
40.0
38.6
37.4
37.1
34.9
34.8
30.4
30.3
26.0
25.1
24.1
δ (ppm)

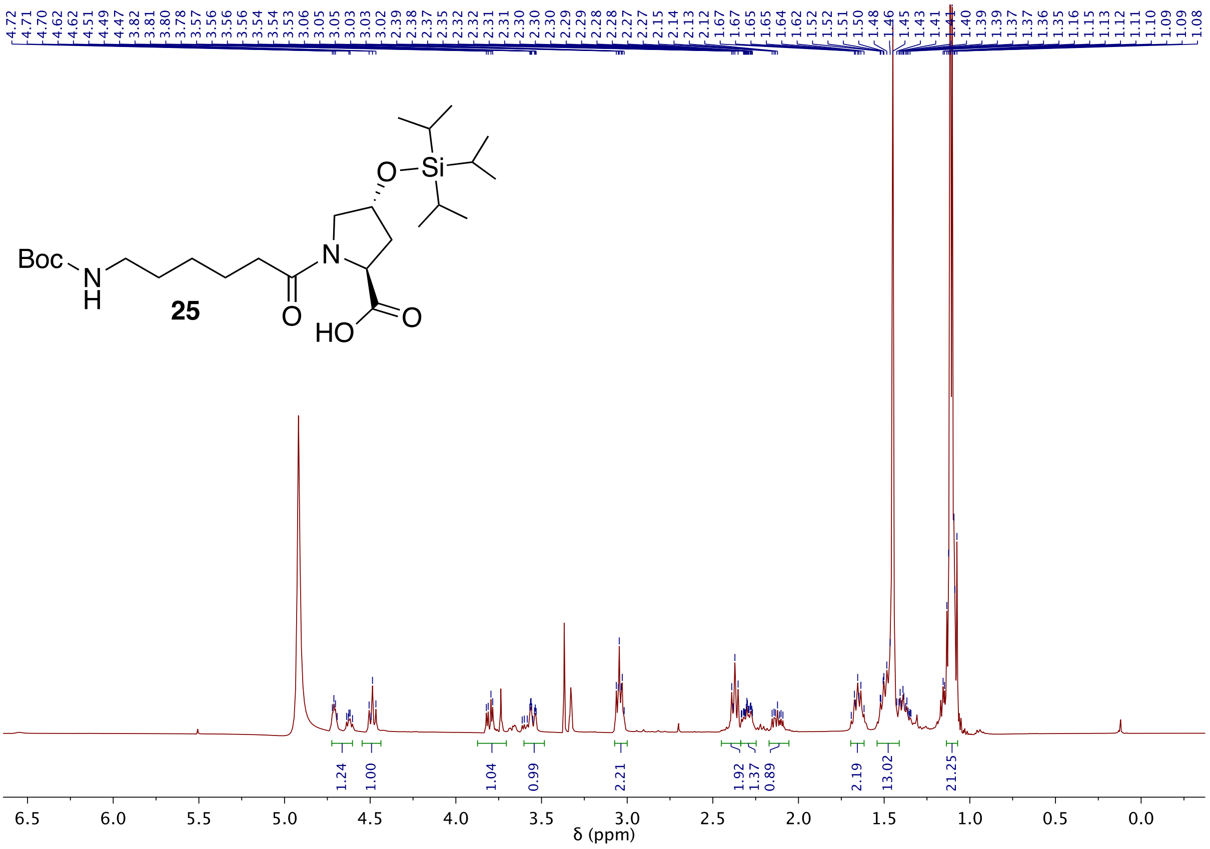
4.72 4.71 4.70 4.62 4.62 4.51 4.49 4.47 3.82 3.81 3.80 3.78 3.57 3.56 3.56 3.56 3.54 3.54 3.53 3.06 3.05 3.05 3.03 3.03 3.02 2.39 2.38 2.37 2.35 2.32 2.32 2.31 2.31 2.30 2.30 2.30 2.29 2.29 2.28 2.28 2.27 2.27 2.15 2.14 2.13 2.12 1.67 1.67 1.65 1.65 1.64 1.62 1.52 1.52 1.51 1.50 1.48 1.46 1.45 1.43 1.41 1.41 1.40 1.39 1.39 1.37 1.37 1.36 1.35 1.16 1.15 1.13 1.12 1.11 1.10 1.09 1.09 1.08
Boc
N
H
25
O
N
O
Si
HO
O
1.24
1.00
1.04
0.99
2.21
1.92
1.37
0.89
2.19
13.02
21.25
6.5 6.0 5.5 5.0 4.5 4.0 3.5 3.0 2.5 2.0 1.5 1.0 0.5 0.0
δ (ppm)

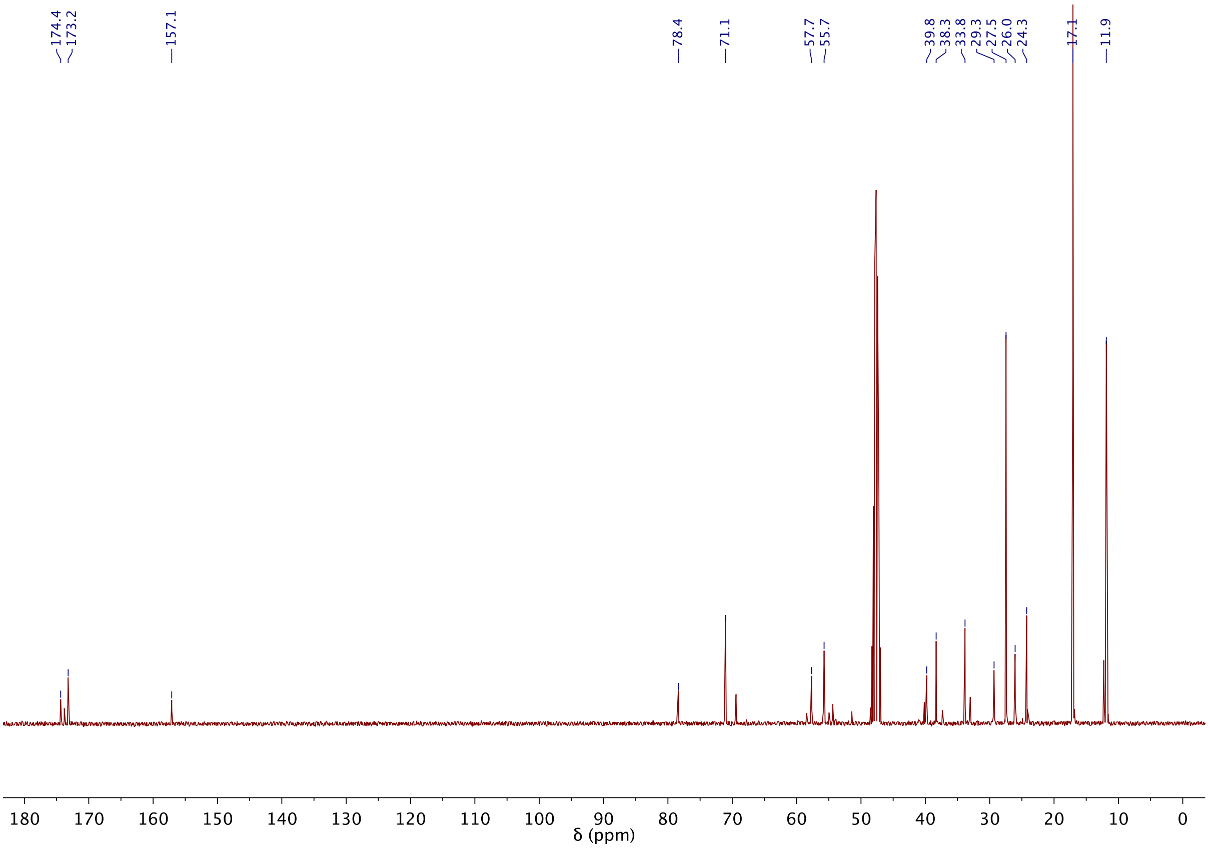
174.4
173.2
157.1
78.4
71.1
57.7
55.7
39.8
38.3
33.8
29.3
27.5
26.0
24.3
17.1
11.9
180 170 160 150 140 130 120 110 100 90 80 70 60 50 40 30 20 10 0
δ (ppm)

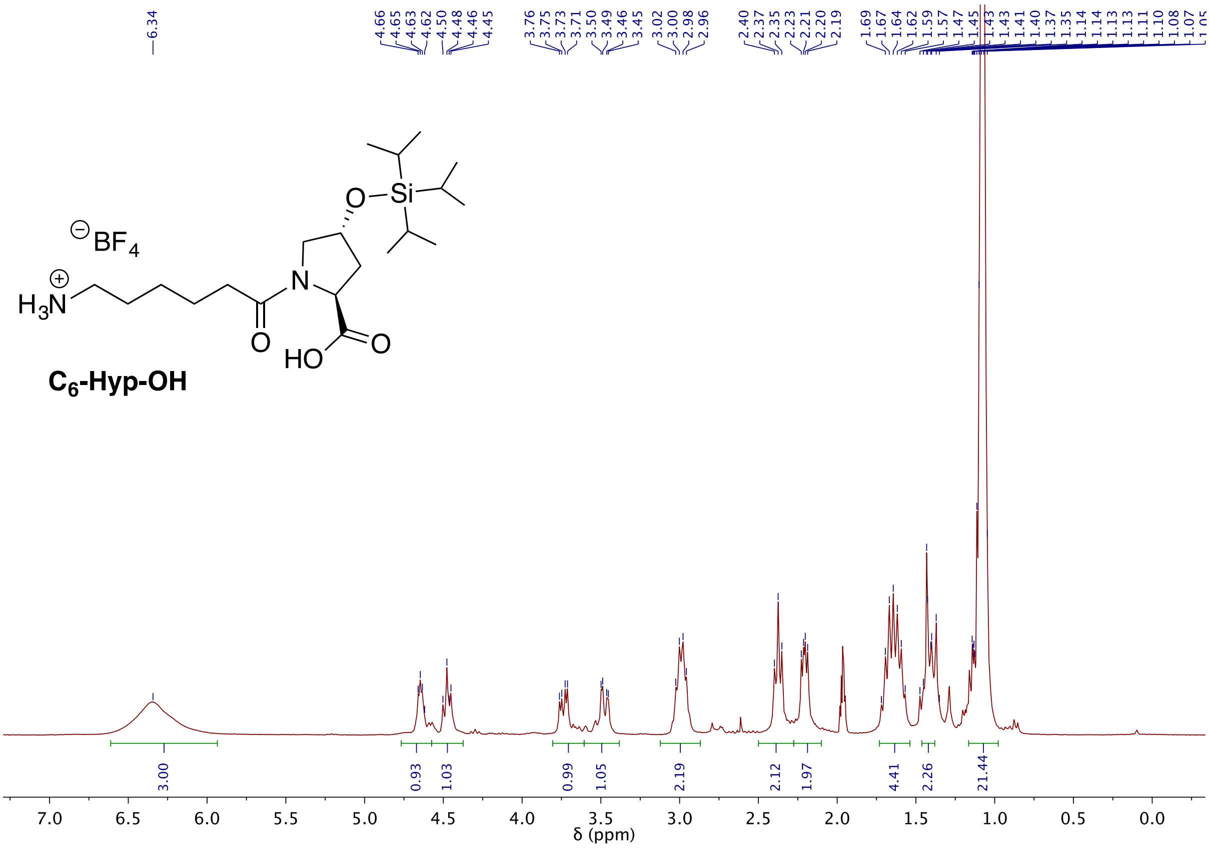
C$_6$-Hyp-OH
3.00
0.93
1.03
0.99
1.05
2.19
2.12
1.97
4.41
2.26
21.44
δ (ppm)

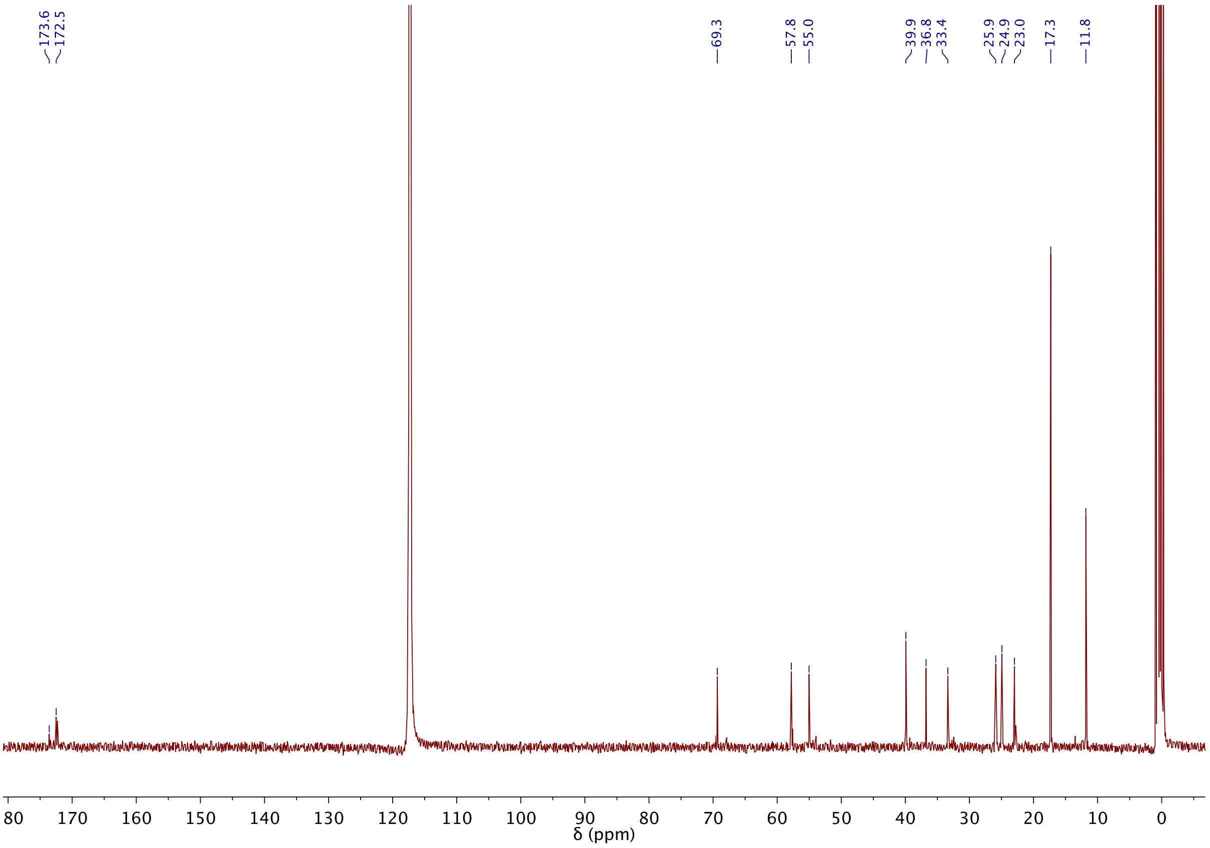
173.6
172.5
69.3
57.8
55.0
39.9
36.8
33.4
25.9
24.9
23.0
17.3
11.8
δ (ppm)

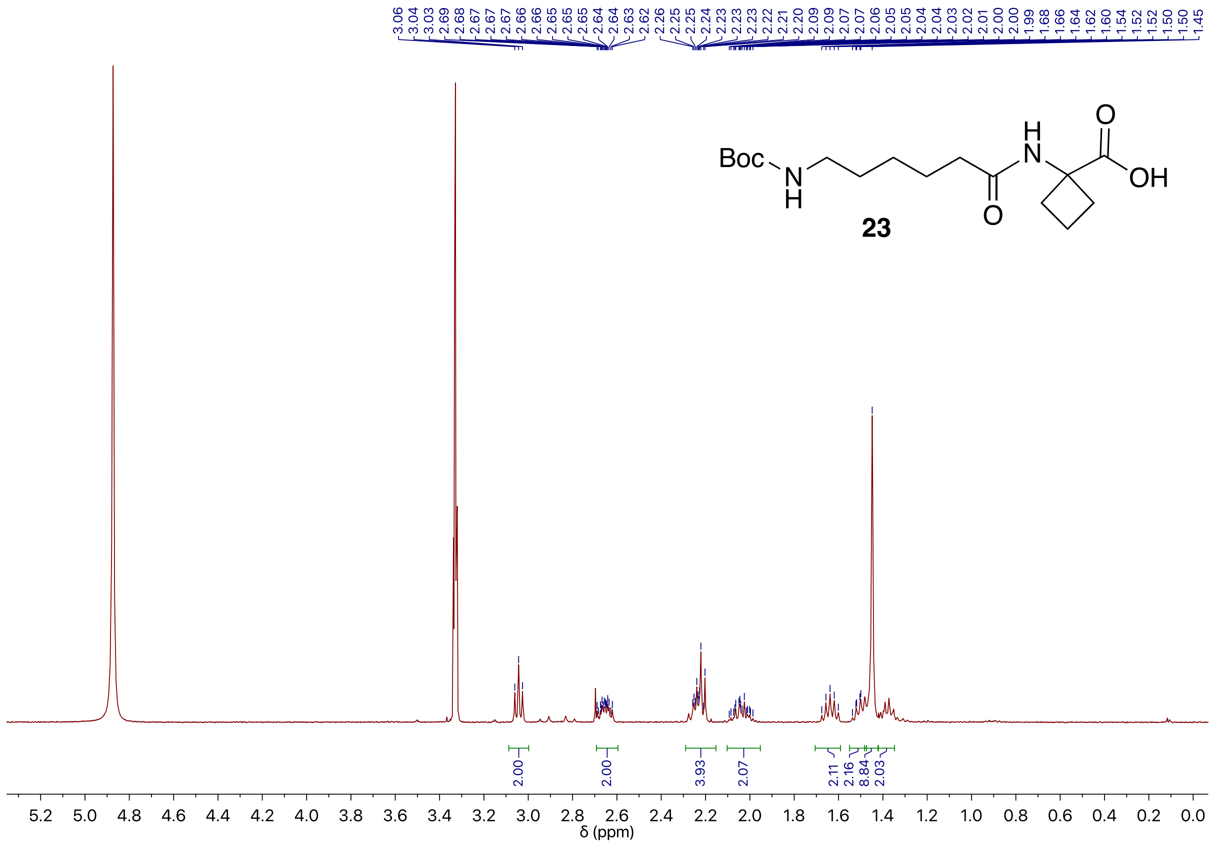
3.06 3.04 3.03 2.69 2.68 2.67 2.67 2.66 2.66 2.65 2.65 2.65 2.64 2.64 2.63 2.62 2.26 2.25 2.25 2.24 2.23 2.23 2.23 2.22 2.21 2.20 2.09 2.09 2.07 2.07 2.06 2.05 2.05 2.04 2.04 2.03 2.03 2.02 2.01 2.00 1.99 1.68 1.66 1.64 1.62 1.60 1.54 1.52 1.52 1.50 1.50 1.45
Boc
N
H
O
OH
23
2.00
2.00
3.93
2.07
2.11
2.16
8.84
2.03
5.2 5.0 4.8 4.6 4.4 4.2 4.0 3.8 3.6 3.4 3.2 3.0 2.8 2.6 2.4 2.2 2.0 1.8 1.6 1.4 1.2 1.0 0.8 0.6 0.4 0.2 0.0
δ (ppm)

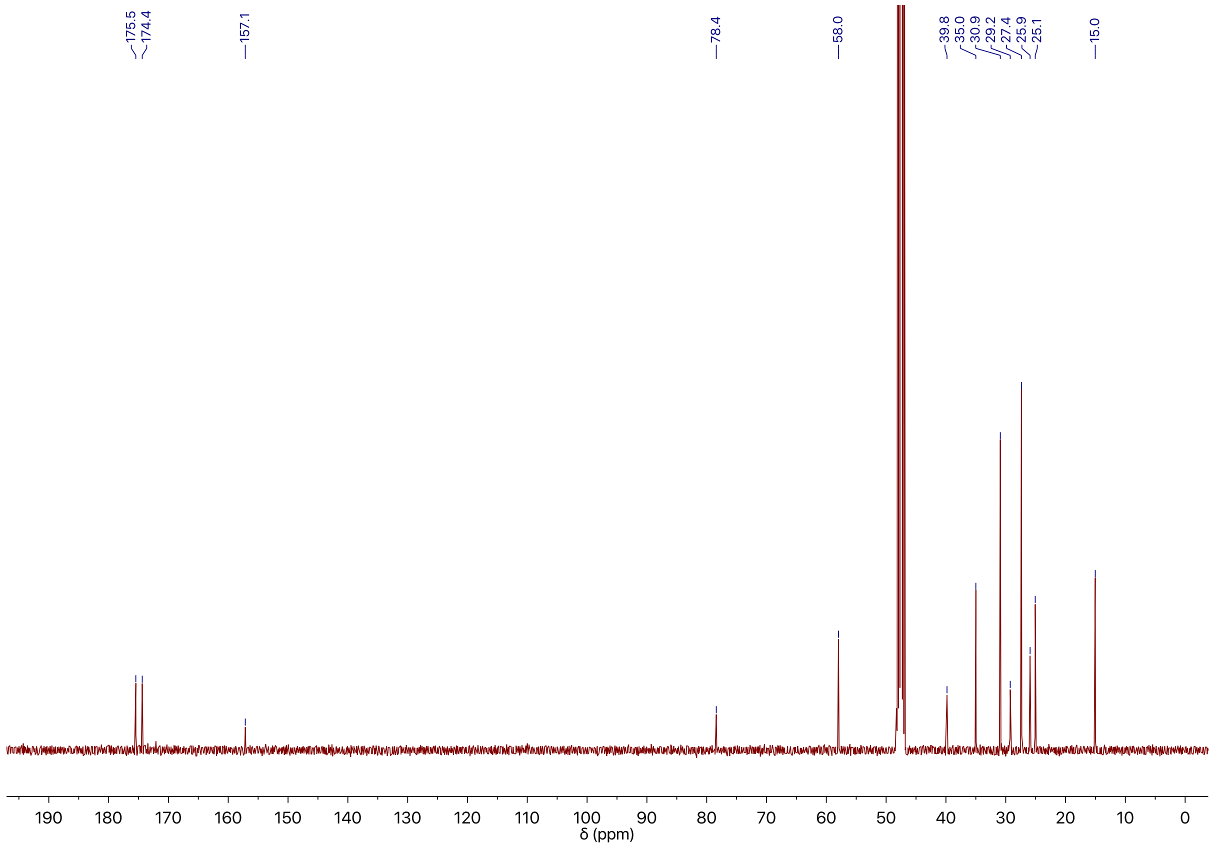
175.5 174.4
157.1
78.4
58.0
39.8 35.0 30.9 29.2 27.4 25.9 25.1
15.0
190 180 170 160 150 140 130 120 110 100 90 80 70 60 50 40 30 20 10 0
δ (ppm)

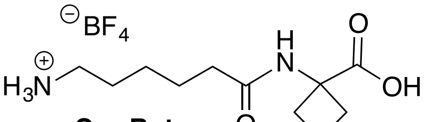
BF4
H3N
H
N
O
OH
O

C_6-cBut

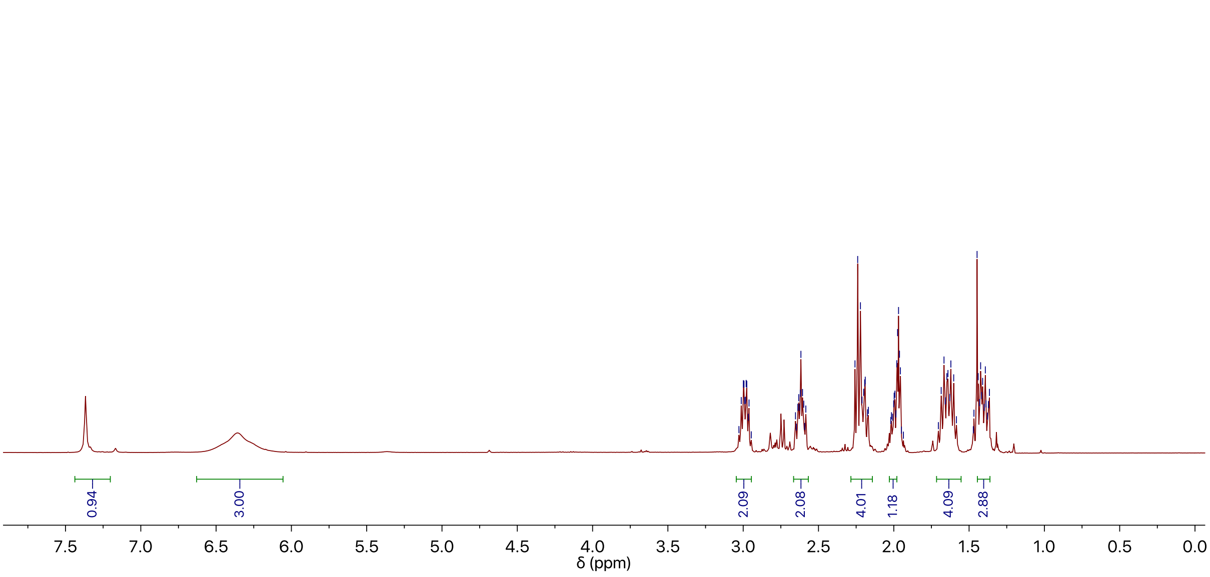
3.03 3.01 3.00 2.99 2.99 2.98 2.98 2.97 2.96 2.95 2.65 2.65 2.64 2.63 2.63 2.62 2.62 2.61 2.60 2.59 2.58 2.26 2.24 2.22 2.21 2.20 2.19 2.19 2.17 2.17 2.02 2.02 2.01 2.00 2.00 1.99 1.99 1.98 1.97 1.97 1.96 1.96 1.95 1.94 1.70 1.69 1.67 1.66 1.65 1.64 1.63 1.62 1.60 1.58 1.47 1.47 1.45 1.44 1.43 1.42 1.42 1.41 1.40 1.39 1.39 1.38 1.37 1.36
0.94
3.00
2.09
2.08
4.01
1.18
4.09
2.88
7.5 7.0 6.5 6.0 5.5 5.0 4.5 4.0 3.5 3.0 2.5 2.0 1.5 1.0 0.5 0.0
δ (ppm)

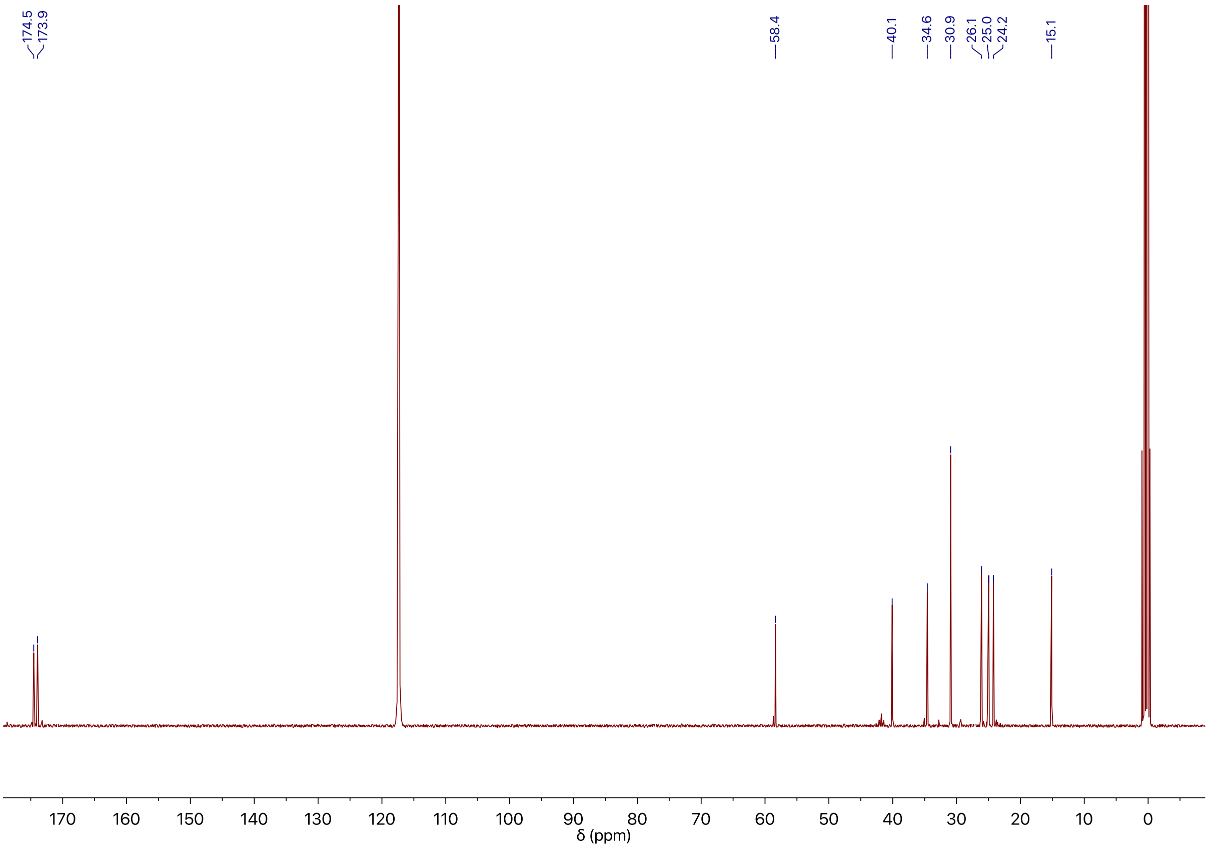
174.5
173.9
58.4
40.1
34.6
30.9
26.1
25.0
24.2
15.1
170 160 150 140 130 120 110 100 90 80 70 60 50 40 30 20 10 0
δ (ppm)

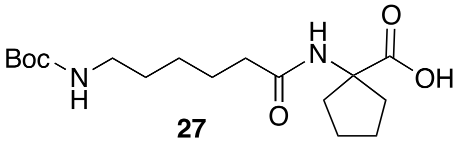
Boc
N
H
H
N
O
O
OH
27

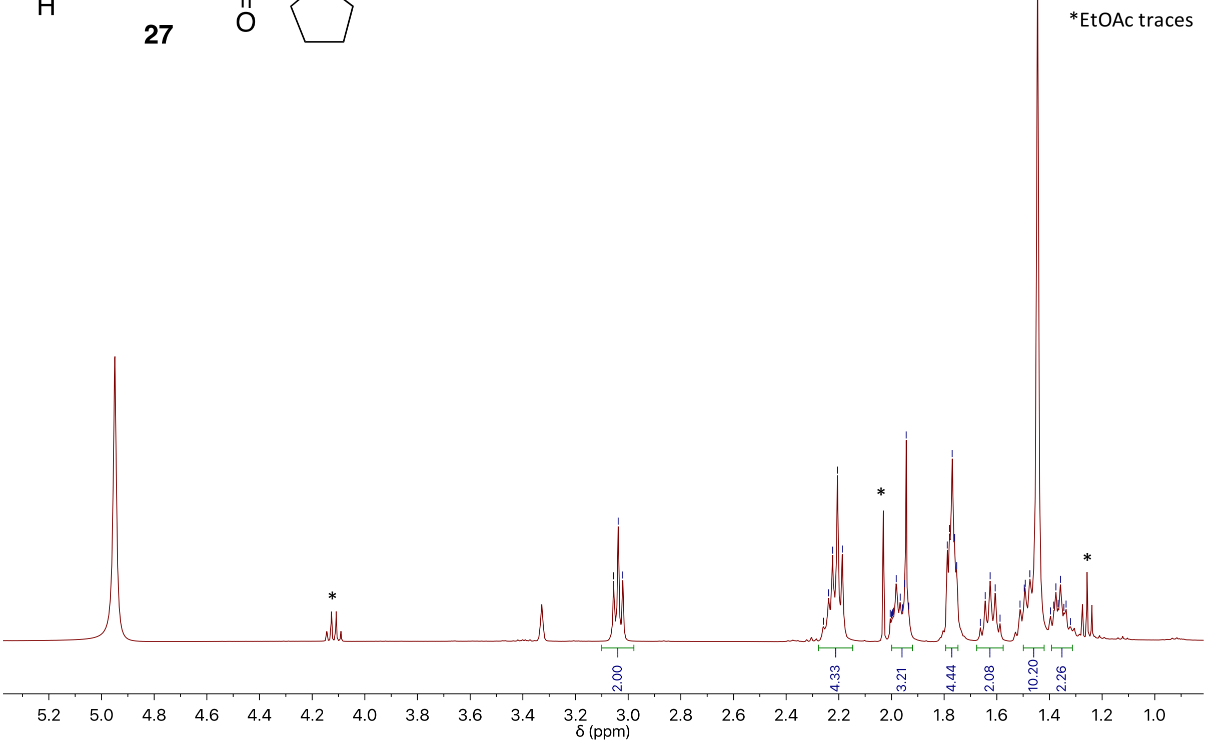
3.06
3.04
3.02
2.26
2.24
2.22
2.21
2.19
2.00
2.00
1.99
1.98
1.97
1.96
1.95
1.94
1.93
1.79
1.78
1.77
1.76
1.75
1.64
1.63
1.61
1.51
1.50
1.49
1.47
1.45
1.38
1.37
1.36
*EtOAc traces
2.00
4.33
3.21
4.44
2.08
10.20
2.26
δ (ppm)

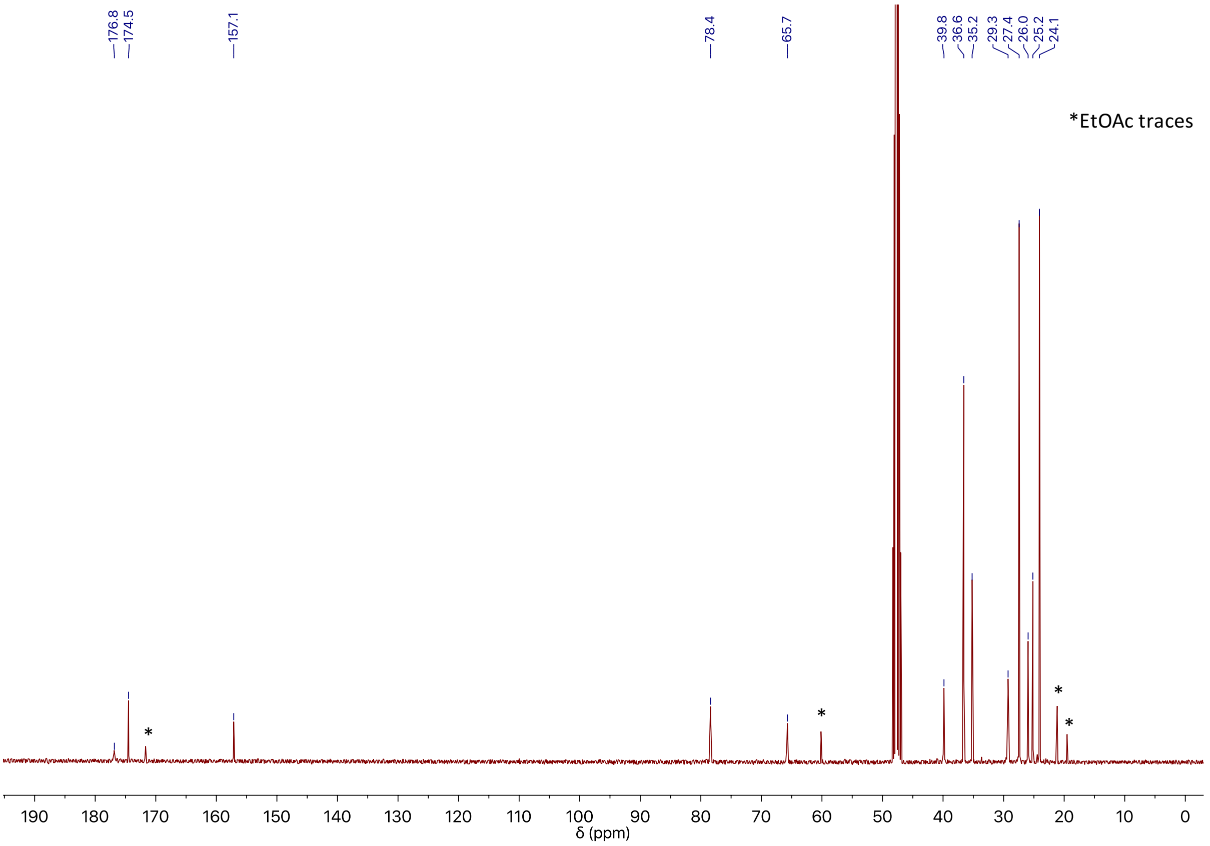
176.8
174.5
157.1
78.4
65.7
39.8
36.6
35.2
29.3
27.4
26.0
25.2
24.1
*EtOAc traces
δ (ppm)

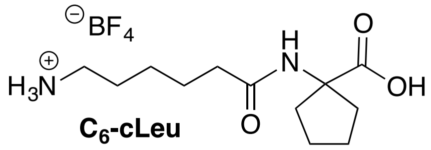
BF4
H3N
H
N
O
OH
O
C6-cLeu

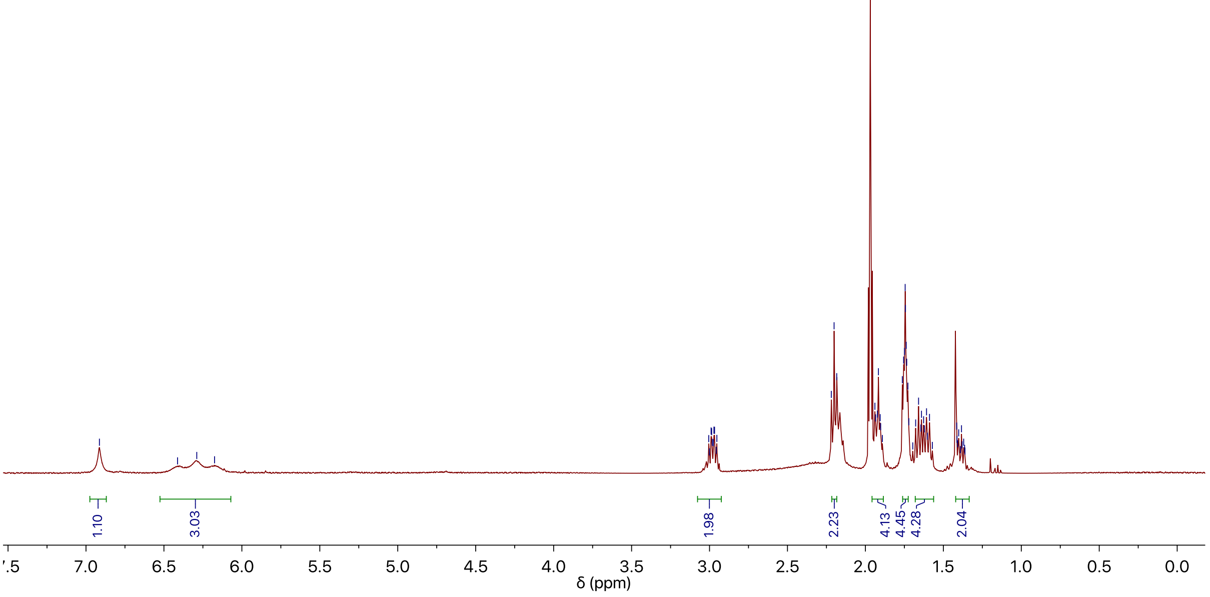
6.91
6.41
6.29
6.18
3.00
3.00
2.99
2.99
2.98
2.98
2.97
2.97
2.96
2.95
2.22
2.20
2.18
1.94
1.93
1.92
1.92
1.91
1.90
1.76
1.76
1.75
1.75
1.74
1.74
1.73
1.72
1.68
1.66
1.64
1.63
1.61
1.59
1.10
3.03
1.98
2.23
4.13
4.45
4.28
2.04
7.0
6.5
6.0
5.5
5.0
4.5
4.0
3.5
3.0
2.5
2.0
1.5
1.0
0.5
0.0
δ (ppm)

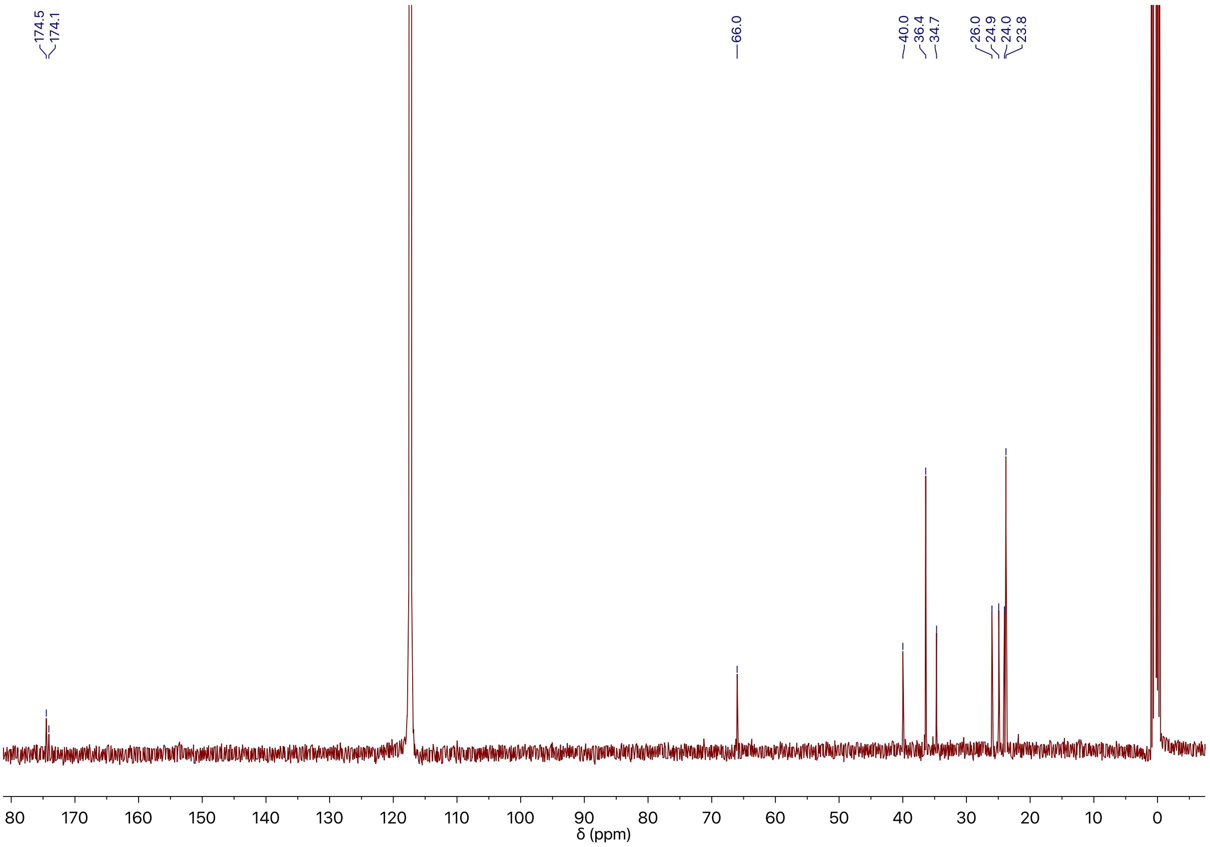
174.5
174.1
66.0
40.0
36.4
34.7
26.0
24.9
24.0
23.8
170
160
150
140
130
120
110
100
90
80
70
60
50
40
30
20
10
0
δ (ppm)

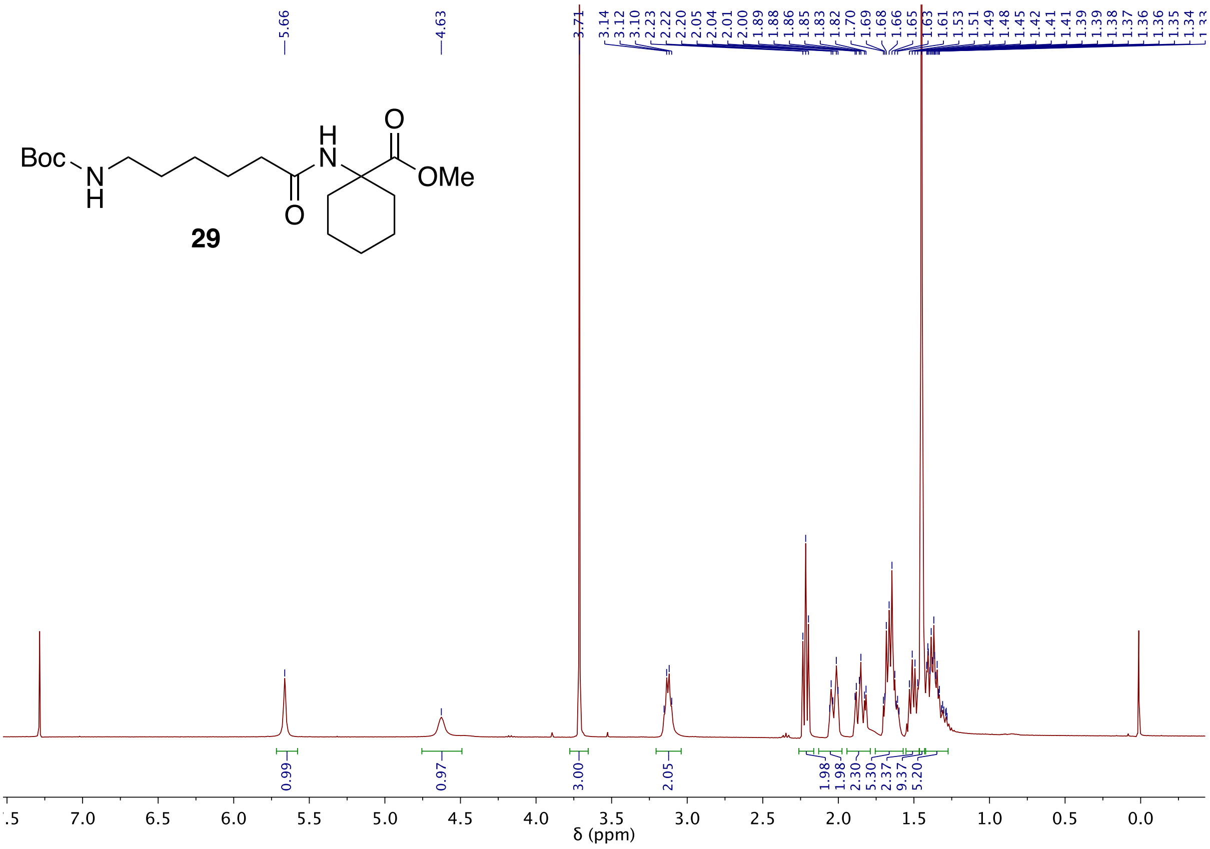

Boc
N
H
H
N
O
O
OMe
29
5.66
4.63
3.71
3.14
3.12
3.10
2.23
2.22
2.20
2.05
2.04
2.01
2.00
1.89
1.88
1.86
1.85
1.83
1.82
1.70
1.69
1.68
1.66
1.65
1.63
1.61
1.53
1.51
1.49
1.48
1.45
1.42
1.41
1.39
1.38
1.37
1.36
1.35
1.34
0.99
0.97
3.00
2.05
1.98
1.98
2.30
5.30
2.37
9.37
5.20
δ (ppm)

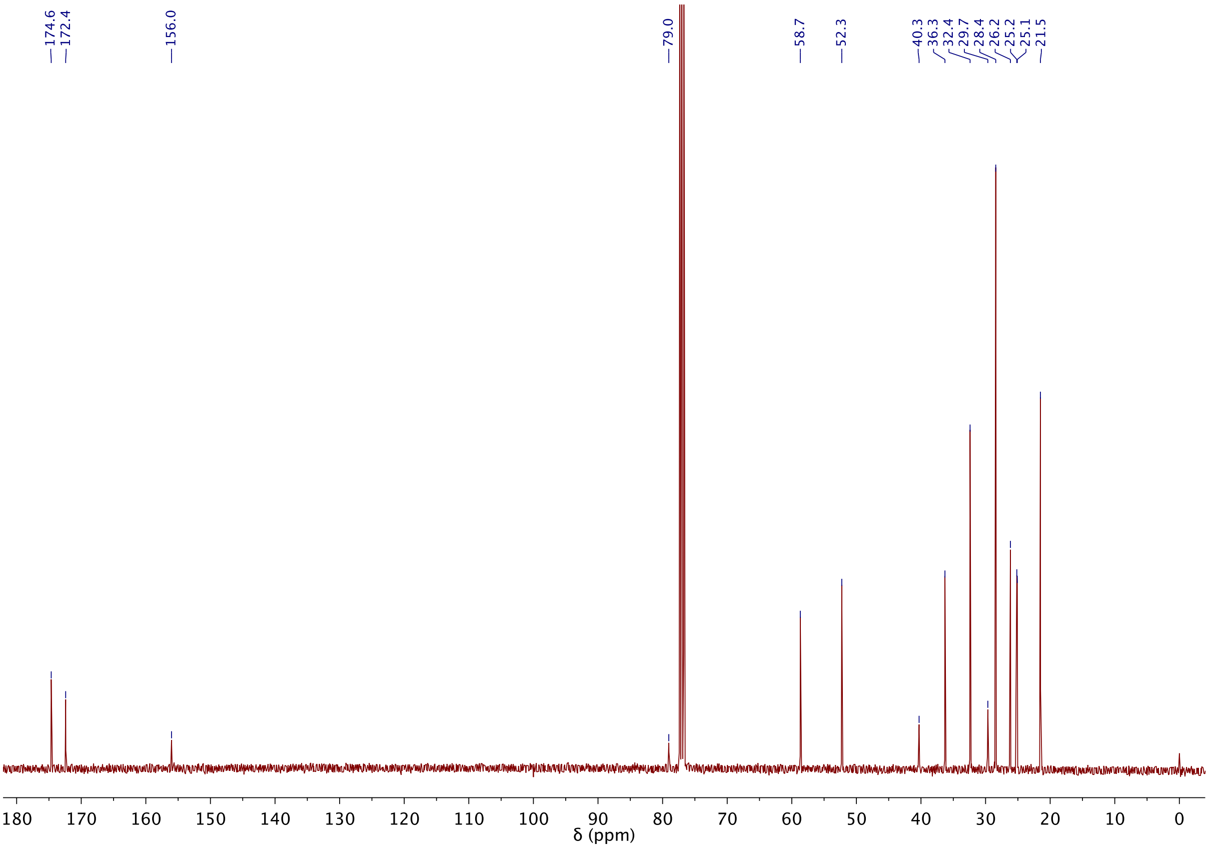

174.6
172.4
156.0
79.0
58.7
52.3
40.3
36.3
32.4
29.7
28.4
26.2
25.2
25.1
21.5
δ (ppm)

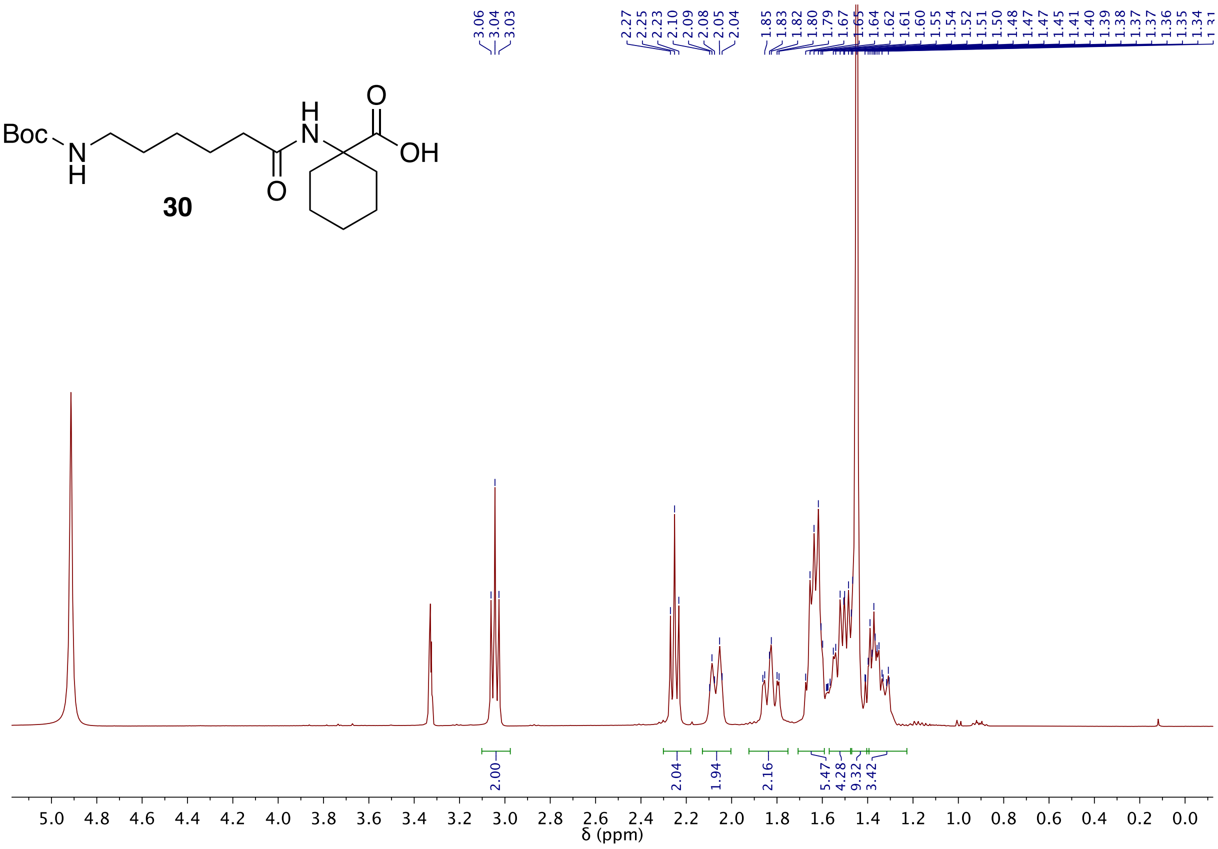

30

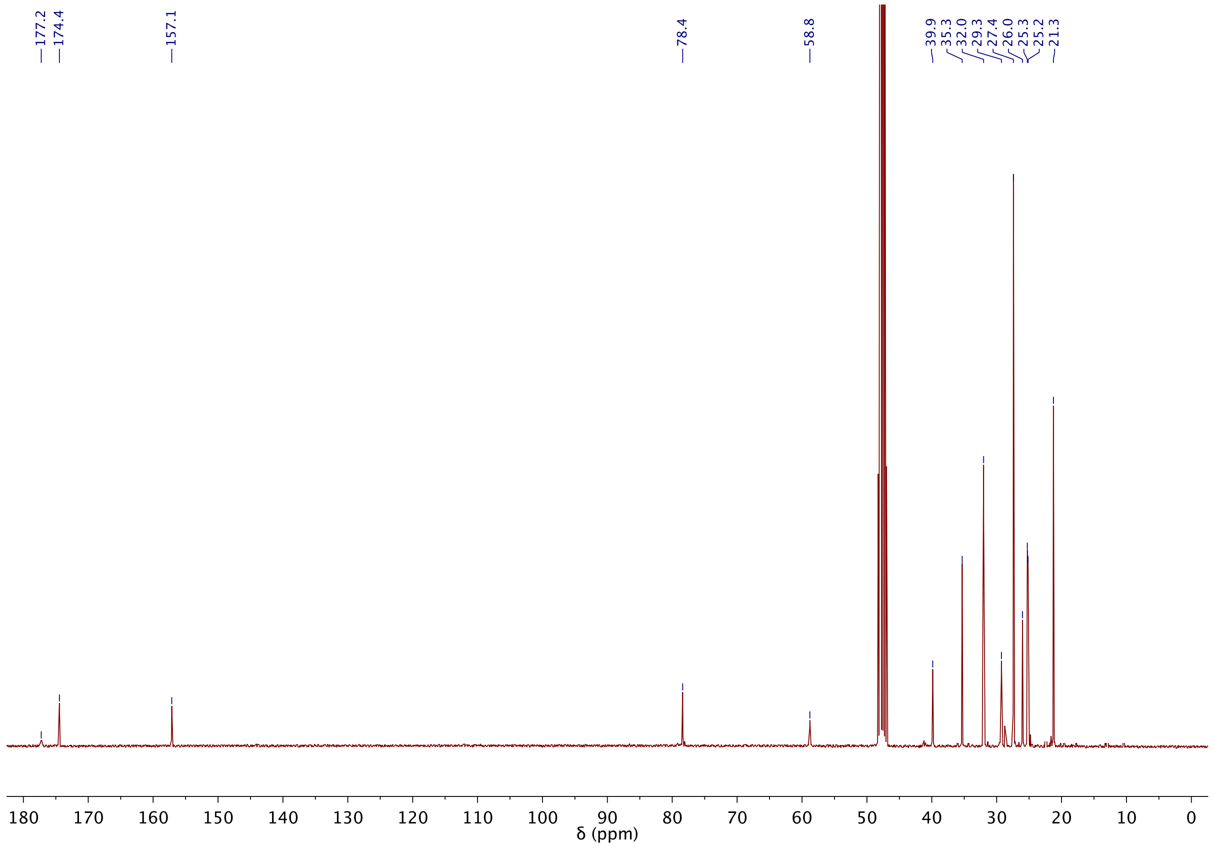

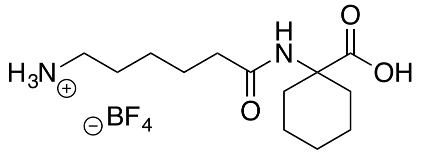
H3N
BF4
O
H
N
O
OH

C_6-HcLeu

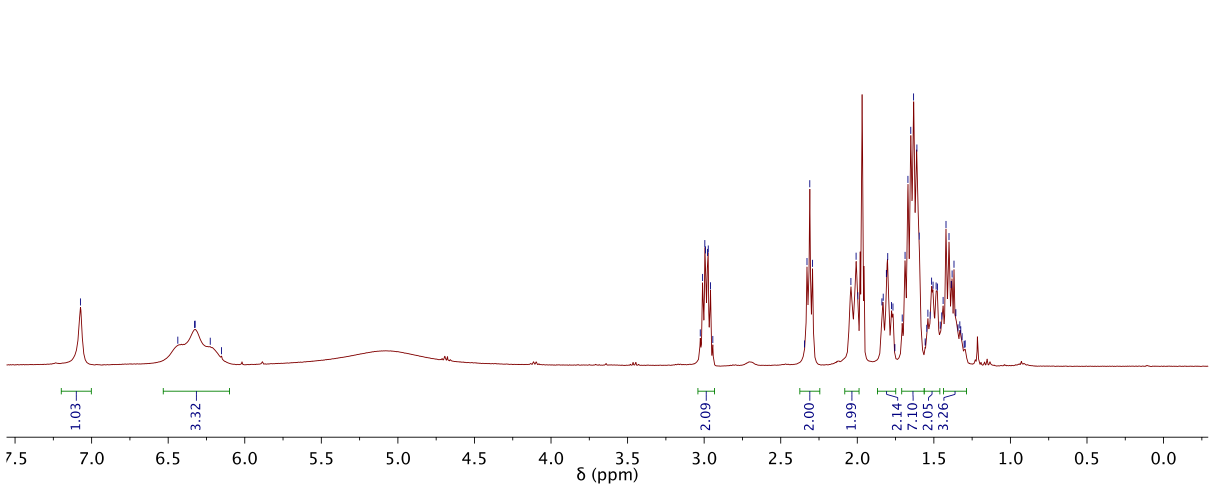
7.07
6.44
6.33
6.32
6.23
6.15
3.03
3.01
3.00
2.99
2.98
2.97
2.96
2.94
2.34
2.33
2.31
2.29
2.04
2.01
2.00
1.84
1.83
1.81
1.80
1.78
1.77
1.75
1.71
1.69
1.67
1.65
1.63
1.61
1.60
1.56
1.55
1.54
1.52
1.51
1.51
1.49
1.48
1.46
1.45
1.44
1.42
1.40
1.39
1.38
1.37
1.36
1.34
1.33
1.32
1.32
1.30
1.30
1.03
3.32
2.09
2.00
1.99
2.14
7.10
2.05
3.26
δ (ppm)

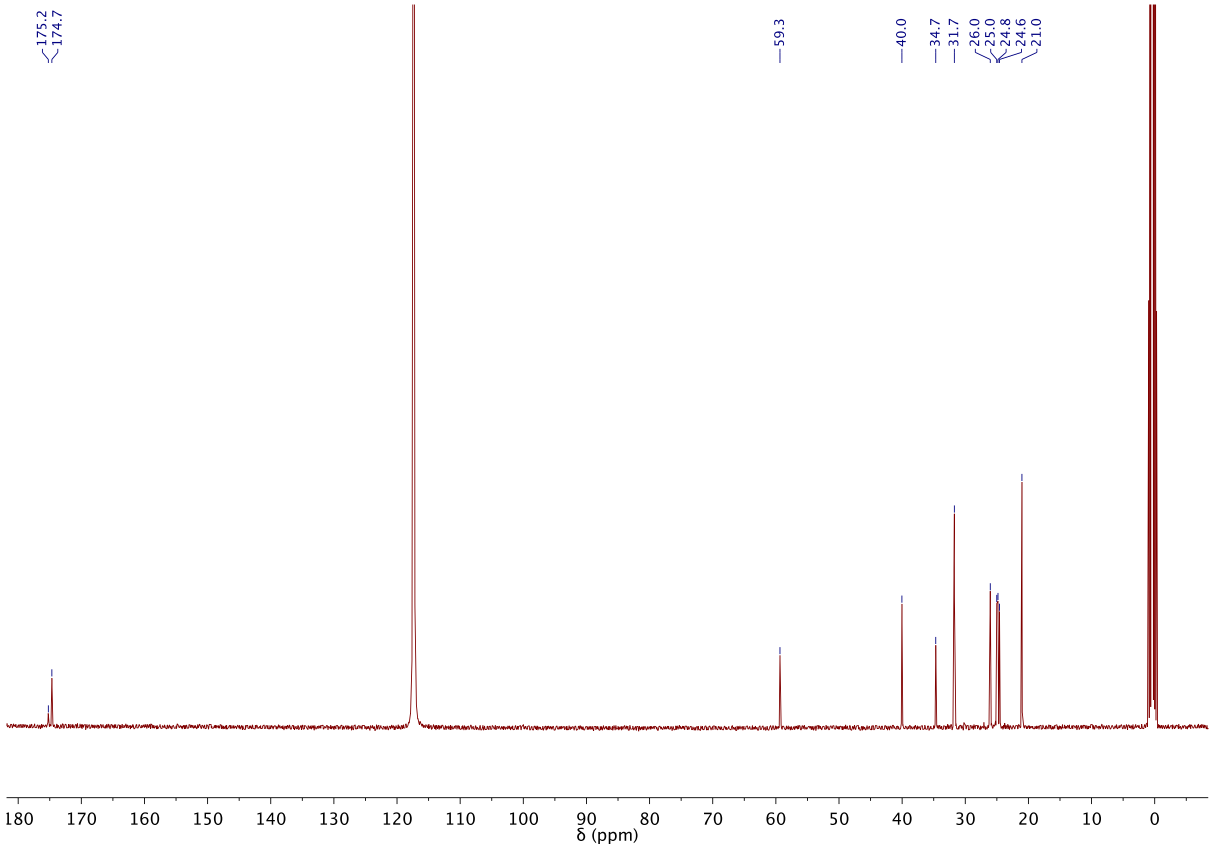
175.2
174.7
59.3
40.0
34.7
31.7
26.0
25.0
24.8
24.6
21.0
δ (ppm)

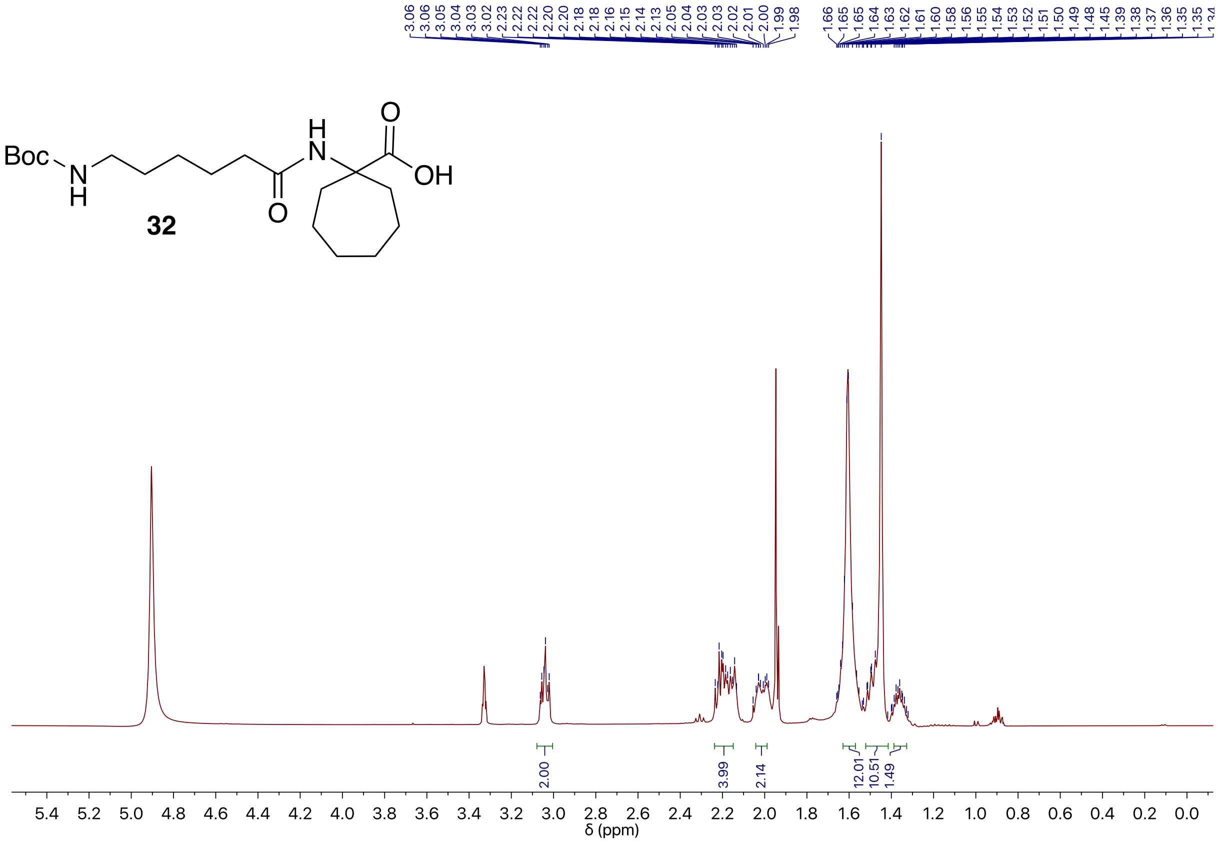

3.06 3.06 3.05 3.04 3.03 3.02 2.23 2.22 2.22 2.20 2.20 2.18 2.16 2.15 2.14 2.13 2.05 2.04 2.03 2.03 2.02 2.01 2.00 1.99 1.98
1.66 1.65 1.65 1.64 1.63 1.62 1.61 1.60 1.58 1.56 1.55 1.54 1.53 1.52 1.51 1.50 1.49 1.48 1.45 1.39 1.38 1.37 1.36 1.35 1.35
Boc
N
H
H
N
O
OH
O
32
2.00
3.99
2.14
12.01
10.51
1.49
5.4 5.2 5.0 4.8 4.6 4.4 4.2 4.0 3.8 3.6 3.4 3.2 3.0 2.8 2.6 2.4 2.2 2.0 1.8 1.6 1.4 1.2 1.0 0.8 0.6 0.4 0.2 0.0
δ (ppm)

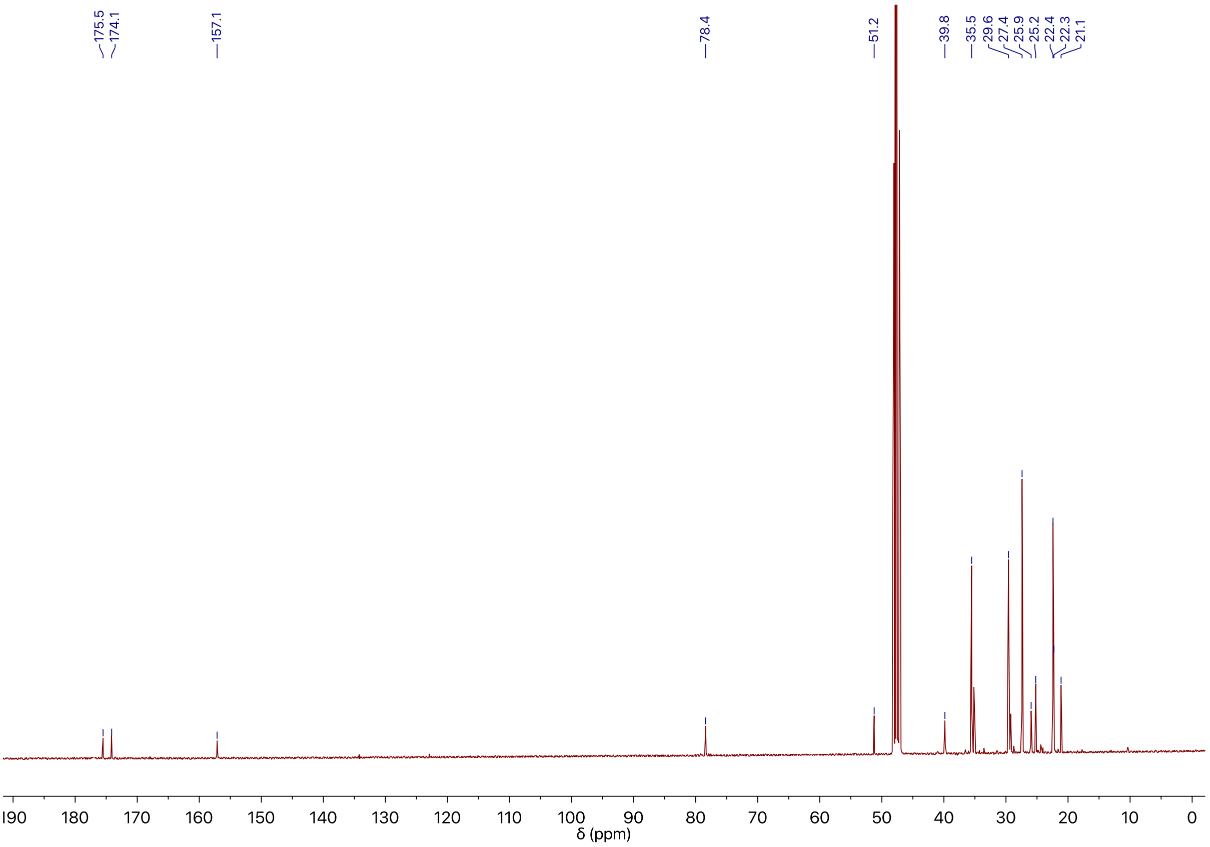

175.5
174.1
157.1
78.4
51.2
39.8
35.5
29.6
27.4
25.9
25.2
22.4
22.3
21.1
190 180 170 160 150 140 130 120 110 100 90 80 70 60 50 40 30 20 10 0
δ (ppm)

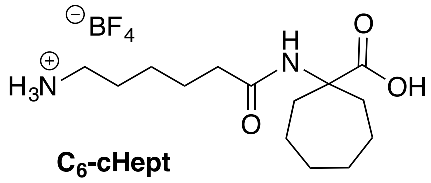
BF4
H3N
H
N
O
OH

C$_6$-cHept

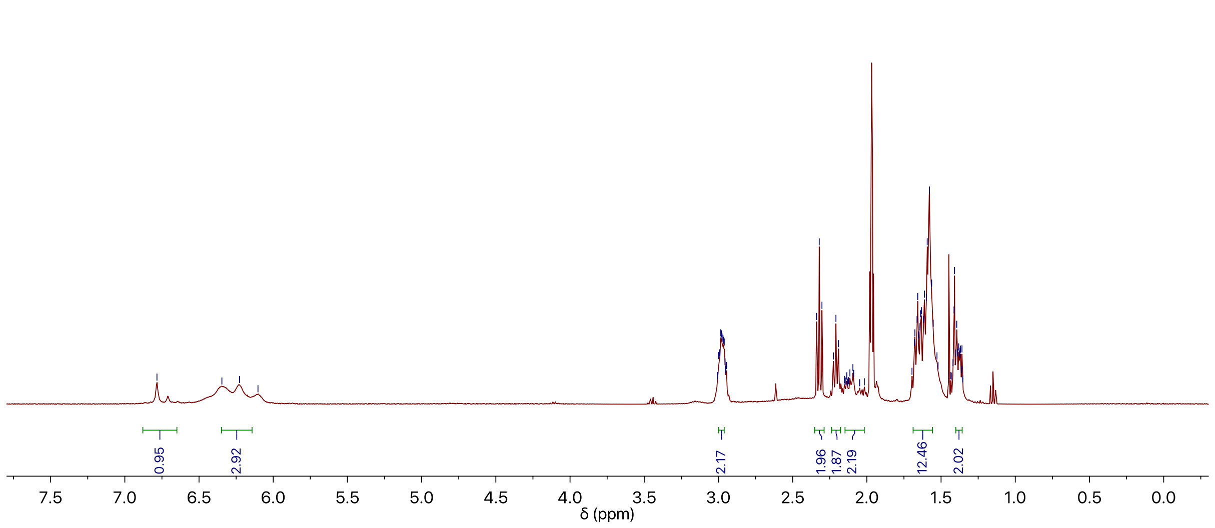
6.78
6.35
6.23
6.10
0.95
2.92
2.17
1.96
1.87
2.19
12.46
2.02
δ (ppm)

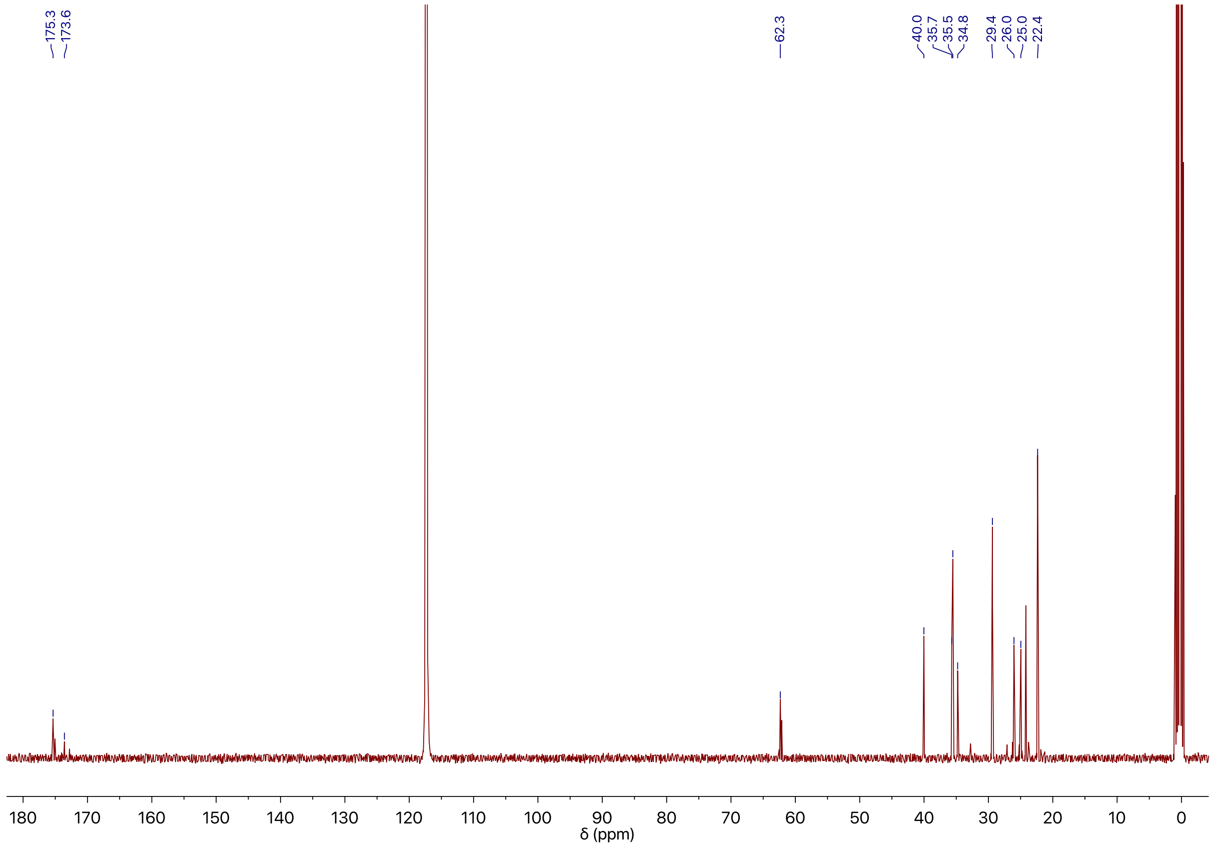
175.3
173.6
62.3
40.0
35.7
35.5
34.8
29.4
26.0
25.0
22.4
δ (ppm)

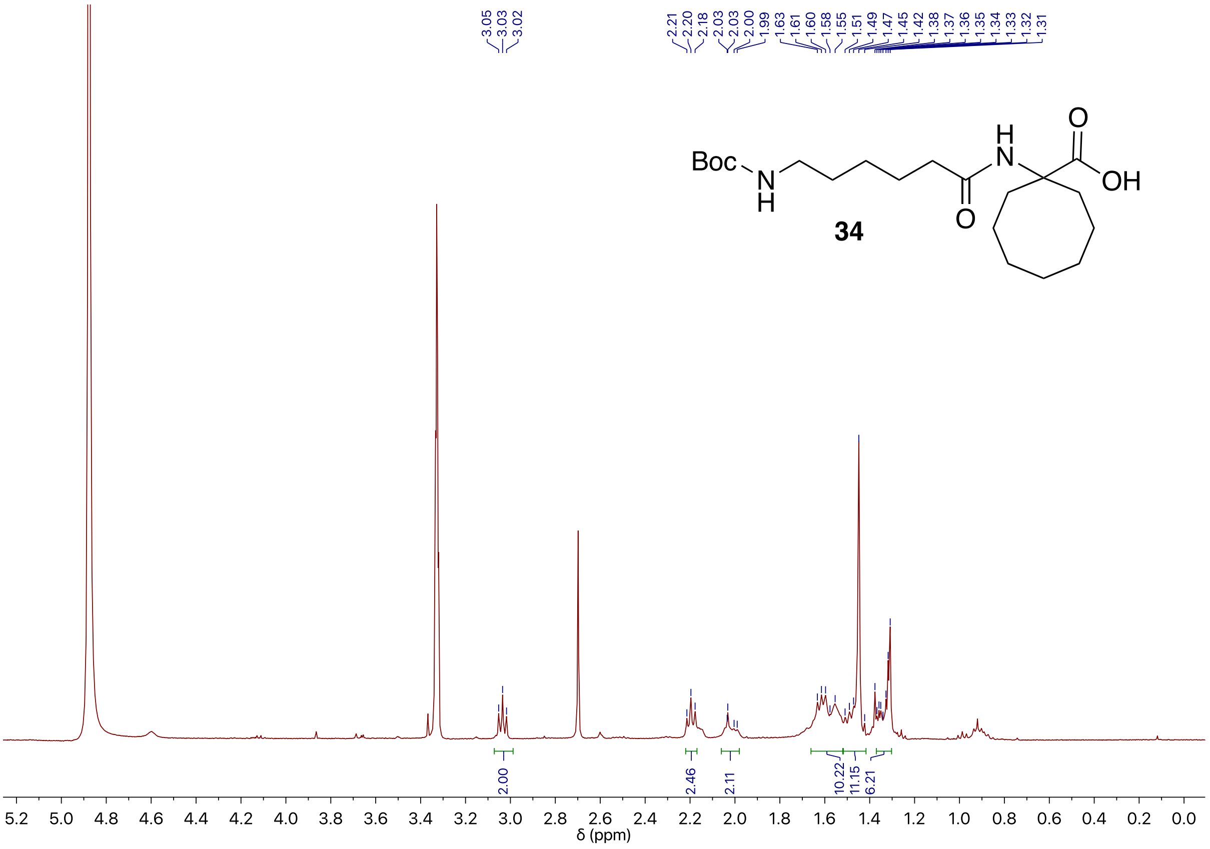
3.05
3.03
3.02
2.21
2.20
2.18
2.03
2.03
2.00
1.99
1.63
1.61
1.60
1.58
1.55
1.51
1.49
1.47
1.45
1.42
1.38
1.37
1.36
1.35
1.34
1.33
1.32
1.31
Boc
N
H
H
N
O
OH
O
34
2.00
2.46
2.11
10.22
11.15
6.21
5.2 5.0 4.8 4.6 4.4 4.2 4.0 3.8 3.6 3.4 3.2 3.0 2.8 2.6 2.4 2.2 2.0 1.8 1.6 1.4 1.2 1.0 0.8 0.6 0.4 0.2 0.0
δ (ppm)

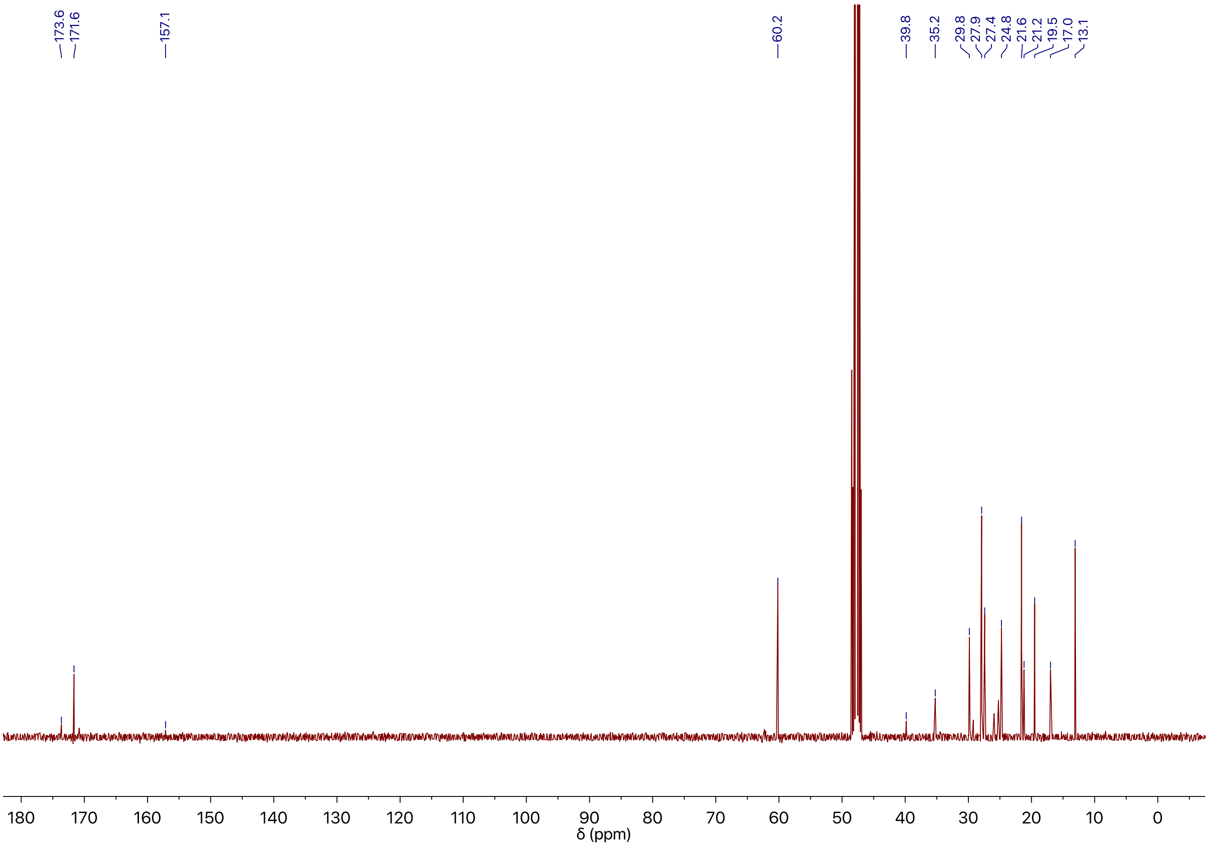
173.6
171.6
157.1
60.2
39.8
35.2
29.8
27.9
27.4
24.8
21.6
21.2
19.5
17.0
13.1
180 170 160 150 140 130 120 110 100 90 80 70 60 50 40 30 20 10 0
δ (ppm)

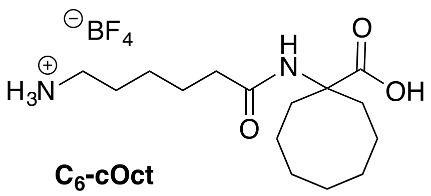

C$_6$-cOct

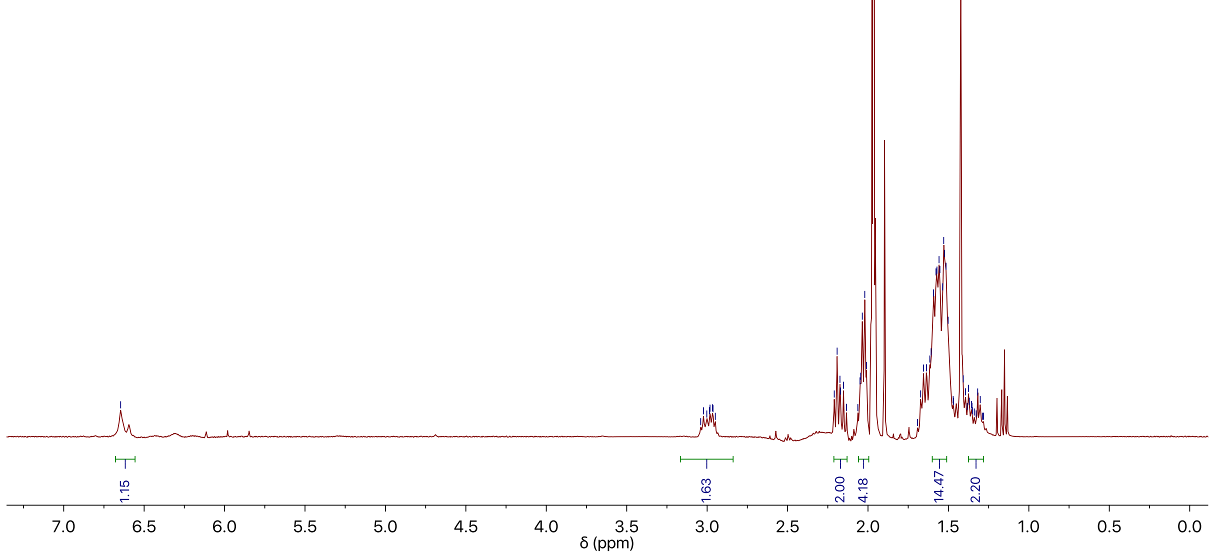

6.65
3.04
3.02
3.00
2.99
2.98
2.97
2.96
2.95
2.21
2.19
2.17
2.15
2.13
2.06
2.05
2.05
2.04
2.02
2.01
1.67
1.65
1.64
1.62
1.61
1.59
1.58
1.57
1.56
1.55
1.54
1.53
1.52
1.51
1.50
1.47
1.41
1.39
1.38
1.15
1.63
2.00
4.18
14.47
2.20
δ (ppm)

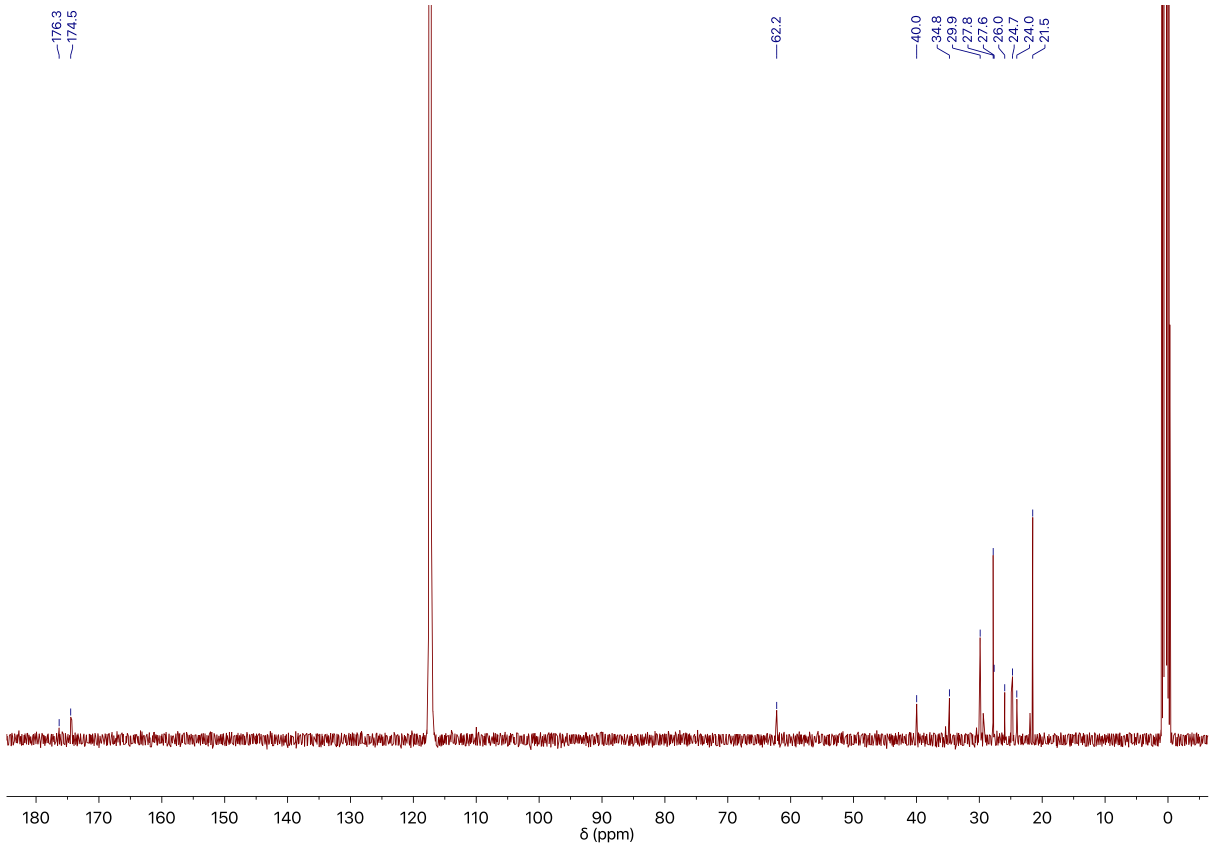

176.3
174.5
62.2
40.0
34.8
29.9
27.8
27.6
26.0
24.7
24.0
21.5
δ (ppm)

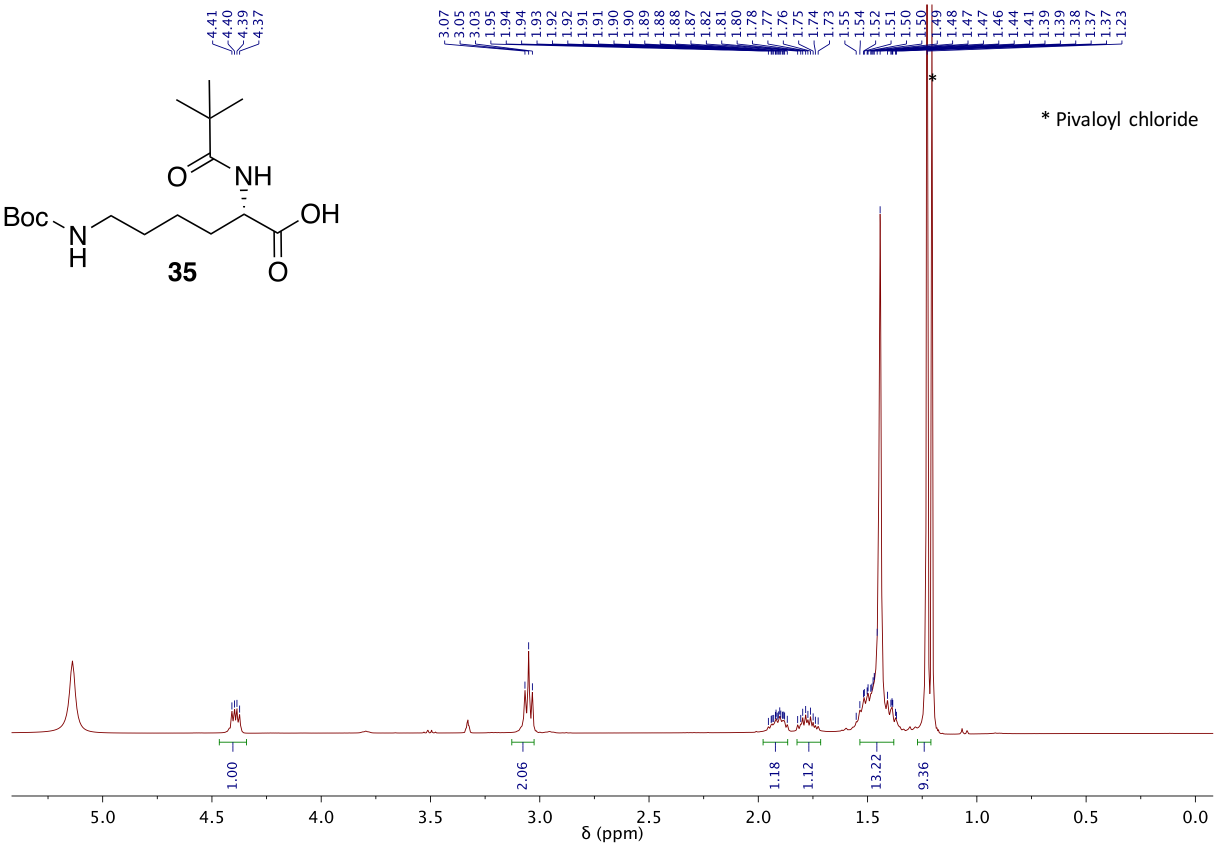

35
4.41
4.40
4.39
4.37
3.07
3.05
3.03
1.95
1.94
1.94
1.93
1.92
1.92
1.91
1.91
1.90
1.90
1.89
1.88
1.88
1.87
1.82
1.81
1.80
1.78
1.77
1.76
1.75
1.74
1.73
1.55
1.54
1.52
1.51
1.50
1.50
1.49
1.48
1.47
1.47
1.46
1.44
1.41
1.39
1.39
1.38
1.37
1.37
1.23
* Pivaloyl chloride
1.00
2.06
1.18
1.12
13.22
9.36
5.0
4.5
4.0
3.5
3.0
2.5
2.0
1.5
1.0
0.5
0.0
δ (ppm)

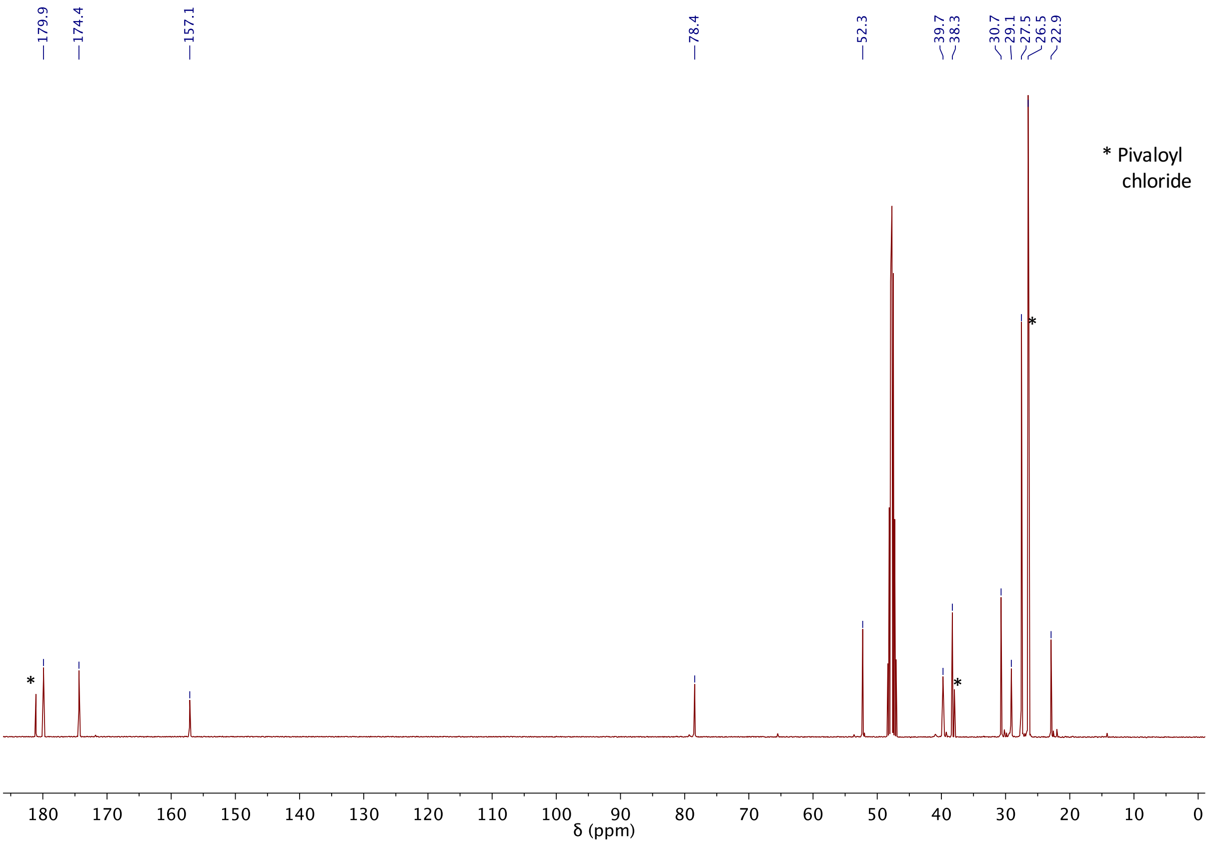

179.9
174.4
157.1
78.4
52.3
39.7
38.3
30.7
29.1
27.5
26.5
22.9
* Pivaloyl chloride
180
170
160
150
140
130
120
110
100
90
80
70
60
50
40
30
20
10
0
δ (ppm)

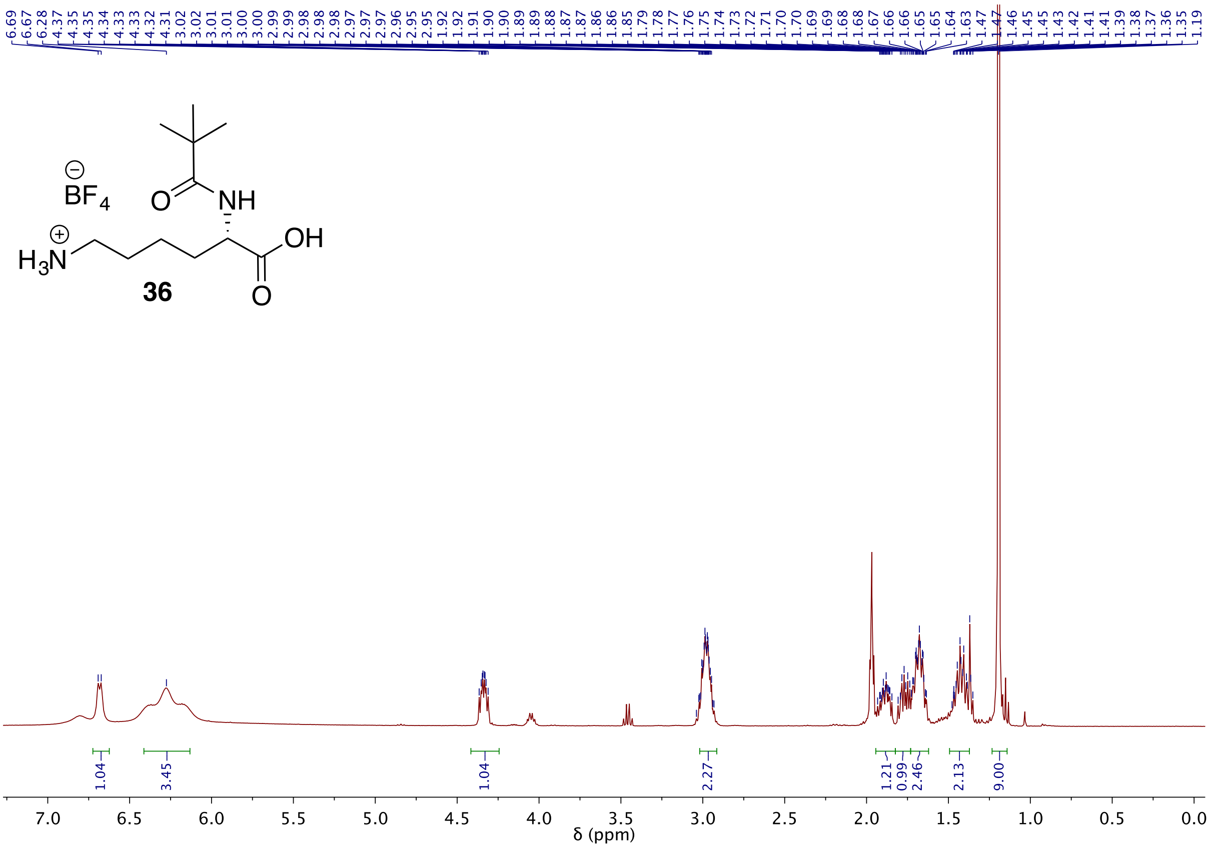
6.69 6.67 6.28 4.37 4.35 4.34 4.33 4.33 4.32 4.31 3.02 3.02 3.01 3.01 3.00 3.00 2.99 2.99 2.98 2.98 2.98 2.97 2.97 2.96 2.95 2.95 1.92 1.92 1.91 1.90 1.90 1.89 1.89 1.88 1.87 1.87 1.86 1.86 1.85 1.79 1.78 1.77 1.76 1.75 1.74 1.73 1.72 1.71 1.70 1.70 1.69 1.69 1.68 1.67 1.66 1.66 1.65 1.65 1.64 1.63 1.47 1.47 1.46 1.45 1.45 1.43 1.42 1.41 1.41 1.39 1.38 1.37 1.36 1.35 1.19
BF4
O
NH
OH
H3N
36
O
1.04
3.45
1.04
2.27
1.21
0.99
2.46
2.13
9.00
δ (ppm)

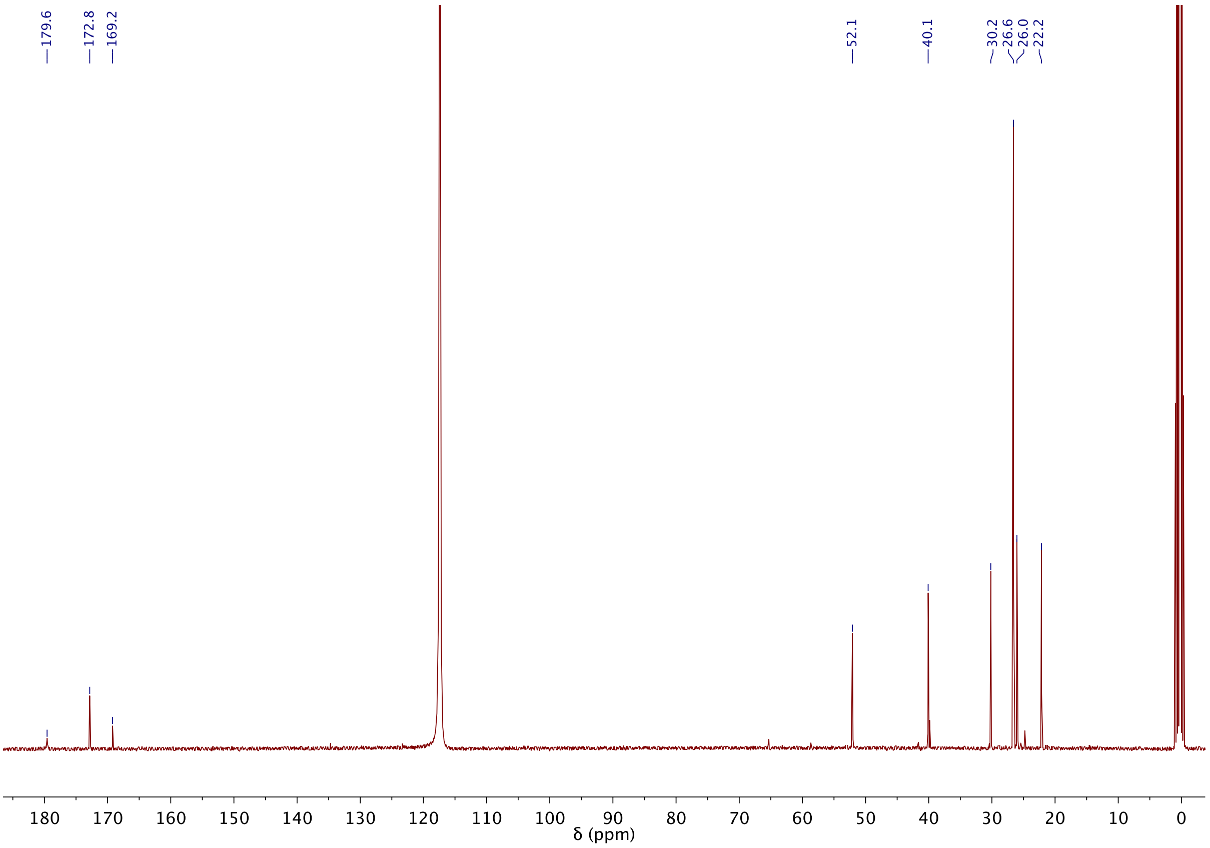
179.6
172.8
169.2
52.1
40.1
30.2
26.6
26.0
22.2
δ (ppm)

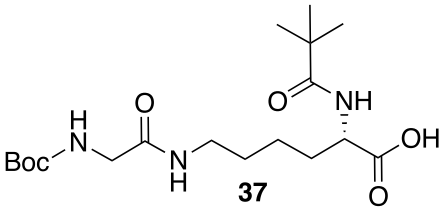
Boc
N
H
O
N
H
O
NH
OH
O

37

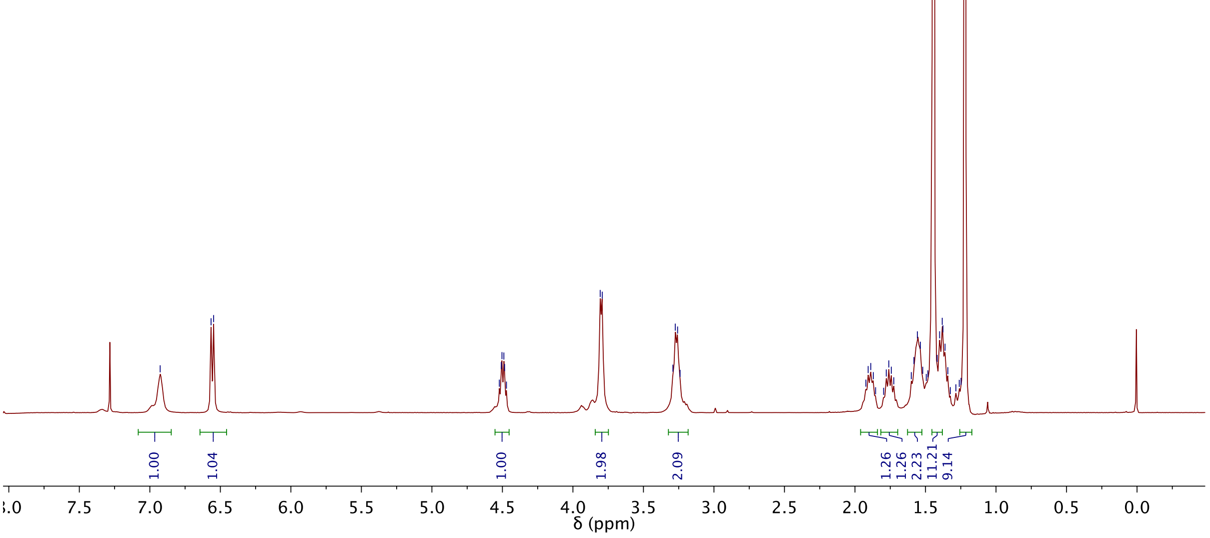
6.93
6.57
6.55
4.52
4.51
4.50
4.49
4.48
4.47
3.81
3.79
3.29
3.27
3.26
3.24
1.92
1.91
1.89
1.87
1.85
1.80
1.78
1.76
1.74
1.72
1.60
1.58
1.56
1.54
1.52
1.49
1.48
1.44
1.42
1.40
1.38
1.36
1.34
1.32
1.28
1.26
1.25
1.22
1.00
1.04
1.00
1.98
2.09
1.26
1.26
2.23
11.21
9.14
δ (ppm)

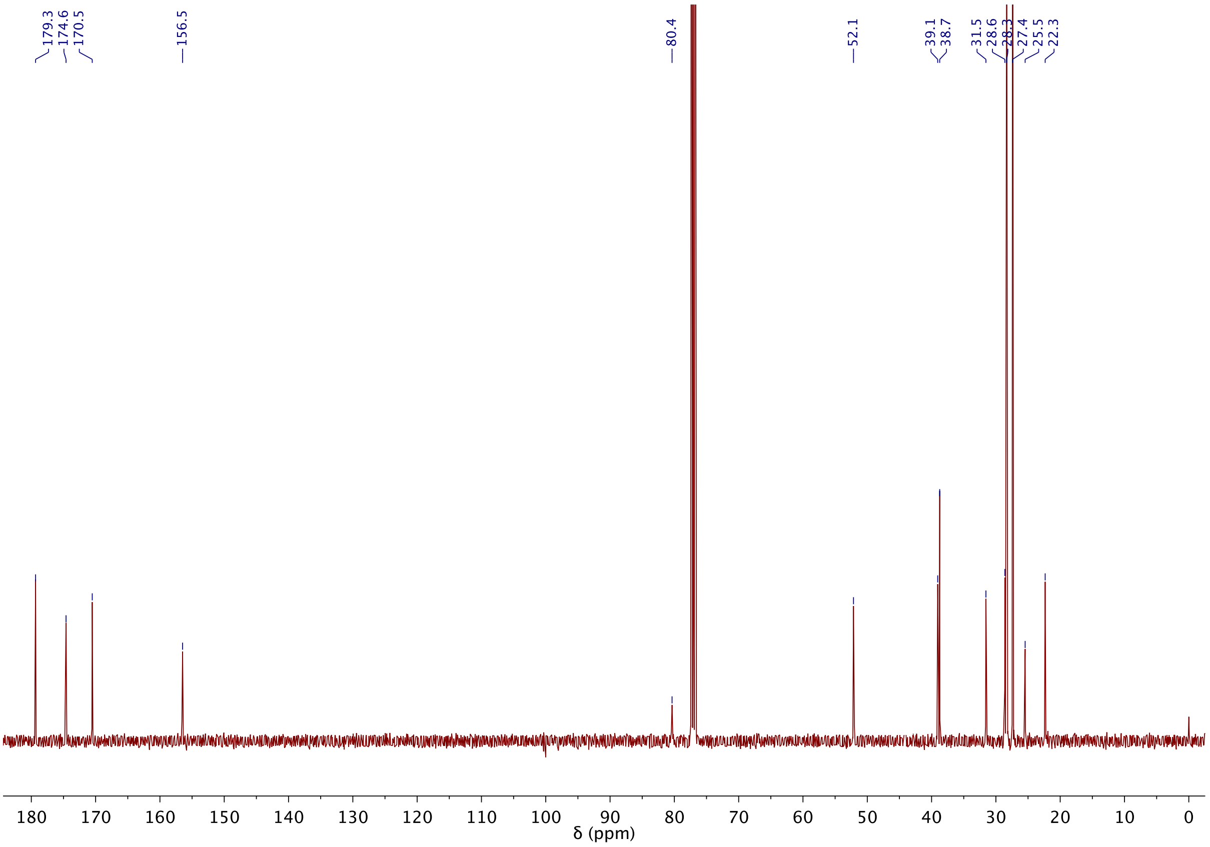
179.3
174.6
170.5
156.5
80.4
52.1
39.1
38.7
31.5
28.6
28.3
27.4
25.5
22.3
δ (ppm)

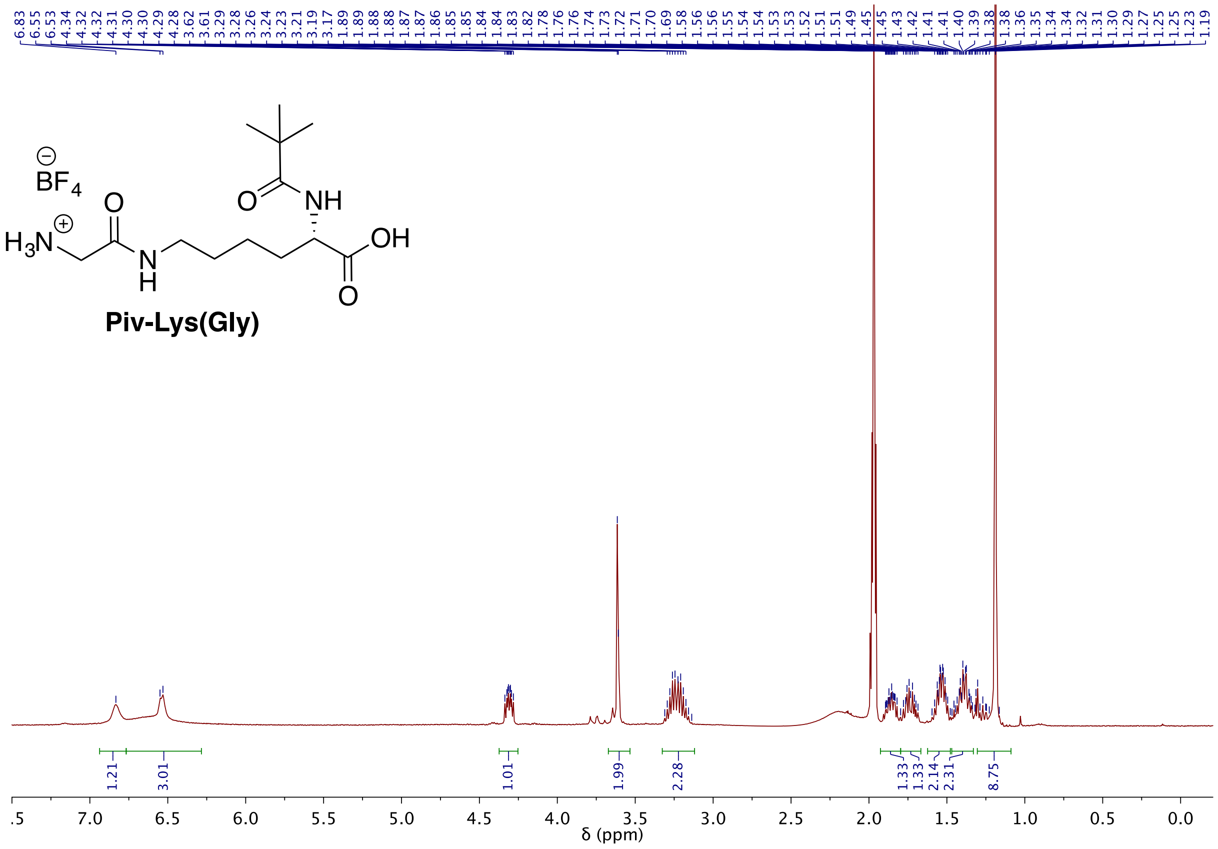
BF4
H3N
O
NH
OH
N
H
O
O
Piv-Lys(Gly)
1.21
3.01
1.01
1.99
2.28
1.33
1.33
2.14
2.31
8.75
δ (ppm)

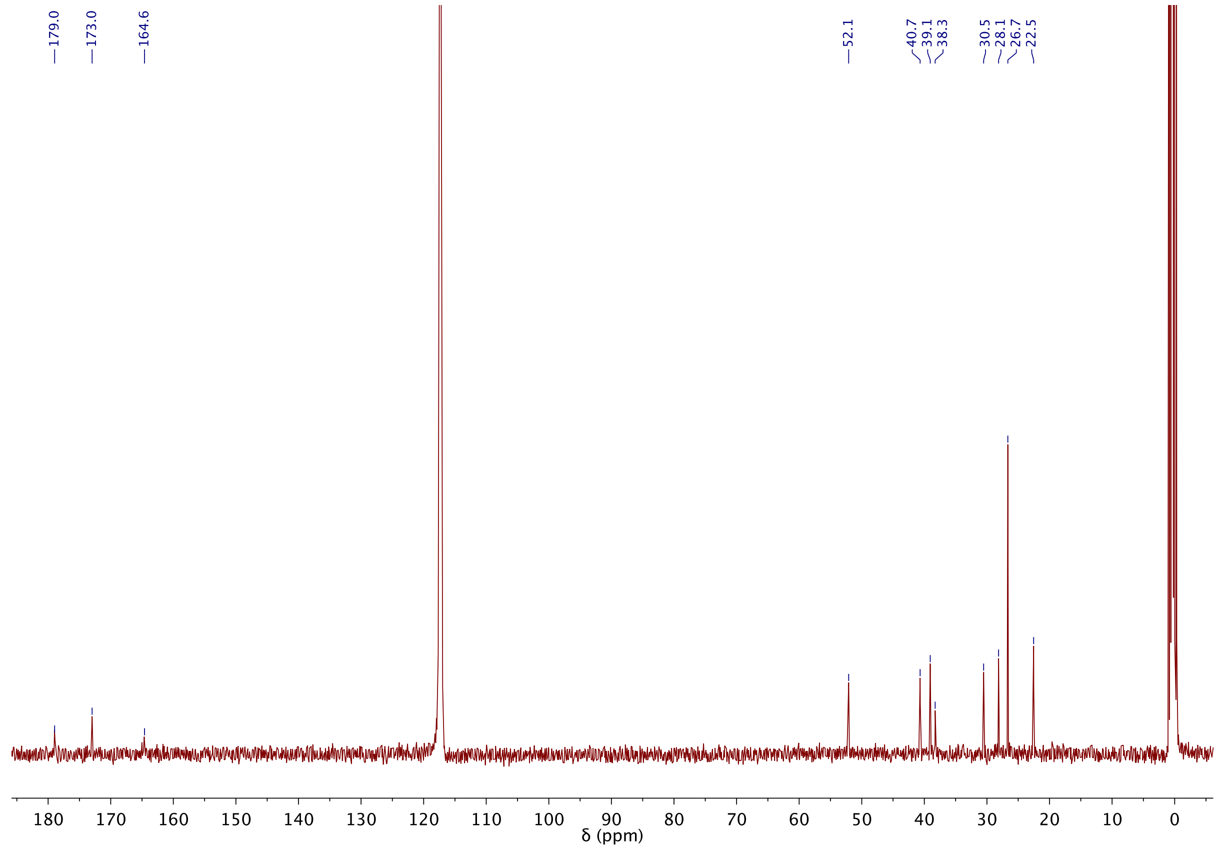
179.0
173.0
164.6
52.1
40.7
39.1
38.3
30.5
28.1
26.7
22.5
δ (ppm)

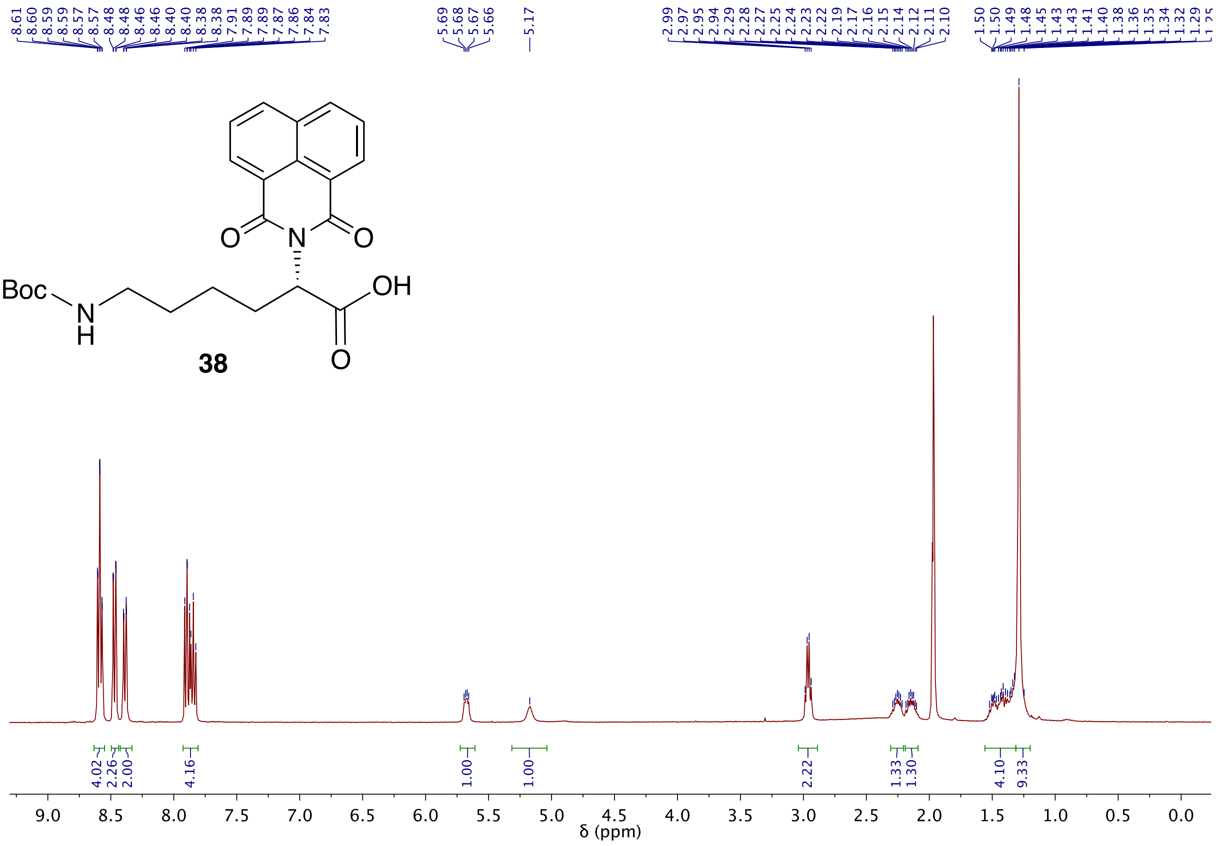
8.61 8.60 8.59 8.57 8.57 8.48 8.48 8.46 8.46 8.40 8.40 8.38 8.38 7.91 7.89 7.89 7.87 7.86 7.84 7.83
5.69 5.68 5.67 5.66
5.17
2.99 2.97 2.95 2.94 2.29 2.28 2.27 2.25 2.24 2.23 2.22 2.19 2.17 2.16 2.15 2.14 2.12 2.11 2.10
1.50 1.50 1.49 1.48 1.45 1.43 1.43 1.41 1.40 1.38 1.36 1.35 1.34 1.32 1.29
Boc
N
H
O
OH
38
4.02 2.26 2.00 4.16 1.00 1.00 2.22 1.33 1.30 4.10 9.33
δ (ppm)

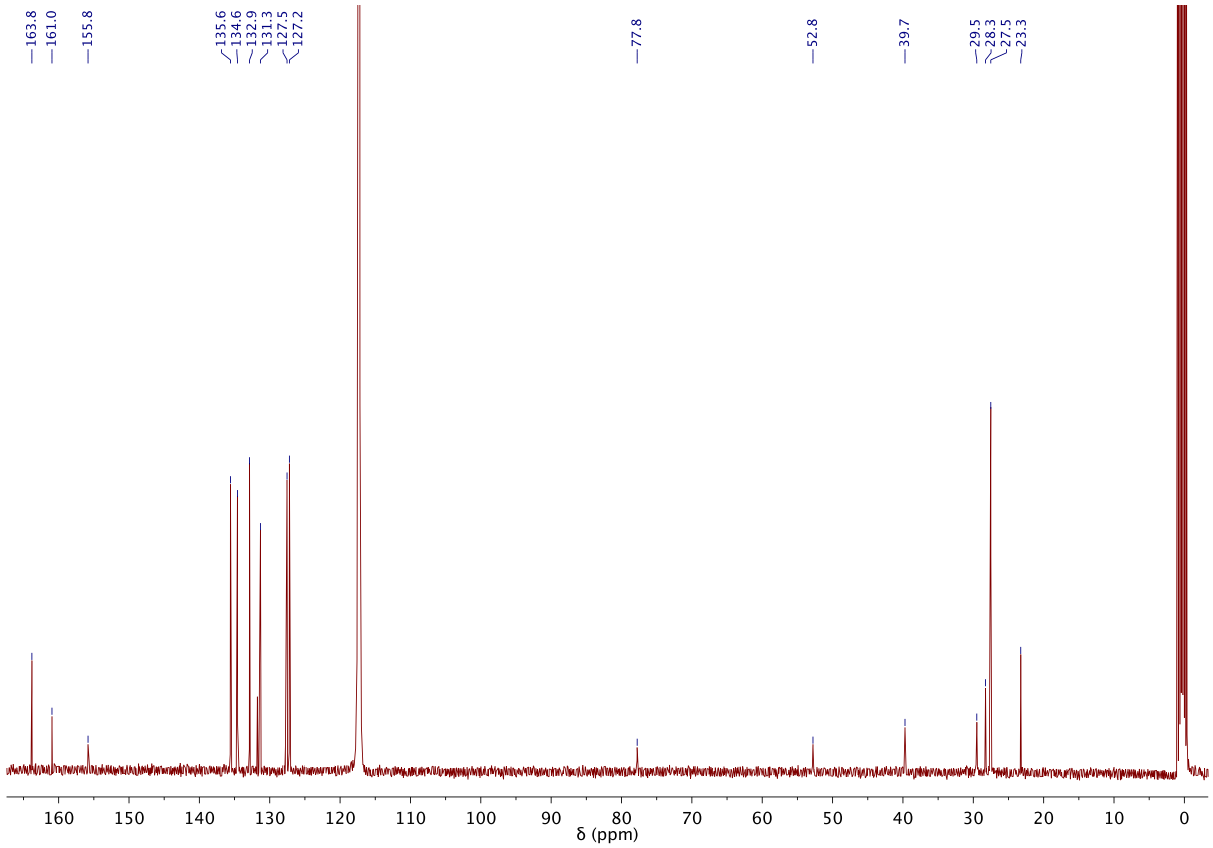
163.8
161.0
155.8
135.6 134.6 132.9 131.3 127.5 127.2
77.8
52.8
39.7
29.5 28.3 27.5 23.3
δ (ppm)

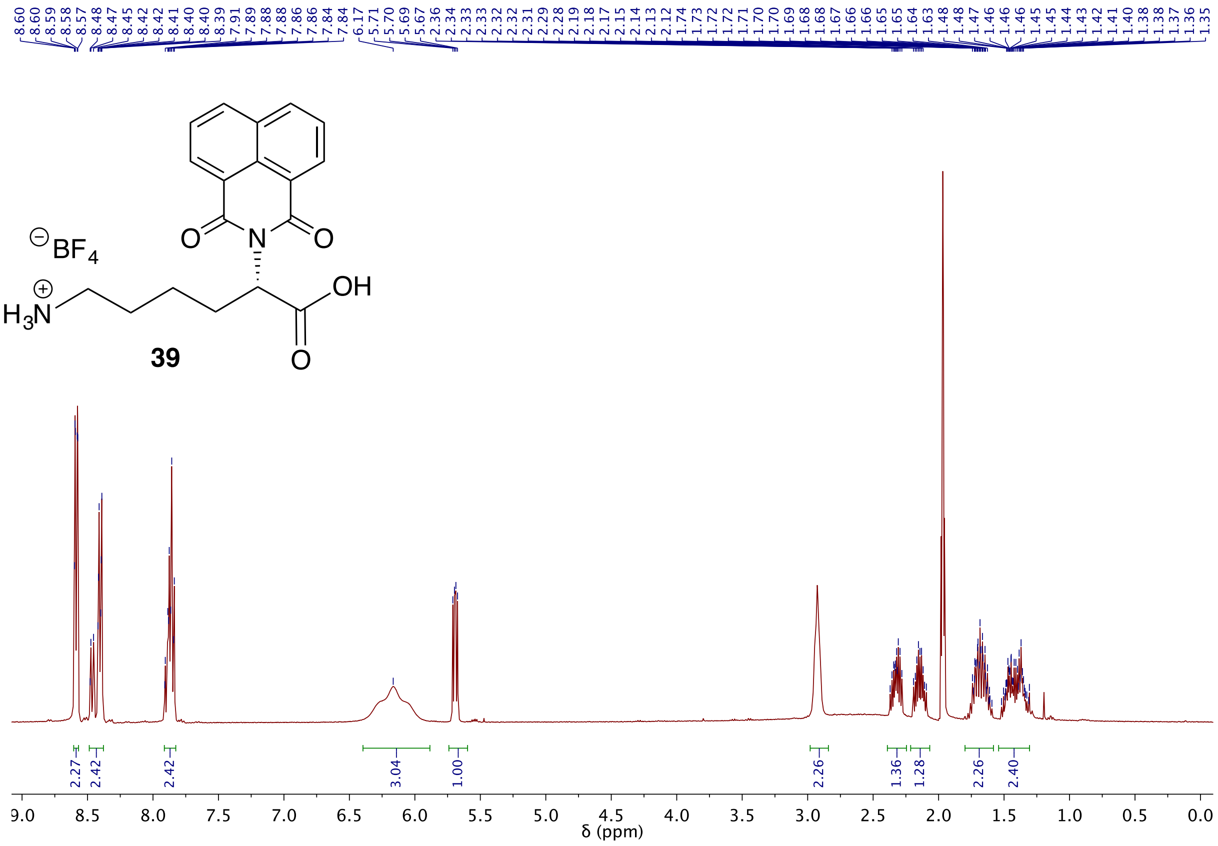

8.60 8.60 8.59 8.58 8.57 8.48 8.47 8.45 8.42 8.42 8.41 8.40 8.40 8.39 7.91 7.89 7.88 7.86 7.86 7.84 7.84 6.17 5.71 5.70 5.69 5.67 2.36 2.34 2.33 2.33 2.32 2.32 2.31 2.29 2.28 2.19 2.18 2.17 2.15 2.14 2.13 2.12 1.74 1.73 1.72 1.72 1.71 1.70 1.69 1.68 1.68 1.67 1.66 1.66 1.65 1.65 1.64 1.63 1.48 1.48 1.47 1.46 1.46 1.45 1.45 1.44 1.43 1.42 1.41 1.40 1.38 1.38 1.37 1.36 1.35
BF4
H3N
O
N
O
OH
O
39
2.27
2.42
2.42
3.04
1.00
2.26
1.36
1.28
2.26
2.40
9.0 8.5 8.0 7.5 7.0 6.5 6.0 5.5 5.0 4.5 4.0 3.5 3.0 2.5 2.0 1.5 1.0 0.5 0.0
δ (ppm)

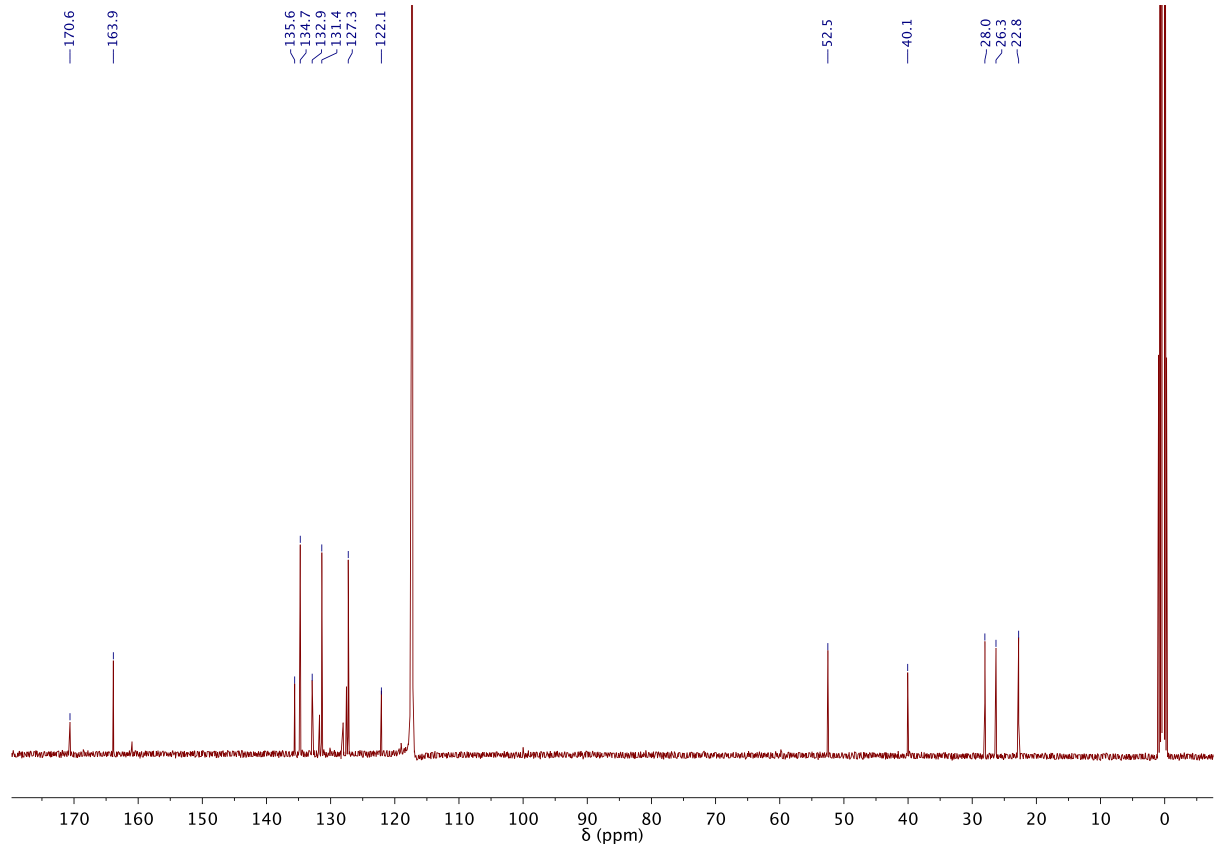

170.6
163.9
135.6 134.7 132.9 131.4 127.3
122.1
52.5
40.1
28.0 26.3 22.8
170 160 150 140 130 120 110 100 90 80 70 60 50 40 30 20 10 0
δ (ppm)

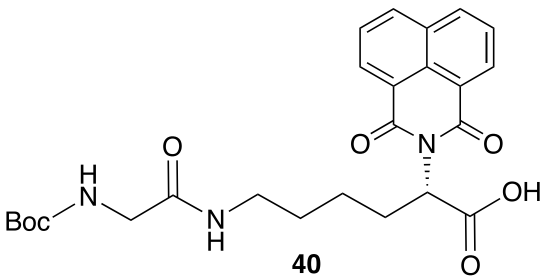

40

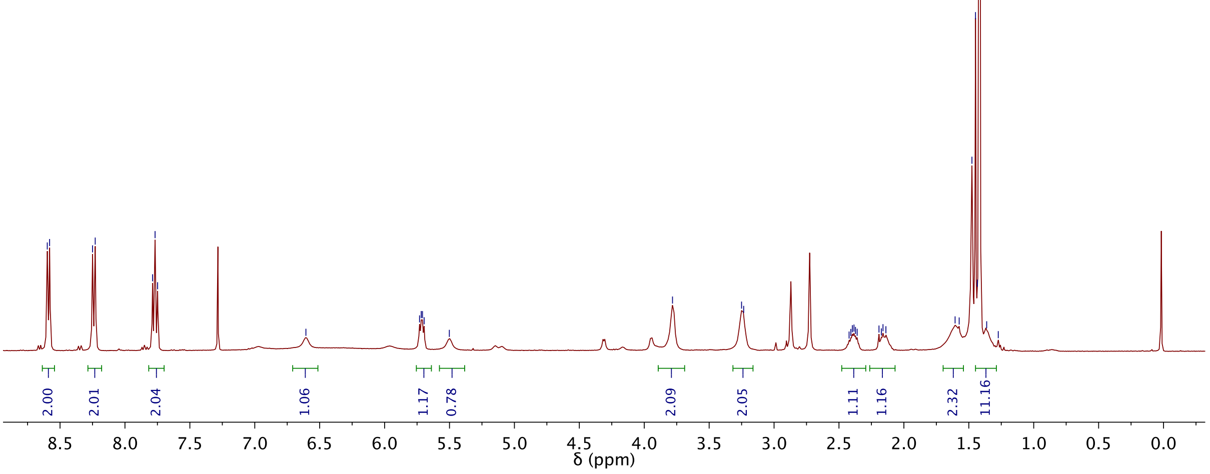
8.60
8.58
8.25
8.23
7.79
7.77
7.75
6.61
5.73
5.72
5.71
5.70
5.50
3.78
3.25
3.23
2.42
2.41
2.40
2.38
2.37
2.36
2.19
2.17
2.16
2.14
1.61
1.57
1.48
1.45
1.43
1.42
1.36
1.27
2.00
2.01
2.04
1.06
1.17
0.78
2.09
2.05
1.11
1.16
2.32
11.16
δ (ppm)

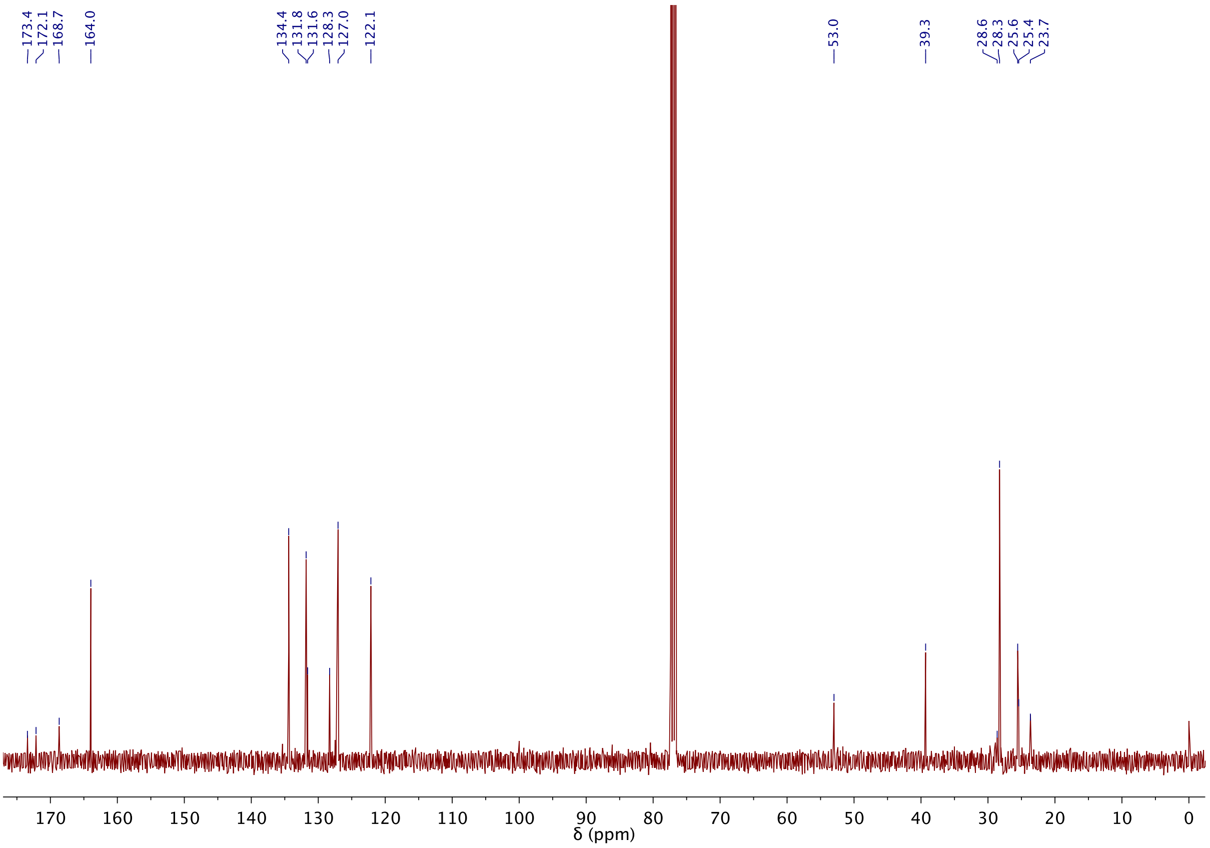
173.4
172.1
168.7
164.0
134.4
131.8
131.6
128.3
127.0
122.1
53.0
39.3
28.6
28.3
25.6
25.4
23.7
δ (ppm)

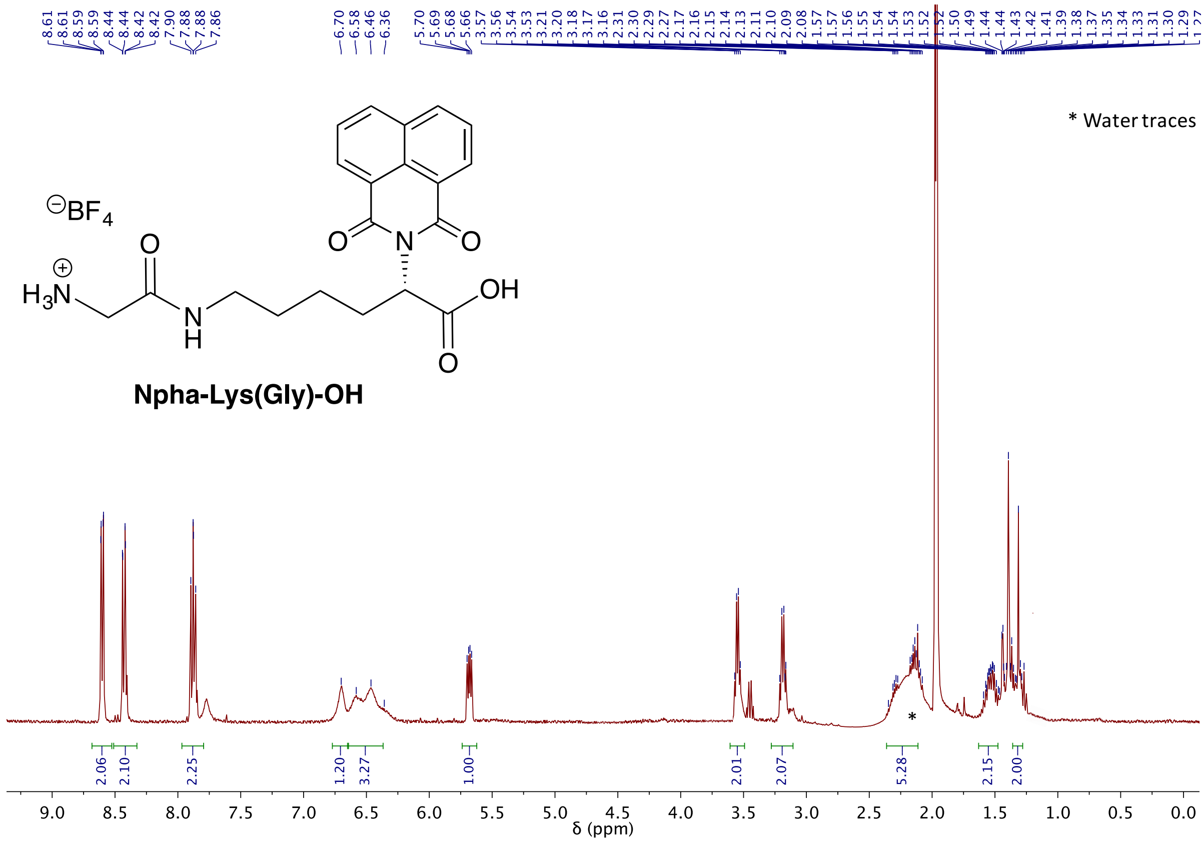

* Water traces
Npha-Lys(Gly)-OH
δ (ppm)

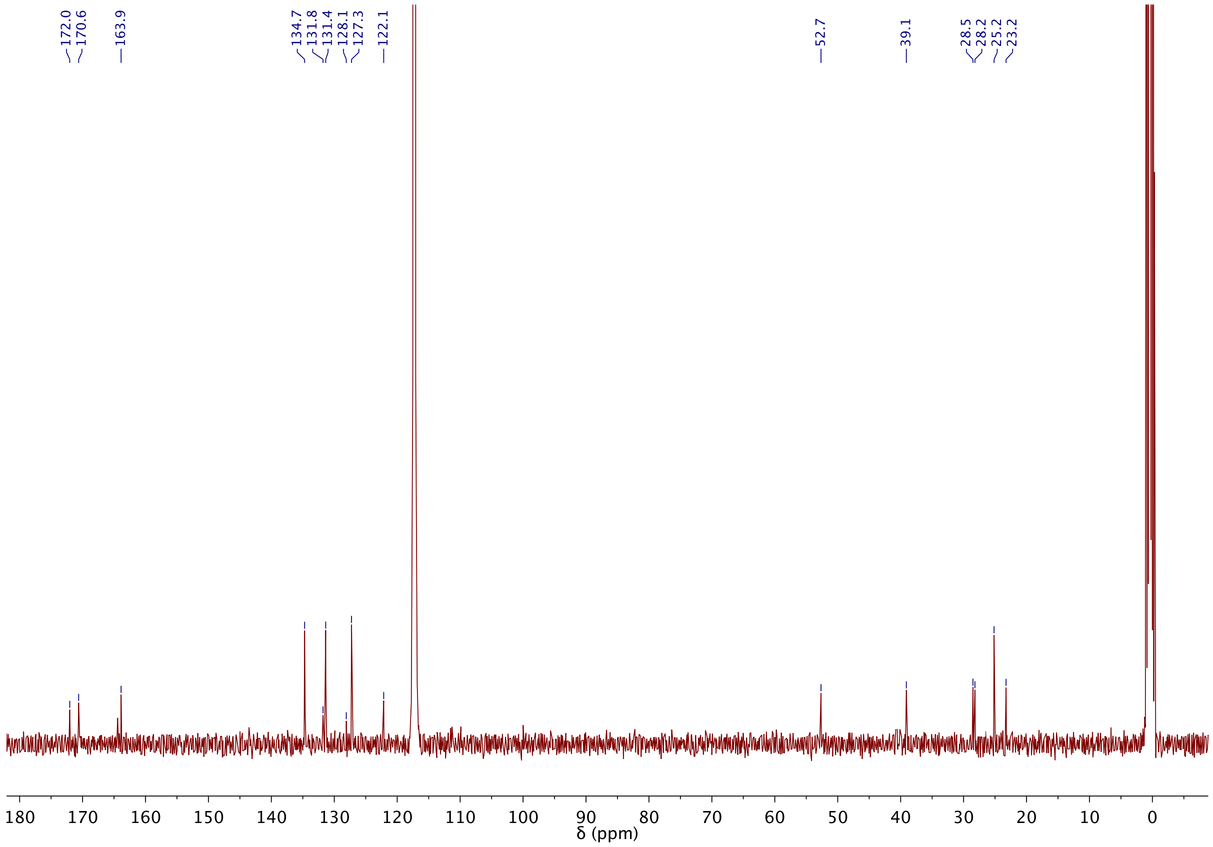

δ (ppm)

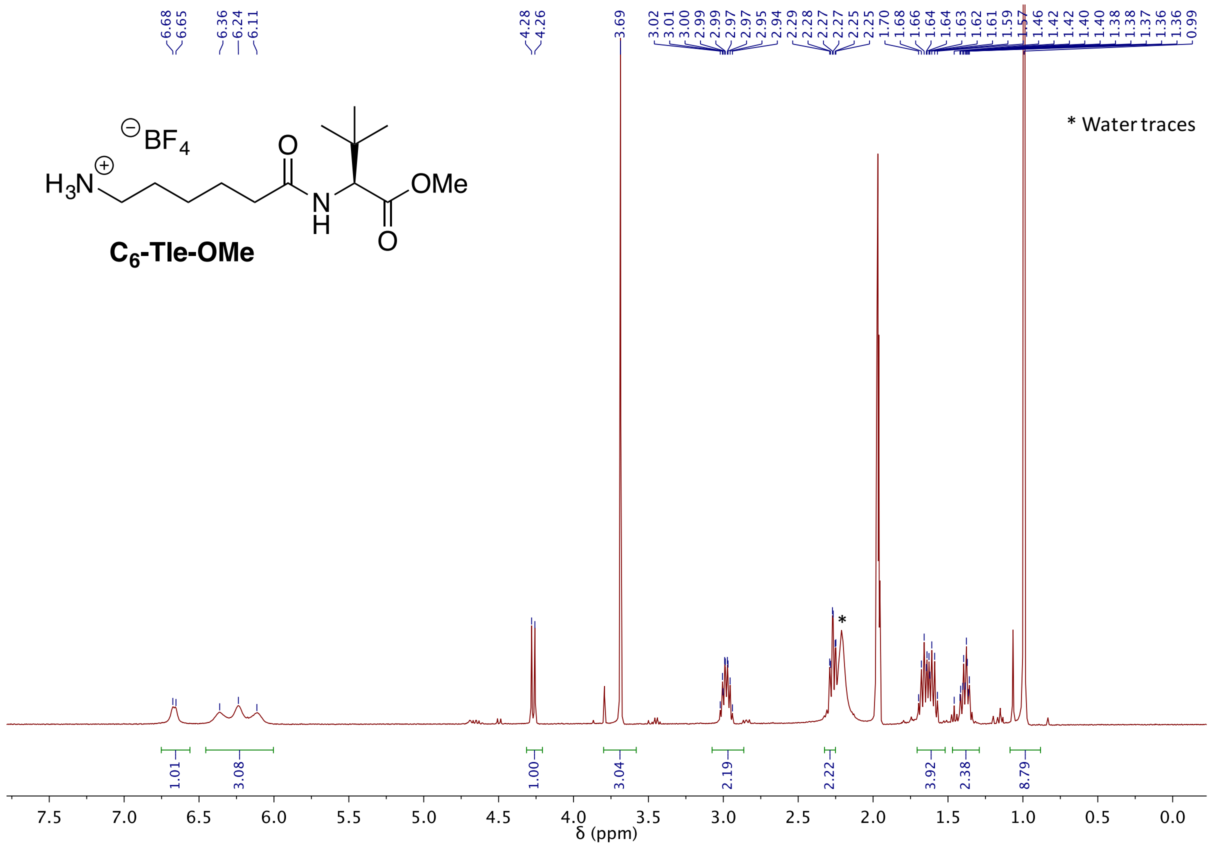
BF4
H3N
O
N
H
OMe
O
C6-Tle-OMe
* Water traces
δ (ppm)

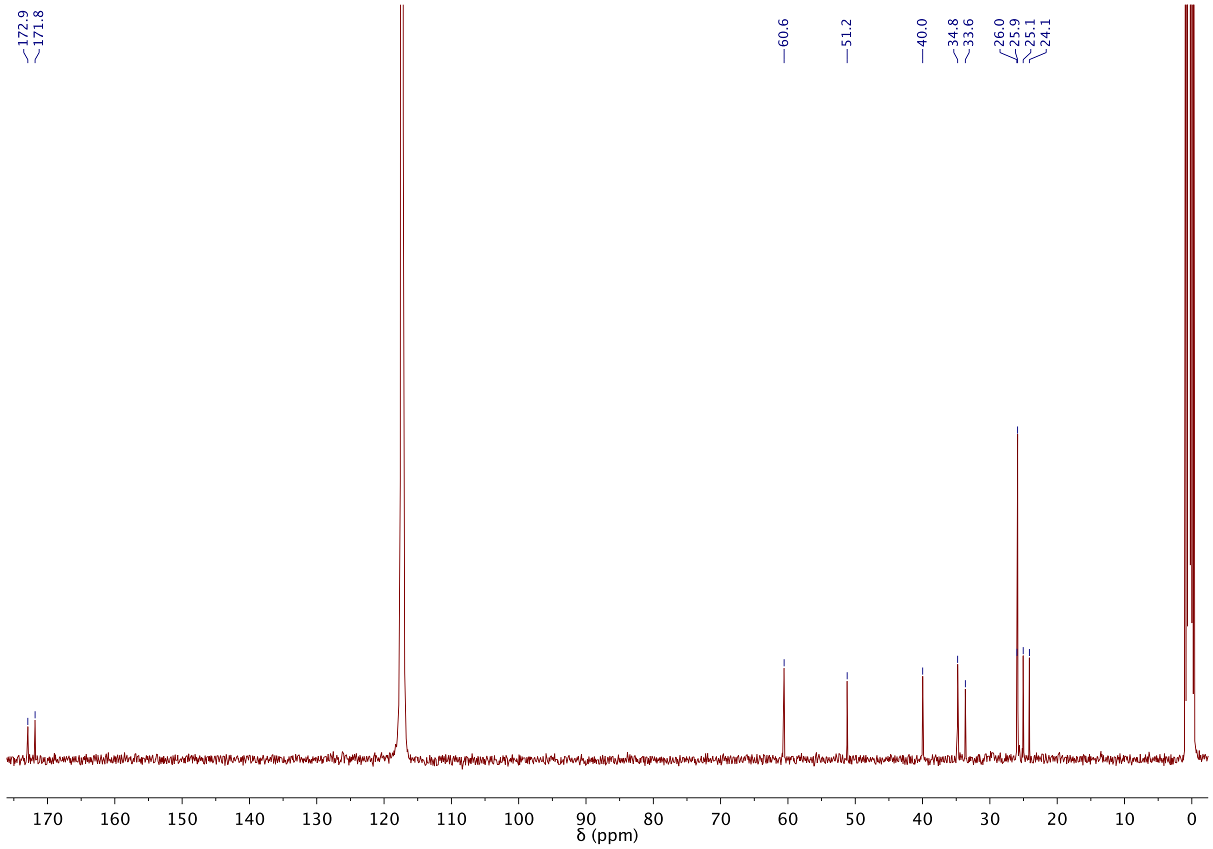
δ (ppm)

14. ^{1}H and $^{13}C\{H\}$ NMR spectra of epoxides

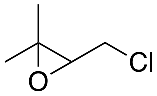

P1

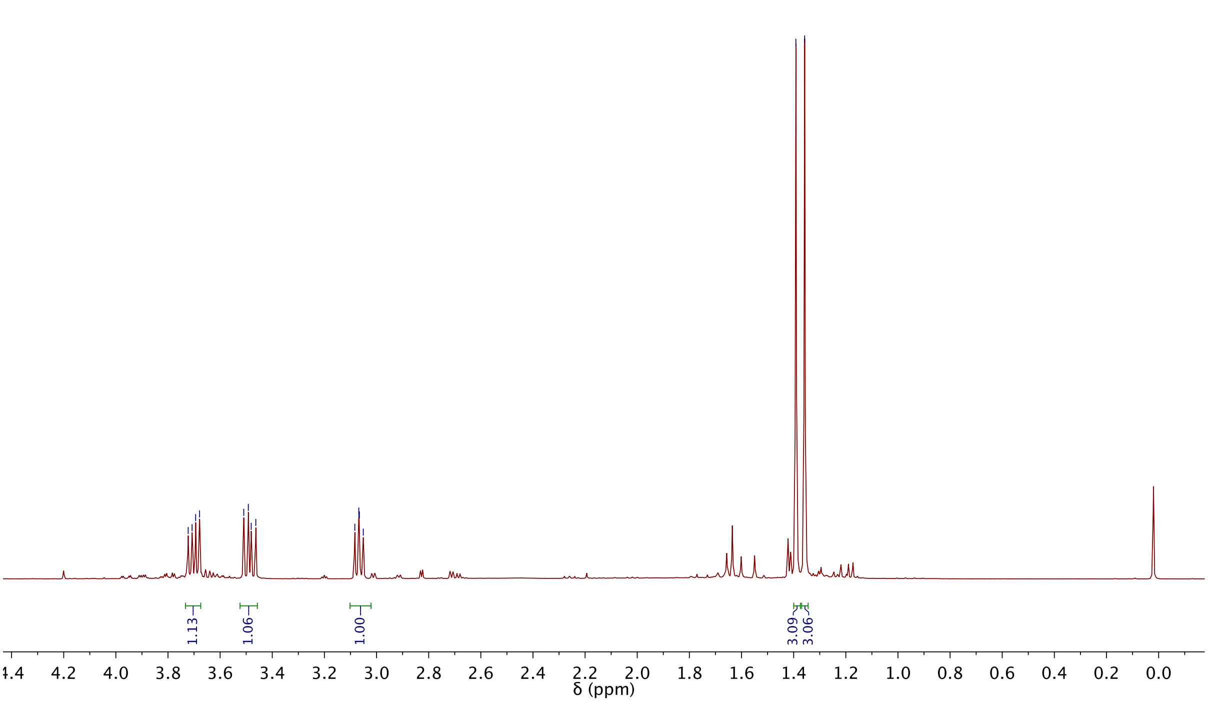

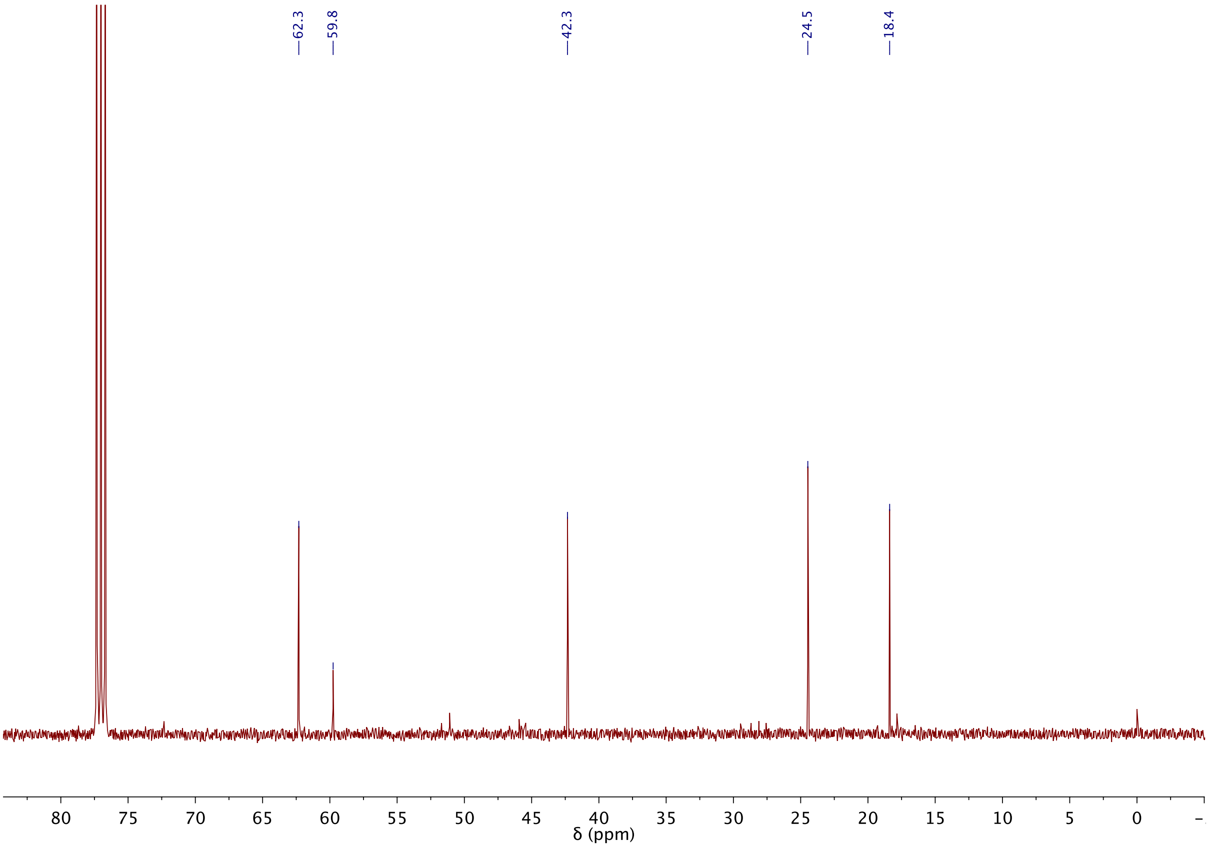

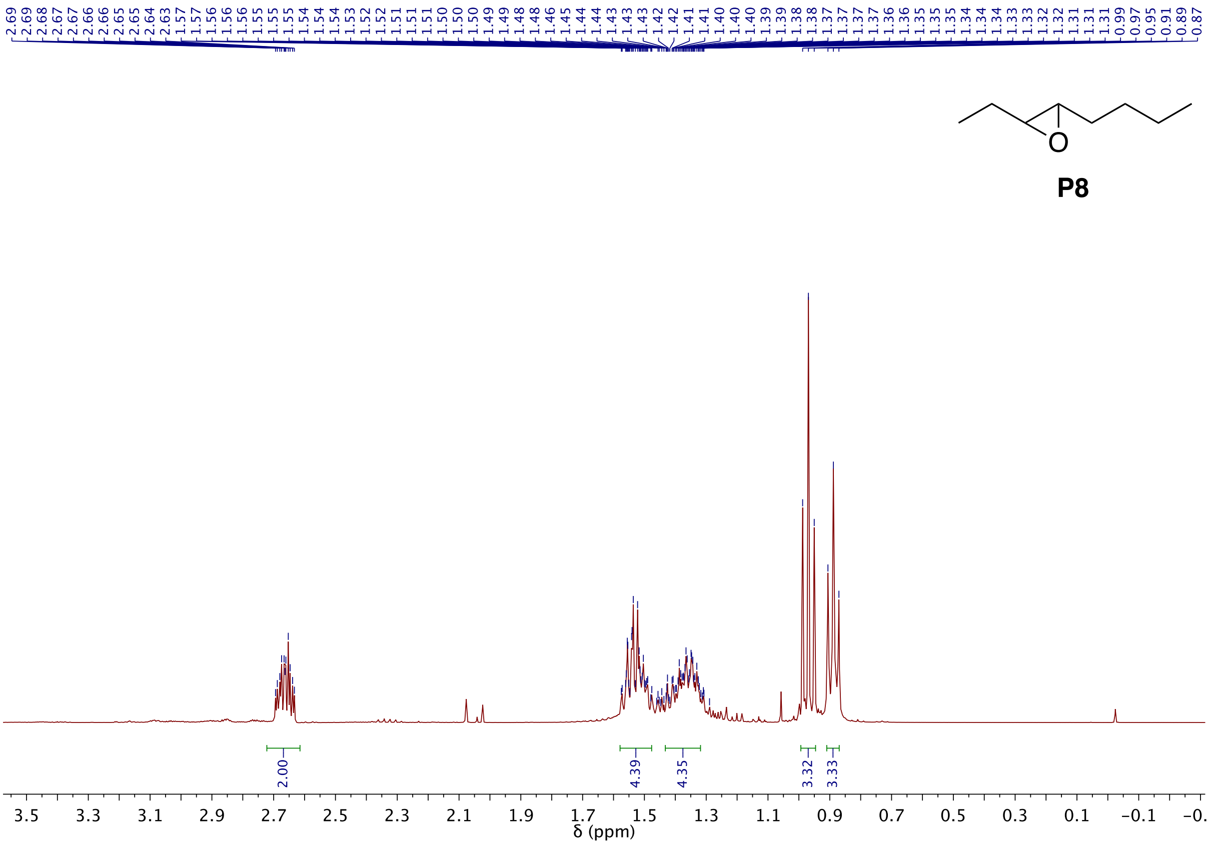

P8

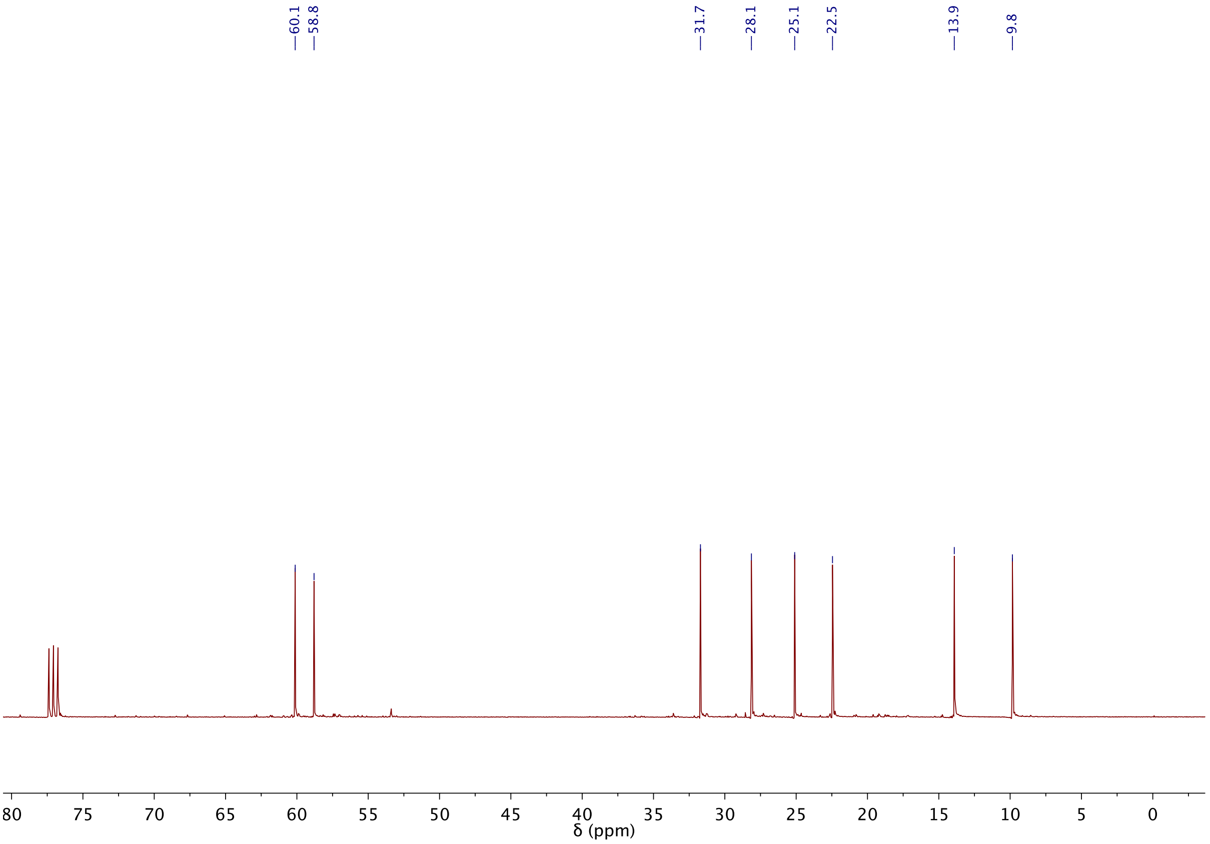

15. GC traces of epoxides

Cyclosyl-B column. GC temperature program: starting at 75°C for 0.5 minutes, then raised to 180°C at 5°C/min and hold 3.5 minutes. The analysis time was 22 minutes. t_1 = 4.0 min, t_2 = 4.1 min.

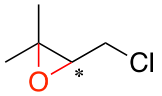

P1

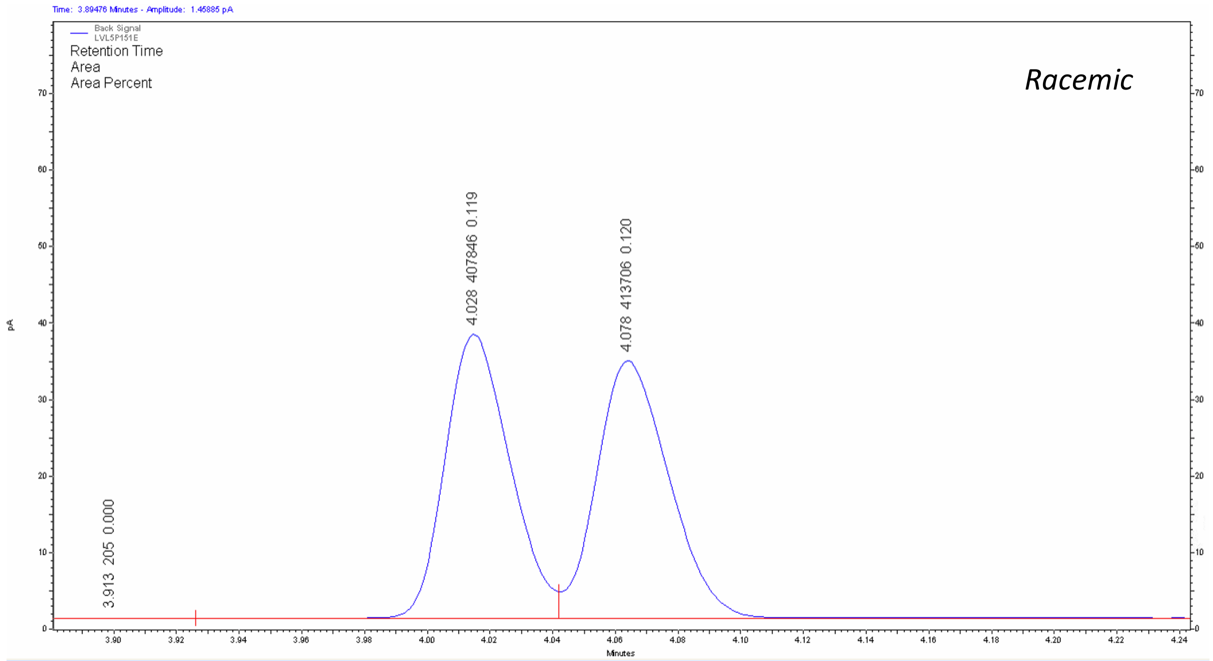

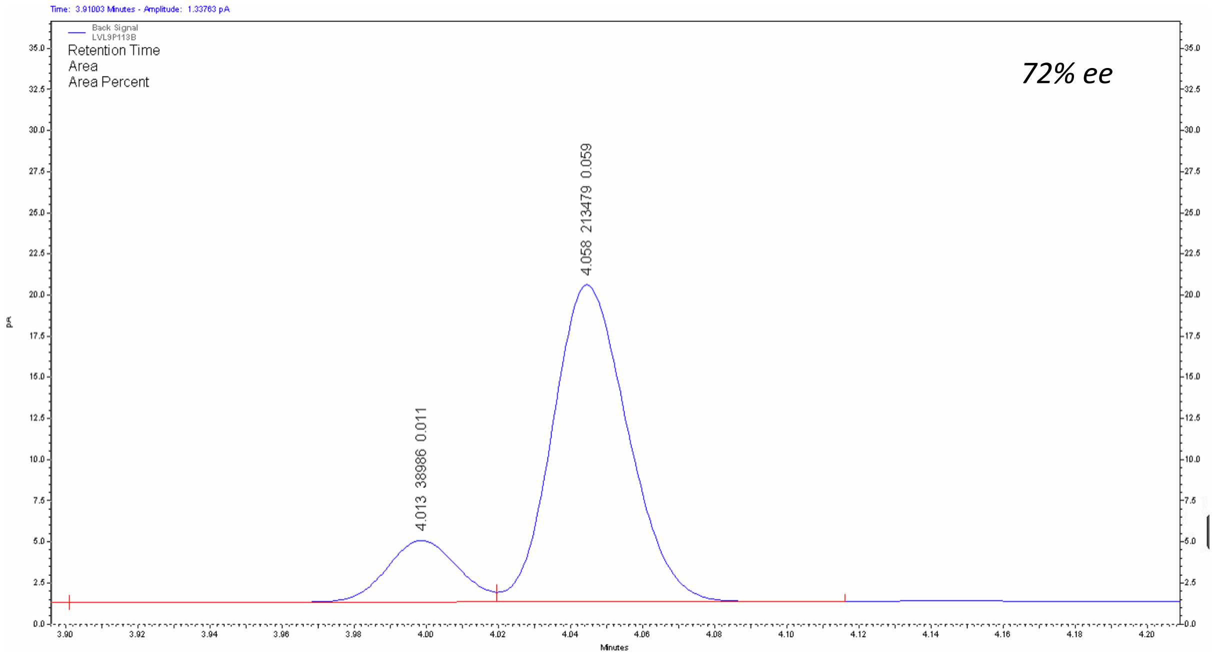

Cyclosyl-B column. GC temperature program: starting at 40°C for 15 minutes, then raised to 110°C at 2°C/min and to 180°C at 13°C/min. The analysis time was 57 minutes. t_1 = 36.6 min, t_2 = 30.9 min.

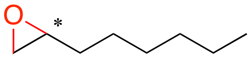

P2

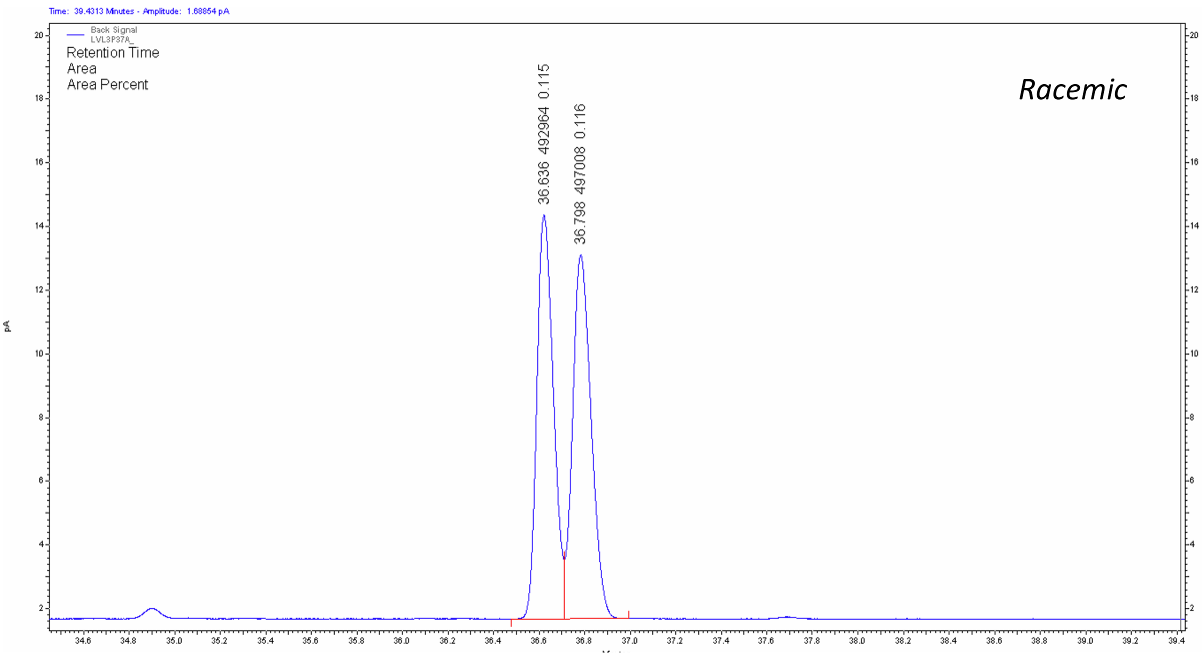

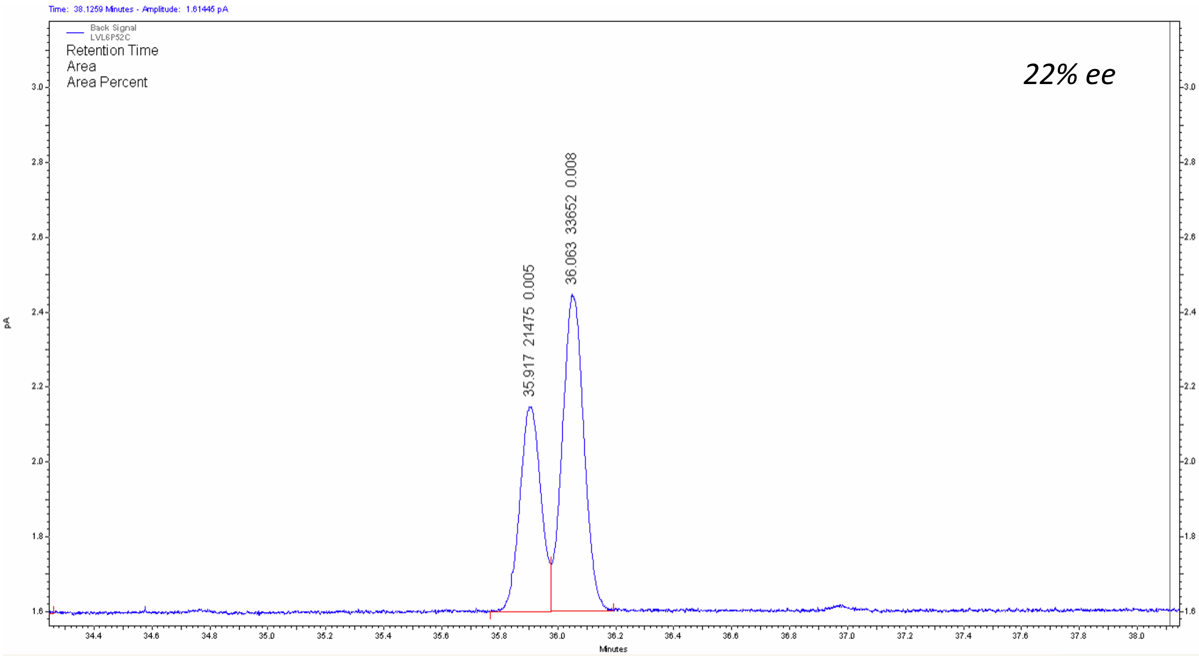

Cyclosyl-B column. GC temperature program: starting at 40°C for 15 minutes, then raised to 110°C at 2°C/min and to 180°C at 13°C/min. The analysis time was 57 minutes. t_1 = 30.8 min, t_2 = 30.9 min.

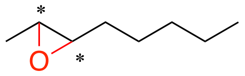

P3

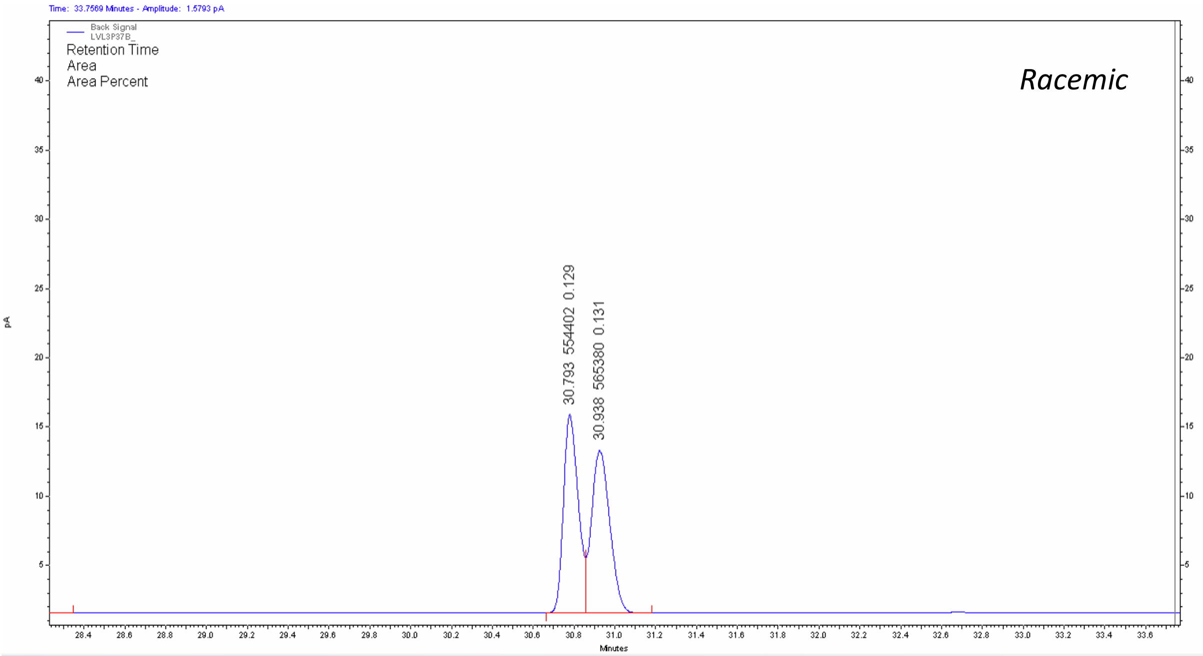

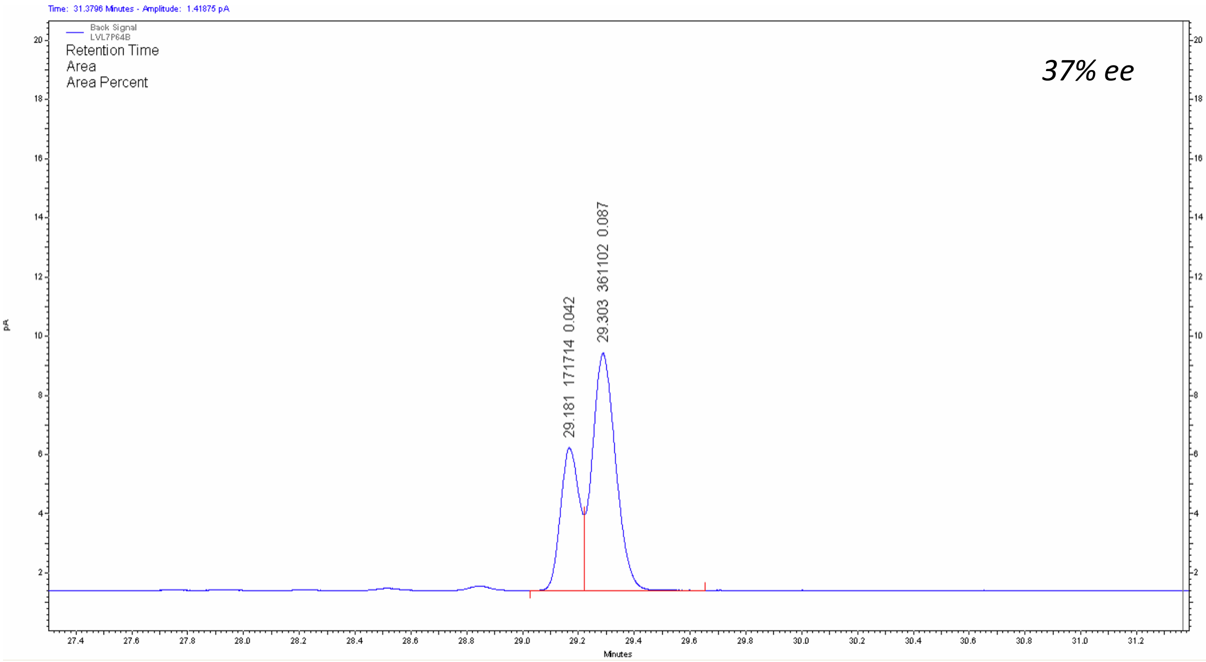

Cyclosyl-B column. GC temperature program: starting at 75°C for 0.5 minutes, then raised to 180°C at 10°C/min and hold 1 minutes. The analysis time was 12 minutes. t_1 = 5.3 min, t_2 = 5.4 min.

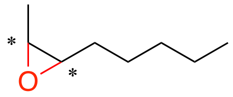

P4

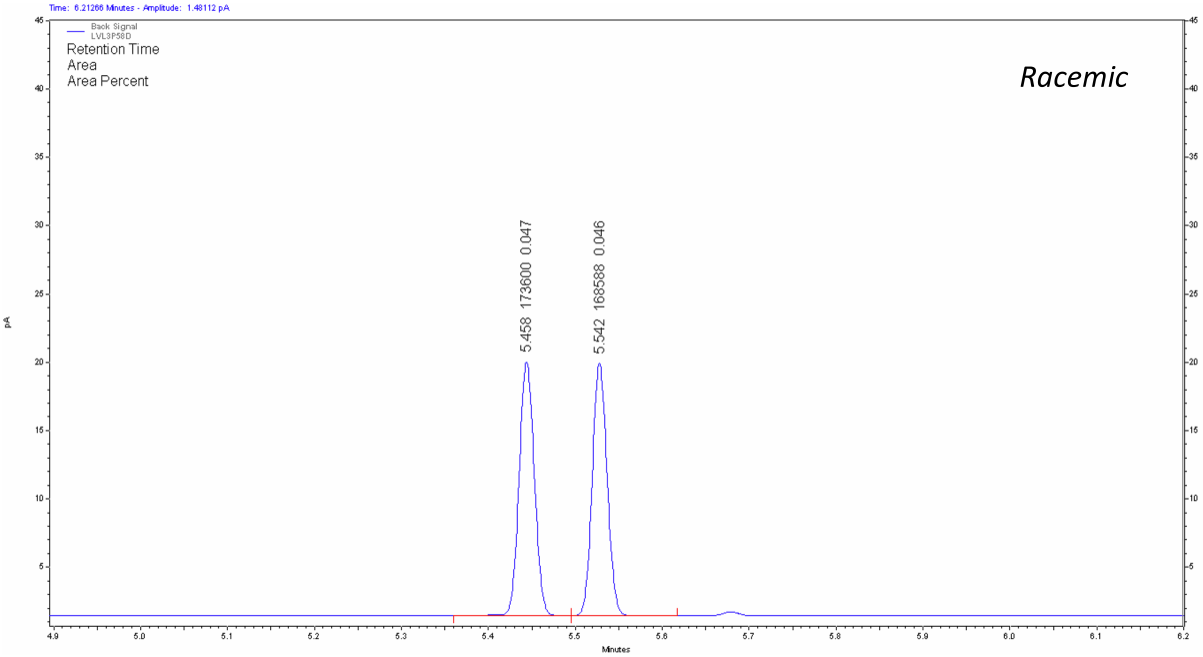

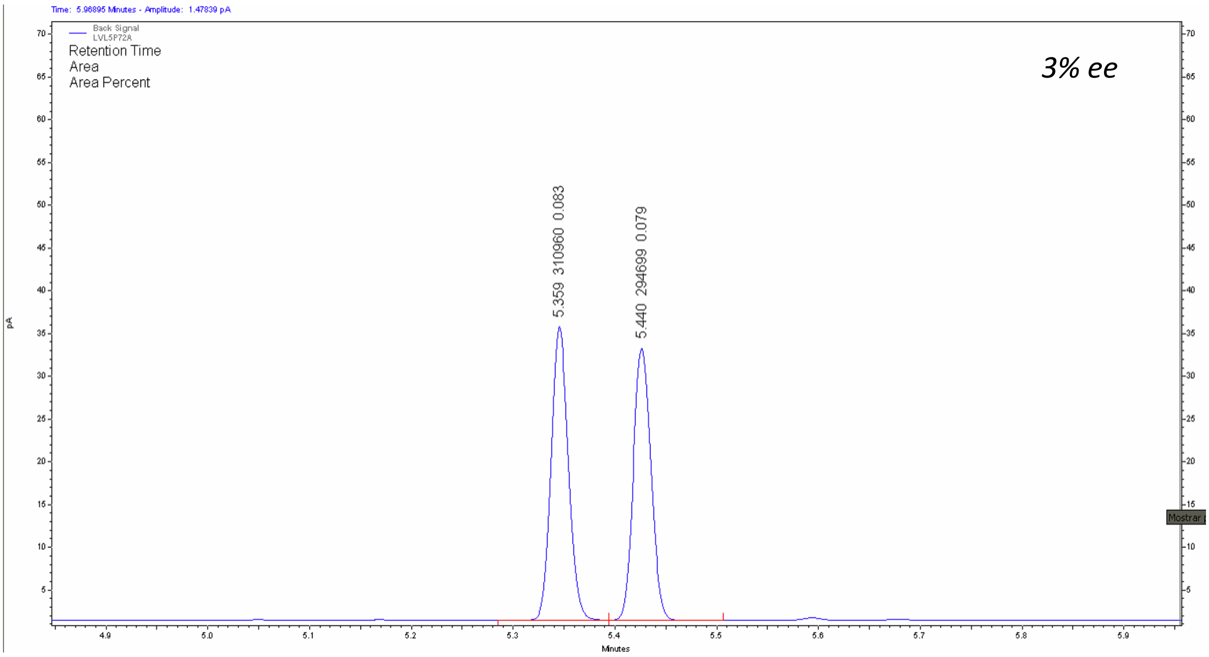

Cyclosyl-B column. GC temperature program: starting at 75°C for 0.5 minutes, then raised to 180°C at 5°C/min and hold 3.5 minutes. The analysis time was 22 minutes. t_1 = 5.7 min, t_2 = 5.8 min.

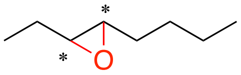

P5

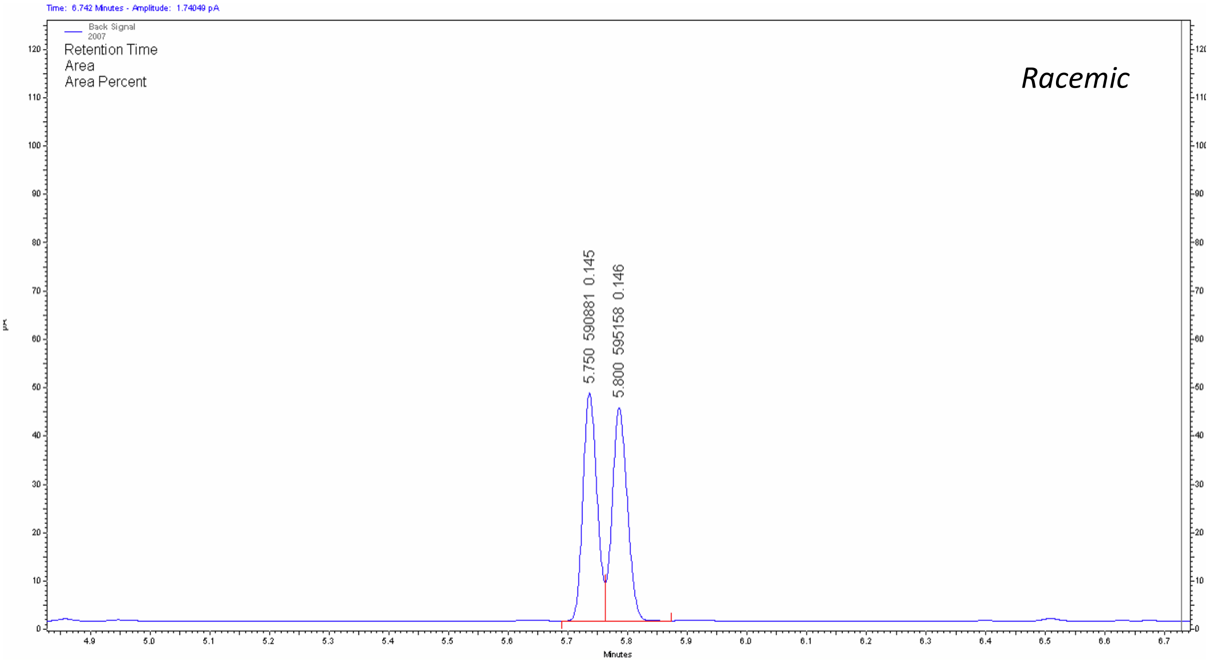

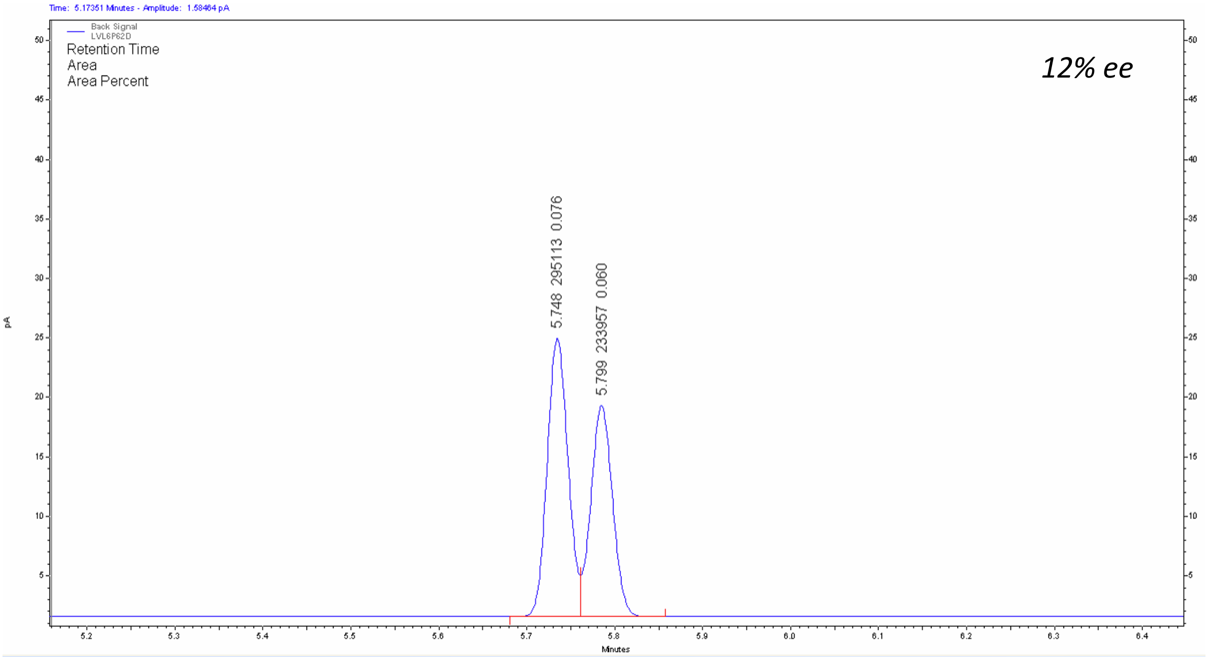

Cyclosyl-B column. GC temperature program: starting at 40°C, then raised to 80°C at 1°C/min and to 180°C at 20°C/min. The analysis time was 45 minutes. t_1 = 24.9 min, t_2 = 25.1 min.

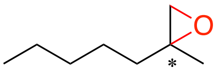

P6

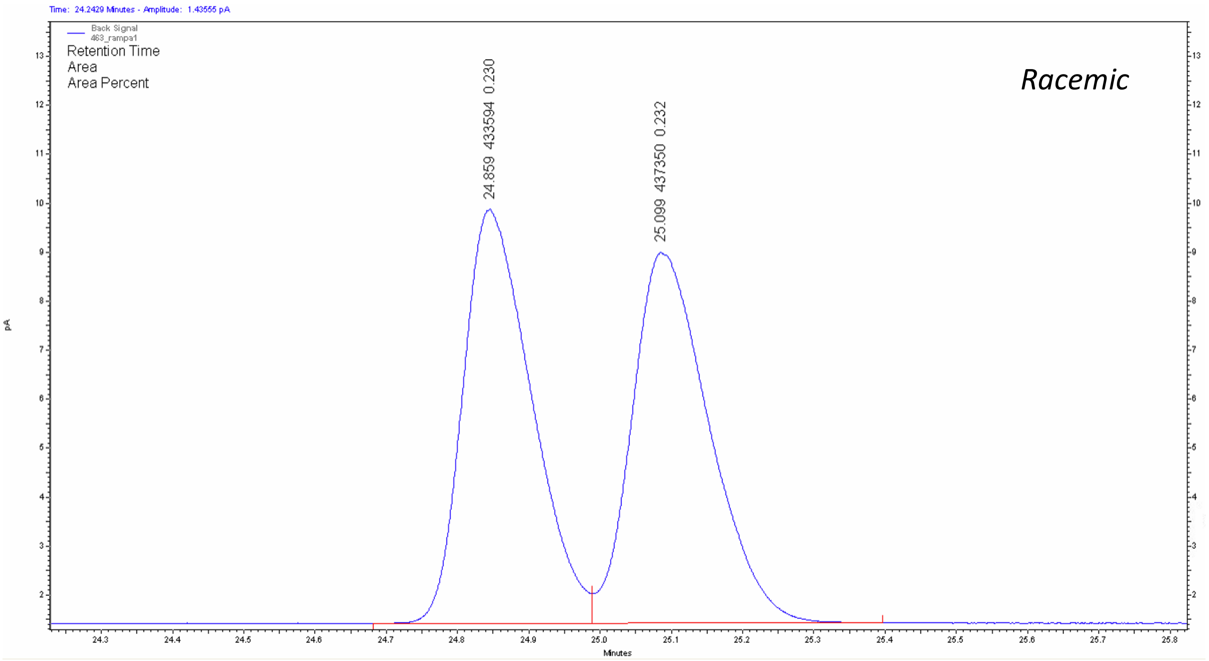

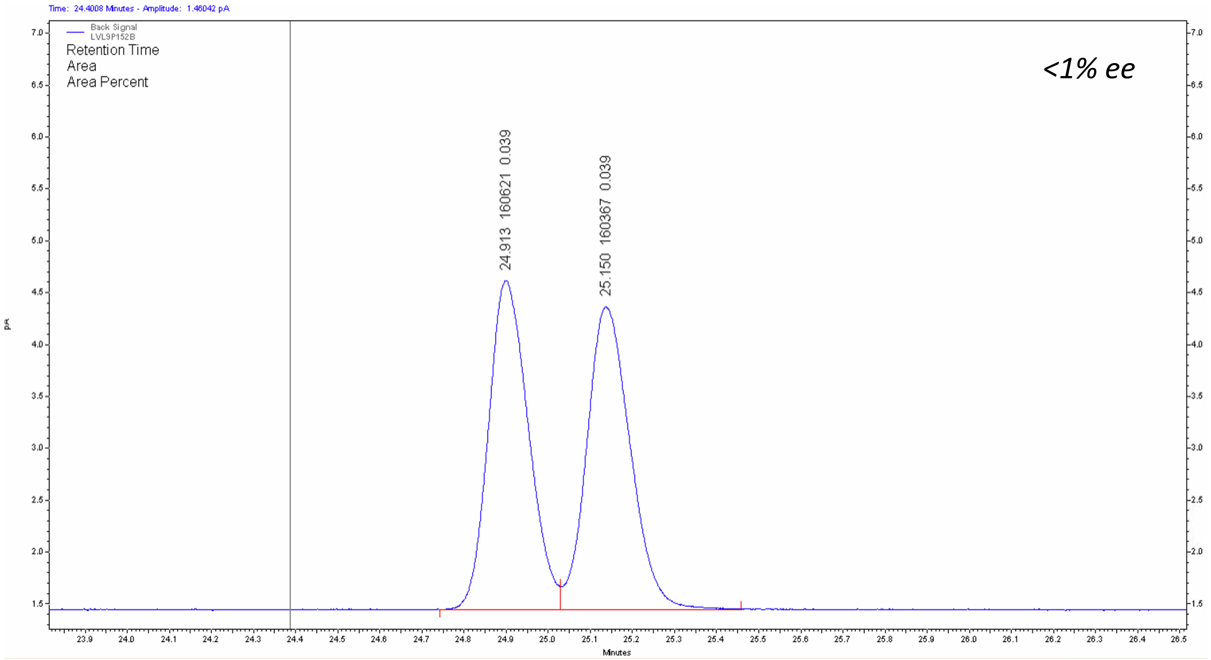

Cyclosyl-B column. GC temperature program: starting at 75°C for 0.5 minutes, then raised to 180°C at 10°C/min and hold 1 minutes. The analysis time was 12 minutes. t_1 = 6.7 min, t_2 = 7.0 min.

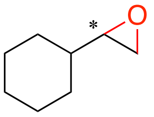

P7

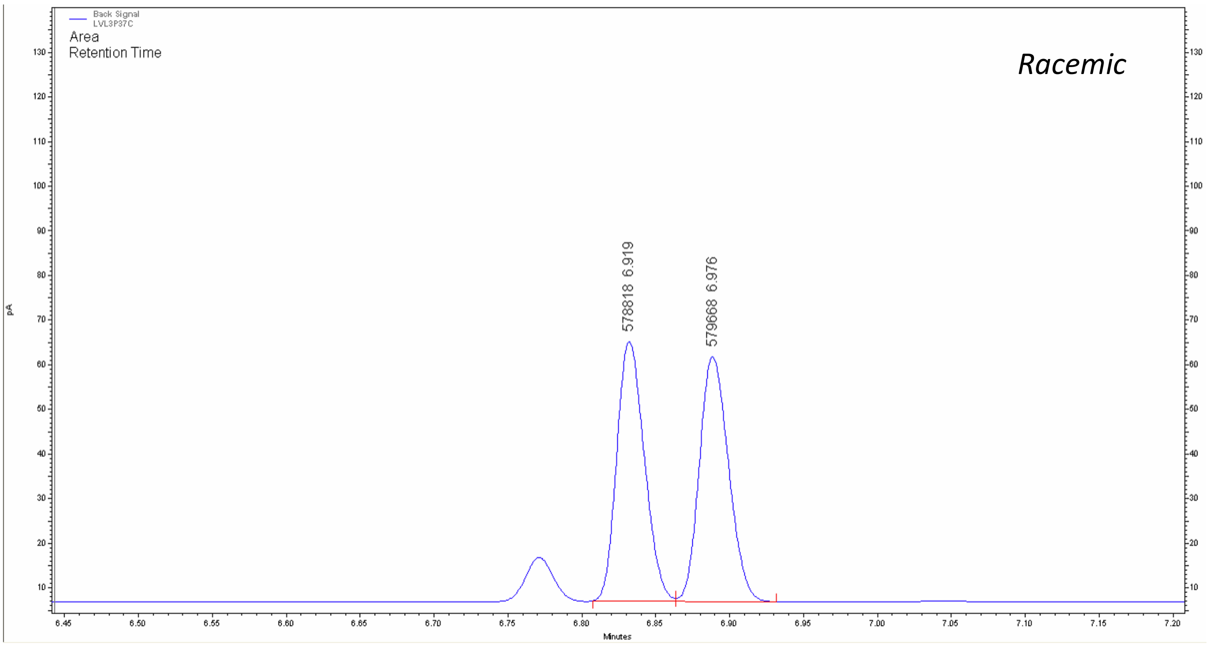

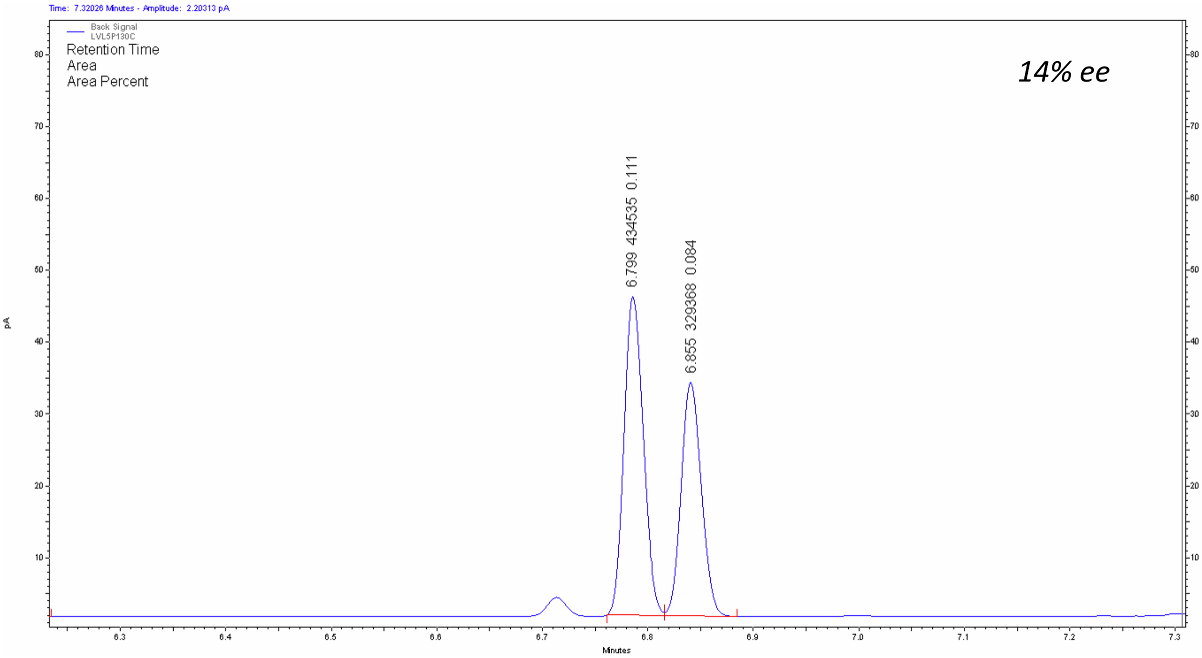

Cyclosyl-B column. GC temperature program: starting at 40°C for 10 minutes, then raised to 50°C at 0.5°C/min and hold 10 minutes. Then raised to 180°C at 20°C/min. The analysis time was 54 minutes. t_1 = 28.7 min, t_2 = 29.0 min.

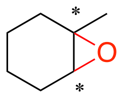

P8

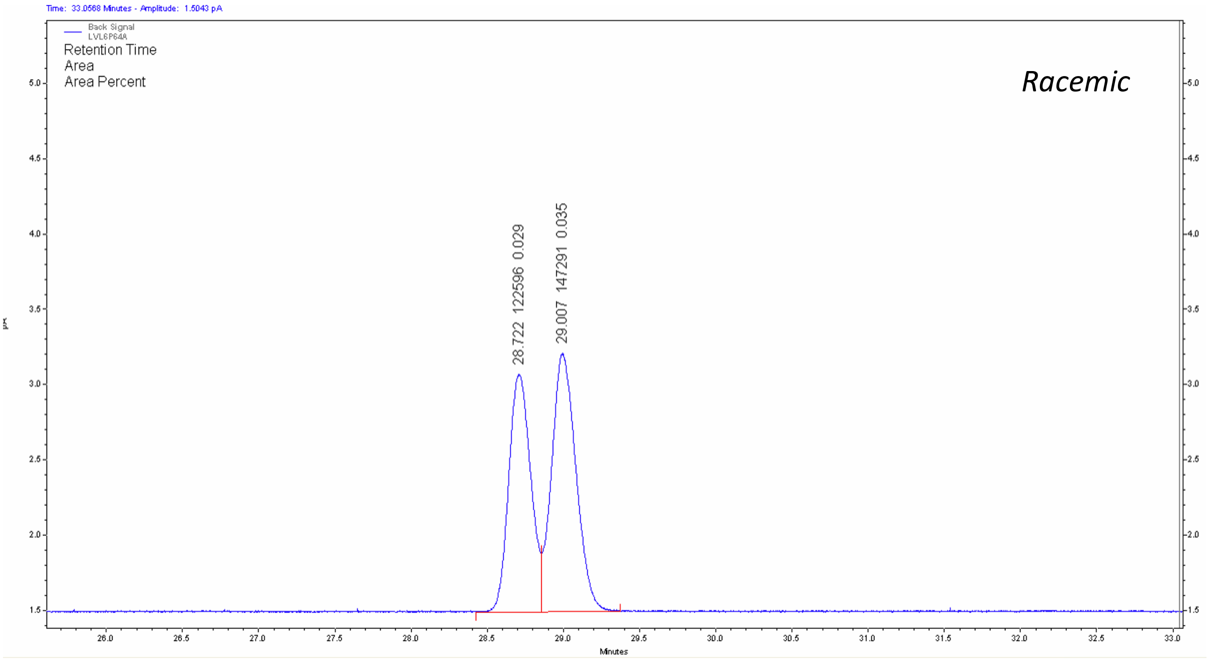

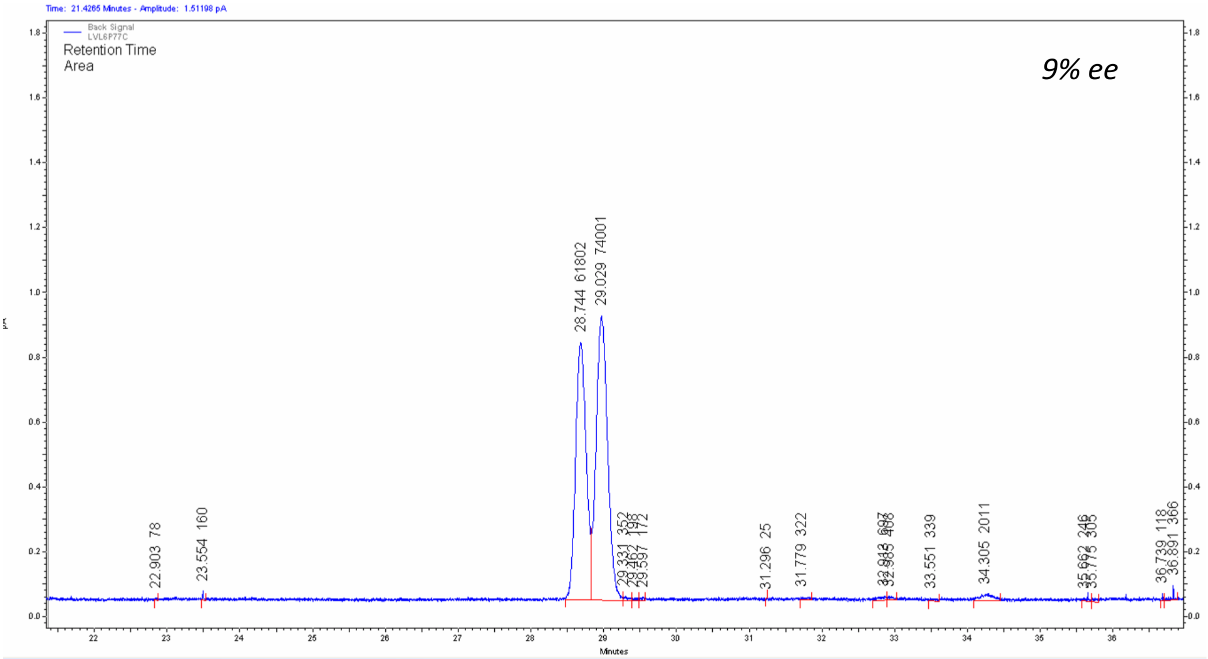

Cyclosyl-B column. GC temperature program: starting at 40°C for 10 minutes, then raised to 180°C at 10°C/min and hold 1 minute. The analysis time was 25 minutes. t_1 = 8.5 min, t_2 = 9.0 min.

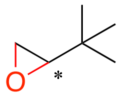

P9

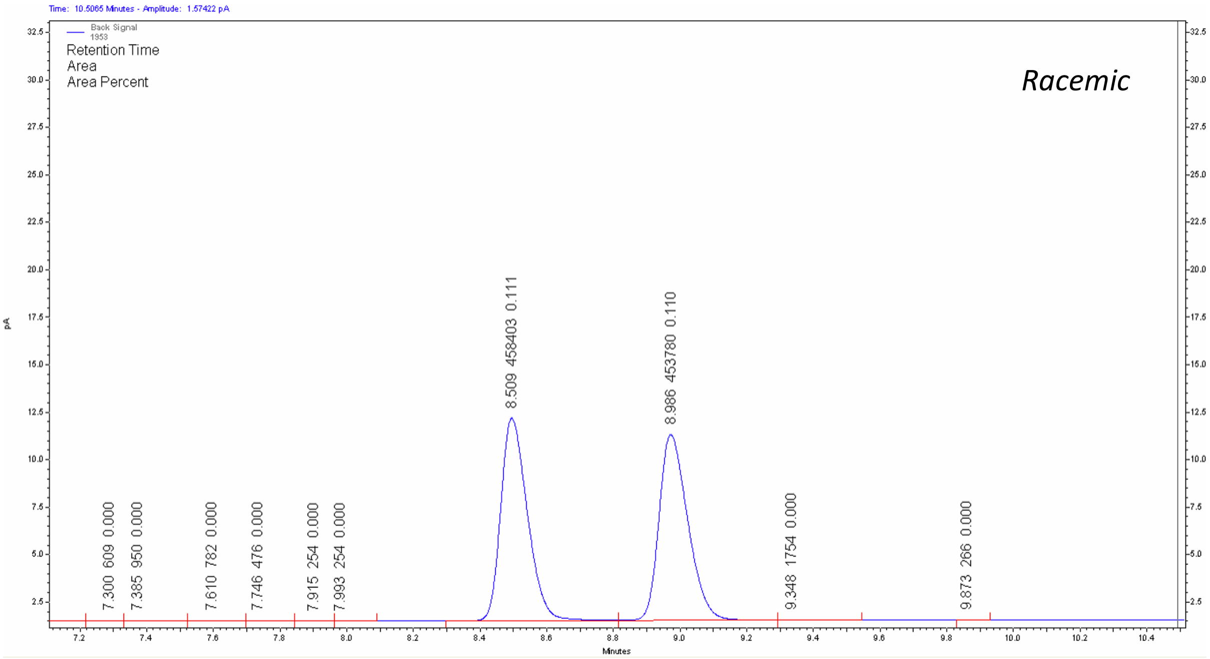

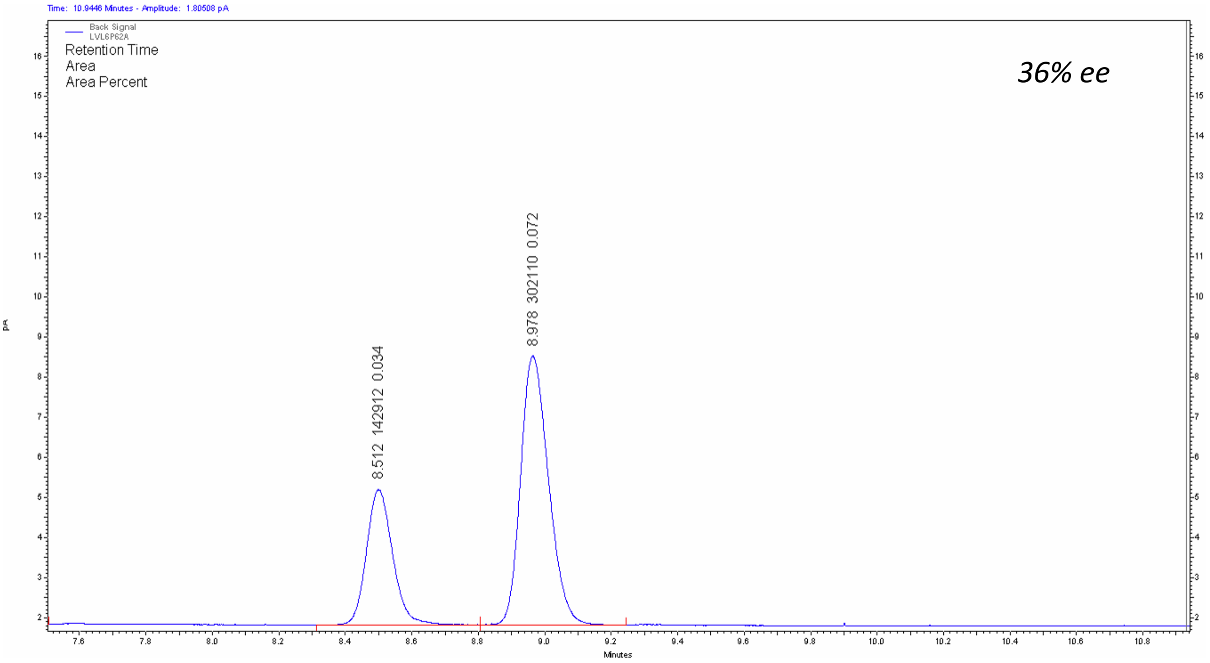

Cyclosyl-B column. GC temperature program: starting at 40°C, then raised to 80°C at 2°C/min and to 180°C at 20°C/min. The analysis time was 25 minutes. t_1 = 16.2 min, t_2 = 16.6 min.

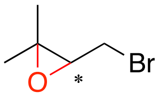

P10

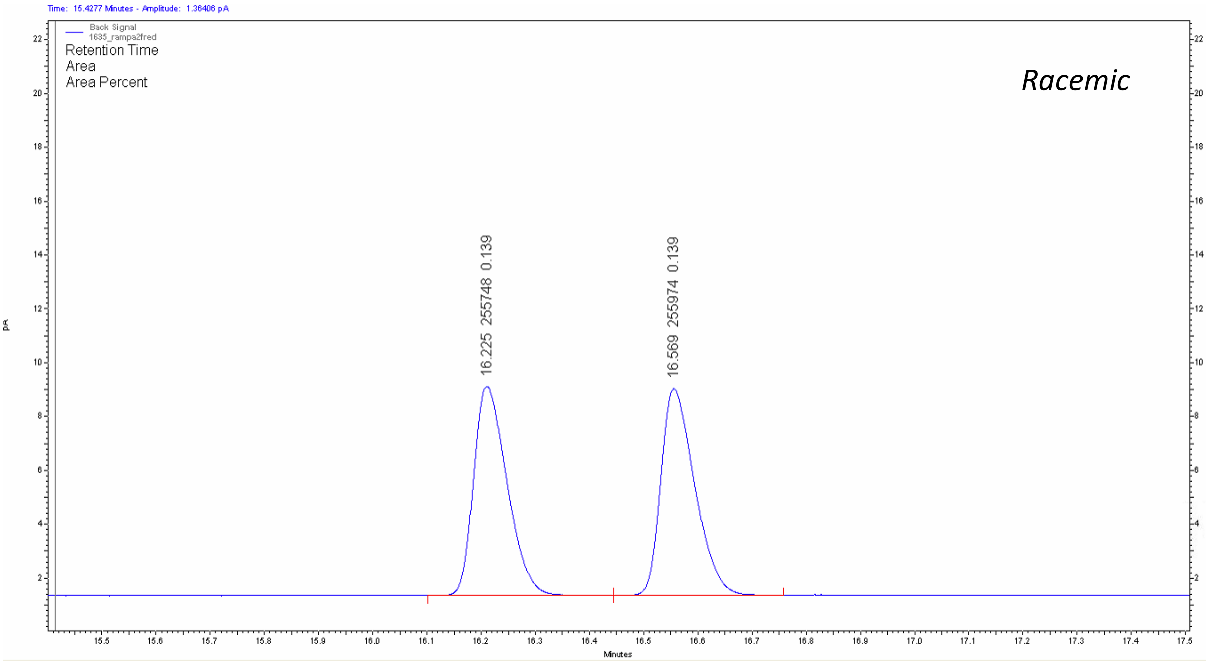

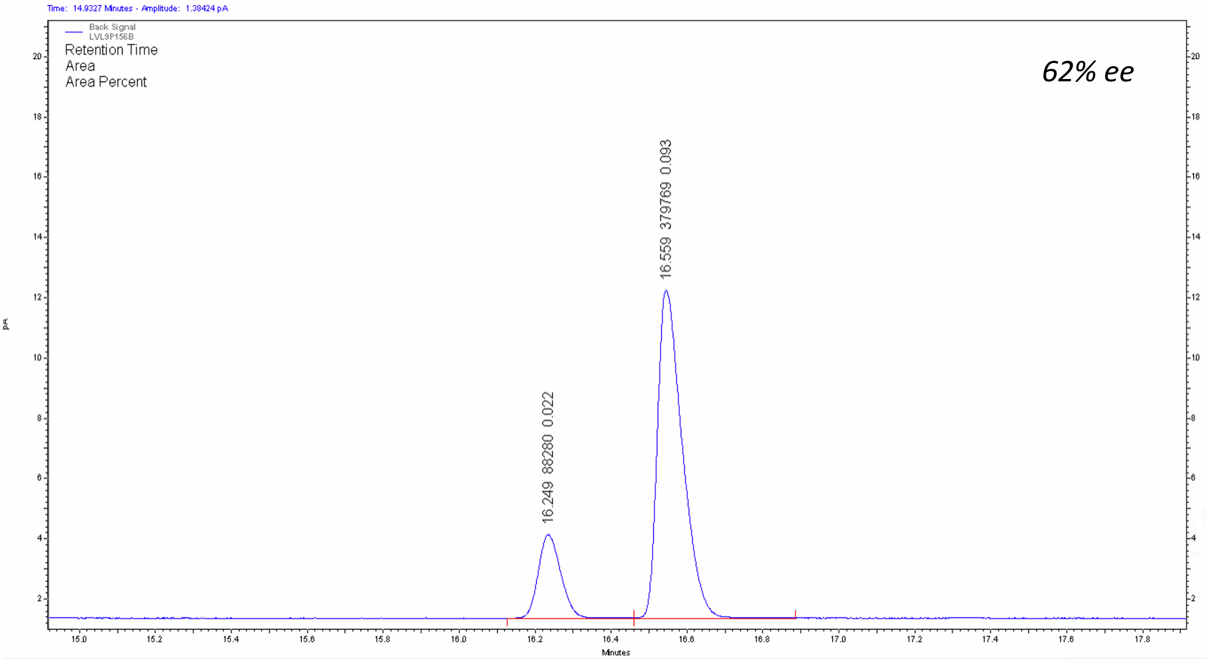

Cyclosyl-B column. GC temperature program: starting at 75°C for 0.5 minutes, then raised to 180°C at 10°C/min and hold 1 minutes. The analysis time was 12 minutes. t_1 = 7.3 min, t_2 = 7.4 min.

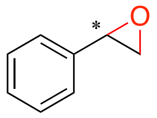

P11

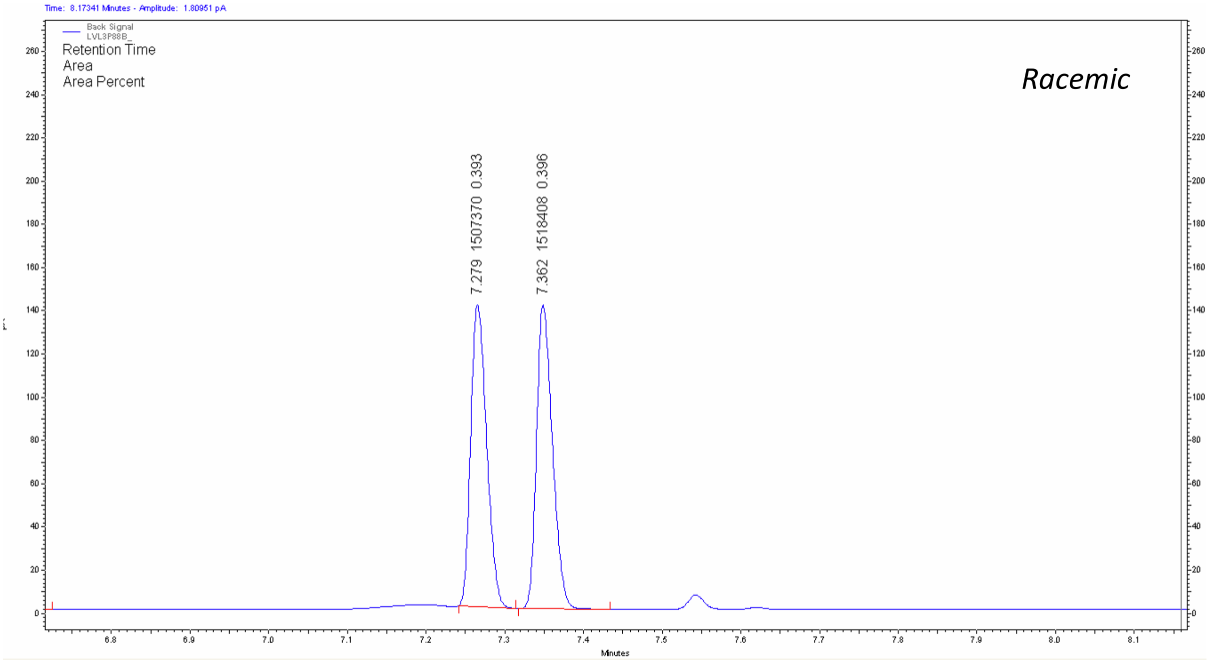

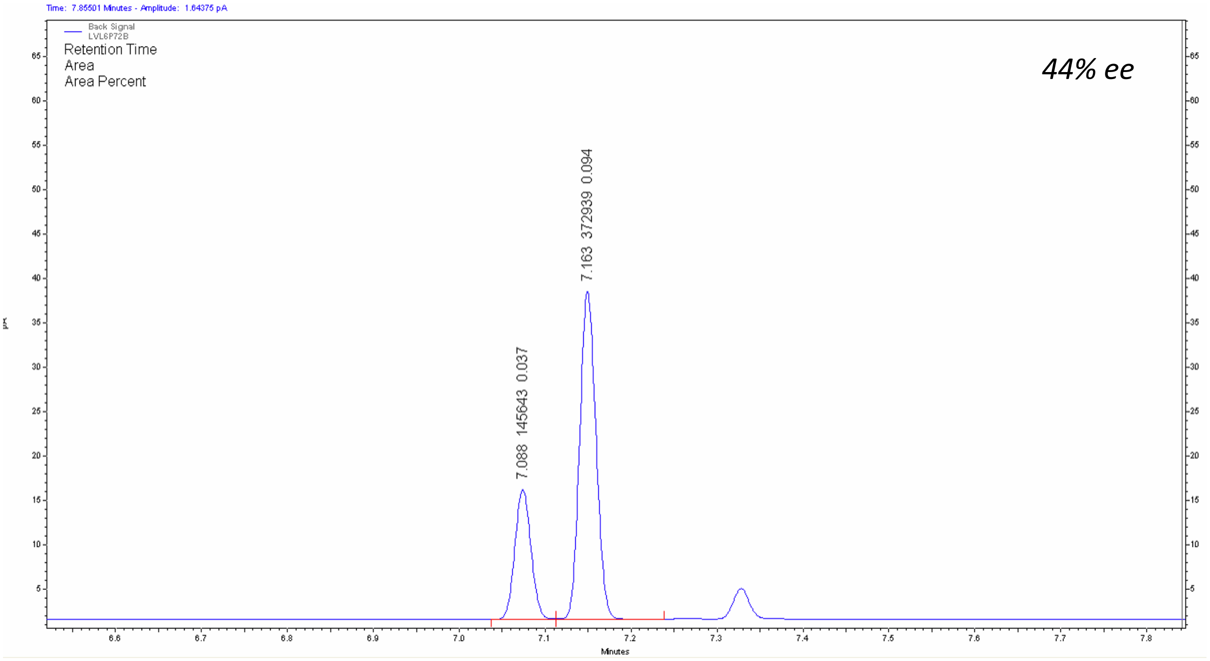

Cyclosyl-B column. GC temperature program: starting at 75°C for 0.5 minutes, then raised to 200°C at 2°C/min and to 240°C at 20°C/min. The analysis time was 65 minutes. t_1 = 58.0 min, t_2 = 58.1 min.

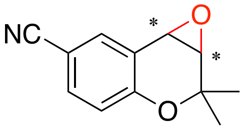

P12

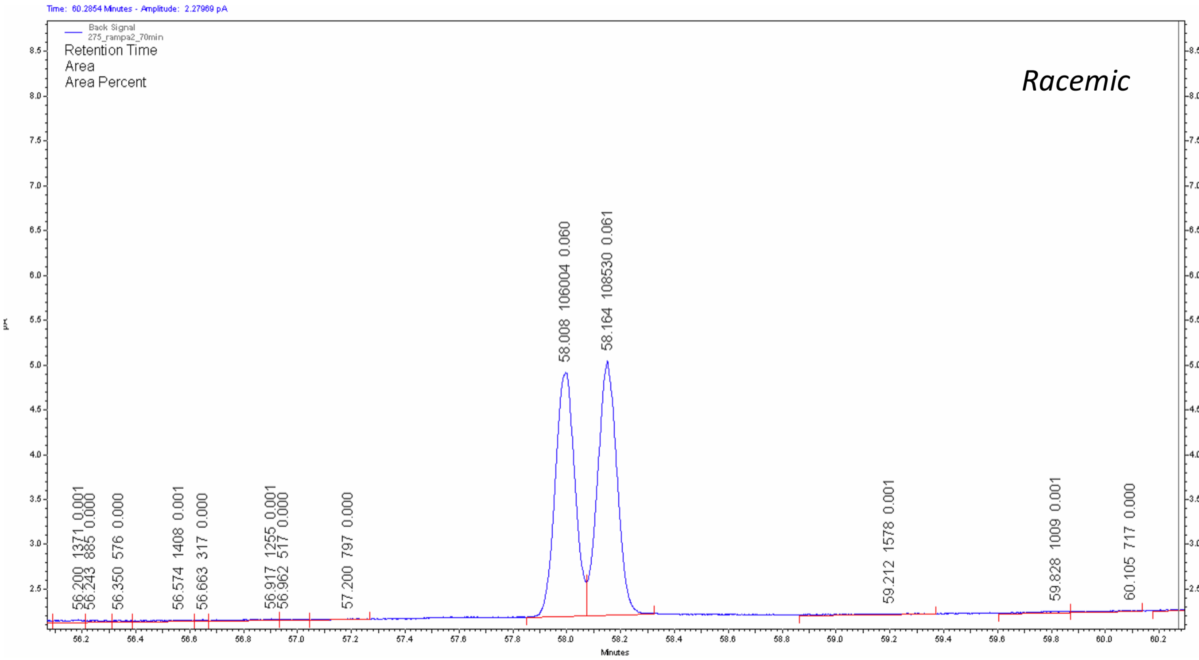

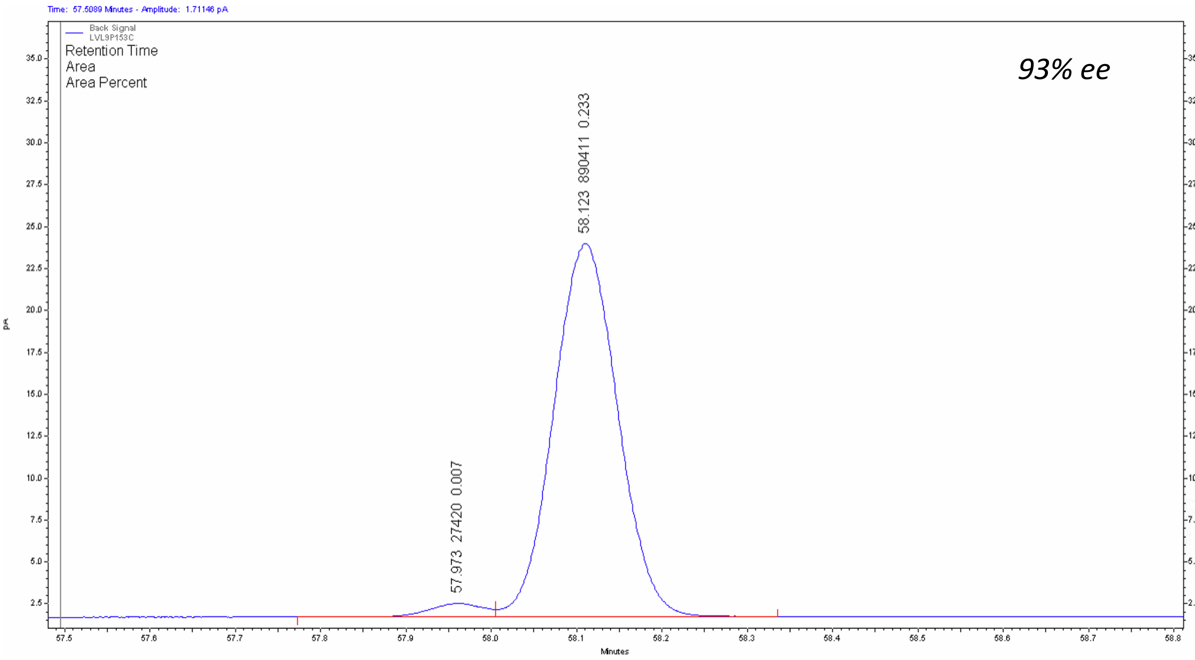

Cyclosyl-B column. GC temperature program: starting at 75°C for 0.5 minutes, then raised to 180°C at 5°C/min and hold 3.5 minutes. The analysis time was 22 minutes. t_1 = 19.5 min, t_2 = 19.6 min.

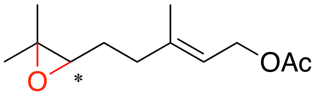

P13

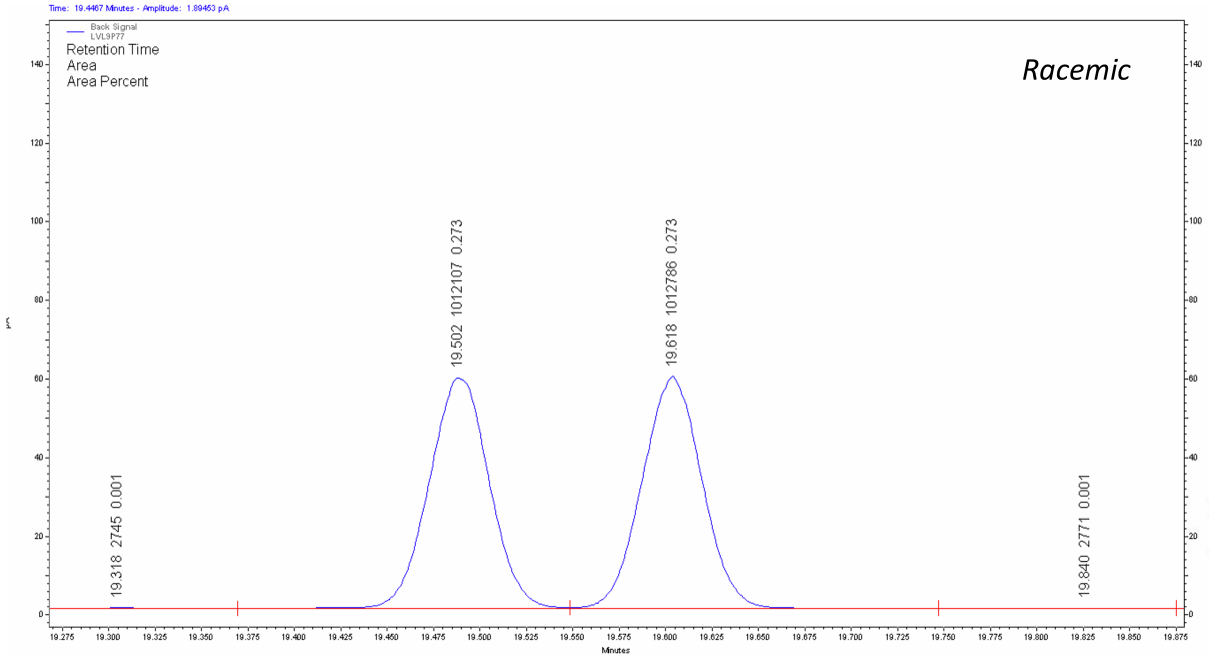

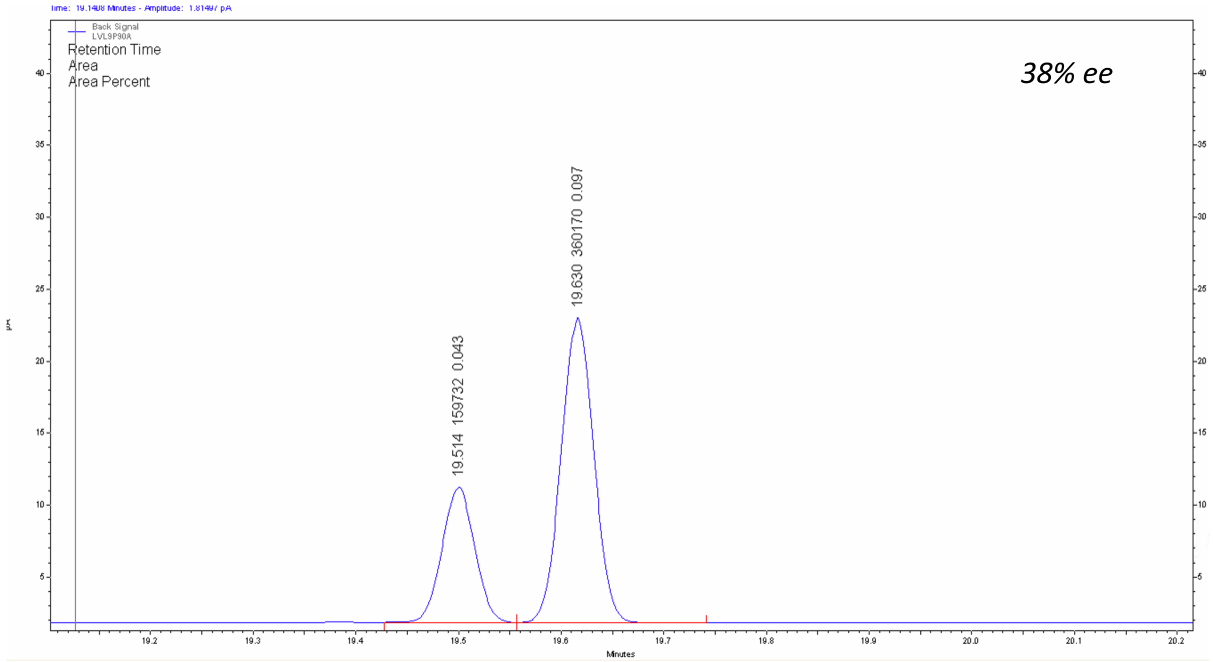

16. References

[1] Milan, M.; Bietti, M.; Costas, M. *ACS Cent. Sci.* **2017,** *3*, 196-204.

[2] Olivo, G.; Farinelli, G.; Barbieri, A.; Lanzalunga, O.; Di Stefano, S.; Costas, M. *Angew. Chem. Int. Ed.* **2017,** *56*, 16347-16351.

[3] Olivo, G.; Capocasa, G.; Lanzalunga, O.; Di Stefano, S.; Costas, M. *Chem. Commun.* **2019,** *55*, 917-920.

[4] Cussó, O.; Cianfanelli, M.; Ribas, X.; Klein Gebbink, R. J.; Costas, M. *J. Am. Chem. Soc.* **2016,** *138*, 2732-8.

[5] López-Rodríguez, R.; Ros, A.; Fernández, R.; Lassaletta, J. M. *J. Org. Chem.* **2012,** *77*, 9915-9920.

[6] Yoon, S.-H.; Seo, S.; Lee, Y.; Hwang, S.; Kim, D. Y. *Bioorg. Med. Chem. Lett.* **1998,** *8*, 1909-1912.

[7] Cussó, O.; Garcia-Bosch, I.; Ribas, X.; Lloret-Fillol, J.; Costas, M. *J. Am. Chem. Soc.* **2013,** *135*, 14871-8.

[8] Olivo, G.; Capocasa, G.; Ticconi, B.; Lanzalunga, O.; Di Stefano, S.; Costas, M. *Angew. Chem. Int. Ed.* **2020,** *59*, 12703-12708.

[9] Vicens, L.; Bietti, M.; Costas, M. *Angew. Chem. Int. Ed.* **2021,** *60*, 4740-4746.

[10] Bhushan, B.; Lin, Y. A.; Bak, M.; Phanumartwiwath, A.; Yang, N.; Bilyard, M. K.; Tanaka, T.; Hudson, K. L.; Lercher, L.; Stegmann, M.; Mohammed, S.; Davis, B. G. *J. Am. Chem. Soc.* **2018,** *140*, 14599-14603.

[11] Srinivasan, B.; Huang, X. *Chirality* **2008,** *20*, 265-277.

[12] Osberger, T. J.; Rogness, D. C.; Kohrt, J. T.; Stepan, A. F.; White, M. C. *Nature* **2016,** *537*, 214-219.

[13] Hata, R.; Nonaka, H.; Takakusagi, Y.; Ichikawa, K.; Sando, S. *Angew. Chem. Int. Ed.* **2016,** *55*, 1765-1768.

[14] Wijeratne, A.; Xiao, J.; Reutter, C.; Furness, K. W.; Leon, R.; Zia-Ebrahimi, M.; Cavitt, R. N.; Strelow, J. M.; Van Horn, R. D.; Peng, S.-B.; Barda, D. A.; Engler, T. A.; Chalmers, M. J. *ACS Med. Chem. Lett.* **2018,** *9*, 557-562.

[15] Stress, C. J.; Sauter, B.; Schneider, L. A.; Sharpe, T.; Gillingham, D. *Angew. Chem. Int. Ed.* **2019,** *58*, 9570-9574.

[16] Dai, P.-F.; Qu, J.-P.; Kang, Y.-B. *Org. Lett.* **2019,** *21*, 1393-1396.

[17] Anderson, Z. J.; Hobson, C.; Needley, R.; Song, L.; Perryman, M. S.; Kerby, P.; Fox, D. J. *Org. Biomol. Chem.* **2017,** *15*, 9372-9378.

[18] Voshavar, C.; Meka, R. C. R.; Samanta, S.; Marepally, S.; Chaudhuri, A. *J. Med. Chem.* **2017,** *60*, 1605-1610.

[19] Evans, V.; Mahon, M. F.; Webster, R. L. *Tetrahedron* **2014,** *70*, 7593-7597.

[20] Nakajima, M.; Oda, Y.; Wada, T.; Minamikawa, R.; Shirokane, K.; Sato, T.; Chida, N. *Chem. Eur. J.* **2014,** *20*, 17565-17571.

[21] Gavande, N.; Kim, H.-L.; Doddareddy, M. R.; Johnston, G. A. R.; Chebib, M.; Hanrahan, J. R. *ACS Med. Chem. Lett.* **2013,** *4*, 402-407.

[22] Freedy, A. M.; Matos, M. J.; Boutureira, O.; Corzana, F.; Guerreiro, A.; Akkapeddi, P.; Somovilla, V. J.; Rodrigues, T.; Nicholls, K.; Xie, B.; Jiménez-Osés, G.; Brindle, K. M.; Neves, A. A.; Bernardes, G. J. L. *J. Am. Chem. Soc.* **2017,** *139*, 18365-18375.

[23] Miyamura, H.; Choo, G. C. Y.; Yasukawa, T.; Yoo, W.-J.; Kobayashi, S. *Chem. Commun.* **2013,** *49*, 9917-9919.

[24] Reddy, D. N.; Thirupathi, R.; Tumminakatti, S.; Prabhakaran, E. N. *Tetrahedron Lett.* **2012,** *53*, 4413-4417.
[25] Müller, J.; Feifel, S. C.; Schmiederer, T.; Zocher, R.; Süssmuth, R. D. *ChemBioChem* **2009,** *10*, 323-328.
[26] Opalka, S. M.; Steinbacher, J. L.; Lambiris, B. A.; McQuade, D. T. *J. Org. Chem.* **2011,** *76*, 6503-6517.
[27] Delaney, J. P.; Brozinski, H. L.; Henderson, L. C. *Org. Biomol. Chem.* **2013,** *11*, 2951-2960.
[28] Aratake, S.; Itoh, T.; Okano, T.; Nagae, N.; Sumiya, T.; Shoji, M.; Hayashi, Y. *Chem. Eur. J.* **2007,** *13*, 10246-10256.
[29] Maegawa, T.; Otake, K.; Hirosawa, K.; Goto, A.; Fujioka, H. *Org. Lett.* **2012,** *14*, 4798-4801.
[30] He, C.; Gaunt, M. J. *Angew. Chem. Int. Ed.* **2015,** *54*, 15840-15844.
[31] Sellers, R. M. *Analyst* **1980,** *105*, 950-954.
[32] O'Sullivan, D. W.; Tyree, M. *Int. J. Chem. Kinet.* **2007,** *39*, 457-461.
[33] Fiorani, G.; Stuck, M.; Martín, C.; Belmonte, M. M.; Martin, E.; Escudero-Adán, E. C.; Kleij, A. W. *ChemSusChem* **2016,** *9*, 1304-1311.
[34] Lorenz, S.; Plietker, B. *ChemCatChem* **2016,** *8*, 3203-3206.
[35] Prat, I.; Font, D.; Company, A.; Junge, K.; Ribas, X.; Beller, M.; Costas, M. *Adv. Synth. Catal.* **2013,** *355*, 947-956.
[36] Hubbell, A. K.; LaPointe, A. M.; Lamb, J. R.; Coates, G. W. *J. Am. Chem. Soc.* **2019,** *141*, 2474-2480.
[37] Ankudey, E. G.; Olivo, H. F.; Peeples, T. L. *Green Chem.* **2006,** *8*, 923-926.
[38] Pouységu, L.; Chassaing, S.; Dejugnac, D.; Lamidey, A.-M.; Miqueu, K.; Sotiropoulos, J.-M.; Quideau, S. *Angew. Chem. Int. Ed.* **2008,** *47*, 3552-3555.
[39] Bew, S. P.; Hiatt-Gipson, G. D.; Mills, G. P.; Reeves, C. E. *Beils. J. Org. Chem.* **2016,** *12*, 1081-1095.
[40] Oberhauser, C.; Harms, V.; Seidel, K.; Schröder, B.; Ekramzadeh, K.; Beutel, S.; Winkler, S.; Lauterbach, L.; Dickschat, J. S.; Kirschning, A. *Angew. Chem. Int. Ed.* **2018,** *57*, 11802-11806.
[41] Moretti, R. A.; Du Bois, J.; Stack, T. D. P. *Org. Lett.* **2016,** *18*, 2528-2531.
[42] Sun, W.; Sun, Q. *Acc. Chem. Res.* **2019,** *52*, 2370-2381.
[43] Vicens, L.; Olivo, G.; Costas, M. *ACS Catal.* **2020,** *10*, 8611-8631.
[44] Katsuki, T.; Sharpless, K. B. *J. Am. Chem. Soc.* **1980,** *102*, 5974-5976.
[45] Gao, Y.; Klunder, J. M.; Hanson, R. M.; Masamune, H.; Ko, S. Y.; Sharpless, K. B. *J. Am. Chem. Soc.* **1987,** *109*, 5765-5780.
[46] Zhang, W.; Basak, A.; Kosugi, Y.; Hoshino, Y.; Yamamoto, H. *Angew. Chem. Int. Ed.* **2005,** *44*, 4389-4391.
[47] Lichtor, P. A.; Miller, S. J. *Nat. Chem.* **2012,** *4*, 990-995.
[48] Nobuta, T.; Kawabata, T. *Chem. Commun.* **2017,** *53*, 9320-9323.
[49] Chin, K. F.; Ye, X.; Li, Y.; Lee, R.; Kabylda, A. M.; Leow, D.; Zhang, X.; Xia Ang, E. C.; Tan, C.-H. *ACS Catal.* **2020,** *10*, 2684-2691.
[50] Garcia, M.-A.; Méou, A.; Brun, P. *Synlett* **1996,** *1996*, 1949-1050.
[51] Gaikwad, R. D.; Kabiraj, S. S.; Bhat, S. V. *Flavour Fragr. J.* **2016,** *31*, 350-355.
